# A Web Application About Herd Immunity Using Personalized Avatars: Development Study

**DOI:** 10.2196/20113

**Published:** 2020-10-30

**Authors:** Hina Hakim, Julie A Bettinger, Christine T Chambers, S Michelle Driedger, Eve Dubé, Teresa Gavaruzzi, Anik M C Giguere, Éric Kavanagh, Julie Leask, Shannon E MacDonald, Rita Orji, Elizabeth Parent, Jean-Sébastien Paquette, Jacynthe Roberge, Beate Sander, Aaron M Scherer, Martin Tremblay-Breault, Kumanan Wilson, Daniel Reinharz, Holly O Witteman

**Affiliations:** 1 Department of Family and Emergency Medicine Laval University Quebec City, QC Canada; 2 Vaccine Evaluation Center, BC Children’s Hospital University of British Columbia Vancouver, BC Canada; 3 Department of Psychology and Neuroscience and Pediatrics Dalhousie University Halifax, NS Canada; 4 Department of Community Health Sciences University of Manitoba Winnipeg, MB, Canada Winnipeg, MB Canada; 5 Institut national de santé publique du Québec Institut national de santé publique du Québec Quebec City, QC Canada; 6 Department of Developmental Psychology and Socialization University of Padova Italy Padova Italy; 7 École de design, Édifice La Fabrique Laval University Quebec City, QC Canada; 8 Faculty of Medicine and Health Susan Wakil School of Nursing and Midwifery University of Sydney Sydney Australia; 9 Faculty of Nursing University of Alberta Edmonton, AB Canada; 10 Faculty of Computer Science Dalhousie University Halifax, NS Canada; 11 University Health Network, Toronto General Hospital Eaton Building Toronto, ON Canada; 12 Department of Internal Medicine University of Iowa Iowa, IA United States; 13 Department of Medicine Bruyere Research Institute and Ottawa Hospital Research Institute University of Ottawa Ottawa, ON Canada; 14 Department of Social and Preventive Medicine Laval University Quebec City, QC Canada

**Keywords:** community immunity, herd immunity, vaccination, vaccine hesitancy, avatar, web application

## Abstract

**Background:**

*Herd immunity* or *community immunity* refers to the reduced risk of infection among susceptible individuals in a population through the presence and proximity of immune individuals. Recent studies suggest that improving the understanding of community immunity may increase intentions to get vaccinated.

**Objective:**

This study aims to design a web application about community immunity and optimize it based on users’ cognitive and emotional responses.

**Methods:**

Our multidisciplinary team developed a web application about community immunity to communicate epidemiological evidence in a personalized way. In our application, people build their own community by creating an avatar representing themselves and 8 other avatars representing people around them, for example, their family or coworkers. The application integrates these avatars in a 2-min visualization showing how different parameters (eg, vaccine coverage, and contact within communities) influence community immunity. We predefined communication goals, created prototype visualizations, and tested four iterative versions of our visualization in a university-based human-computer interaction laboratory and community-based settings (a cafeteria, two shopping malls, and a public library). Data included psychophysiological measures (eye tracking, galvanic skin response, facial emotion recognition, and electroencephalogram) to assess participants’ cognitive and affective responses to the visualization and verbal feedback to assess their interpretations of the visualization’s content and messaging.

**Results:**

Among 110 participants across all four cycles, 68 (61.8%) were women and 38 (34.5%) were men (4/110, 3.6%; not reported), with a mean age of 38 (SD 17) years. More than half (65/110, 59.0%) of participants reported having a university-level education. Iterative changes across the cycles included adding the ability for users to create their own avatars, specific signals about who was represented by the different avatars, using color and movement to indicate protection or lack of protection from infectious disease, and changes to terminology to ensure clarity for people with varying educational backgrounds. Overall, we observed 3 generalizable findings. First, visualization does indeed appear to be a promising medium for conveying what community immunity is and how it works. Second, by involving multiple users in an iterative design process, it is possible to create a short and simple visualization that clearly conveys a complex topic. Finally, evaluating users’ emotional responses during the design process, in addition to their cognitive responses, offers insights that help inform the final design of an intervention.

**Conclusions:**

Visualization with personalized avatars may help people understand their individual roles in population health. Our app showed promise as a method of communicating the relationship between individual behavior and community health. The next steps will include assessing the effects of the application on risk perception, knowledge, and vaccination intentions in a randomized controlled trial. This study offers a potential road map for designing health communication materials for complex topics such as community immunity.

## Introduction

### Background

*Herd immunity* or *community immunity* refers to the reduced risk of transmission of infection among susceptible individuals in a population through the presence and proximity of immune individuals. Community immunity (the term we use throughout this paper) works by breaking the chain of transmission and decreasing the probability of contact with an infectious agent, thereby preventing the spread of infectious agents in susceptible populations [1,2]. High vaccination coverage is generally needed to achieve this protection at the population level [3]. Decisions not to vaccinate affect population-level vaccine coverage and can result in outbreaks of vaccine-preventable diseases by pushing the vaccine coverage rate below the community immunity threshold [4-6].

Although some research suggests that people’s immunization decisions are primarily influenced by perceived benefits and harm at the individual level rather than those at the community level [7], other studies have suggested that improving the understanding of community immunity may lead to an increase in the intention to be vaccinated [8-10].

Community immunity is a challenging concept to convey. It depends on multiple factors, namely, vaccine effectiveness and coverage, whether or not susceptible individuals form clusters, timing of vaccine administration (ie, delayed vaccination results in longer periods of susceptibility and therefore increased likelihood of infection), and the presence or absence of serotype replacement [11]. It is also affected by historical rates of vaccination coverage where there are potential immunity gaps among people in specific age groups (eg, adolescents and young adults for MMR [measles, mumps, and rubella] vaccines). Possibly because the interplay between all these variables is complicated, people demonstrate an uneven understanding of the connection between individual-level vaccination behavior and community-level risk and benefits [12].

A systematic review identified visualization as a promising avenue for communicating the complex concept of community immunity [13]. By visualization, we mean the visual presentation of data or information. Visualization is a powerful communication mechanism because it enables people to rapidly understand complex information [14]. In this paper, we use the term *visualization* to refer to a brief narrated animation about community immunity. We use the term *application* when referring to a complete web-based application, combining the visualization with an interactive section in which people make their own avatars.

### Objectives

In this study, we seek to iteratively develop an application about community immunity that would be understood by people with varying levels of education and to assess and optimize people’s cognitive and emotional responses to the application. Our focus included emotions because emotions influence people’s decisions [15-17], including health decisions [18-20], and specifically vaccination decisions [21,22].

Our study aims to determine (1) whether and how people attend to different visual elements to explain the concept of community immunity (what is community immunity and how it works), (2) whether these elements are understandable, and (3) whether people understand how community immunity safeguards people, especially vulnerable populations who cannot be vaccinated or who may not respond to vaccines owing to their age or suppressed immune system.

## Methods

### Ethics Approval and Consent to Participate

This project was approved by ‘Comité d’éthique de la recherche en sciences de la santé’ ethics committee of Laval University (approval no: 2017-137 R-2/15-07-2019). All participants provided written informed consent.

### Concept Map

Before designing the first prototype, our multidisciplinary team began by developing a concept map [23] of what the prototype should convey (Multimedia Appendix 1). Concept maps are defined as tools for organizing and representing knowledge [24] or a graphical representation of different concepts and the relationship between those concepts [25]. Our concept map was used to organize the underlying content presented in the visualization within three major themes: (1) community, (2) infection, and (3) vaccines. We expanded and refined the components of each theme throughout the study in response to participants’ feedback. The theme *community* included content about how a community is made up of individuals, including vulnerable people living among other individuals. The theme *infections* included content about how different pathogens cause different infections and spread at different rates. The theme *vaccines* included content about how effective vaccines may or may not be, how some vaccine effectiveness may wane over time, and how different diseases require different vaccine coverage to prevent the spread of infection and to create community immunity.

### Overall Approach

We developed our prototype application according to the concept map and predefined our communication goals for each element of the prototype. Each element of the prototype was linked to what it was intended to convey in the concept map, and what cognitive and/or affective (emotional) responses we aimed to evoke among participants. Across multiple iterative cycles (Multimedia Appendices 2-5), we then measured participants’ responses to assess the extent to which each element of our application met its associated communication goals. In each cycle, we further sought to understand participants’ needs, strengths, and limitations; observe how they attended to visual elements and colors; and identify potential improvements that could be made to the application.

### Framework

**Figure 1:**
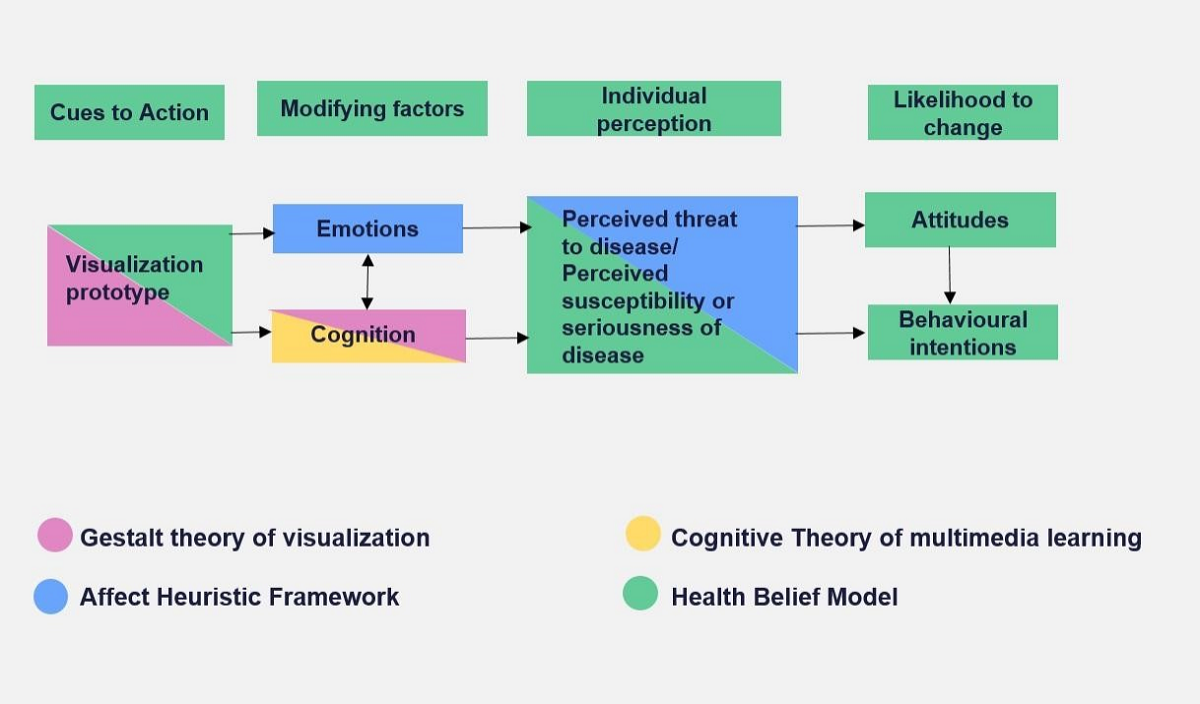
Conceptual frameworks used in this study and their relationship to the outcomes assessed.

To design our application and interpret people’s responses, we developed an integrated framework, as shown in Figure 1, combining four existing frameworks or models: (1) the *Health Belief Model* [26], (2) *Gestalt visual principles* [27], (3) the *Cognitive Theory of Multimedia Learning* [28,29], and (4) *Affect Heuristic* [30,31].

We selected the *Health Belief Model* [26] as the most likely framework to help us understand potential health behavior as a result of exposure to our intervention. This model has been developed and used to assess behavioral changes among people. However, this model hypothesizes that the intention or likelihood of an individual to take action stems from individual perception, and there is less detail regarding how such perceptions are shaped by different cues to action. We augmented this model to better understand the antecedents of perception by using *Gestalt visual principles* to inform the design of our visualization. Gestalt visual principles emphasize that the whole cannot be determined by simply knowing the individual pieces but emerges through how the pieces are combined or structured. These principles can be used to understand how the structure, configuration, or layout of elements in a visualization influence how people perceive the visualization. For example, the figure-ground principle describes how humans perceive objects or figures according to the contrast between elements and their backgrounds, and the proximity principle describes how images or figures located near each other are considered as a part of the same group, whereas objects apart are perceived as separate. Gestalt visual principles can thus help predict the effects of spacing, timing, and configuration when presenting information visually [32]. The *Cognitive Theory of Multimedia Learning* describes how people learn via two channels—auditory and visual—and use both together to build mental representations from words (audio) and images (visual) [28,29]. Finally, the *Affect Heuristic* provides an explicit framework for how the experiential system influences decisions via affect and emotions. The experiential system encodes reality in images, metaphors, and narratives, to which people have affective responses [31]. The Affect Heuristic helps structure analyses of emotions in response to the visualization.

Our guiding methodological framework was that of a user-centered design [33] in which potential users are consulted early and often, with their responses to prototype versions serving to help guide iterative improvements of the intervention or tool.

### Study Participants and Setting

Across all four study cycles, we recruited participants who were aged 18 years or older, had either no vision problems or corrected vision problems (eg, using eyeglasses or contact lenses), and were able to provide written informed consent, read and understand French or English, and use computers. In cycles 1, 3, and 4, we recruited participants to come to our university-based human-computer interaction laboratory by sending invitations to a university-wide listserv directed at all students, staff, and others. In cycle 2, we recruited participants in person by approaching them at a university-based cafeteria. In cycle 3, in addition to the listserv recruitment, we also recruited participants in person at a public library and two shopping malls located in areas of the city whose postal codes are associated with more diverse educational backgrounds. An incentive of either Can $10 (US $7.46; cycles 1, 2, 4) or Can $20 (US $14.92; cycle 3) was offered for their time and any transportation costs incurred. In cycle 3, we offered a larger incentive because, after viewing our visualization, participants subsequently interacted with materials developed for other studies, meaning that the individual sessions were of a longer duration.

### Psychophysiological Measurement

Design cycles 1, 3, and 4 used four psychophysiological data collection methods: eye tracking, galvanic skin response, electroencephalogram (EEG), and facial emotion recognition. We used eye tracking to determine what people were looking at and to measure participants’ visual attention [34]. We used galvanic skin response to determine when participants experienced peaks in emotional arousal [35]. Such peaks indicate instances of strong emotions. We expected the visualization to elicit strong emotions when, for example, something alarming happened, such as a vulnerable person getting infected with a contagious disease. We used facial emotion recognition software to assess emotional valence (ie, whether emotions were positive or negative) [36]. We expected participants’ emotions to be positive when the visualization depicted positive things happening, for example, community immunity being achieved and protecting community members, and to be negative when the visualization depicted negative things happening, for example, an infection spreading in the community. We used EEG to assess participants’ cognitive workload and engagement while looking at the information provided in the visualization [37]. We aimed for participants to experience higher engagement when interacting with the visualization without exceeding a cognitive load threshold above which they might be less likely to process new information.

### Apparatus and Procedures

**Figure 2:**
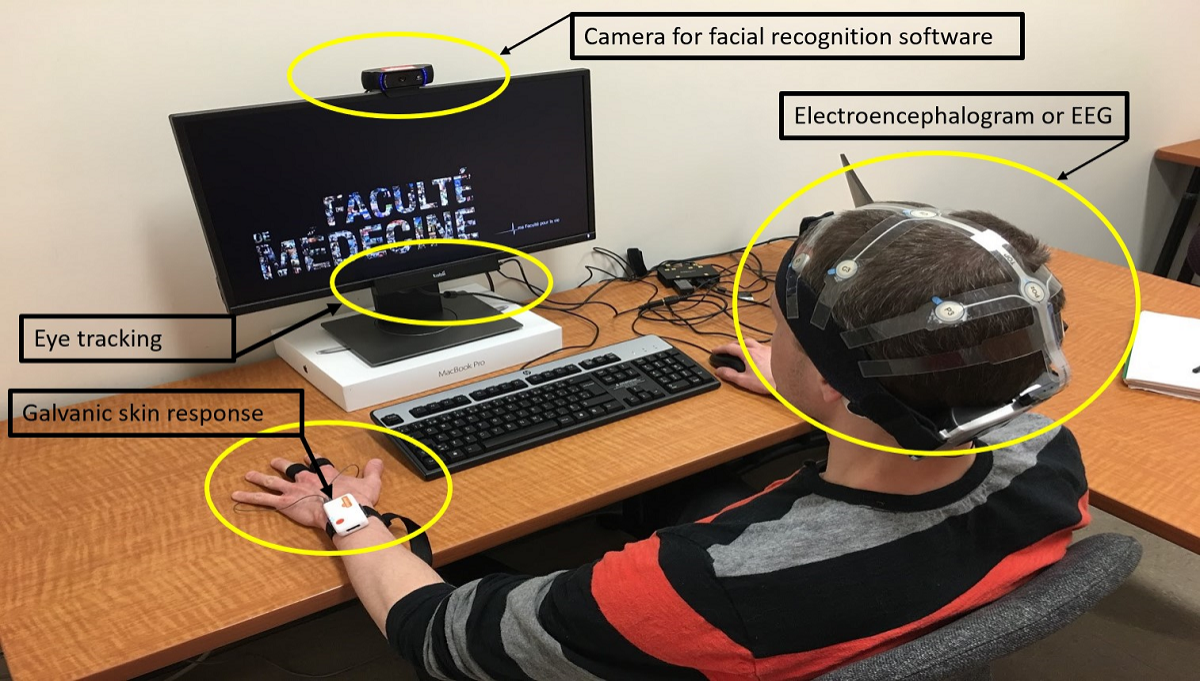
Human-computer interaction laboratory apparatus.

As shown in Figure 2, participants sat in a stationary chair in front of a desk with a mobile eye tracker (Tobii X2-30) and a webcam mounted on the computer monitor, a keyboard, a mouse, and computer speakers. A member of the research team explained each participant-worn device while placing it. These participant-worn devices were a portable galvanic skin response apparatus (Shimmer Sensing Shimmer3 GSR+) worn on the participant’s nondominant hand and an EEG (Advanced Brain Monitoring B-Alert X-Series) fitted on the participant’s head, using a gel on the electrodes. We followed standard procedures for each device’s calibration [38-41]. Data streams for all devices were synchronized and saved using the iMotions Attention Tool version 7 (cycle 1) or version 8 (cycles 3 and 4) [42].

### Verbal Feedback

We complemented psychophysiological data on participants’ nonverbal reactions with brief verbal feedback. Using semistructured interview questions (Multimedia Appendices 6-8), we asked participants to summarize in their own words what they saw in the visualization, what message it aimed to convey, and anything they found confusing or unclear. They were also asked questions about how to improve the visualization or personalized avatar building. If their explanation about the visualization indicated that they may have missed some visual elements, we probed for more specific information on how to improve those visual elements. We recorded responses using an audio recorder and took notes. Table 1 shows the summarized study design.

**Table 1:** Summarized study design.

Cycles	Study setting	Sample size	Method for data collection
First cycle	University-based human-computer interaction laboratory via university-wide listserv (email)	n=8	Psychophysiological measurement and verbal feedback
Second cycle	University-based cafeteria (by approaching them)	n=11	Verbal feedback
Third cycle	University sample: university-based human-computer interaction laboratory via university-wide listserv (email) *Community sample:* a public library and two shopping malls (by approaching them)	University sample: n=49 Community sample: n=34	Psychophysiological measurement and verbal feedback
Fourth cycle	University-based human-computer interaction laboratory via university-wide listserv (email)	n=8	Psychophysiological measurement (eye-tracking only) and verbal feedback

### Analysis

Our analytical aim was to assess whether the application achieved its communication goals. To analyze *psychophysiological measurements*, we examined participants’ reactions to each element according to its associated communication goal. We first identified the periods of each element in the visualization according to the voice-over timing. We assessed whether the participant was visually attending to each element by defining an area of interest for each element (eg, a rectangular region around a symbol) and examining whether the participant had any eye fixations of 200 ms or more in that area of interest. Fixations are described in the literature as lasting from 100 to 500 ms [43,44], 150 to 600 ms [45], or as low as 100 ms but *typically 200 to 600 ms* [46]. We selected 200 ms to maximize the likelihood of detecting fixations among people viewing a rapidly moving visualization while avoiding contaminating our data with shorter pauses in eye movement that might not indicate the person extracting any visual information. During the times when the element was present, we then also examined galvanic skin response, facial emotion, and EEG data as predefined for each communication goal. To analyze the galvanic skin response, we used an algorithm to detect peaks in arousal [47]. Previous literature suggests that this algorithm performs well in detecting such peaks [48,49]. To account for known lags in galvanic skin response (ie, the fact that skin response lags behind experience of heightened arousal by 3 to 5 s [50]), we inspected data for peaks in arousal during the defined time for each communication goal and for an additional 5 s afterward. The existence of such peaks would indicate that the participant experienced a heightened emotion of some kind while that element was displayed. For instance, in the first cycle, some participants showed a peak in arousal when the visualization showed vulnerable people getting infected. To analyze facial emotions, we used the facial recognition software FACET (Emotient) within the iMotions Attention Tool [51]. This software uses algorithms to translate the movement of facial features, such as eyes, eye corners, brows, mouth corners, and nose tip, into classifications of emotional valence. Recent work suggests that this automated facial-expression analysis software performs well for detecting emotional states [52,53]. We inspected the aggregated data for the number of occurrences across all respondents, and for any positive, negative, or neutral emotional valence elicited by the visualization. To analyze the EEG data, we used algorithms to estimate participants’ cognitive workload and engagement [39]. Cognitive workload indicates the extent to which working memory is being used. Engagement indicates a participant’s attentiveness while watching the visualization. Previous studies have validated these algorithms for measuring cognitive workload and engagement [54-56]. Cognitive workload is reported on a continuous scale from 0 to 1, with 0 to 0.4 classified as boredom, 0.4 to 0.7 as optimal workload, and 0.7 and above as information overload. Engagement levels are also reported on a continuous scale from 0 to 1, with 0 to 0.1 classified as sleepiness and drowsiness, 0.3 as distraction, 0.6 as low engagement, and 0.6 to 1 as high engagement. A summary score was computed by averaging values for each communication goal across all participants. For cycles with fewer than 10 participants, we examined emotional valence and EEG data at the individual level only. For cycles with 10 or more participants, to summarize data while continuing to weigh data from each participant equally, we calculated the mean valence, cognitive load, and engagement for each participant for each element, and then computed summary statistics and indices of dispersion across all participants. When these mean values were normally distributed across participants, our summary statistic was a global sample mean and our index of dispersion was a sample SD. When these mean values were skewed across participants, our summary statistic was a global sample median and our index of dispersion was an IQR. In addition to analyses by area of interest, we also inspected the heat maps of full screens. Heat maps are visual representations of data showing the relative intensity of participants’ visual attention to see where participants are looking at the most.

To analyze *verbal feedback,* two independent analysts (HH and EP) examined the responses independently and assessed the extent to which responses aligned with communication goals for each cycle by deductively comparing participant responses to our detailed concept maps. Any disagreement was resolved through discussion with the senior author (HW). We noted anything that failed to align with communication goals or was confusing to participants to guide changes for the next cycle.

After collecting data for each cycle, the first author (HH) compiled and reviewed data with coauthors (EP and MTB), summarized problems, and drafted recommendations. These recommendations were then discussed with the senior author (HW) and, when necessary, the larger team (remaining authors) to determine changes for the next cycle.

### Iterative Cycles

#### First Cycle

Our multidisciplinary team developed the first version of a visualization based on epidemiological evidence that we had organized in the concept map. We prespecified communication goals for different visual design elements (ie, what we wanted to convey with each element of the visualization and how we expected people to respond). We used four devices (Figure 2) and brief verbal feedback (audio-recorded) to assess participants’ interpretations and reactions to the content of the visualization. After viewing, the participants described the visualization in their own words. They were also asked the following questions: What do they think this visualization wants to convey? Is there anything in the visualization that they find unclear or confusing?

#### Second Cycle

We developed a revised version of the visualization based on participants’ feedback in the first cycle. We predefined our communication goals for the second cycle (Multimedia Appendix 9) and refined the concept map by adding how different diseases spread differently (pertussis, measles, and influenza as test case) and that different diseases require a different number of vaccine doses (eg, a single dose, multiple, booster, or annual doses). The visualization showed how different parameters (eg, vaccine coverage and intracommunity contact) can influence community immunity. We audio-recorded a brief verbal feedback.

#### Verbal Feedback

In this cycle, we only used audio-recorded verbal feedback (no psychophysiological measurements were used in this cycle [Table 1]) to assess participants’ interpretations of the visual content and their suggestions to improve it. We chose this method to increase the richness of verbal responses for each visual element. We asked participants to describe their understanding of the visualization, how vaccines work to protect people from diseases, what it means to be immune, and if there was anything confusing or unclear in the visualization. We showed images from the visualization to participants and asked specific questions (eg, what do the icons of the older woman and the baby represent in this visualization? What do the images of viruses causing different diseases represent?). We also asked participants about different terms used to explain community immunity, that is, herd immunity, community immunity, and community protection and which term they prefer.

#### Third Cycle

We developed a third version of a visualization based on participants’ feedback in the second cycle. We used the same techniques as in the human-computer interaction laboratory described earlier, along with verbal feedback. The third cycle was tested in two different settings: a university and different locations in a community setting (two shopping malls and a public library). We predefined the communication goals for the third cycle (see Multimedia Appendix 10 for university sample and Multimedia Appendix 11 for community sample). We asked participants to describe, in their own words, the visualization shown to them. We included a larger number of participants in this cycle, as our visualization was closer to launch and we wanted to make sure that it was easily understood and that people grasped the concept of community immunity. We also wanted to test if our communication goals were achieved among people with varied levels of education.

#### Fourth Cycle

By the fourth cycle, the content of our visualization had achieved nearly all predefined communication goals. However, one major issue remained. Up to this cycle, we had used generic avatars in our visualization. On the basis of data from previous cycles, we were concerned about the extent to which people could identify with the generic avatars presented in the visualization. Therefore, we developed an additional piece in which people were asked to build their own communities by making personalized avatars (their own, 2 vulnerable people in their community, and 6 avatars of people around them who could be family members or coworkers). We added this feature so that people could better identify with the avatars that were subsequently integrated into our application to explain community immunity. We asked participants to provide critical feedback on the process of creating their own avatars and building their own communities. In this cycle, we focused on three questions related to the new features: (1) Was an onboarding tutorial describing how to build avatars a useful addition? (2) Was it easy to build the avatars? and (3) Was the length of the avatar-building process reasonable? We further asked what participants thought of the avatar-building options, including the accessories and color palettes for skin tone and hair color. Participants also described the application in their own words. We only used the eye-tracking device in this cycle to assess visual attention.

## Results

### Study Participants

A total of 110 eligible participants across the four cycles (cycle 1 [n=8], cycle 2 [n=11], cycle 3 [n=83], and cycle 4 [n=8]) participated in the study (Table 2). Overall, 61.8% (68/110) of the participants were women and 34.5% (38/110) were men; 3.6% (4/110) did not report their gender. The mean age was 38 years (SD 17). Furthermore, 96.3% (106/110) of the participants spoke and understood French, 29.0% (32/110) spoke and understood English, whereas 3.6% (4/110) did not report the language spoken. More than half of the participants (65/110, 59.0%) had a university-level education. Most participants (85/110, 77.2%) reported no physical disability, 16.3% (18/110) reported some form of disability, and 2.7% (3/110) preferred not to answer. Across the 110 participants, 3 (2.7%) did not complete the sociodemographic questionnaire.

**Table 2:** Sociodemographics of each cycle.

Demographic characteristic		First cycle (n=8)	Second cycle (n=11)	Third cycle (university sample; n=49)	Third cycle (community sample; n=34)	Fourth cycle (n=8)	Across all cycles (N=110)
**Self-identified gender** **, n (%)**							
	Female	3 (38)	7 (64)	34 (69)	16 (47)	8 (100)	68 (62)
	Male	2 (25)	4 (36)	15 (31)	17 (50)	0 (0)	38 (35)
	Not reported	3 (38)	0 (0)	0 (0)	1(3)	0 (0)	4 (4)
Age (years), mean (SD)		28 (8)	24 (7)	37 (13)	52 (15)	26 (8)	38 (17)
**Language, n (%)**							
	French	5 (63)	11 (100)	48 (98)	34 (100)	8 (100)	106 (96)
	English	5 (63)	10 (91)	14 (29)	1 (3)	2 (25)	32 (29)
	Not reported	3 (38)	0 (0)	1 (3)	0 (0)	0 (0)	4 (4)
**Physical disability, n (%)**							
	Yes	0 (0)	0 (0)	9 (18)	9 (26)	0 (0)	18 (16)
	No	5 (63)	11 (100)	40 (82)	21 (62)	8 (100)	85 (77)
	Not reported	3 (38)	0 (0)	0 (0)	1 (3)	0 (0)	4 (4)
	Prefer not to answer	0 (0)	0 (0)	0 (0)	3 (9)	0 (0)	3 (3)
**Education level, n (%)**							
	Some elementary School	0 (0)	0 (0)	0 (0)	4 (12)	0 (0)	4 (4)
	High school diploma	0 (0)	0 (0)	2 (4)	9 (26)	1 (13)	12 (11)
	College or polytechnic school certificate or diploma (CÉGEP^a^, AEC, DEC)	1 (13)	4 (36)	8 (16)	6 (18)	3 (38)	22 (20)
	University graduate degree (bachelor’s)	1 (13)	2 (18)	14 (29)	9 (26)	1 (13)	27 (25)
	University graduate degree (master’s)	3 (38)	5 (45)	20 (41)	1 (3)	3 (38)	32 (29)
	University graduate degree (doctorate)	0 (0)	0 (0)	5 (10)	1 (3)	0 (0)	6 (5)
	Do not know	0 (0)	0 (0)	0 (0)	0 (0)	0 (0)	0 (0)
	Prefer not to answer	0 (0)	0 (0)	0 (0)	3 (9)	0 (0)	3 (3)
	Not reported	3 (38)	0 (0)	0 (0)	1 (3)	0 (0)	4 (4)

^a^Quebec educational level requiring 2 years of study after completion of grade 11. CÉGEP students are typically 17 to 19 years old, and students typically must complete CÉGEP to be admitted to university.

### First Cycle

#### Findings From This Cycle

We obtained psychophysiological data from 6 of 8 participants and qualitative verbal feedback from 8 of 8 participants. There were missing psychophysiological data for 2 participants because of technical issues with the devices. Specifically, we had problems initializing the EEG.

As described in Table 3, the design elements of the visualization achieved their communication goals to varying degrees. All participants (8/8) reported that people in clusters of hexagons represented members of the community. Most participants (7/8) reported that a yellow background indicated vulnerable people, and 6 of 6 participants responded psychophysiologically in desired ways, that is, peaks in arousal, and high engagement when a vulnerable person became infected. All participants (8/8) reported that red connecting lines represented the spread of infection. Half of the participants (3/6) did not visually attend to the appearance of a thick blue band indicating community immunity upon its appearance. When questioned about its meaning, most participants (6/8) reported that the blue band around vulnerable people meant community immunity, whereas 2 of 8 participants interpreted it as some sort of linkage between the older woman and the baby. All participants (8/8) explicitly mentioned in their explanation that when enough people were vaccinated, this created a protective barrier of community immunity to prevent the spread of infection. Overall, all participants had a neutral (6/6) facial expression when community immunity was explained in the visualization.

#### Changes for the Next Cycle

A number of aspects of the first version of the visualization needed improvement. First, only a few participants (1/6) visually attended to the appearance of the central avatar, and only half of the participants (4/8) reported that the central avatar represents them. Second, most people (5/8) did not understand that the avatars around them could be others they see often but who are not members of their immediate family, for example, coworkers. To address these two issues, for the next cycle, we presented the center avatar, immediate family members, colleagues, and other regular contacts in the same visual frame by zooming in and out. Third, participants showed either low engagement (2/6) or drowsy or unengaged (4/6) when an infection first entered the community. To address this, rather than having the infection simply appear, we used a red line to visually represent the entry and spread of infection in the community. Fourth, participants (8/8) suggested that the visualization came across as a simple promotion of immunization rather than explaining how community immunity works. Although these concepts are interrelated in the sense that community immunity requires sufficient numbers of people to be immunized, our goal was to explain community immunity. To address this issue, we increased the focus on community immunity in the narration of our visualization. In discussing this latter change among our team, we identified a need to test the terms herd immunity, community protection, and community immunity by asking participants in the next cycle about their reactions to each of the three terms. In our team discussions, we also identified the need for new visual elements about different viruses (using measles, pertussis, and influenza as examples) to explain in greater detail why different diseases require vaccine doses and schedules.

**Table 3:** The communication goals set for the first cycle of visualization.

S. no.	Design element or a concept	Message design elements intended to convey in the visualization (desired interpretation and/or reaction)	What users reported when viewing these design elements (verbal feedback; n=8)	How users reacted to these design elements (psychophysiology; n=6)

1.	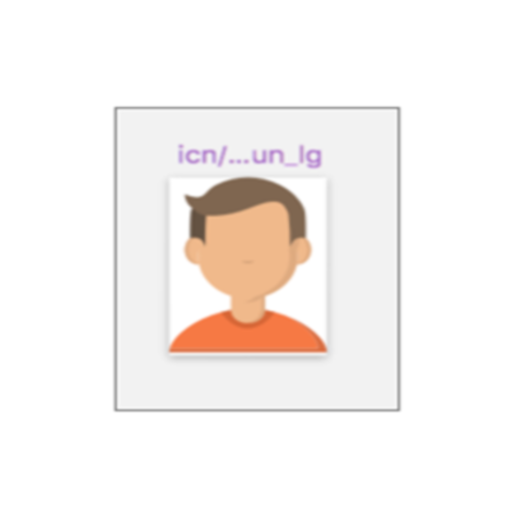	The avatar represents the participant.	Of 8 participants, 4 reported that the avatar represents them. The other 4 participants interpreted it as representing a person, but not them.	Of 6 participants, 1 visually attended to the appearance of the avatar. Overall valence was positive across the 6 participants.
2.	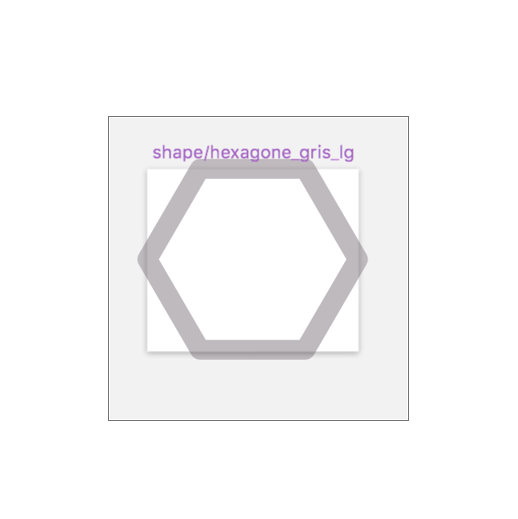	The hexagonal shape represents a unit.	Of 8 participants, 2 reported that each hexagonal shape was a separate unit. The other 6 participants interpreted it as an unspecified symbol or a honeycomb.	N/A^a^ (no psychophysiology data specific to this visual element).
3.	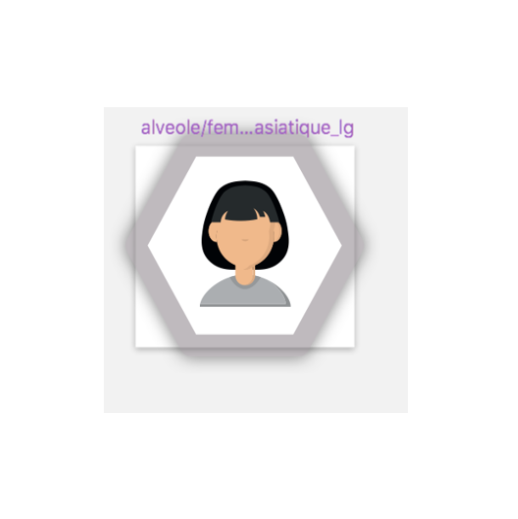	A person in a hexagonal shape around the central avatar represents the participant’s regular contacts (family members, friends, neighbors, or colleagues).	Of 8 participants, 3 reported that a person in hexagonal shape was a member of their community; 5 participants interpreted it as their family member.	N/A (no psychophysiology data specific to this visual element).
4.	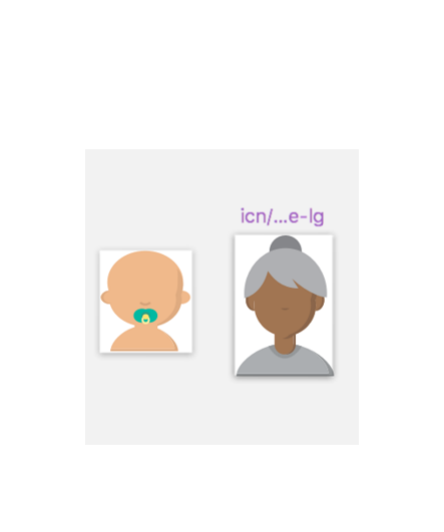	Icon of an older woman and a baby represents vulnerable people or those with fragile immune systems (eg, patients with cancer). High arousal and visual attention were expected when vulnerable people appeared in the visualization.	All participants (8/8) reported that an older woman and a baby in the visualization represent vulnerable people.	Of 6 participants, 4 visually attended when vulnerable people appeared in the visualization. Of 6 participants, 3 showed a peak in arousal when vulnerable people appeared.
5.	Yellow color behind *baby* and *an older woman*	Yellow color signals vulnerable people.	Of 8 participants, 7 reported that yellow color signals vulnerable people; 1 participant did not pay attention to the yellow color in the visualization.	N/A (no psychophysiology data specific to this visual element).
6.	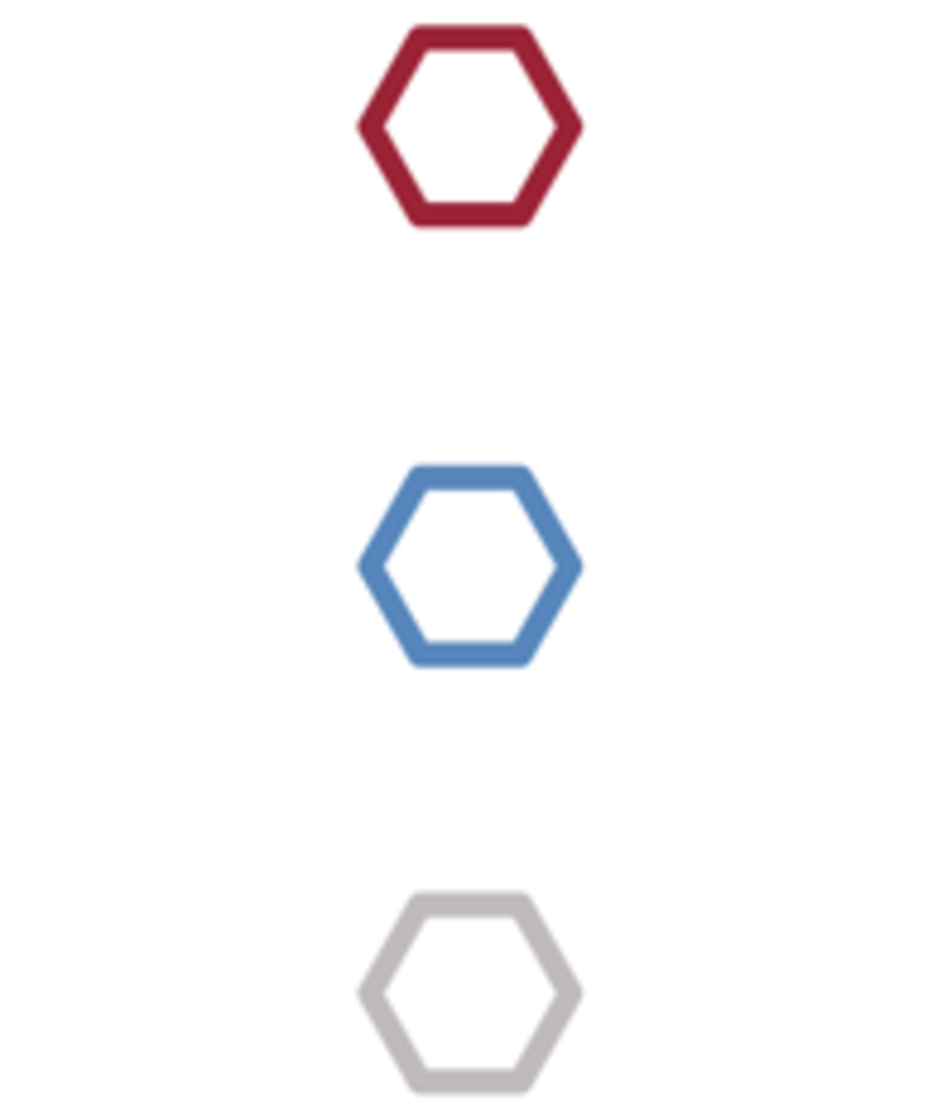	Red color signals diseased or infected; blue color signals vaccinated or protected; gray color signals susceptible to disease or infection	All participants (8/8) reported that the red color in the visualization represents disease, infection, or danger. All participants (8/8) reported that the blue color in the visualization signals being safe from diseases or vaccinated. Of 8 participants, 6 reported that gray color signals being susceptible to disease/infection or not vaccinated; 2 interpreted gray color as people who can be vulnerable.	N/A (no psychophysiology data specific to this visual element).
7.	When infection first enters the community.	High arousal, engagement, and visual attention were expected when the visualization shows when the infection first enters the community.	No comments recorded.	Of 6 participants, 3 visually attended when infection first entered the community. Of 6 participants, 2 showed a peak in arousal when infection first entered the community. No participants (0/6) were most likely to be in a high-engagement state when the infection first entered the community; 2 of 6 participants were most likely to be in a low-engagement state; 4 of 6 participants were most likely to be in a drowsy (unengaged) state.
8.	When the central avatar gets infected.	High arousal, engagement, and visual attention were expected when the visualization shows the central avatar representing the participant getting infected.	No comments recorded.	Of 6 participants, 1 visually attended when the avatar got infected. Of 6 participants, 4 showed peaks in arousal when the avatar got infected. Of 6 participants, 4 were most likely to be in a high-engagement state when the avatar got infected.
9.	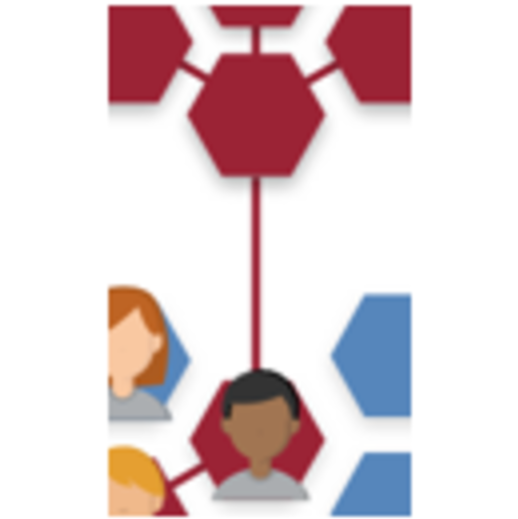	Red connecting lines represent the spread of infection. High arousal, engagement, and visual attention was expected when the visualization showed red connecting lines indicating the spread of infection.	All participants (8/8) reported that red connecting lines indicate the spread of infection.	Of 6 participants, 1 visually attended to red connecting lines. Of 6 participants, 1 showed peak in arousal when red connecting lines appeared. All participants (6/6) were most likely to be in a high-engagement state when red connecting lines appeared.
10.	When the vulnerable people get infected.	High arousal, engagement, and visual attention were expected when the vulnerable people got infected.	No comments recorded.	Of 6 participants, 3 visually attended when vulnerable people got infected. All participants (6/6) showed a peak in arousal and a negative valence when vulnerable people got infected. All participants (6/6) were most likely to be in the state of high engagement when vulnerable people got infected.
11.	When community immunity was explained	Participants’ explanations include the concept of community immunity. High arousal, visual attention, and positive valence was expected when the visualization demonstrated the concept of community immunity.	All participants’ (8/8) explanations include the concept of community immunity, that is what it is and how it works.	Of 6 participants, 4 visually attended when community immunity was explained. Of 6 participants, 4 showed peak in arousal when community immunity was explained. Overall facial expression was neutral across the 6 participants.
12.	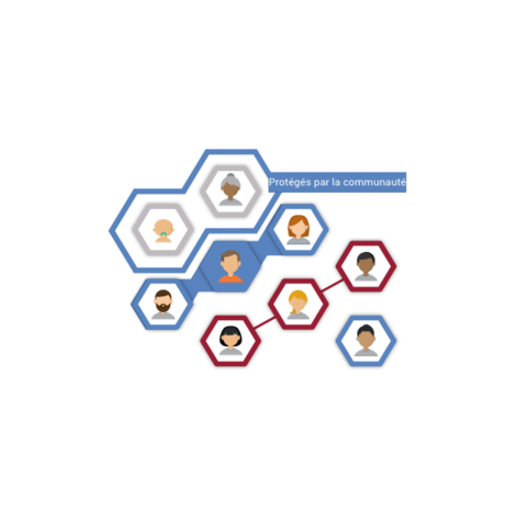	Thick blue band around vulnerable people indicates community immunity. High engagement and visual attention was expected when the thick blue band appeared around vulnerable people.	Of 88 participants, 6 reported that the thick blue band around vulnerable people represents community immunity, which protects them from getting infected.	Of 6 participants, 3 visually attended when the blue line appeared around vulnerable people. All participants (6/6) were most likely to be in a high-engagement state when the blue line appeared around vulnerable people.
13.	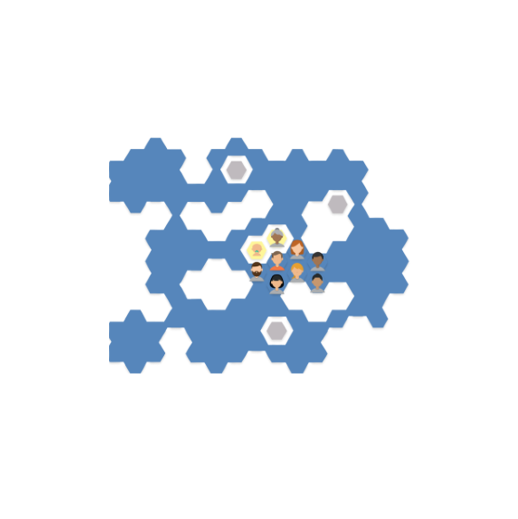	Blue lines spreading out from vaccinated people indicate the community immunity. High engagement was expected when blue lines appeared indicating the community immunity.	All participants (8/8) reported that blue lines spreading out from vaccinated people show the protective barrier that is community immunity.	All participants (6/6) were most likely to be in a high-engagement state when blue lines appeared indicating the community immunity.
14.	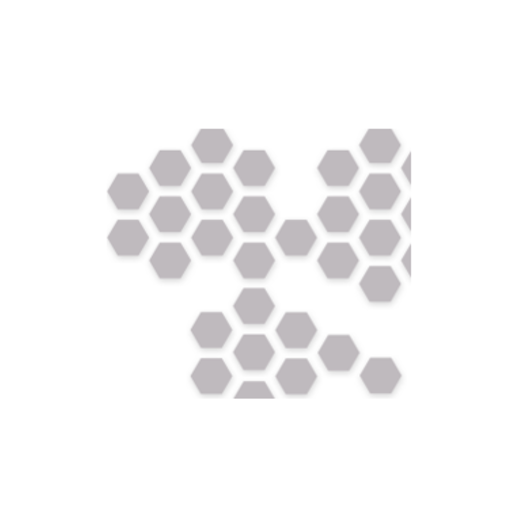	The cluster of hexagons represent different communities.	All participants (8/8) reported that clusters of hexagons represent different communities.	N/A (no psychophysiology data specific to this visual element).
15.	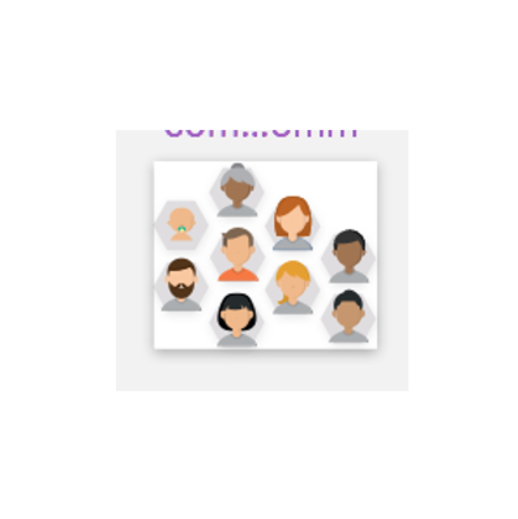	The avatar in the cluster of hexagons represents members of the community.	All participants (8/8) reported that the avatar in the clusters of hexagons represents members of the community.	N/A (no psychophysiology data specific to this visual element).
16.	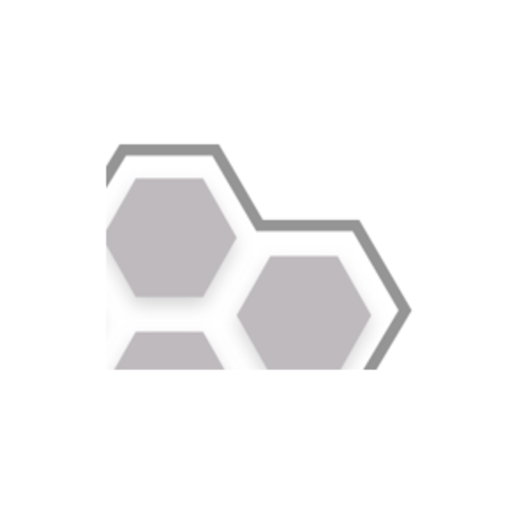	The gray outline around the cluster of hexagons indicates a group or members of the same community.	Of 8 participants, 6 reported that the gray outline indicates the group or members of the same community.	N/A (no psychophysiology data specific to this visual element).
17.	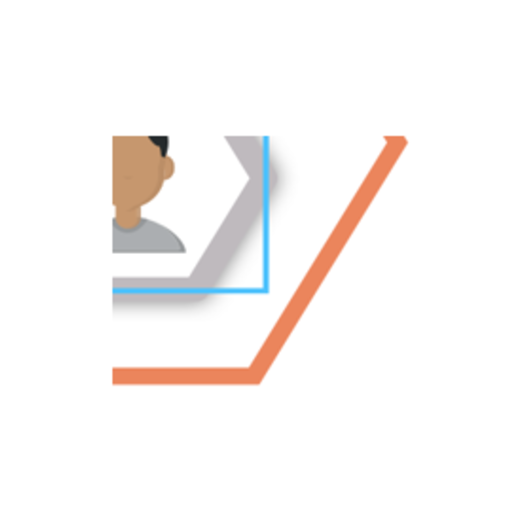	The orange outline showed the participant’s community. High engagement was expected when an orange outline appeared around their community.	Of 8 participants, 7 interpreted the orange outline as their community.	Of 6 participants, 3 visually attended when the orange outline appeared around their community. All participants (6/6) were most likely to be in a high-engagement state when the orange outline appeared around their community.

^a^N/A: not applicable.

### Second Cycle

#### Findings From This Cycle

The second version of the visualization achieved most of its communication goals. Multimedia Appendix 9 provides details analogous to those provided in Table 3 for the first cycle. All participants (11/11) reported that the people in the hexagon represent members of their community or people with whom they were in daily contact, the *older woman* and *the baby* in the visualization represented vulnerable members of the community, and the hexagons represented individuals. Most participants (7/11) reported that the visualization communicated that vaccines are not perfect, and nearly all reported that some vaccines require multiple doses or booster shots to work (10/11). All participants’ (11/11) responses showed that they understood the use of colors to signal vulnerability and infection such as a yellow background indicating vulnerable people, and that red color showed propagation of the disease. All participants (11/11) reported that community immunity safeguards vulnerable people, that is, when sufficient number of people around them were vaccinated, whereas lower vaccine coverage puts communities and the people within them, especially vulnerable populations, at risk of becoming sick. Participants indicated that the term *community immunity* best conveyed the concept compared with terms *herd immunity* (which implies herds of animals) or *community protection* (which participants indicated evoked images of protection via firearms.) Few (2/11) participants reported that the color blue indicated immunity, and none (0/11) showed understanding that the color gray indicated susceptibility to infection. Some participants reported that diseases differ (3/11) and spread at different rates (3/11). Few participants (2/11) reported vaccine-induced immunity, whereas none reported the concept of natural immunity (0/11). Some participants (3/11) reported the role of vaccine effectiveness in creating community immunity, whereas others did not.

#### Changes for the Next Cycle

Aspects of the visualization that needed to be improved included conveying that the color blue means being vaccinated or immune, the color gray means being susceptible, and focusing attention on the fact that different diseases spread at different rates. In addition, the visualization did not yet help participants understand the role of vaccine effectiveness in community immunity or distinguish between natural immunity and vaccine-induced immunity. Participants further suggested that the visualization was too long and provided too much information to retain. In the third cycle, we kept the colors blue and gray but explained their meaning in the narration. We removed the images representing different viruses but kept the narration explaining how different infections spread at different rates, illustrating it with infection spread. We further added depictions of different vaccine coverage for different diseases to show how community immunity prevents the spread of infection. We removed images illustrating natural and vaccine-induced immunity and different vaccine doses, and instead wove this information into the narration illustrated by a single image of immunity. We shortened the visualization for the next cycle to about 2 min and used the term community immunity in the narration.

### Third Cycle

#### Findings From This Cycle

The third cycle mostly achieved its communication goals (see Multimedia Appendix 10 for university sample and Multimedia Appendix 11 for community sample). A total of 83 participants (university sample: n=48; community sample: n=34) participated in our third cycle. Most participants (51/83, 61%) reported that the *older woman* and *the baby* represented vulnerable people or those with fragile immune systems (eg, patients with cancer). Most participants’ (60/83, 72%) verbal feedback summarizing the visualization included the point that vaccines prevent the spread of infection. Most participants (42/83, 50%) reported that community immunity safeguards everyone, some participants (34/83, 41%) reported that the thick blue band around an *older woman* and *the baby* demonstrated community immunity protecting vulnerable population and that an individual’s decision to get vaccinated or not has an impact on other people in their community (36/83, 43%). Most participants visually attended to all communication goals in a desired way; for example, nearly all participants (80/83, 96%) visually attended when the contagious disease spread to vulnerable people, when vaccines wane over time (74/83, 89%), and when community immunity safeguards everyone (81/83, 97%). Overall, across all 83 participants, people were likely in a state of high engagement and optimal workload during the explanation of how community immunity safeguards everyone.

#### Changes for the Next Cycle

Results from this cycle suggested that participants mostly understood the information presented in the visualization. However, some wording was unclear, so we made changes to the script to clarify it. For example, in the portion of the animation explaining how vaccines’ effectiveness wanes over time, we changed the script from, *They don’t work every time, and can wane over time*, to, *their protection can fade over time.* We also changed the order of some design elements to better align with how people appeared to understand the information during testing. For example, rather than first presenting how different diseases spread at different rates and then explaining community immunity, we changed these to present community immunity first, facilitating the explanation of why some diseases need more people to be vaccinated to create community immunity. Most importantly, until this cycle, we used generic avatars in the visualization. However, the generic avatars continued to be difficult for participants to interpret. To personalize the avatars so that people could better identify with them, we added a functionality so that people could build their own communities by making an avatar for themselves, 2 avatars for vulnerable people in their community, and 6 avatars of other people they see regularly, such as family members or coworkers. These personalized avatars were then integrated into our visualization to help participants better understand and respond emotionally to the idea of family members, friends, or other close contacts being vulnerable and at risk of infection.

### Fourth Cycle

We tested the last version of the application with 8 participants. All participants (8/8) reported what community immunity was and how it worked. Participants found the tutorial on how to create avatars confusing and preferred to make avatars by reading instructions. All participants (8/8) were able to easily create avatars by following step-by-step instructions, without a tutorial. All participants liked the color palettes for skin tone and hair colors. A participant suggested adding different accessories with options such as caps, hats, and a hijab to include more culturally diverse and realistic avatars.

The key findings of all cycles and major changes implemented are summarized in Table 4.

**Table 4:** Key findings of all cycles.

Cycles	Key communication goals achieved	Key communication goals not achieved	Summary of psychophysiological data (where applicable)	Summary of how issues were addressed in the next cycle
First cycle	• Nearly all participants reported that the color yellow represents vulnerable people. • Most participants reported that the blue band around vulnerable people meant protection. • All participants reported that the avatars in the cluster of hexagons represent members of the community.	• Most participants did not understand that the central avatar represents them. • Some participants did not understand that the avatars around them could include nonfamily contacts, for example, coworkers. • All participants understood the purpose of visualization as promoting immunization rather than explaining the concept of community immunity.	• Most participants visually attended to the appearance of vulnerable people. • Most participants had peaks in arousal and showed high engagement when avatars got infected. • All participants showed high engagement when red lines showed the spread of infection. • Most participants visually attended, and all participants had peaks in arousal and showed high engagement when vulnerable people got infected. • Most participants visually attended and had peaks in arousal when community immunity was explained. • All participants showed high engagement when blue lines around people spreading out from vaccinated people showed community immunity.	• We presented the center avatar, immediate family members, colleagues, and communities in the same visual frame by zooming in and out. • We removed the term immunization in the narration script and focused more on community immunity. • We decided to test the terms herd immunity, community protection, and community immunity by asking participants which term they relate to and prefer. • We added a question to be asked in the next cycle about the shape of hexagons presented in the visualization. • We added a new design element in the next cycle explaining how different viruses (eg, measles, pertussis, influenza) spread at different rates and require different vaccine schedules.
Second cycle	• All participants reported that yellow signaled vulnerability. • All participants reported that red signaled infection. • All participants reported that hexagons represent people. • Nearly all participants reported community immunity safeguards vulnerable people when sufficient people around them are vaccinated. • All participants preferred the term community immunity over herd immunity or community protection.	• Few participants reported that blue meant vaccinated or immune. • No participants reported that gray meant susceptible. • Most participants did not report that different diseases spread at different rates. • Most participants did not report the role of vaccine effectiveness in community immunity. • Few participants reported vaccine-induced immunity. • None of the participants reported accurate understanding of natural immunity. • All participants suggested that the visualization should be shorter.	• N/A^a^	• We removed images of viruses but retained in the narration explanation of how different infections spread at different rates. • We added images about different levels of vaccine coverage to achieve community immunity for different diseases. • We removed images about different vaccine doses and natural and vaccine-induced immunity. • We shortened the visualization to about 2 min and used the term community immunity.
Third cycle	• Most participants reported that the older woman and baby avatars represent vulnerable people or those with fragile immune systems. • Most participants reported that vaccines prevent the spread of infection. • Most participants reported that community immunity safeguards everyone. •Some participants reported that the thick blue band around an older woman and the baby shows community immunity protecting vulnerable populations. •Some participants reported that their decision to get vaccinated or not has an impact on other people in their community.	• Nearly all participants found it difficult to identify with the generic avatars.	• Some participants had peaks in arousal when the avatar first appeared. • Most participants had peaks in arousal when the vulnerable population was explained in the visualization. • Some participants had peaks in arousal when the infection first entered the community in the visualization. • Most participants visually attended when the community immunity was shown, along with how it safeguards everyone. • Overall, participants showed high engagement and an optimal workload throughout the visualization.	• We added a functionality for people to build their own avatars and their own communities.
Fourth cycle	• All participants reported an accurate understanding of what community immunity is and how it works. • All participants reported that community immunity safeguards vulnerable people and everyone in the community. • All participants reported that some infections spread faster and need enough people to get vaccinated to prevent the spread of infections. • All participants found it easy to create avatars by following instructions without a tutorial. • All participants liked the palettes for skin and hair colors.	• For all participants, the avatar creation tutorial was confusing. They preferred to make avatars just by reading the step-by-step instructions. • A participant suggested adding additional accessories such as caps, hats, hijab, and other head and hair coverings.	• Nearly all participants visually attended to the avatar creation elements, including written instructions.	• Head and hair covering options (caps, hats, hijab, turban) were added.

^a^N/A: not applicable.

In Table 4, we summarize quantitative findings by referring to all participants when 100% of participants exhibited this; nearly all: 80% to 99%; most: 50% to 79%; some: 25% to 49%; few: 1% to 24%; no participants: 0%.

## Discussion

### Principal Findings and Comparison With Previous Literature

Considering our study as a whole, we observed three principal findings. First, visualization does indeed appear to be a promising medium for conveying what community immunity is and how it works. Our project builds on the limited previous literature on visualization to convey the concept of community immunity. On the basis of our systematic review [13], Betsch et al [10] are the only team to have developed and evaluated such an interactive visualization. Their visualization increased vaccination intentions and demonstrated the promise of this medium for conveying the concept of community immunity. We built on this by adding personalization to increase the user’s identification with the avatars, a voice-over to increase learning, especially among people with lower literacy, and a focus on the protection of vulnerable members of a community. In addition to previous research by Betsch et al [10,57], other studies have also pointed to the potential advantages of using visualization and videos to convey the concept of community immunity [58,59].

Second, our study shows that by involving users in iterative cycles during the design process, it is possible to create a relatively short and simple visualization that conveys a mathematically complex topic. This aligns with previous literature suggesting that visualizations can support people in understanding complex concepts [60-62], and users’ involvement in the design process can facilitate an understanding of the information [63].

Finally, our study shows that considering emotion during the design process can help inform the final design of the intervention. Emotions play an important role in health decision making [17], especially when deciding on behalf of loved ones [64], as is the case when deciding about vaccinating one’s children [65]. To the best of our knowledge, no previous study has considered emotions in developing a tool to explain community immunity [13]. In keeping with the Affect Heuristic within our framework, our study explicitly considered emotion, as expressed in verbal feedback and measured with psychophysiological data. According to Peters et al [66], affect has four possible functions in health communication and decision making. Affect can directly influence decisions according to a person’s subjective sense of the *goodness* or *badness* of options; it can function “as a spotlight” to direct a person’s attention toward information, which, in turn, shapes their judgments and decisions; it can motivate information processing and behavior; and it can help people trade-off between concepts that are difficult to compare directly. Because our application is designed primarily to convey a complex concept to inform decisions, we focused on affect’s function as a spotlight and adapted our application to better provoke emotional reactions to key information, such as the vulnerability of some community members. Attending to data about participants’ emotions throughout the design process therefore helped us carefully adapt our application to the way people perceive and use information to make health-related or other decisions.

### Limitations

This study has four main limitations. First, study participants were primarily French-speaking people in Quebec City, Canada, predominantly women, and many had a relatively high level of education. Our recruitment materials for different cycles mentioned that the study was about vaccination or health, which may have contributed to the over-representation of women in our university-based samples. Women seek health services more frequently than men [67] and are the overwhelming majority of participants in studies on childhood vaccine decision making [68]. To address this, in our largest cycle (third cycle), we expanded our recruitment strategy to include community-based settings. By deliberately recruiting a large subsample from a population that was more likely to include men and more likely to include people with lower levels of education, we were better able to ensure that the final design would be appropriate for a broad audience. However, despite our best efforts to diversify our study sample, our results may not be generalizable to other populations. Second, our application currently requires that users be able to visually perceive presentations on a screen. Further work will ensure accessibility for people who are blind or visually impaired. Third, building avatars and launching an application requires a certain level of computer literacy, meaning that the application will not necessarily serve people who are uncomfortable using or unable to use computers. Finally, the studies included people who were specifically recruited to participate in a study. It remains to be seen whether people are willing to view a 2-min visualization of community immunity outside of a study setting.

### Conclusions

Our application shows promise as a method of conveying the concept of community immunity to a broad range of members of the general public. This study has practical implications regarding how to design health communication materials about complex topics, such as community immunity, and other concepts that combine individual and population benefits and harms, such as antibiotic resistance, health resource allocation, and interventions during epidemics. Applications with personalized avatars may be more effective than abstract visual representations or text-based explanations to help people understand their personal role in population health. Further research could evaluate the specific effects of personalization. Our future work will test our application in a web-based randomized controlled trial to assess its effects on risk perception, knowledge, and vaccination intentions.

## Appendix

Multimedia Appendix 1 Concept map.

Multimedia Appendix 2 Script for 1st cycle.

Multimedia Appendix 3 Script for 2nd cycle.

Multimedia Appendix 4 Script for 3rd cycle.

Multimedia Appendix 5 Script for 4th or last cycle.

Multimedia Appendix 6 First cycle verbal feedback.

Multimedia Appendix 7 Second cycle verbal feedback.

Multimedia Appendix 8 Third cycle verbal feedback.

Multimedia Appendix 9 The communication goals set for the second iterative cycle of visualization.

Multimedia Appendix 10 The communication goals set for the third iterative cycle of visualization (university sample).

Multimedia Appendix 11 The potential communication goals set for the third iterative cycle of visualization (community sample).

## Abbreviations

EEG: electroencephalogram
