# Supplementary material for: A Web Application About Herd Immunity Using Personalized Avatars: Development Study
Source: J Med Internet Res. 2020 Oct 30;22(10):e20113. doi: 10.2196/20113 (PMC7665952; doi:10.2196/20113)
Supplement: Multimedia Appendix 2 [file jmir_v22i10e20113_app2.docx]

**Appendix 2: Script for cycle 1**

| **Liz** | **Visual** | **English narration** | **Narration française** |
| --- | --- | --- | --- |
| 001 | Introduction | What role do you play in protecting your community? | Quel rôle jouez-vous dans la protection de votre communauté? |
| Individual | | |  |
| 004 | 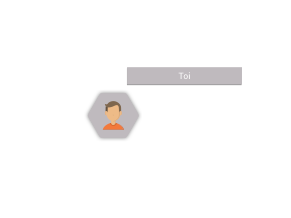 | Imagine this is you | Imaginez que c’est vous |
| 006 | 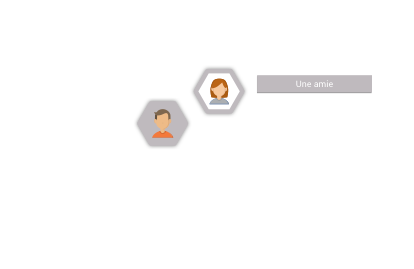 | This is a friend | Ceci est un ami |
| 008 | 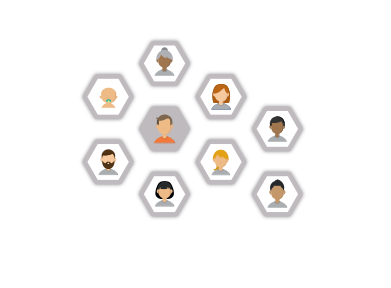 | These are your family members, friends, neighbours or colleagues. | Ici, ce sont les membres de votre famille, vos amis, vos voisins ou vos collègues de travail. |
| 010 | 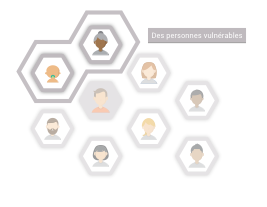 | There are people in your community who cannot be vaccinated because they are too young, too old, or they may have health problems; for example, they may be cancer patients. | Parmi ces gens, certains ne peuvent être vaccinés parce qu'ils sont trop jeunes, trop âgés ou parce qu’ils ont des problèmes de santé, par exemple, les patients atteints du cancer. |
| 011 | 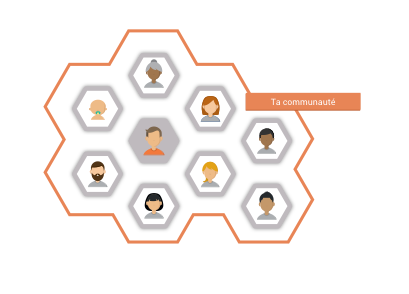 | Together you form a community | Ensemble, vous formez une communauté |
| 013 | 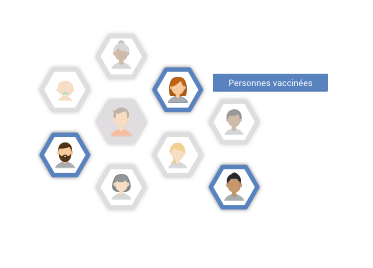 | Let’s see what happens when just a few people in your community get vaccinated. | Voyons ce qui se produit ~~lorsque~~ dans votre communauté lorsque quelques personnes sont vaccinées. |
| 015 | 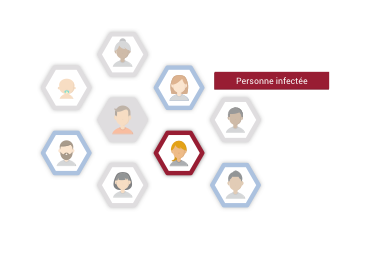 | When a disease or infection comes in your community | Lorsqu’une maladie ou une infection survient... |
| 017-022 | 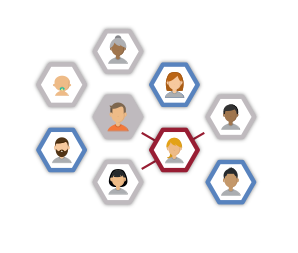 | It spreads from person to person in your community | elle se propage d'une personne à l'autre... |
| 022 | 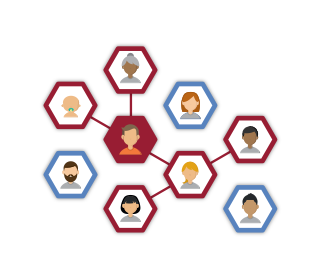 | And reaches vulnerable people who cannot get vaccinated. | et atteint les personnes vulnérables qui ne peuvent pas être vaccinées. |
| 024 | 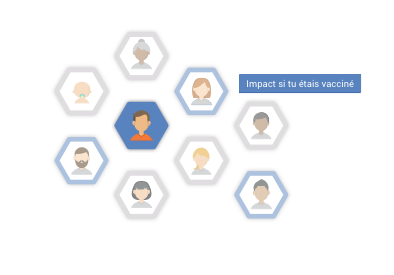 | But if enough people get vaccinated in your community. | Mais si, dans votre communauté, suffisamment de personnes sont vaccinées. |
| 024b | 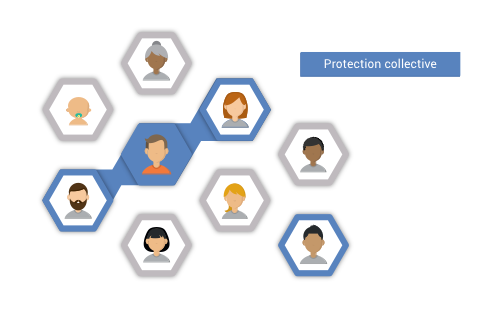 | This stops infections from spreading | Cela empêche les infections de se propager. |
| 024c | 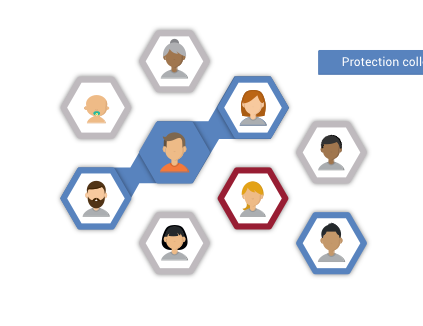 | So, when an infection comes in | Donc, lorsqu'une infection survient... |
| 026-028 | 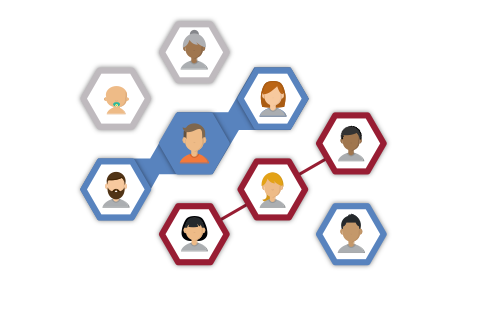 | And it tries to spread in your community, it can’t get through the protective barrier formed by people who got vaccinated. | et essaie de se répandre, elle ne peut pas traverser la barrière protectrice formée par des personnes qui ont été vaccinées dans votre communauté. |
| 030 | 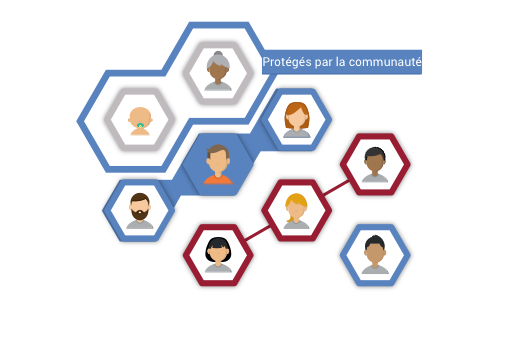 | Those of you who are vaccinated provide protection to vulnerable people in your community who cannot get vaccinated. This is called community protection. Community protection happens when enough people in a community get vaccinated. Otherwise there aren’t enough vaccinated people to create community protection, so the community is at risk of having a disease spread. | Les personnes vaccinées parmi vous fournissent une protection aux personnes vulnérables de votre communauté qui ne peuvent pas être vaccinées.  C'est ce qu’on appelle la protection communautaire.  La protection communautaire existe seulement lorsque suffisamment de personnes sont vaccinées.  S’il n'y a pas suffisamment de personnes vaccinées pour former la protection communautaire, la communauté est alors à risque qu’une maladie se propage. |
| Community. | | |  |
| 033 | 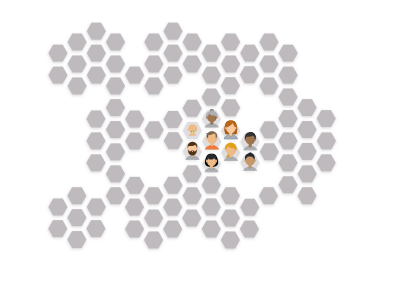 | What happens when your immediate community interacts with other communities? | Voyons maintenant ce qui arrive à votre communauté lorsqu’elle entre en contact avec d'autres communautés |
| 035 | 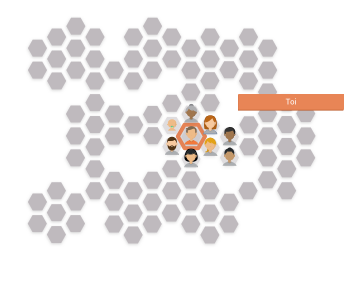 | Imagine this is you | Imaginez que c’est vous |
| 036 | 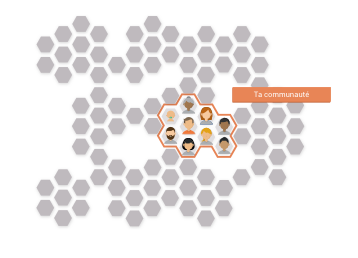 | And this is your community | Et ceci est votre communauté immédiate |
| 040 | 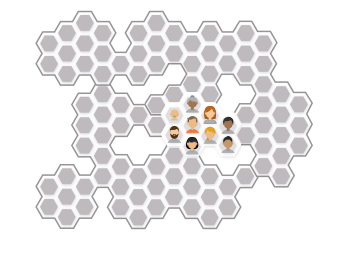 | These are other communities around yours, full of people who might share contact at work, at school, at the grocery store, or in any place where people interact. | Voici d'autres communautés près de la vôtre. Ces communautés sont composées de personnes que vous pourriez rencontrer au travail, à l'école, au supermarché ou à n'importe quel endroit où les gens coexistent. |
| 042 | 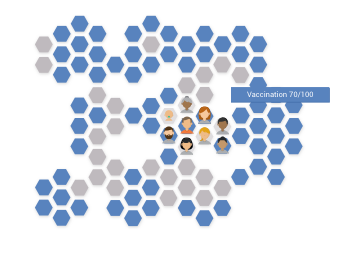 | So, when not enough people around your community get vaccinated | Si un nombre insuffisant de personnes autour de votre communauté est vacciné... |
| 042b | 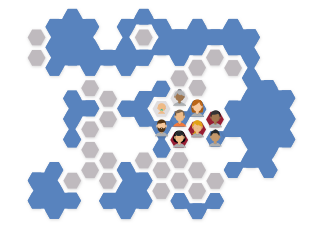 | This creates a fragmented community protection. | cela forme une protection communautaire fragmentée. |
| 044 | 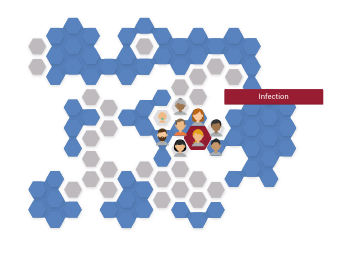 | So, when an infection comes in | Alors quand une infection survient... |
| 044-052 | 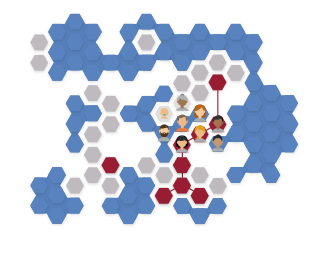 | It spreads | elle se propage |
| 052b | 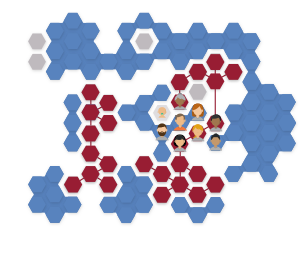 | and can reach vulnerable people. The community protection here wasn’t strong enough to protect them. | et peut atteindre les personnes vulnérables. La protection communautaire est trop divisée pour les protéger. |
| 056 | 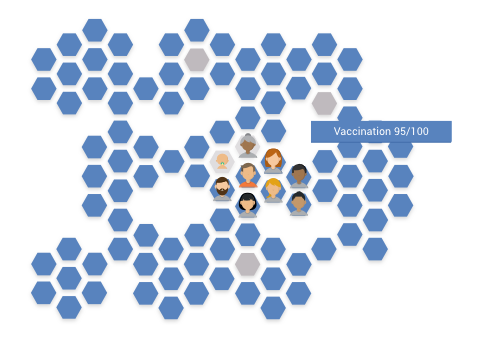 | But when enough community members are vaccinated | Mais lorsque suffisamment de membres de la communauté sont vaccinés |
| 056b | 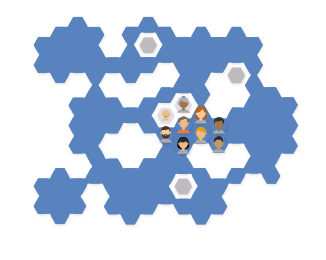 | it builds stronger community protection and prevents the spread of infection. | Il se construit une protection communautaire plus forte qui empêche la propagation de l'infection. |
| 058 | 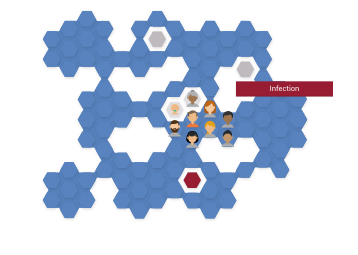 | So, if an infection comes in | Alors, si une infection survient |
| 058-060 | 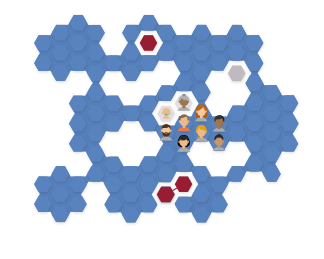 | And tries to spread | Et essaie de se propager |
| 060b | 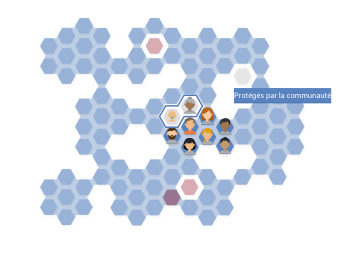 | Vaccinated members of communities protect vulnerable people through strong community protection. | Les personnes vaccinées dans les différentes communautés fournissent une protection communautaire aux personnes vulnérables. |
|  | | |  |
| ZZZ | Conclusion | Vulnerable people are members of our community. They have people who love them and who want them to stay healthy. They rely on the rest of us being vaccinated to provide community protection. | Les personnes vulnérables font partie intégrante de nos communautés.  Elles sont entourées de gens qui les aiment et qui veulent qu'elles restent en bonne santé.  Leur santé dépend du fait que le reste d’entre nous soit vacciné afin de créer la protection communautaire qui les gardera à l’abri des infections. |
