# Supplementary material for: A Web Application About Herd Immunity Using Personalized Avatars: Development Study
Source: J Med Internet Res. 2020 Oct 30;22(10):e20113. doi: 10.2196/20113 (PMC7665952; doi:10.2196/20113)
Supplement: Multimedia Appendix 3 [file jmir_v22i10e20113_app3.docx]

**Appendix 3: Script for cycle 2**

| **Visuel** | **English narration** | **Narration française** |
| --- | --- | --- |
| Introduction | What role does each of us play in protecting our community? | Quel rôle jouez-vous dans la protection de votre communauté? |
| Community | |  |
| 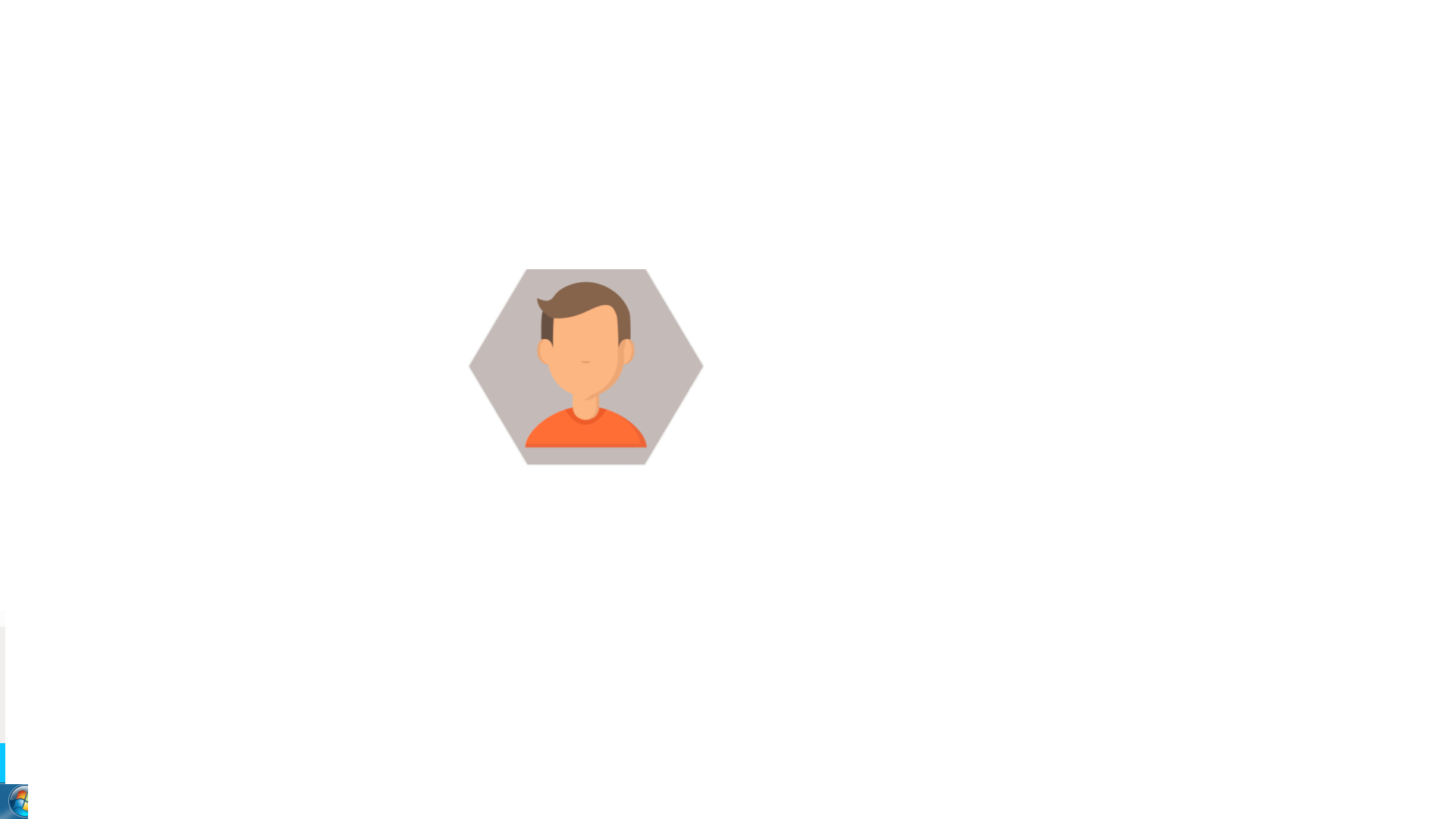 | Imagine this is you | Imaginez que c’est vous |
| 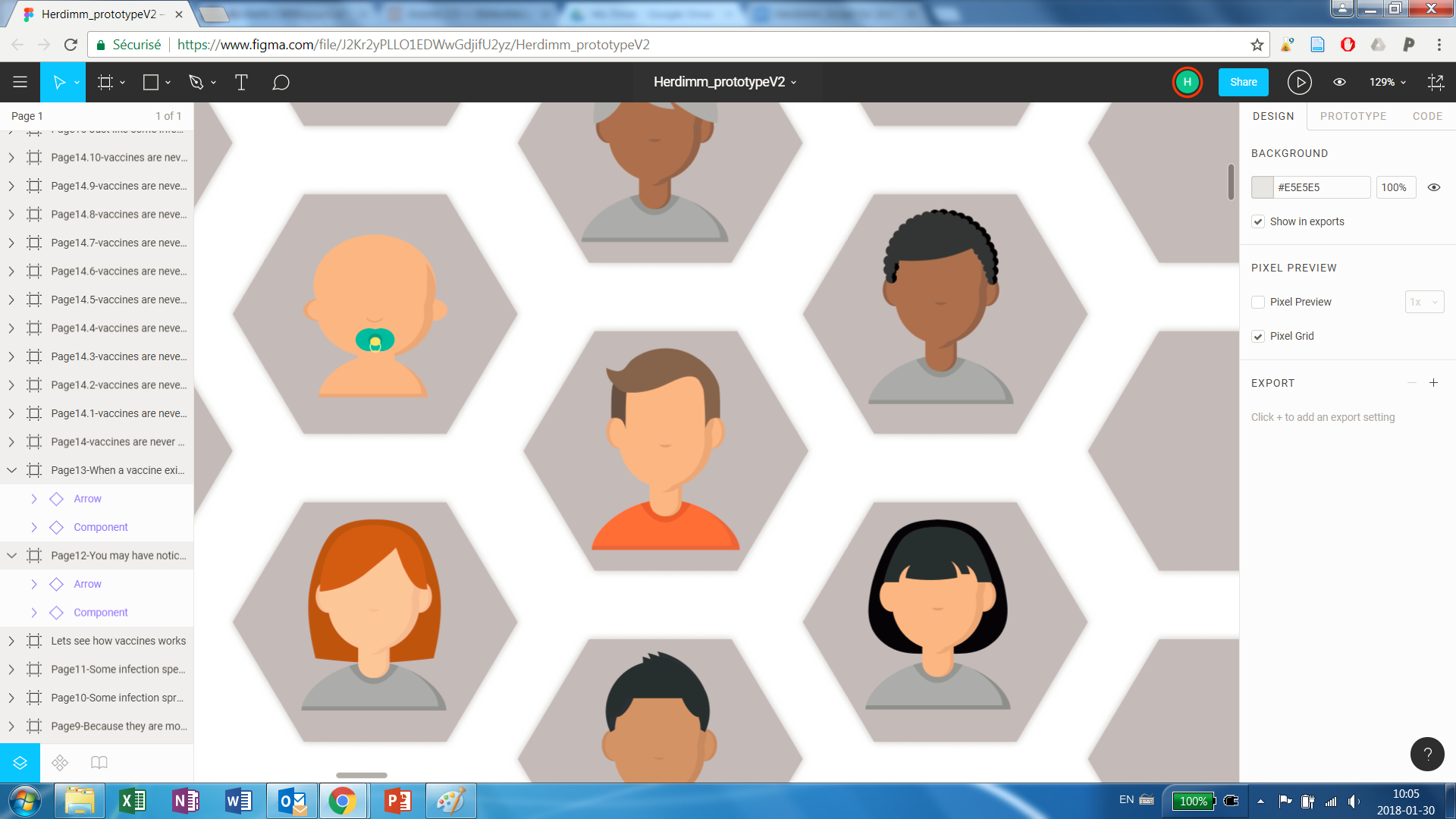 | These are people you are in contact with. | Voici des personnes que vous côtoyez tous les jours . |
| 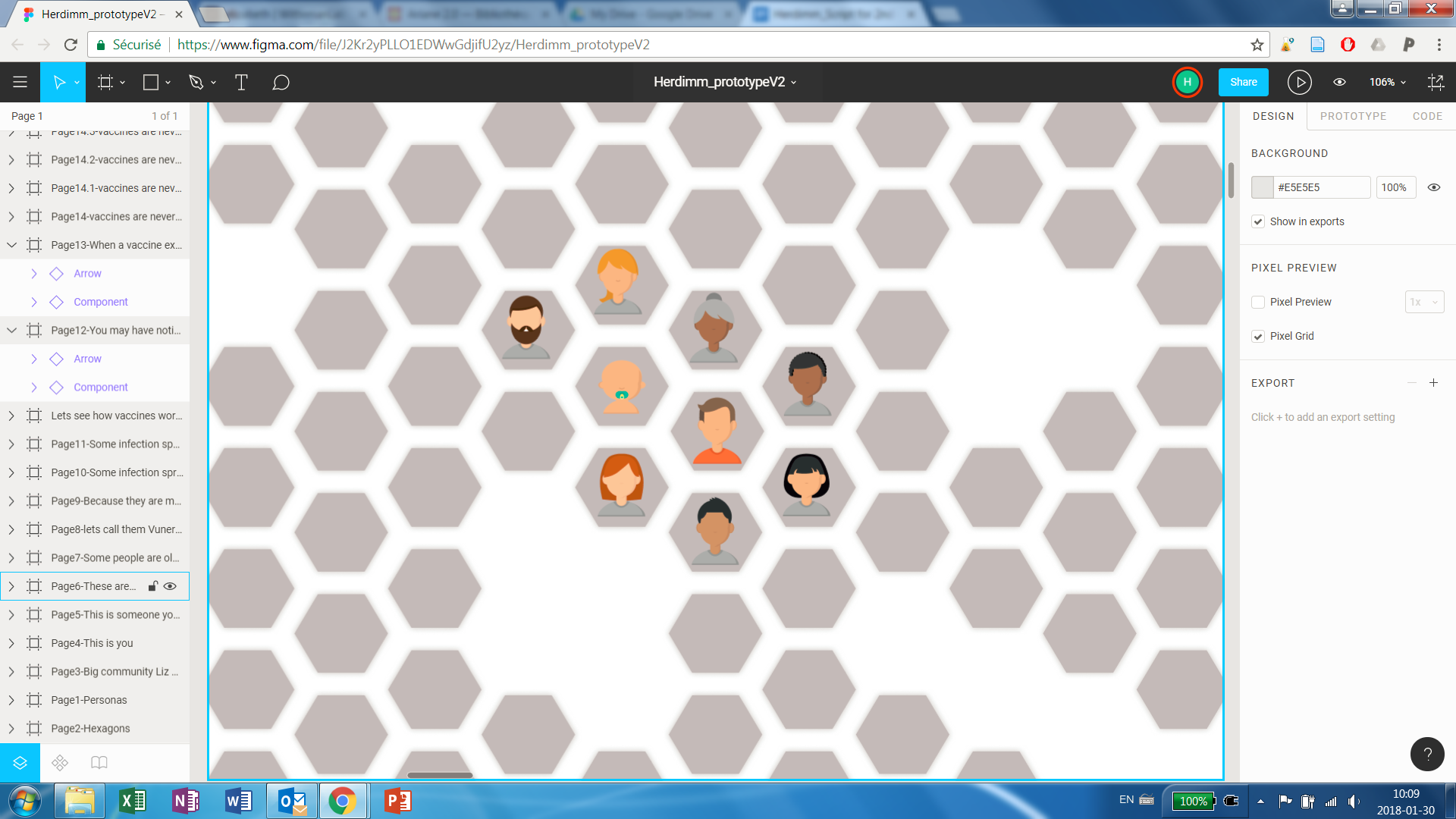 | These are other people who are in contact with each other. Together you form a community. | Ces personnes sont également en contact avec d’autres personnes. Cela forme une communauté. |
| 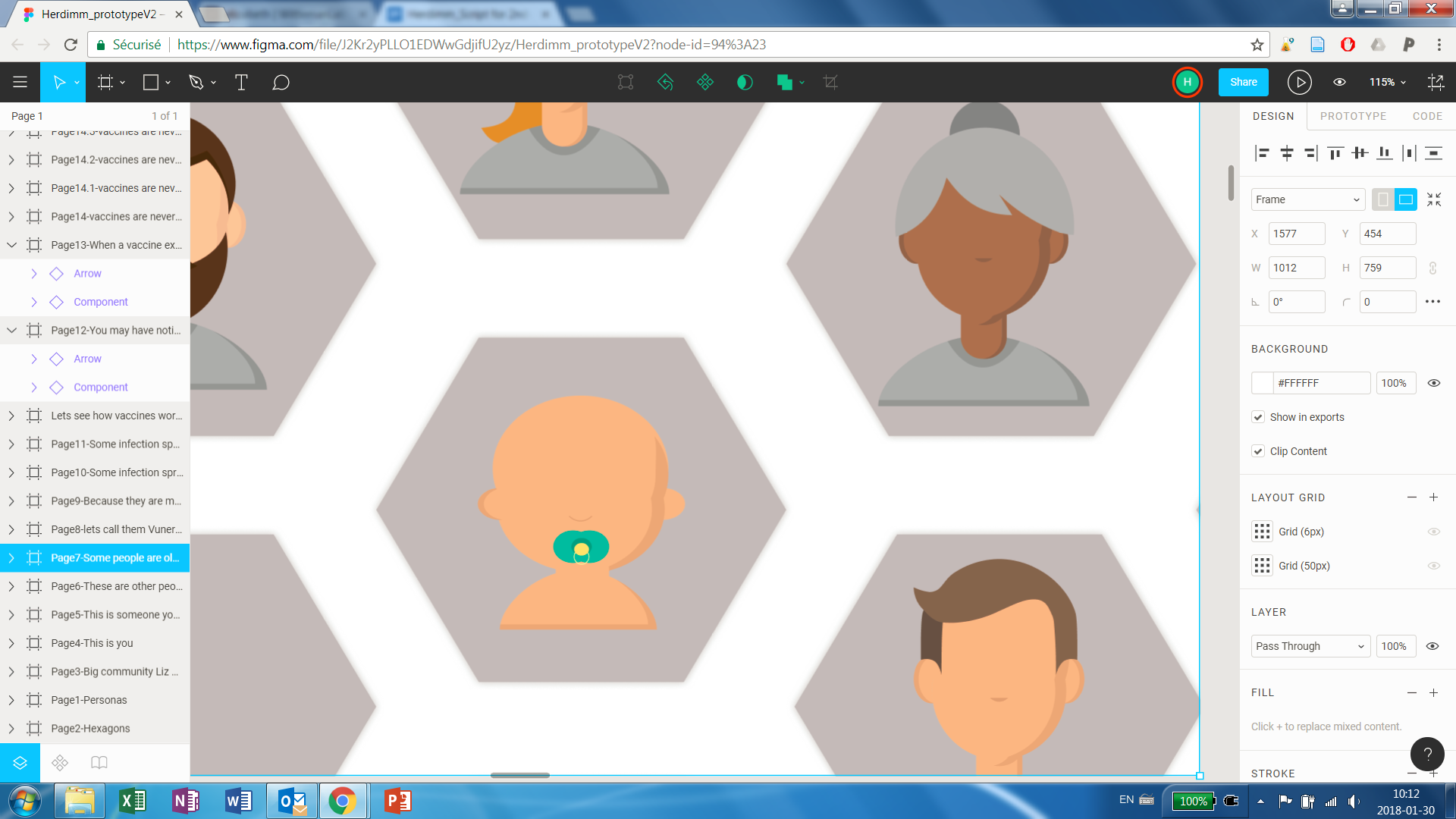 | There are people in your community who are younger, older, or sicker than others; for example, they may be babies, older people, or cancer patients. | Dans votre communauté, il y a des gens qui sont plus jeunes, plus vieux ou plus malades que les autres. Il s’agit, par exemple, de bébés de personnes âgées ou de patients atteints de cancer. |
|  | These are vulnerable people. | Ces personnes sont considérées comme vulnérables |
| 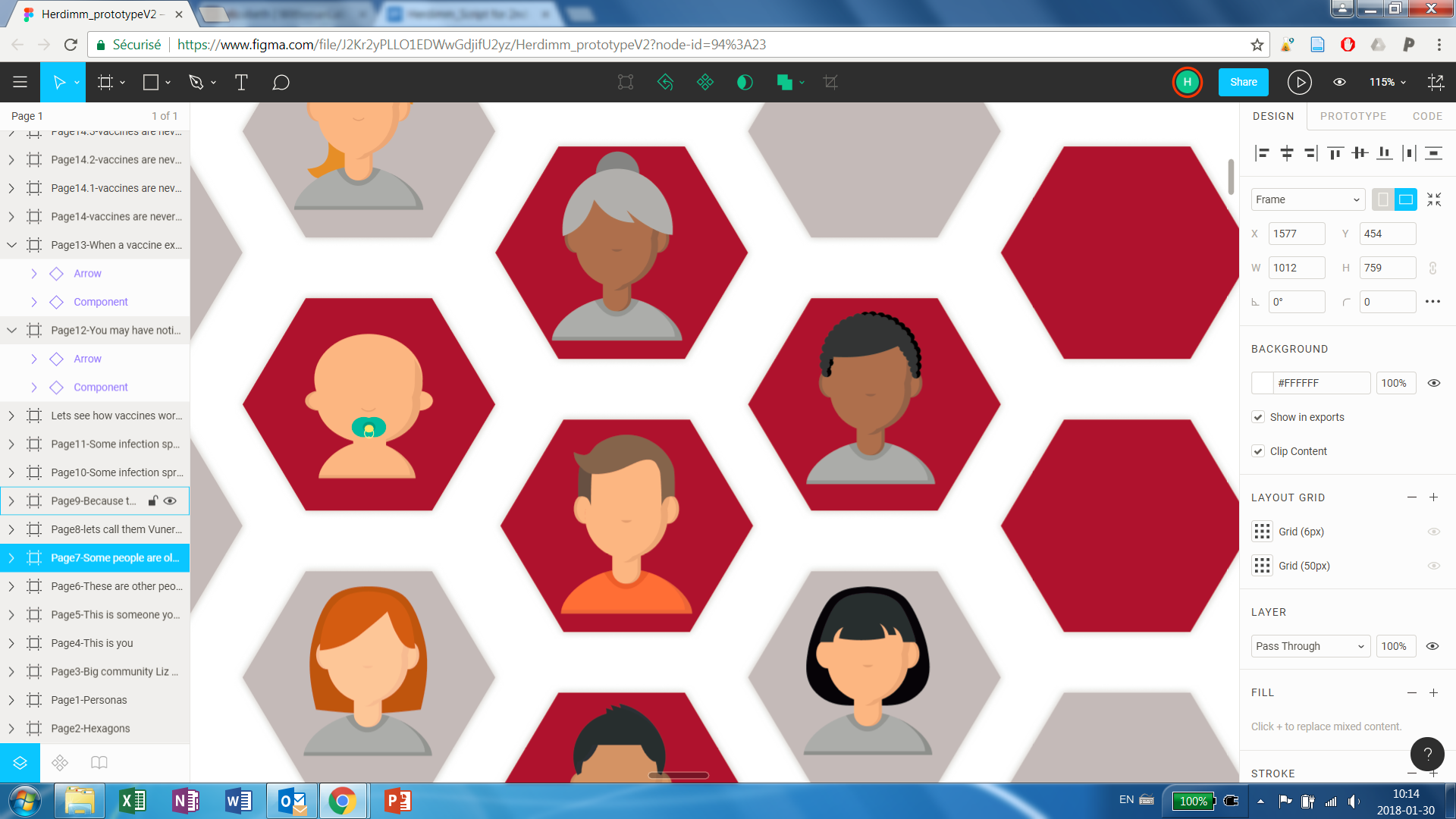 | When there is a contagious disease going around, anyone can get sick or even die from it. Vulnerable people are often more likely to get very sick or die. | Lorsqu’une maladie contagieuse se propage, n'importe qui peut tomber malade ou même en mourir. Les personnes vulnérables sont souvent plus susceptibles d'être très malades ou de mourir. |
| Infection/Disease | | |
| 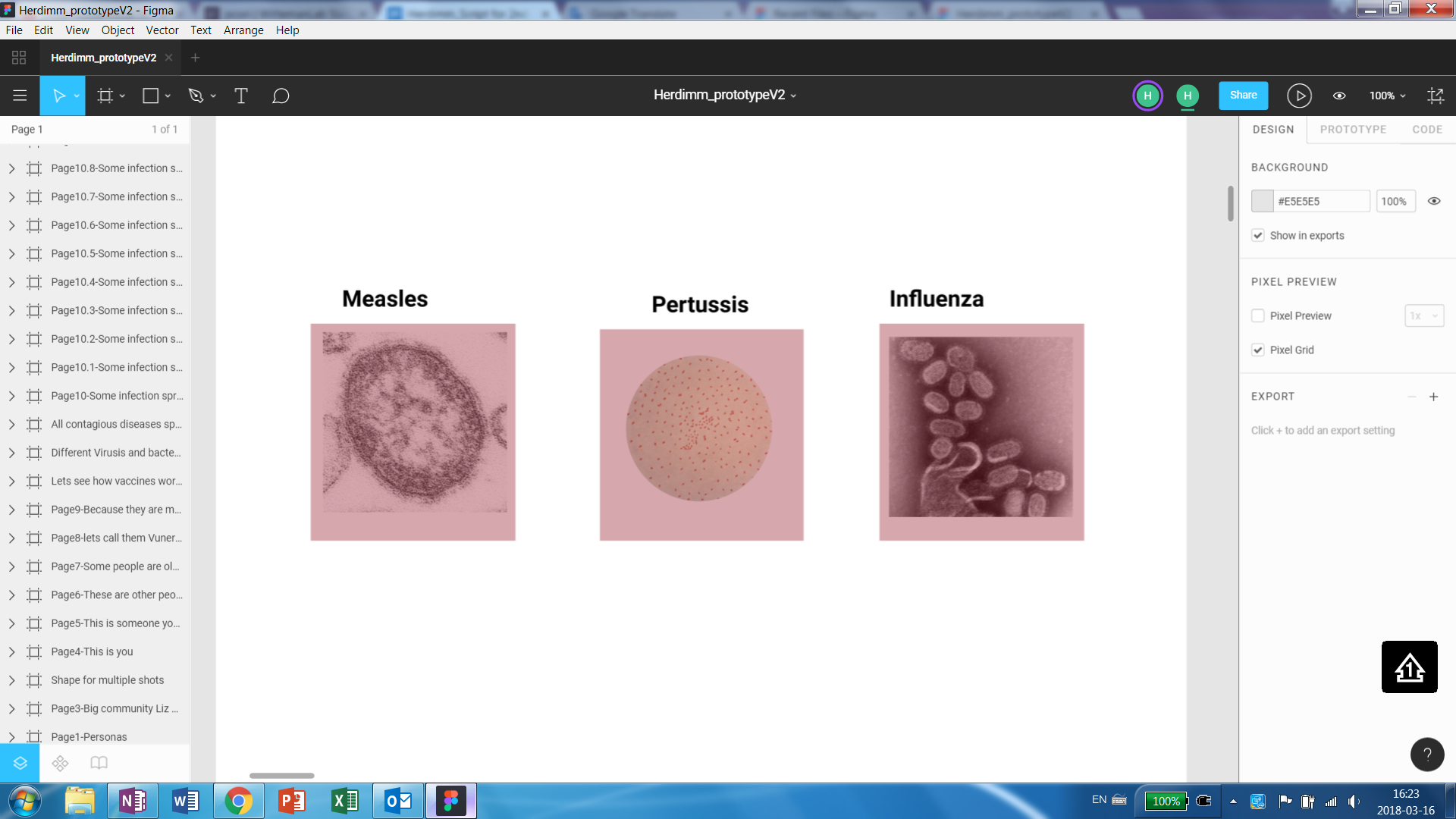 | The chances of this happening depend on different factors, including which disease it is. Different diseases are caused by different viruses or bacteria. For example, you can see here that measles, flu, and pertussis, or whooping cough, are different from each other. | Les chances que cela se produise dépendent de différents facteurs, par exemple, de quelle maladie il s'agit. Différentes maladies sont causées par différents virus ou différentes bactéries. Par exemple, vous pouvez voir ici que la rougeole, la grippe et la coqueluche sont différentes les unes des autres. |
| 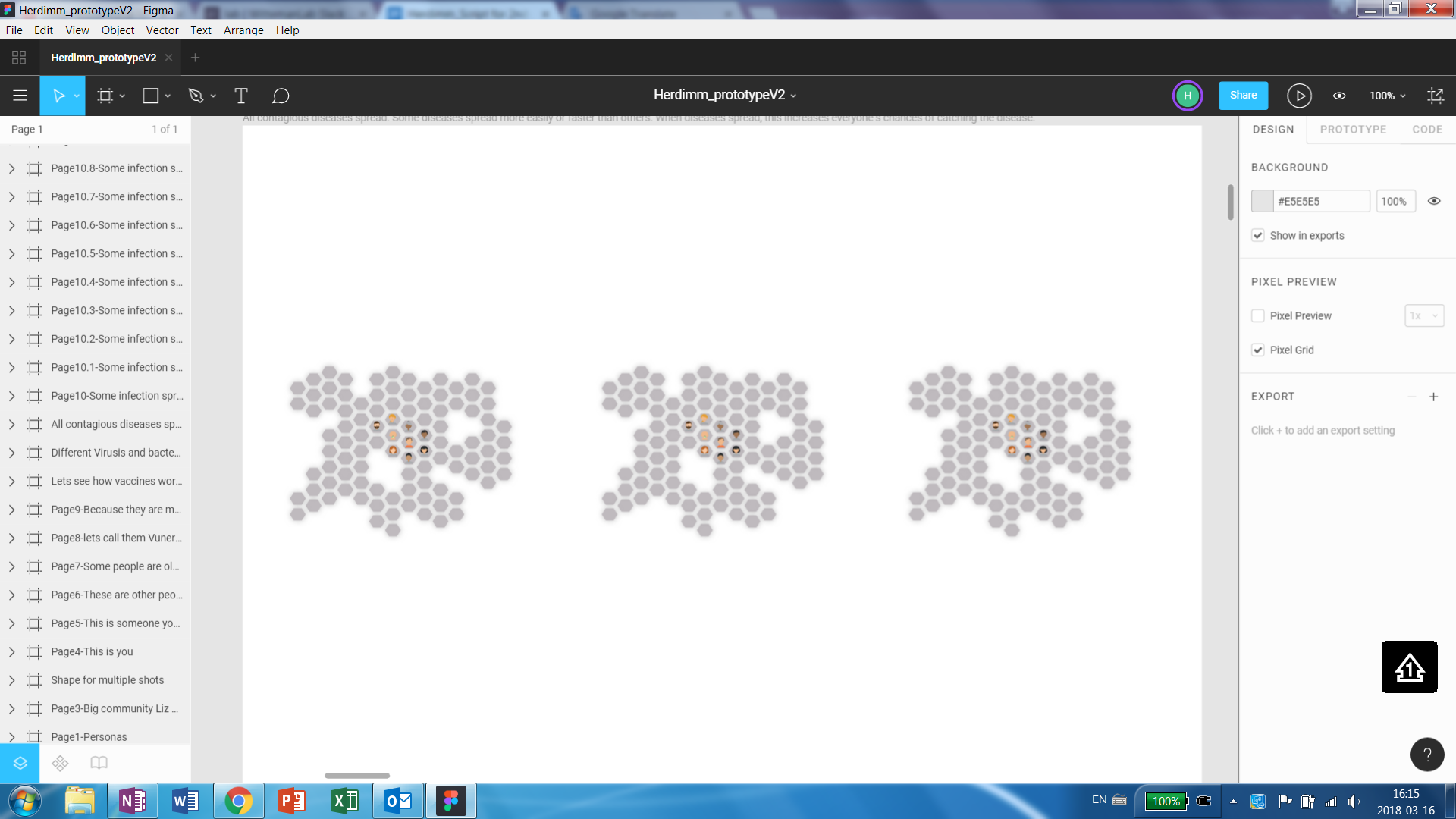 | All contagious diseases spread. Some diseases spread more easily or faster than others. When diseases spread, this increases everyone’s chances of catching the disease. | Toutes les maladies contagieuses se propagent. Certaines maladies se propagent plus facilement ou plus rapidement que d'autres. Lorsque les maladies se propagent, cela augmente les chances de tous d'attraper la maladie. |
| 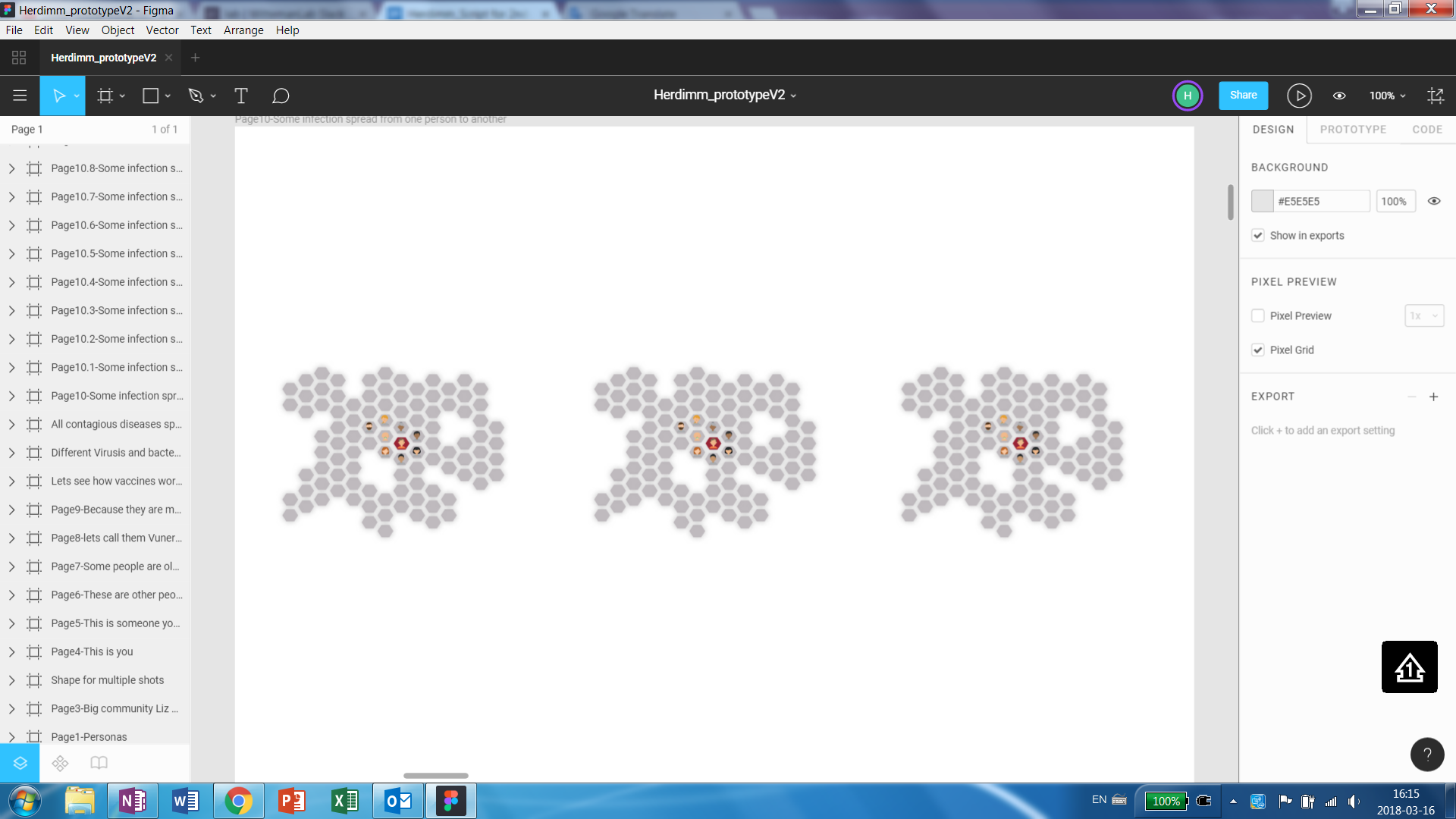 |  |  |
| 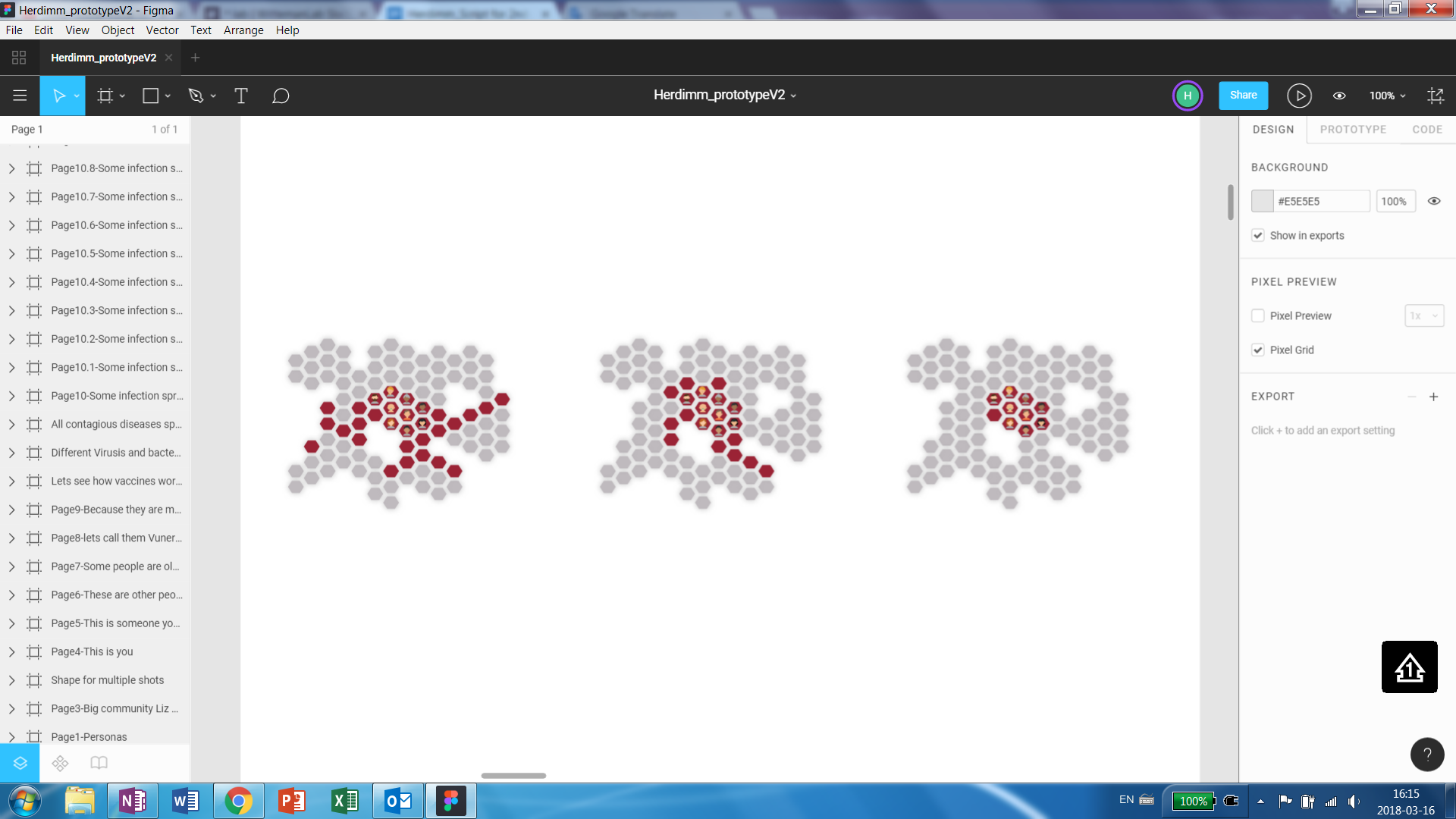 |  |  |
| 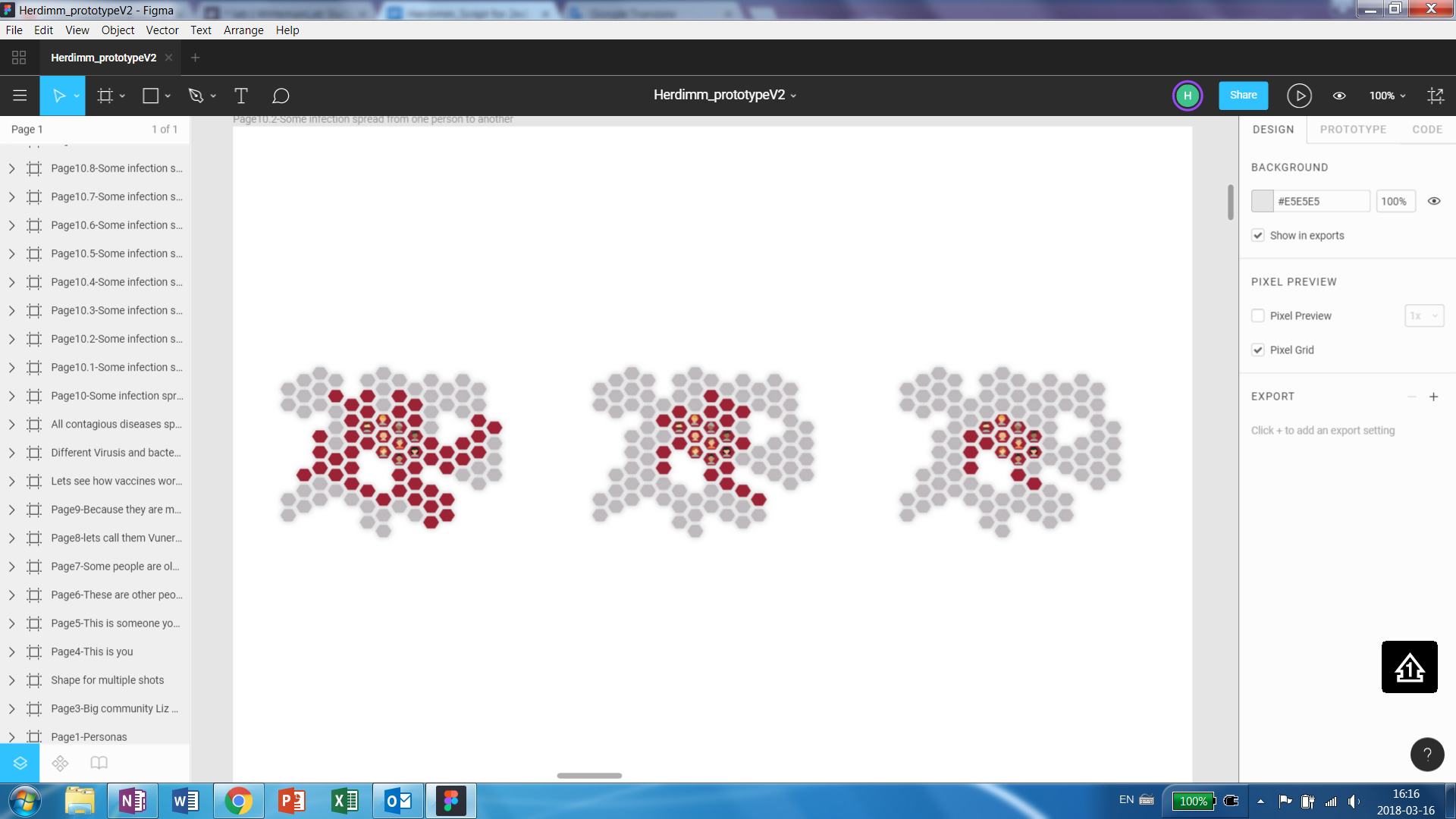 |  |  |
| 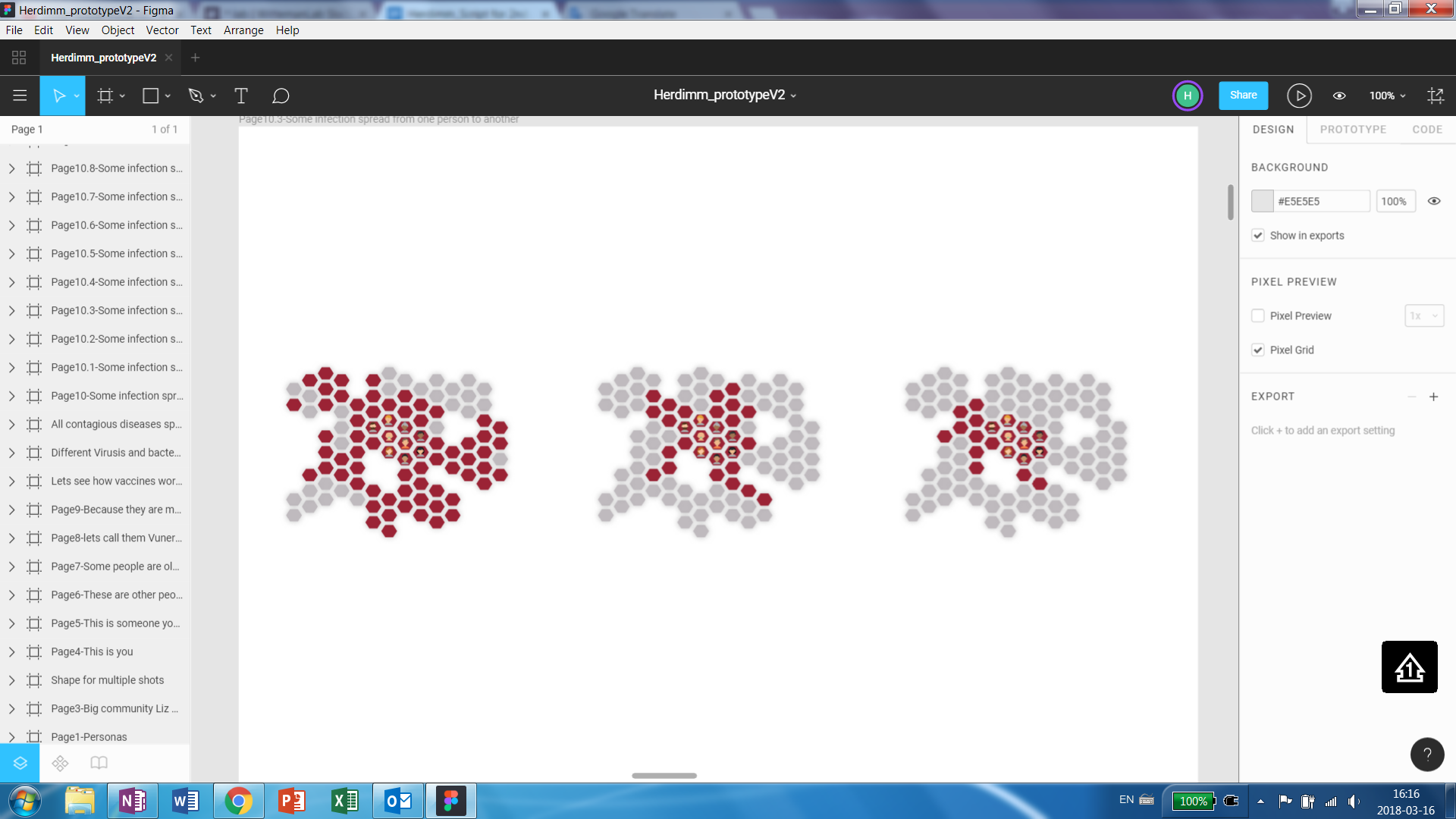 |  |  |
| 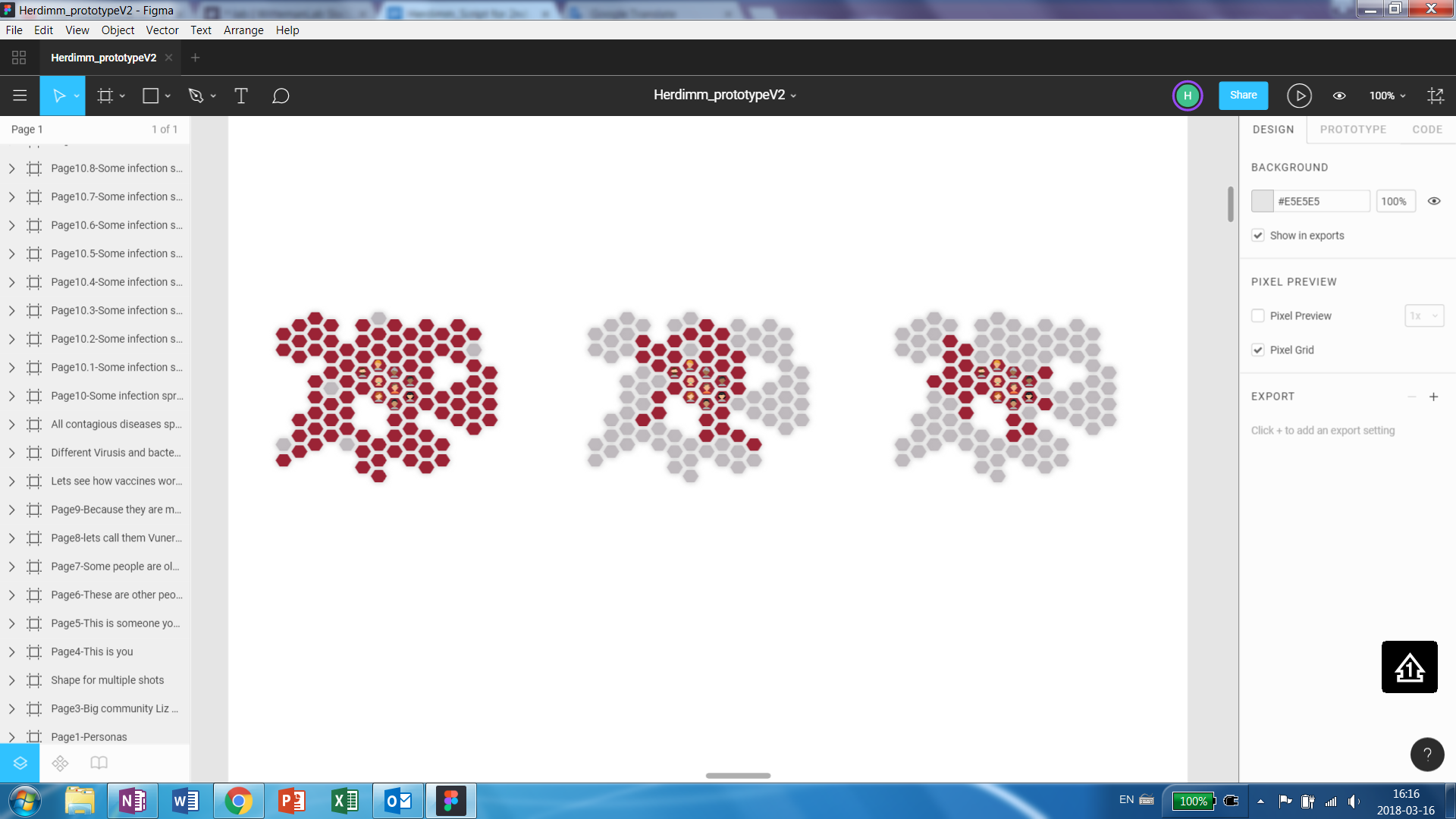 |  |  |
| 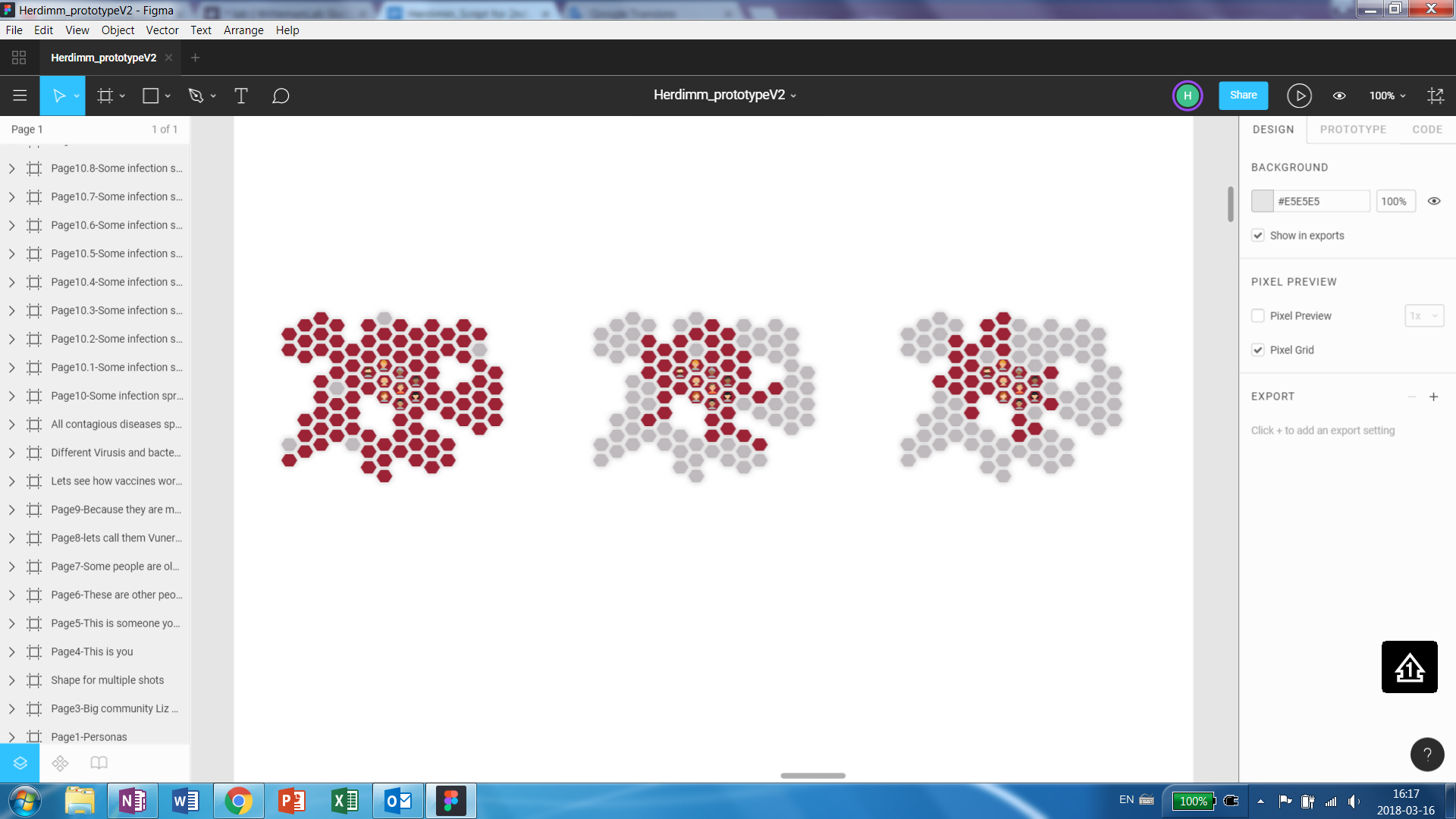 |  |  |
| 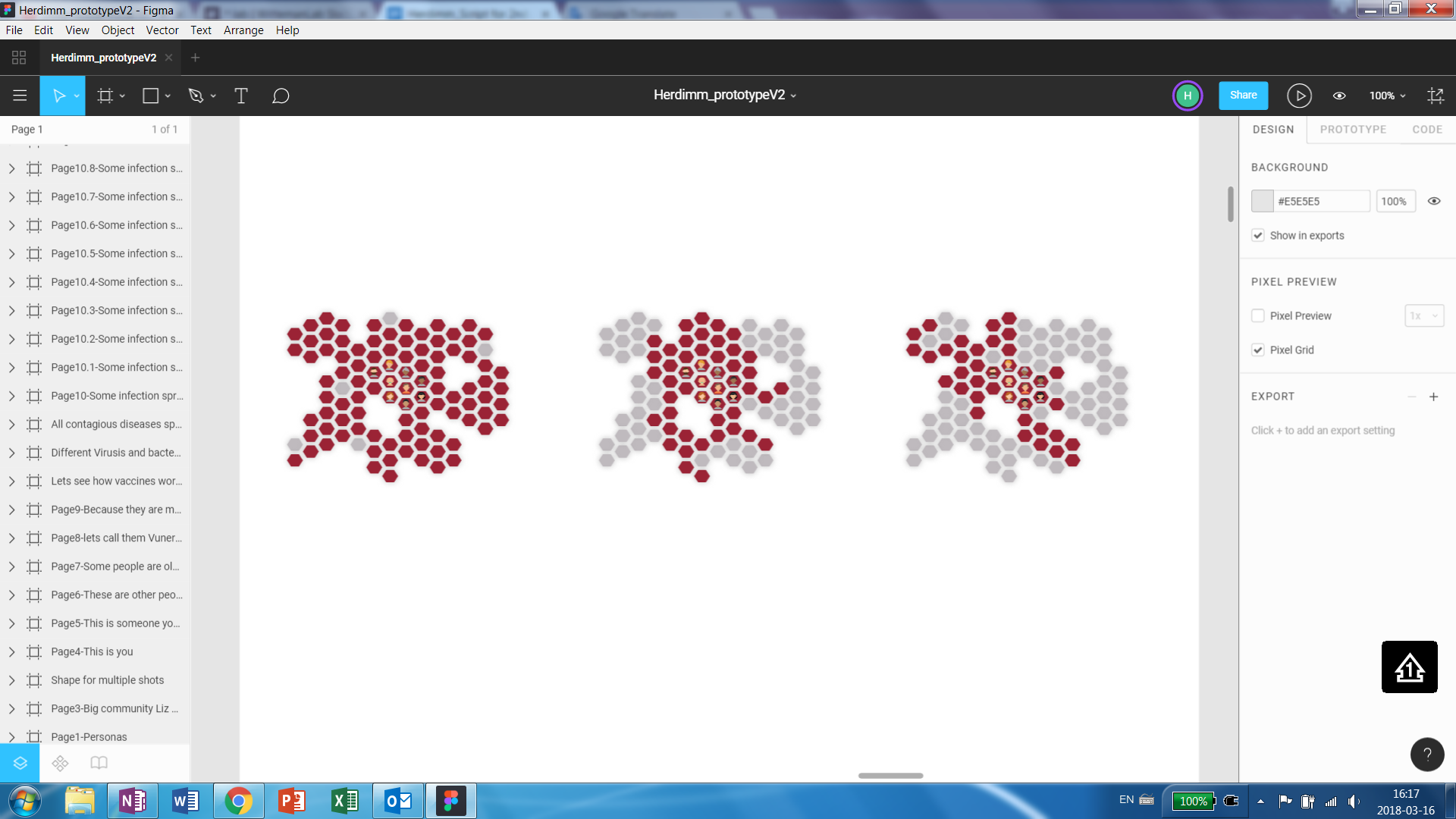 |  |  |
| 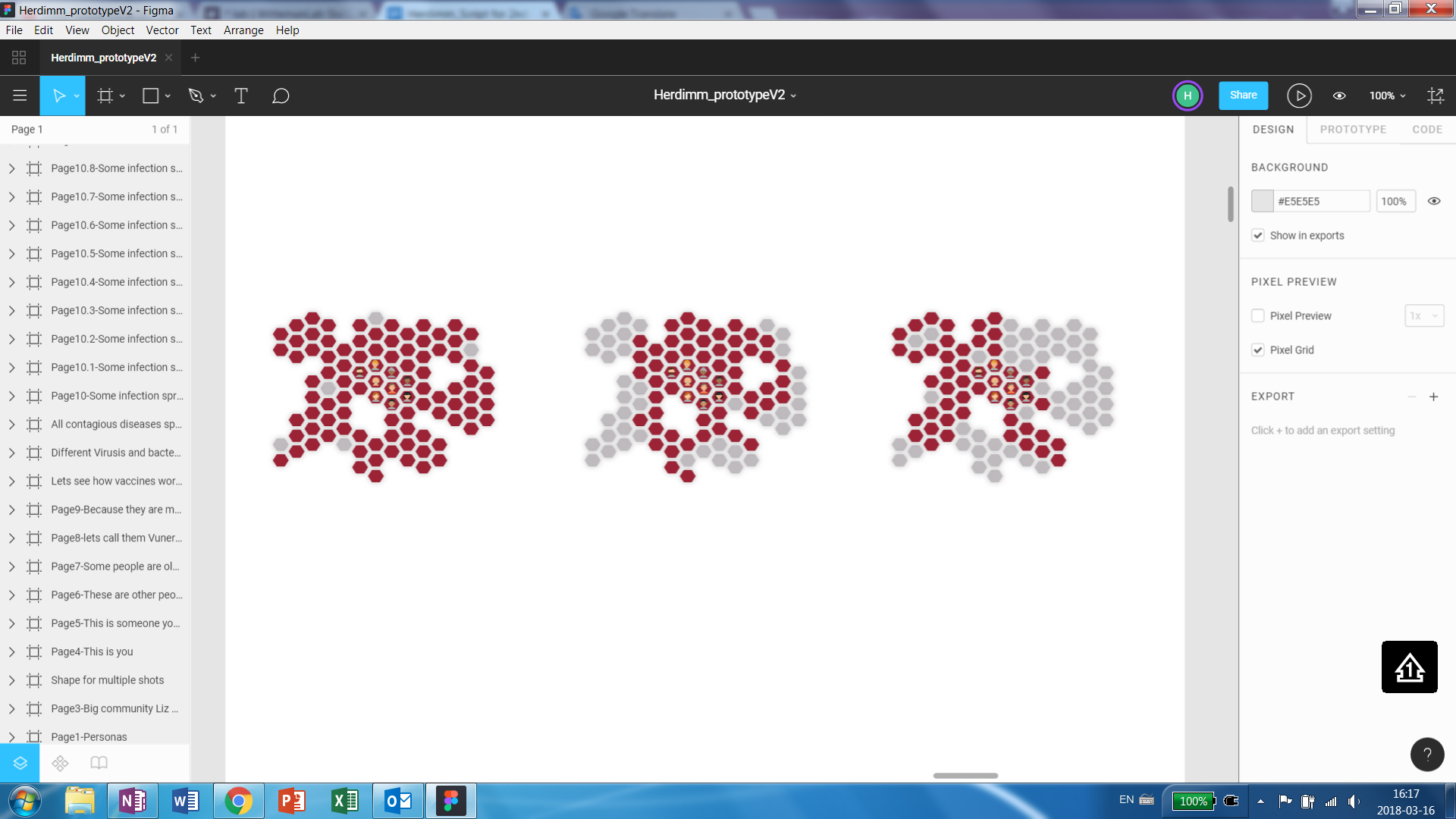 |  |  |
| 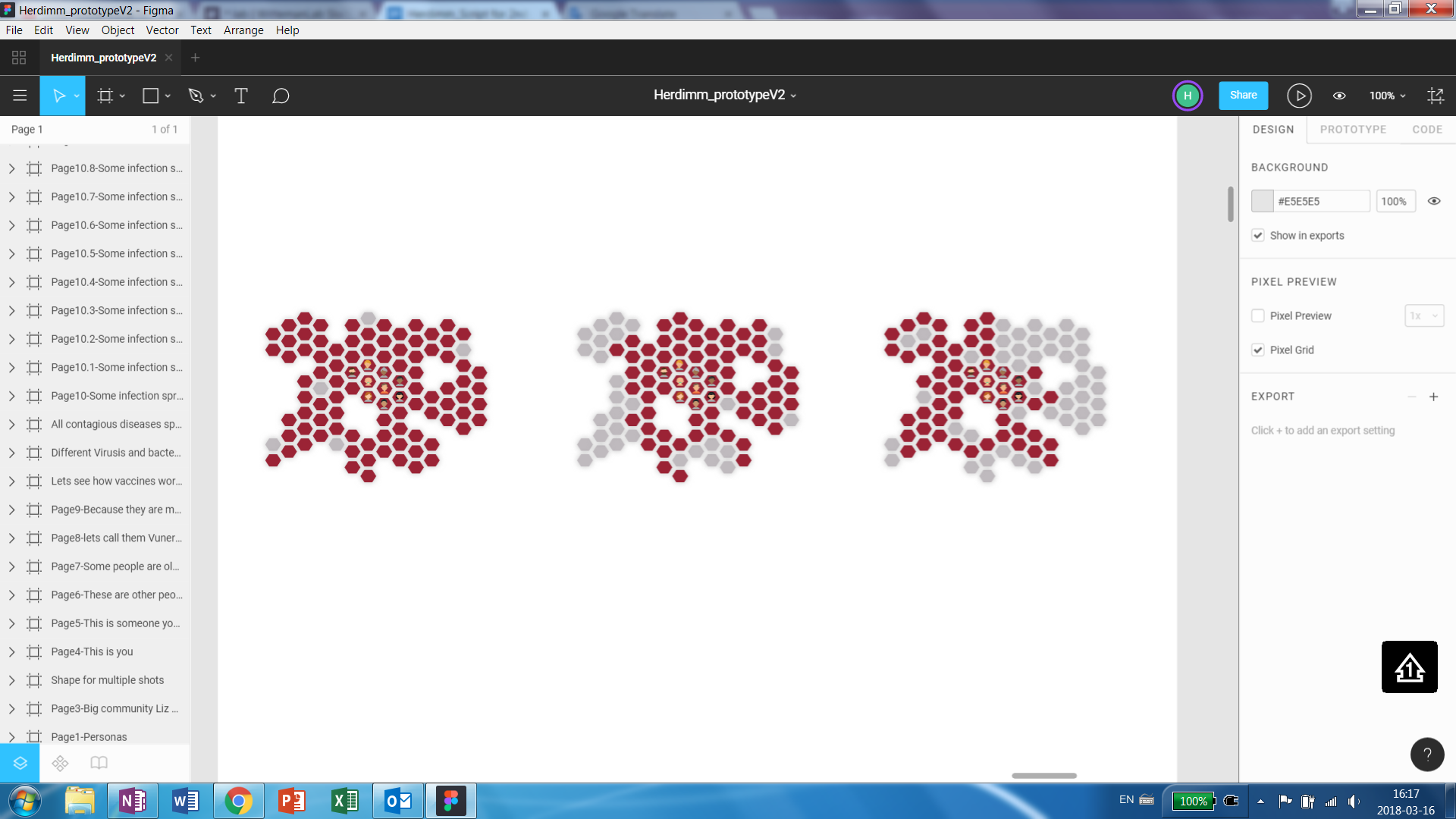 |  |  |
| 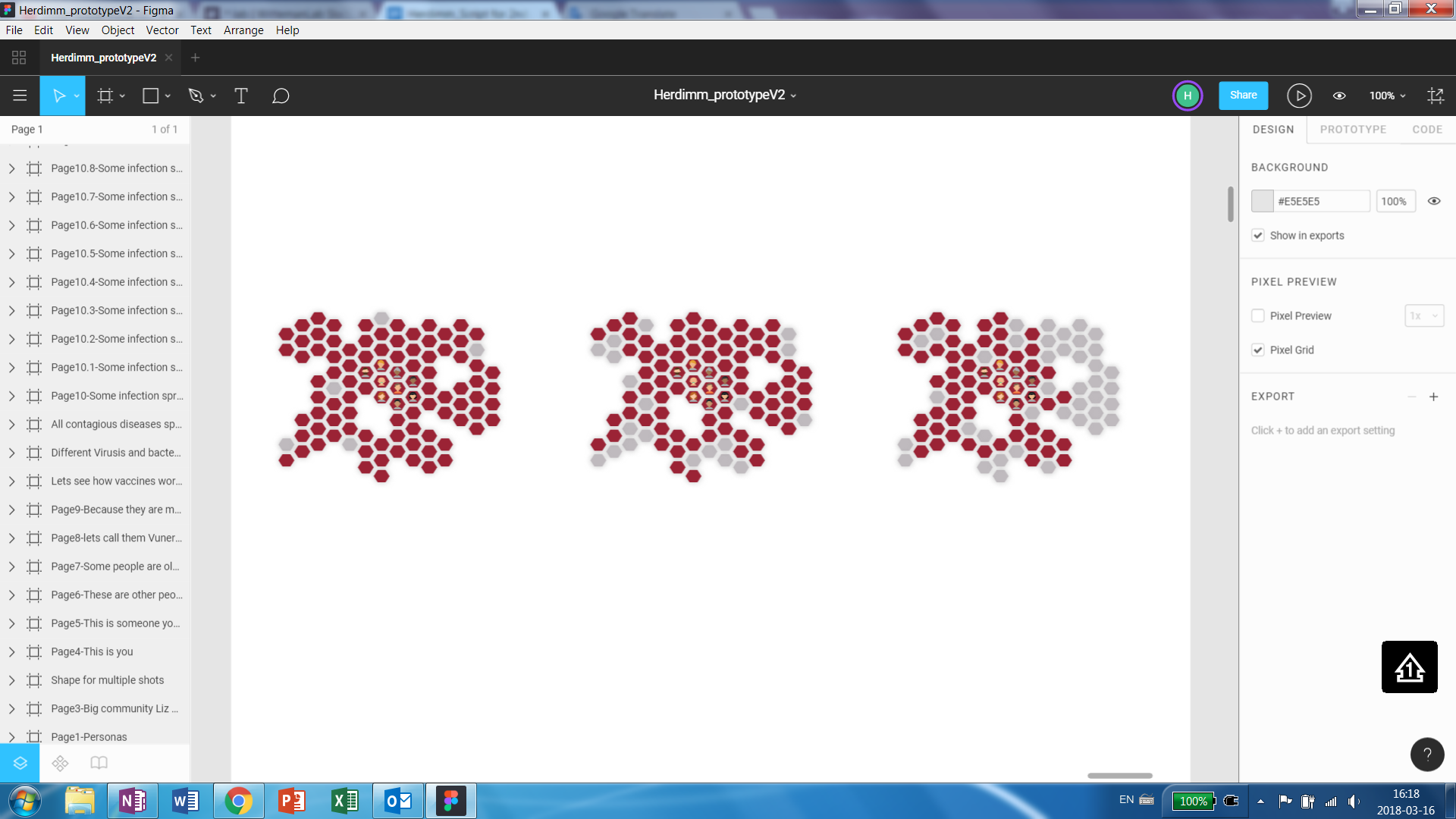 |  |  |
| 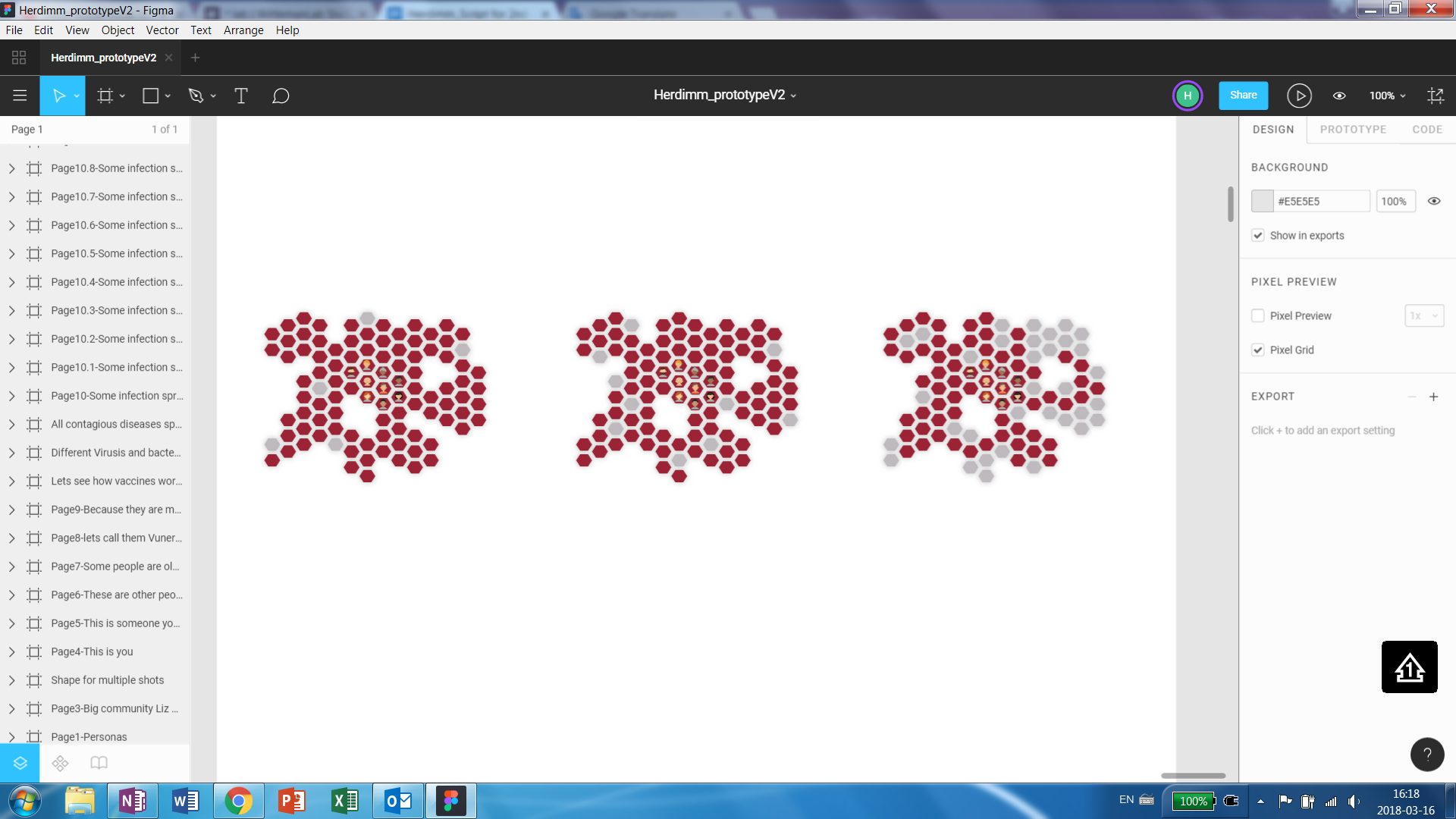 |  |  |
| Vaccines | | |
| 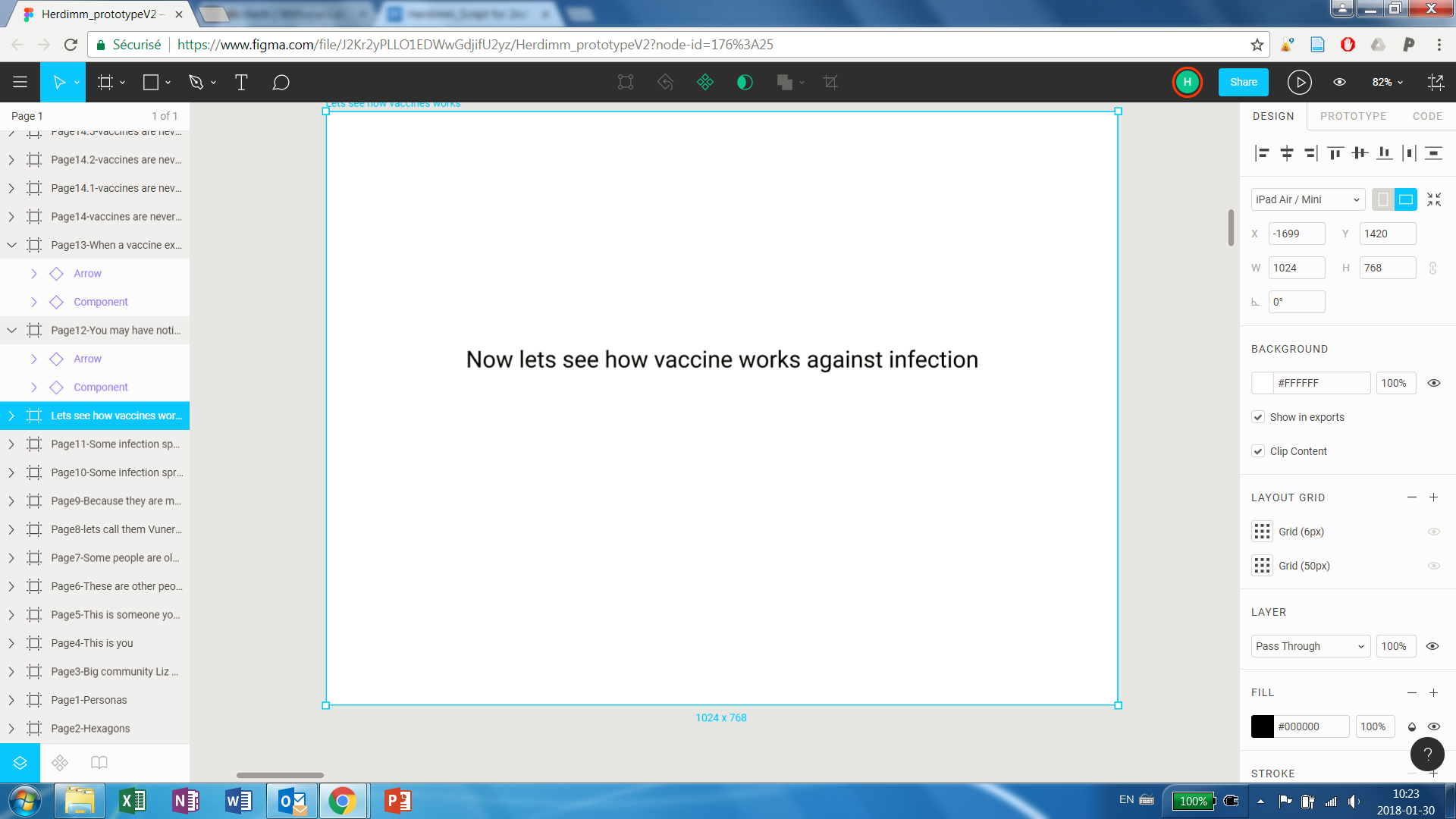 | Now let's see how vaccines work to protect people from diseases. | Voyons maintenant comment les vaccins protègent les gens contre les infections. |
| 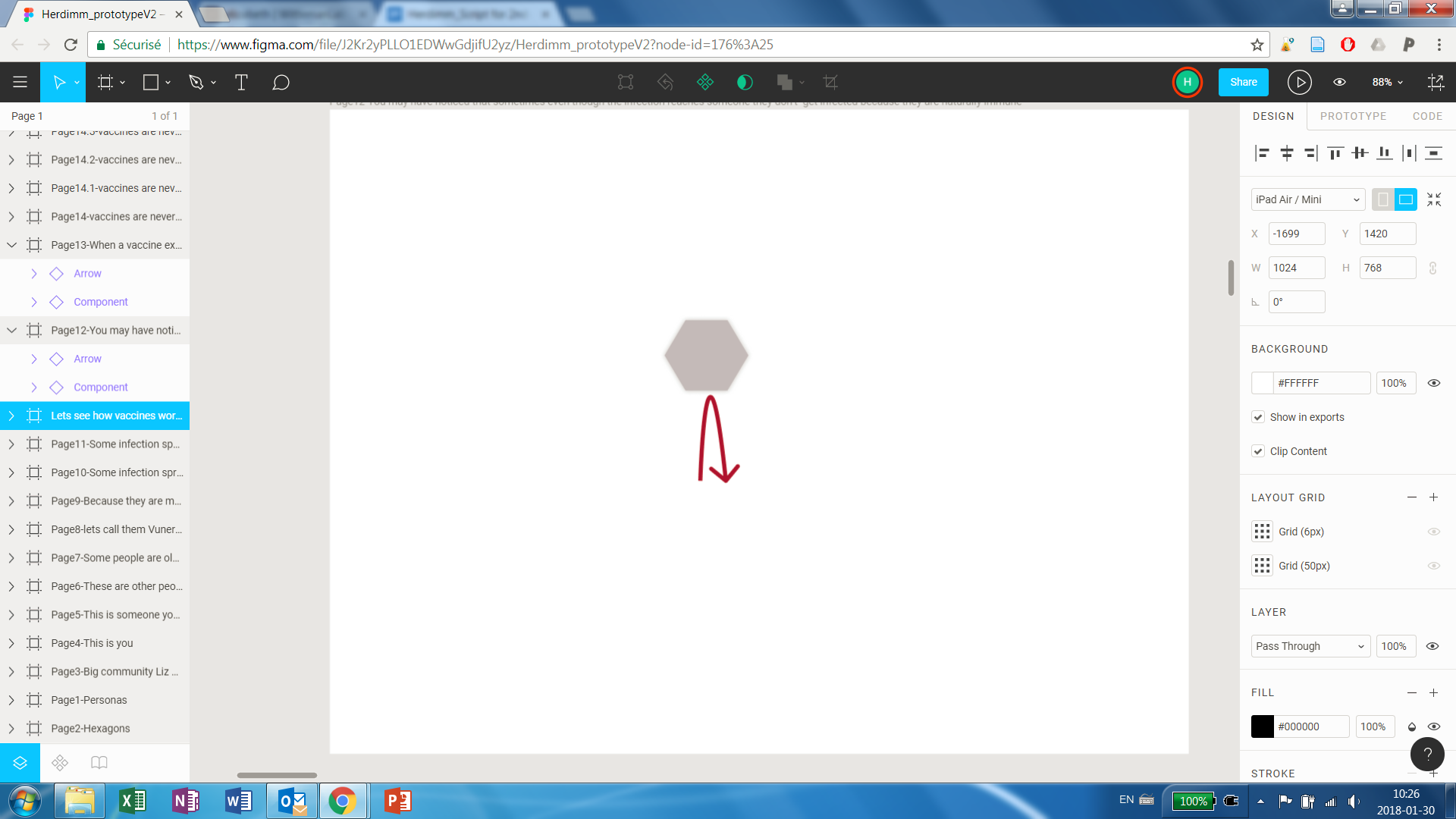 | You may have noticed that sometimes even though a disease reaches someone they don't catch it. This is because they are immune to that disease. | Vous avez peut-être remarqué que parfois, même si une maladie atteint une personne , elle ne l'attrape pas. C'est parce qu'elle est immunisée contre cette maladie. |
| 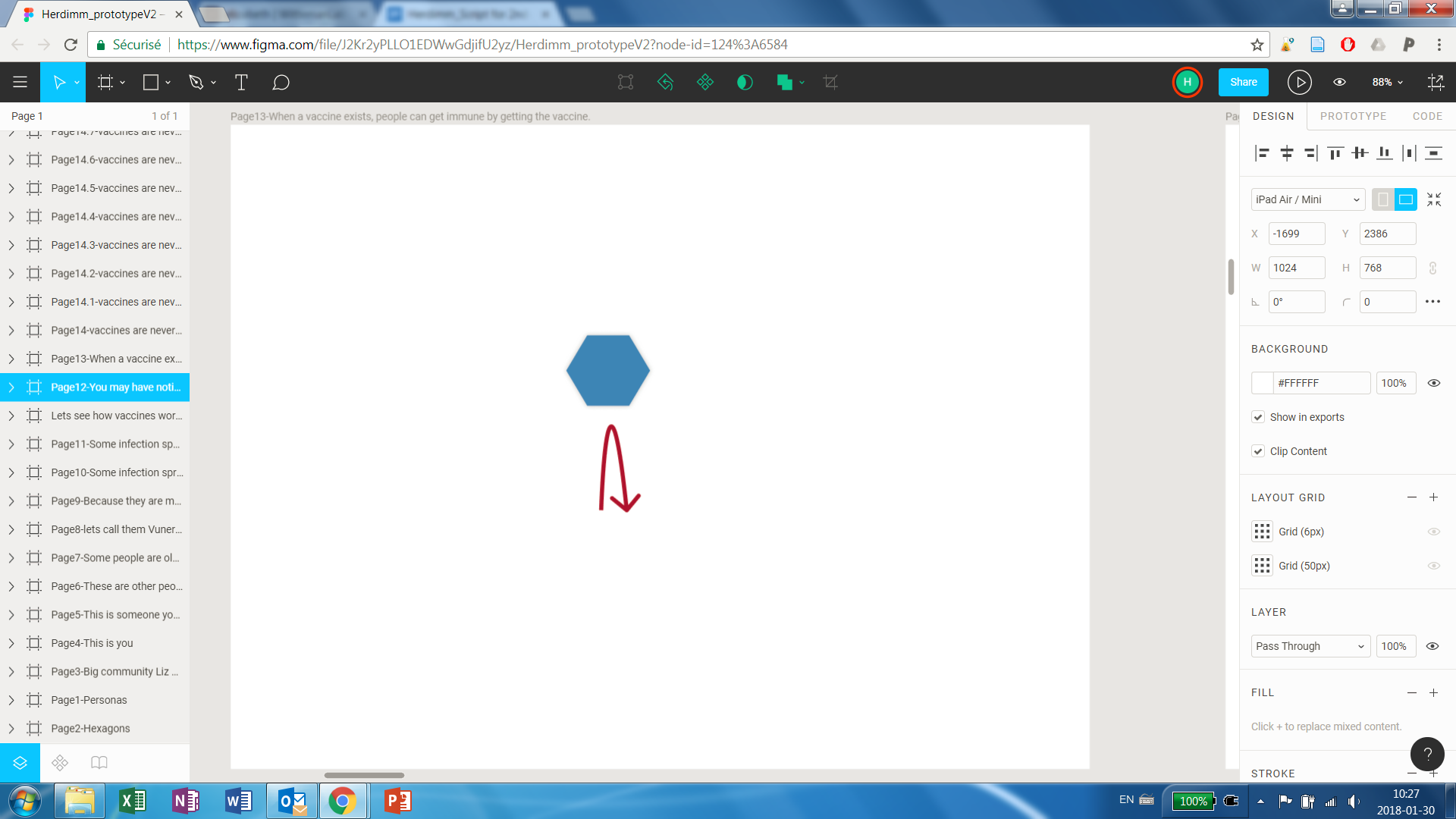 | When a vaccine exists against a disease, people can become immune to that disease by getting the vaccine. This helps their body resist catching the disease and getting sick. | Lorsqu’un vaccin existe contre une infection, les gens sont immunisés en recevant le vaccin. *Cela aide leur corps à résister à la maladie et à éviter de tomber malade.* |
| 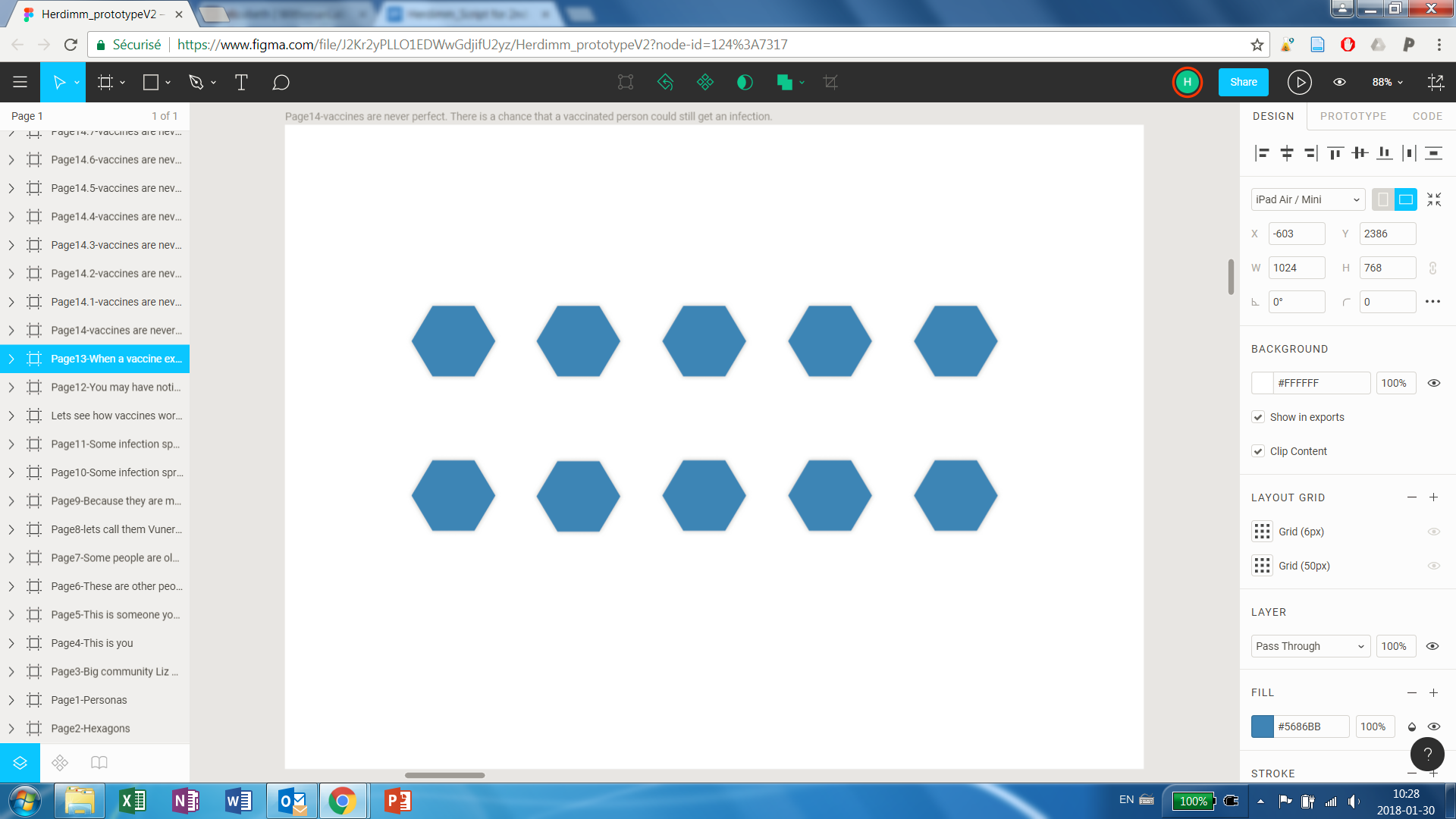 | But vaccines are never perfect. | Mais les vaccins ne sont pas parfaits. |
| 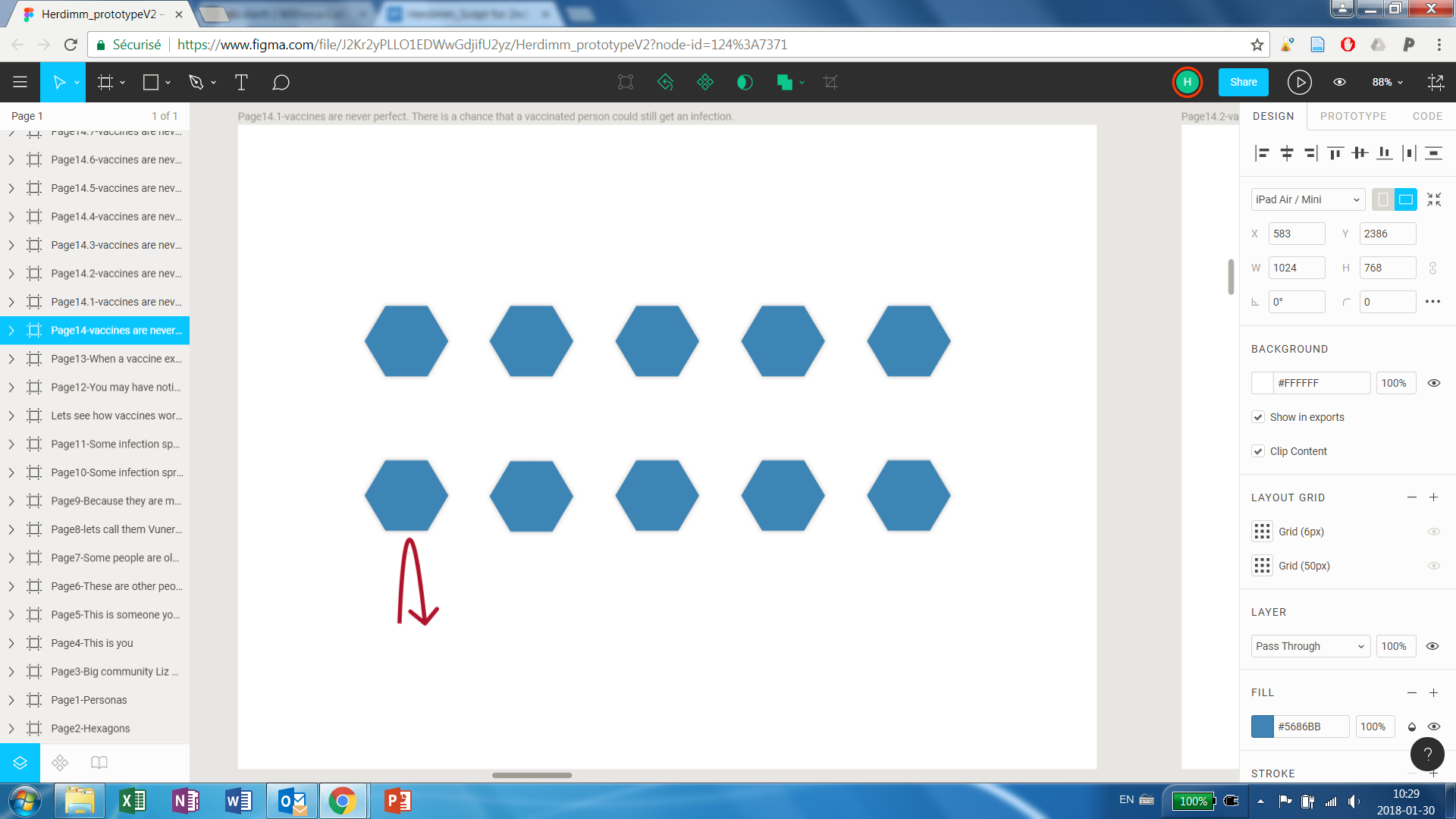 | There is a chance that a vaccinated person could still catch the disease.  (Show one by one hit of hexagons, with infection bouncing off most of them: “bing!” At the end, 1 hexagon is red.) | Il existe toujours la possibilité qu'une personne vaccinée attrape une infection.  (Montrer un par un coup d'hexagones, avec l'infection rebondissant la plupart d'entre eux: "bing!" À la fin, 1 hexagone est rouge.) |
| 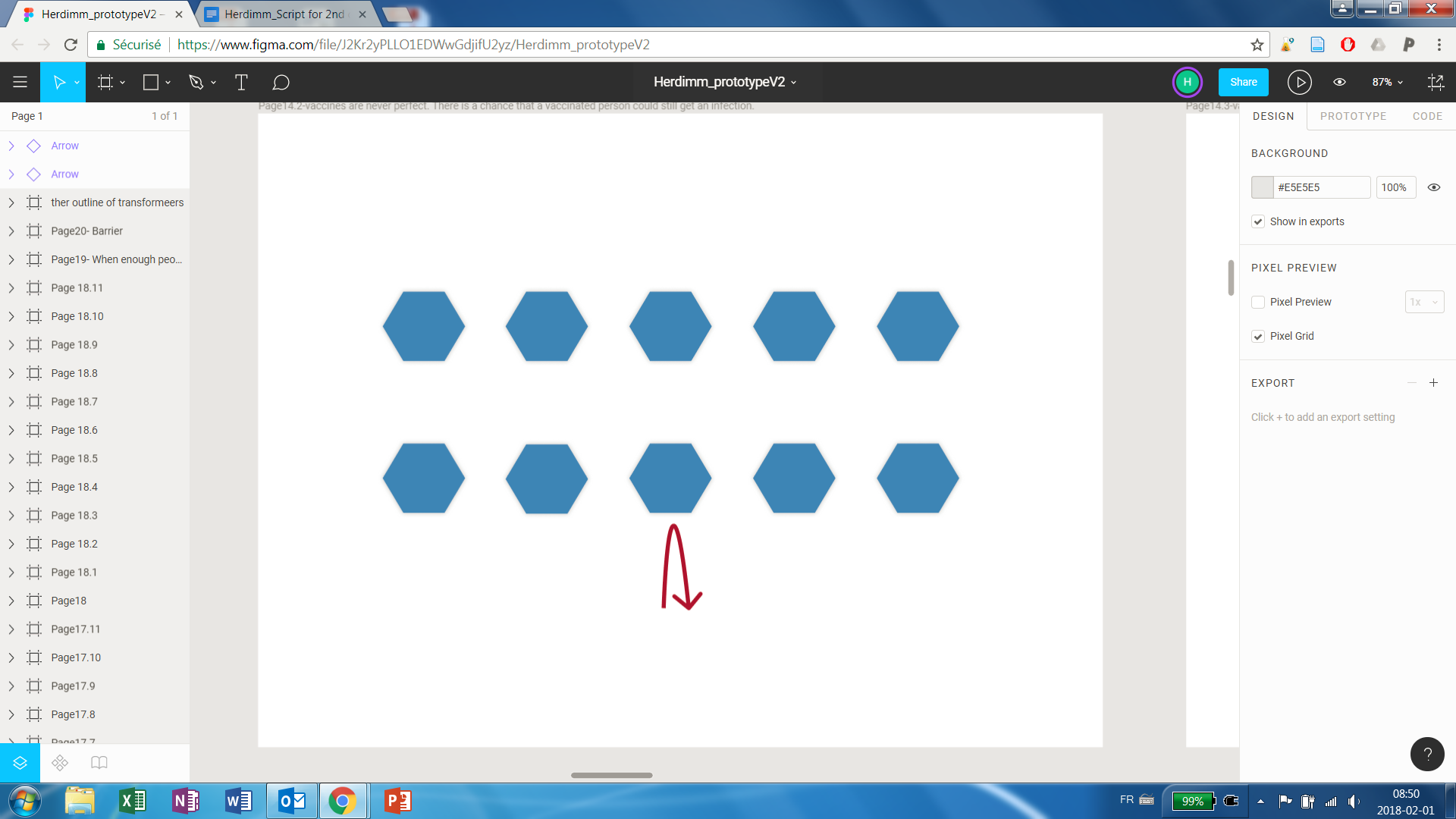 | (bing!) | (bing!) |
| 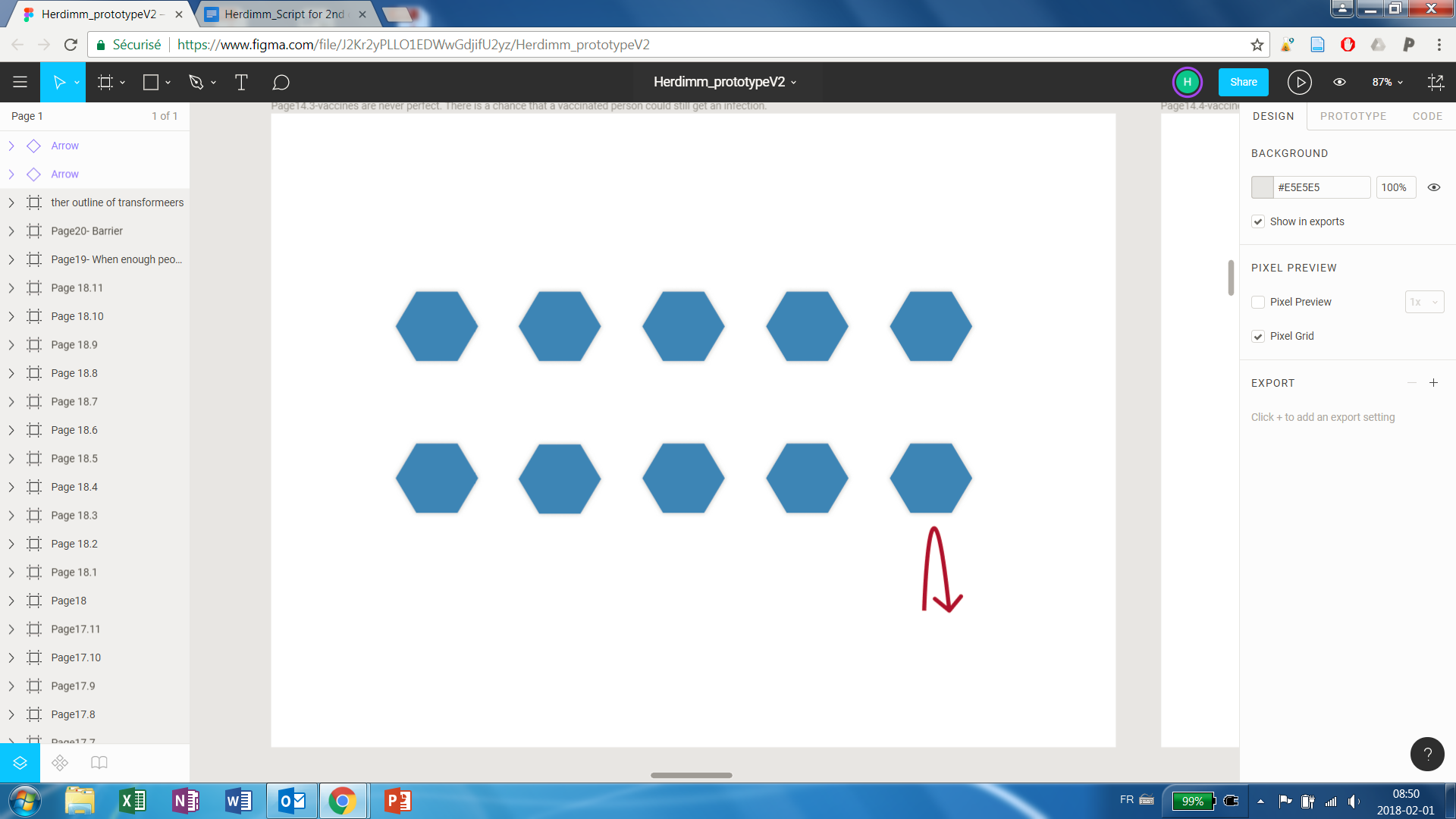 | (bing!) | (bing!) |
| 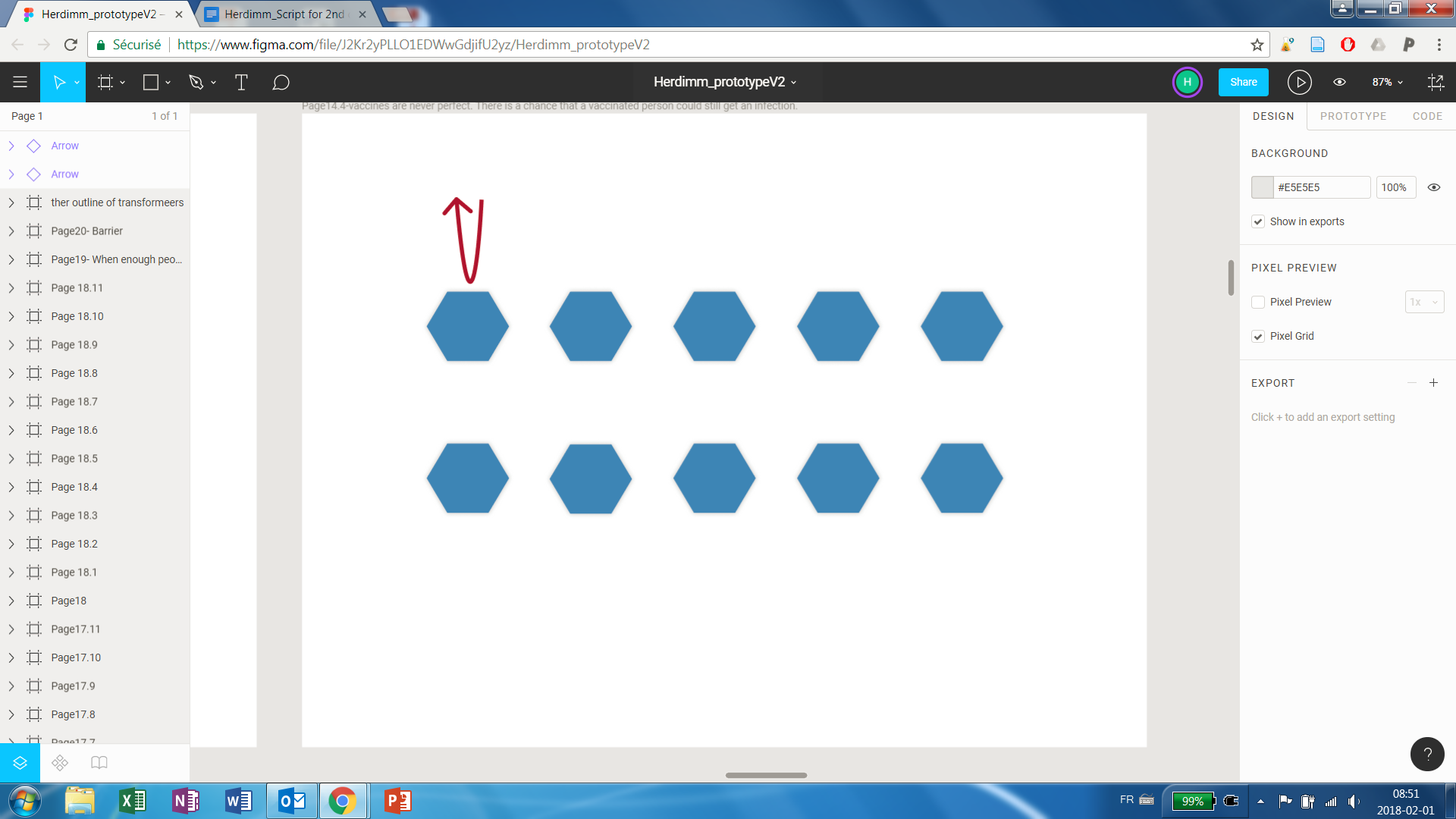 | (bing!) | (bing!) |
| 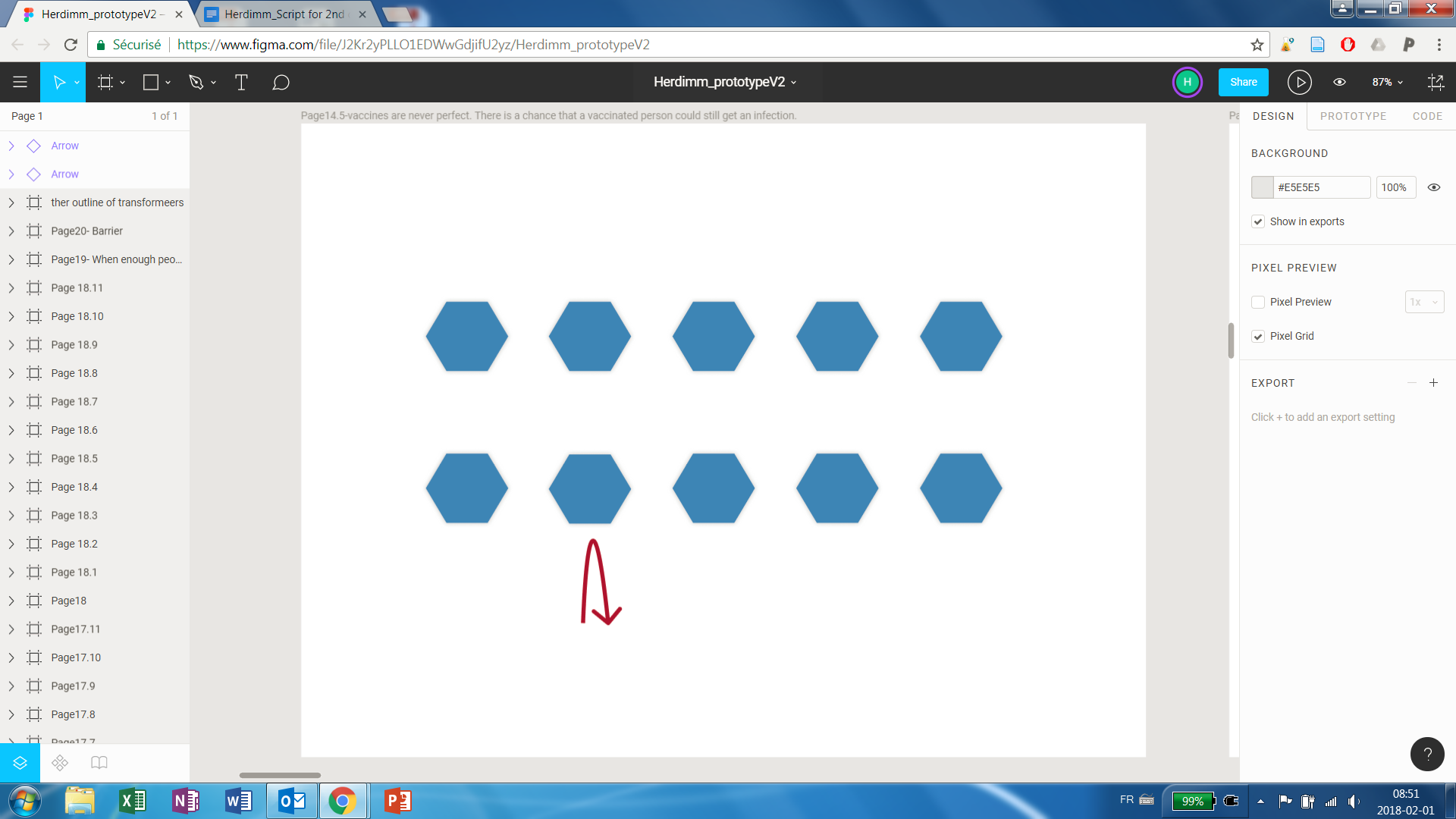 | (bing!) | (bing!) |
| 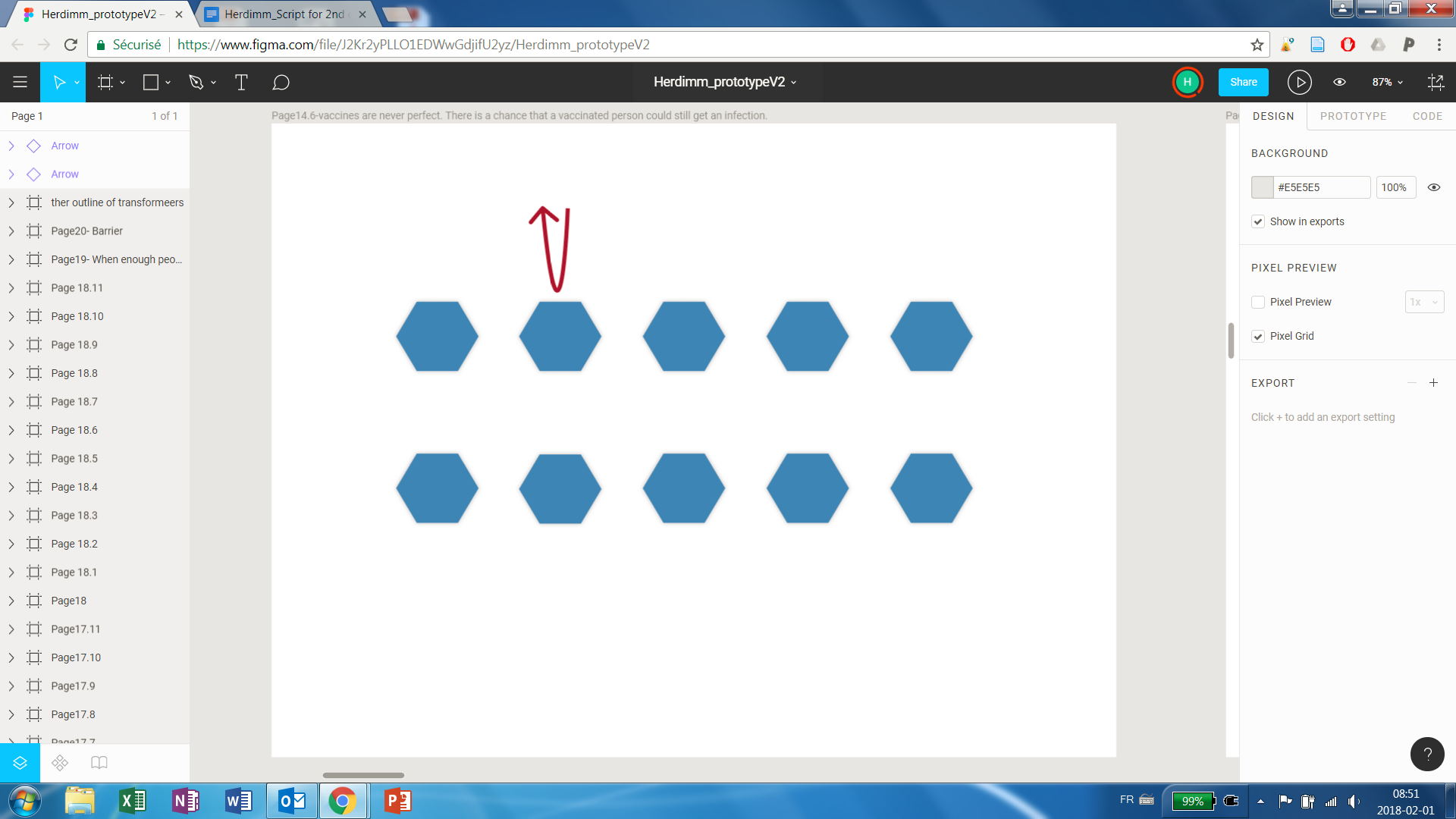 | (bing!) | (bing!) |
| 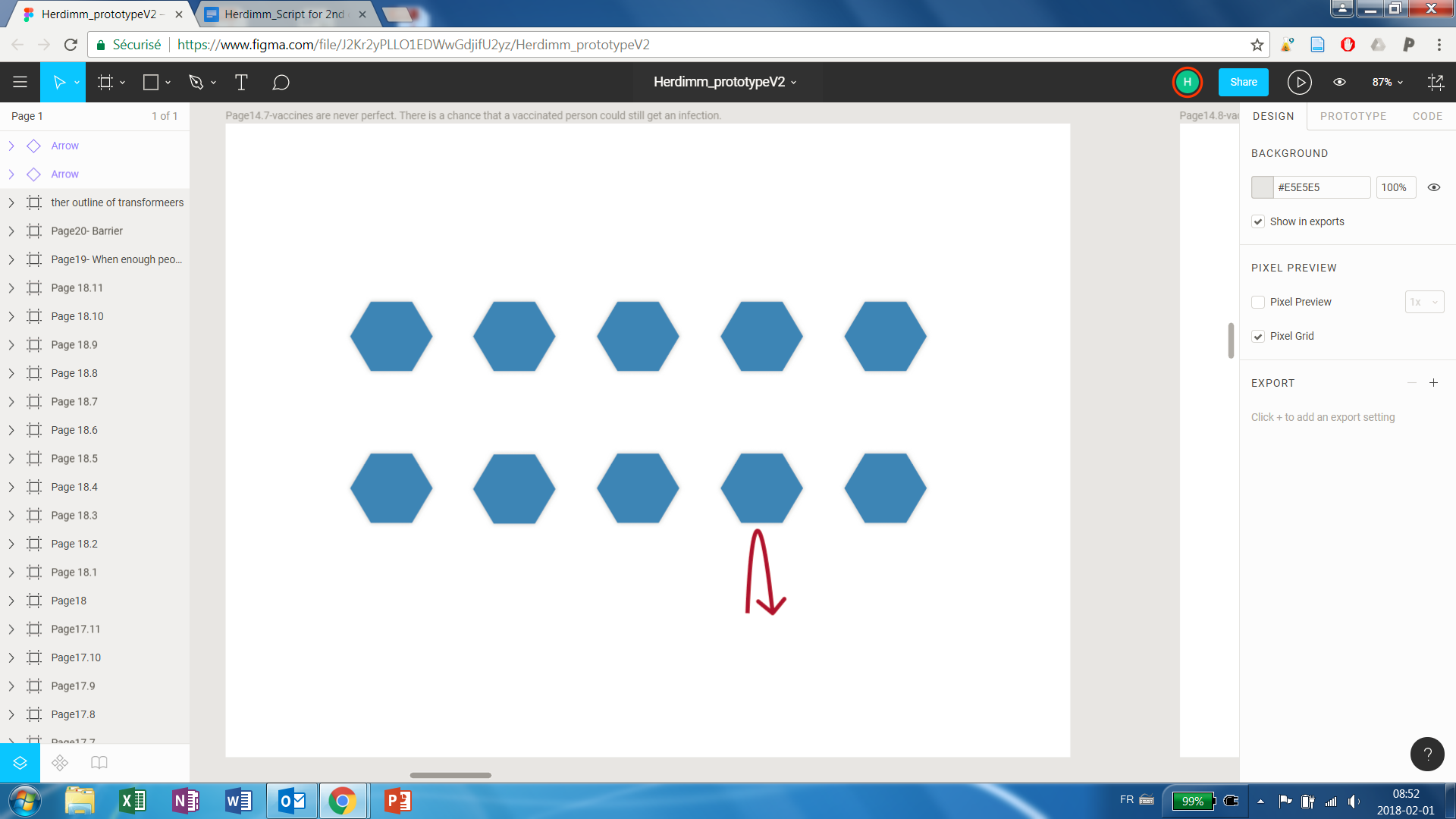 | (bing!) | (bing!) |
| 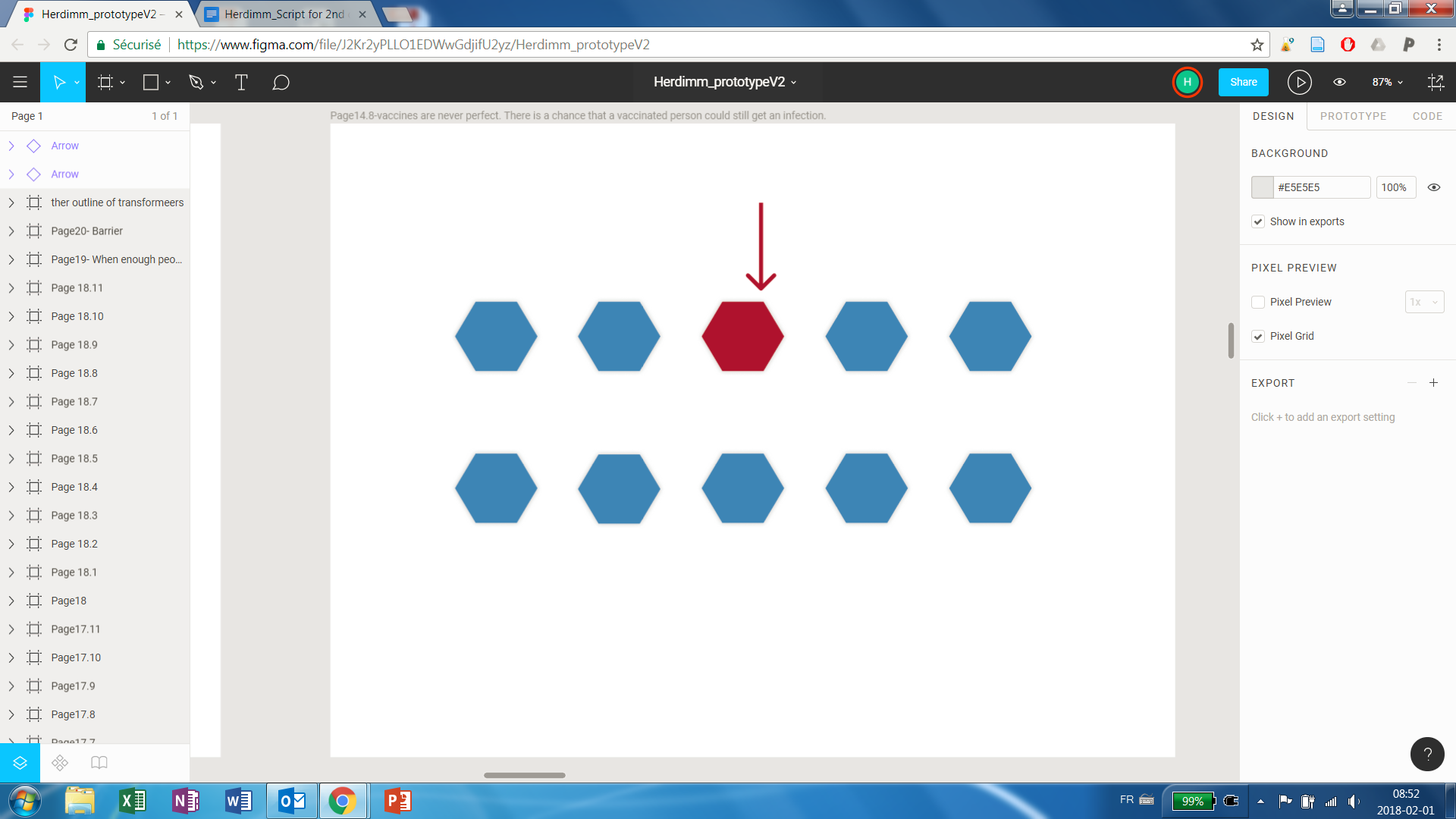 | (bing!) | (bing!) |
| 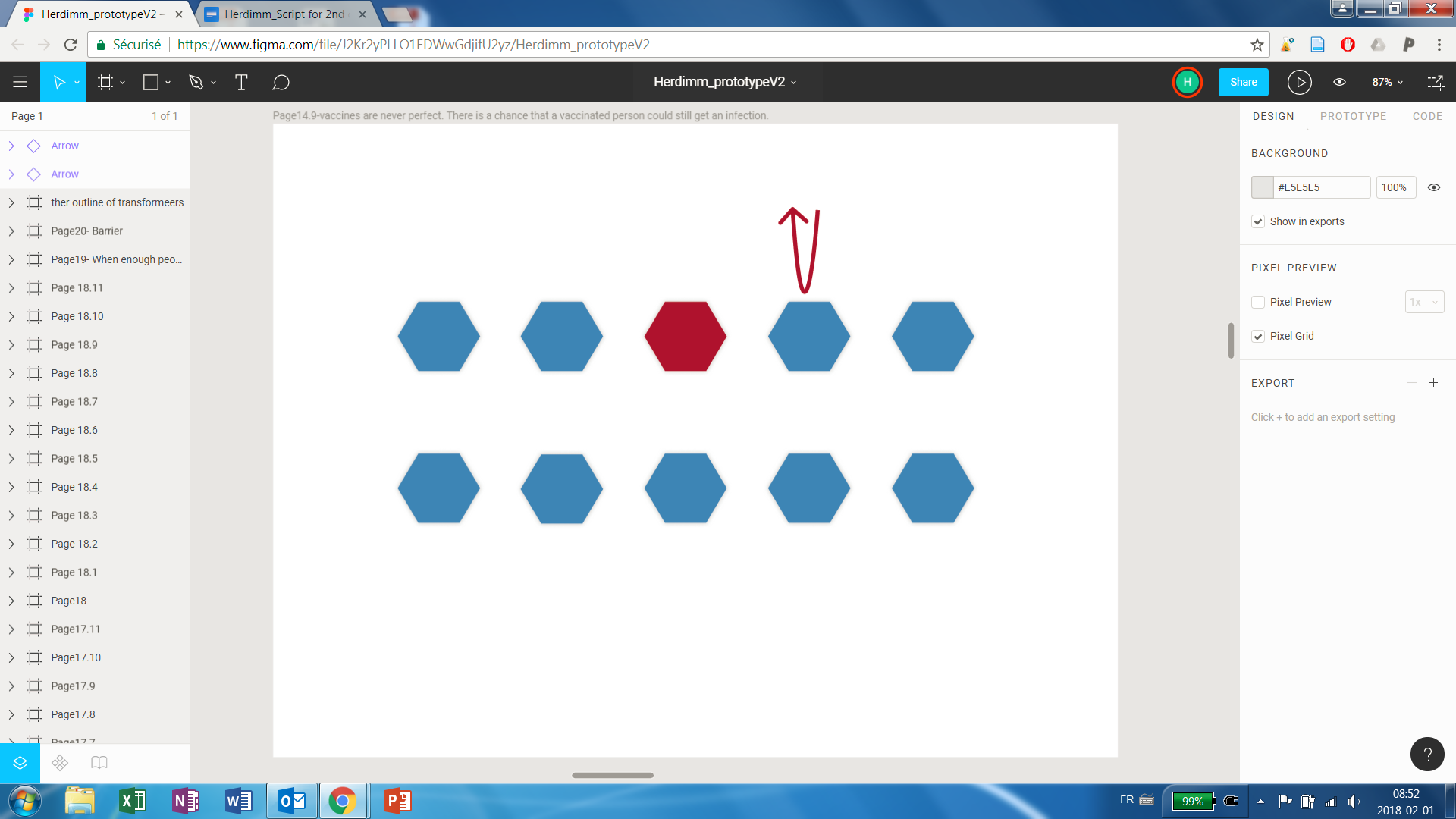 | (bing!) | (bing!) |
| 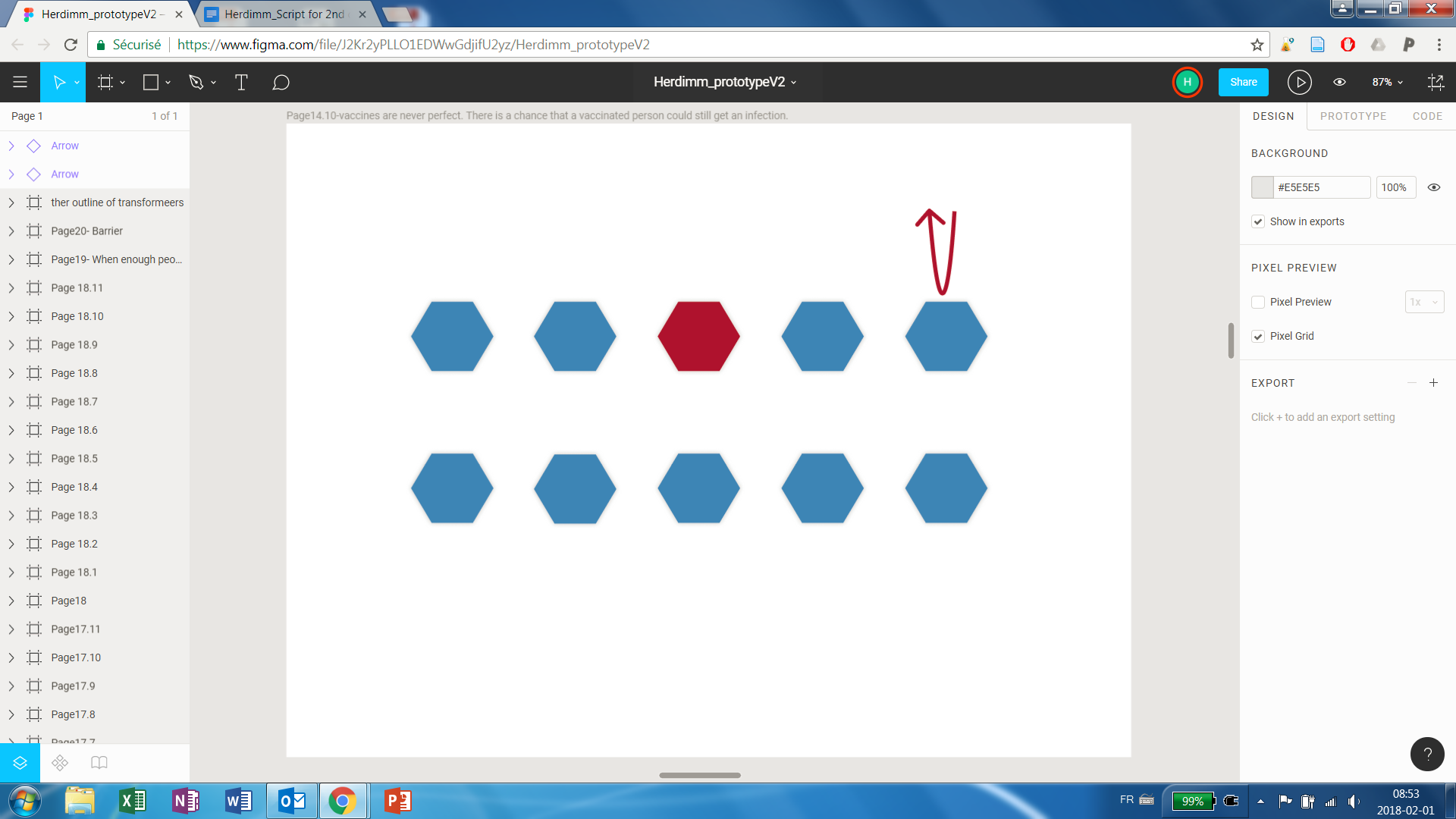 | (bing!) | (bing!) |
| 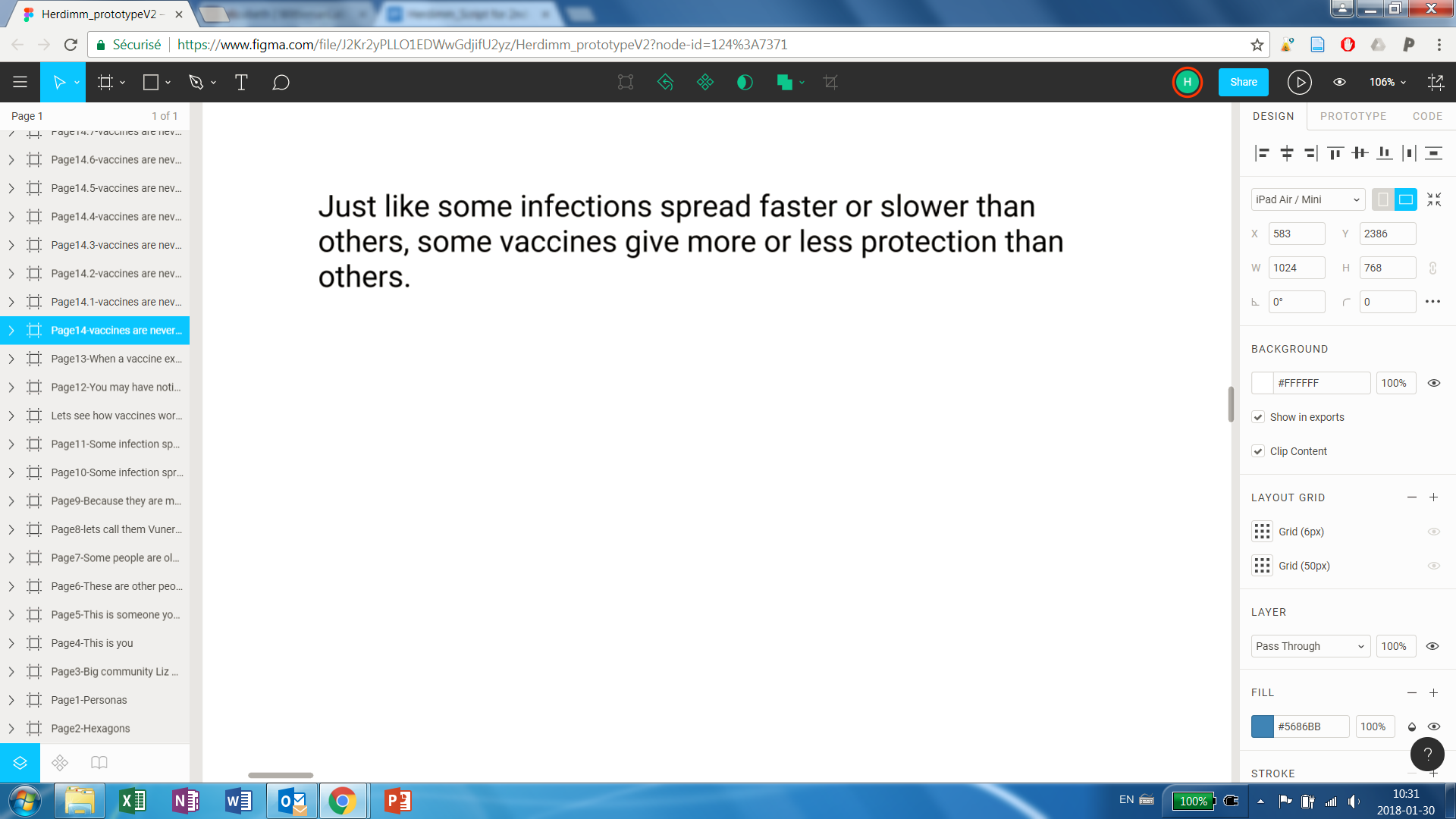 | Just like some infections spread faster or slower than others, some vaccines give more or less protection than others. | Tout comme certaines infections se propagent plus vite ou plus lentement que d'autres, certains vaccins offrent plus ou moins de protection que les autres. |
| 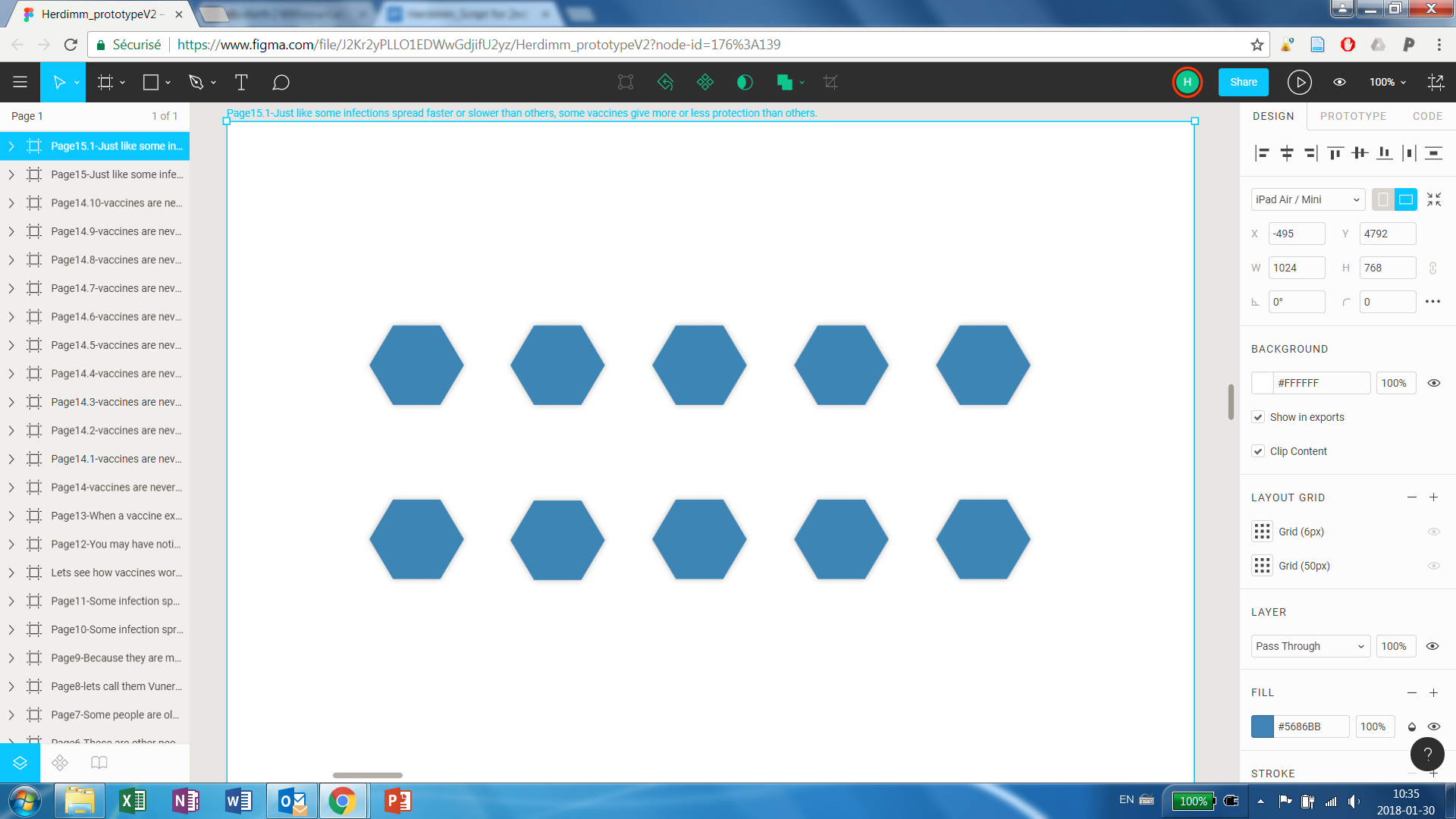 | (Infection hits one by one. At the end, 3 hexagons are red.) |  |
| 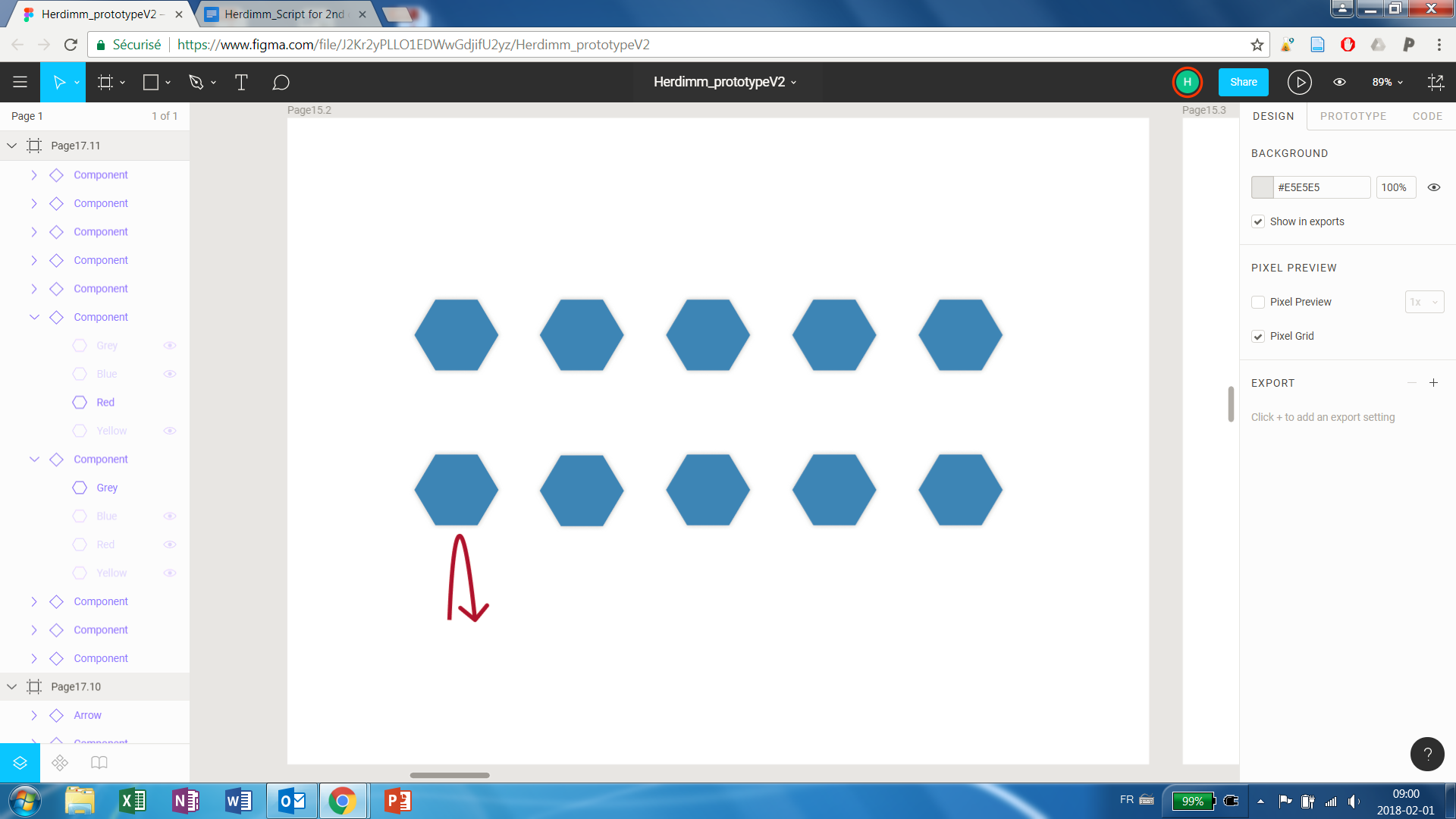 | (bing!) | (bing!) |
| 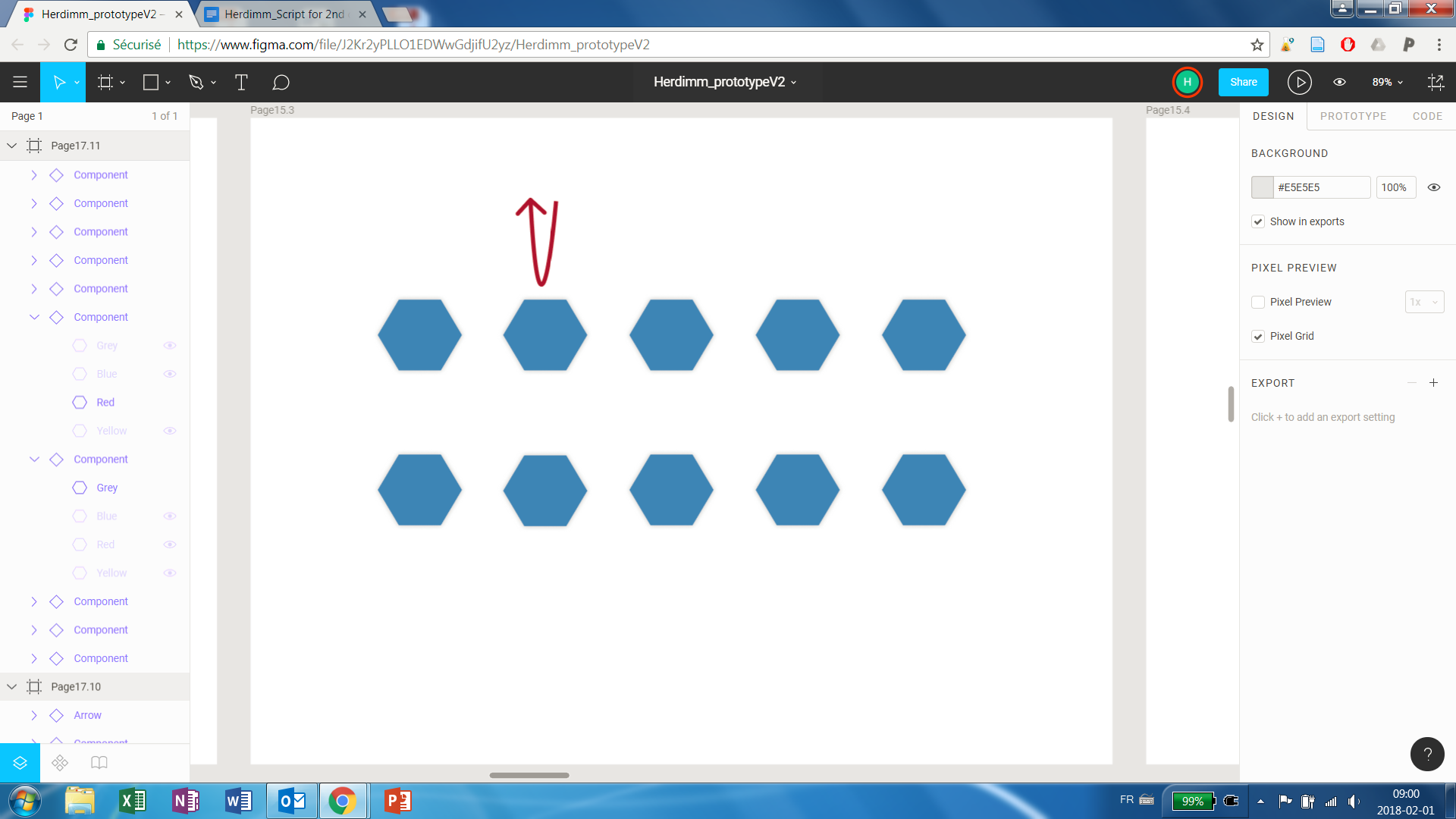 | (bing!) | (bing!) |
| 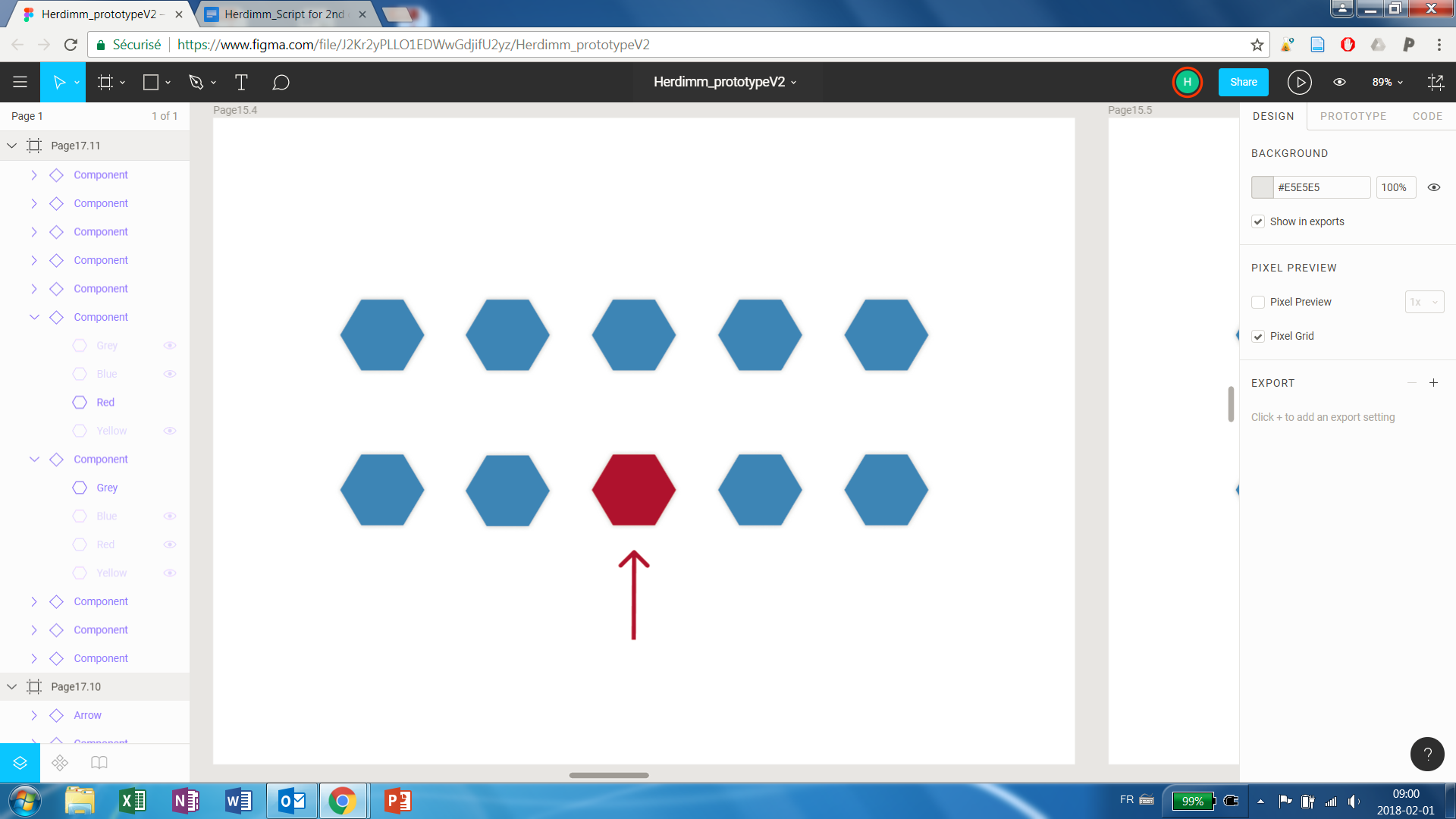 | (bing!) | (bing!) |
| 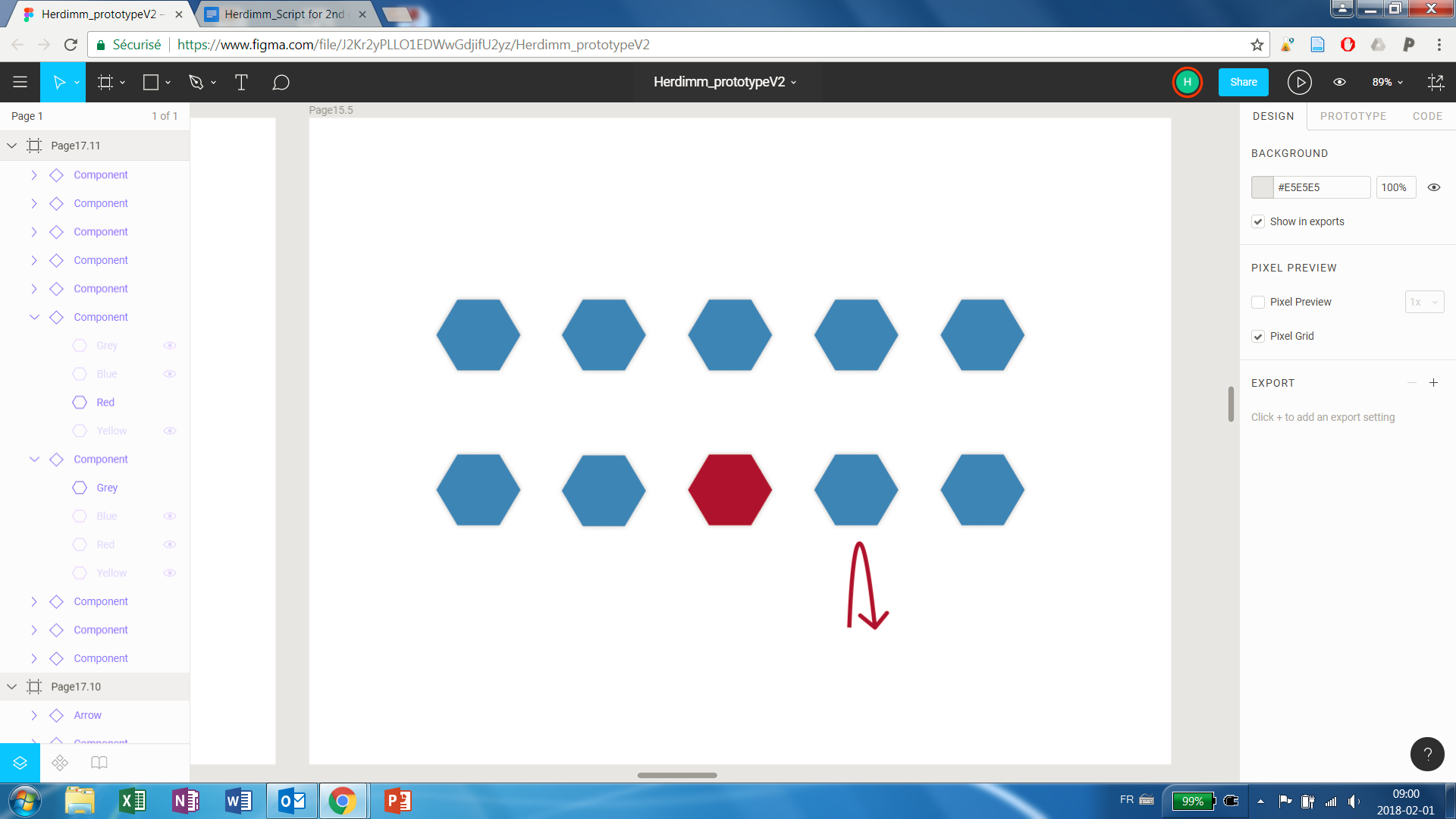 | (bing!) | (bing!) |
| 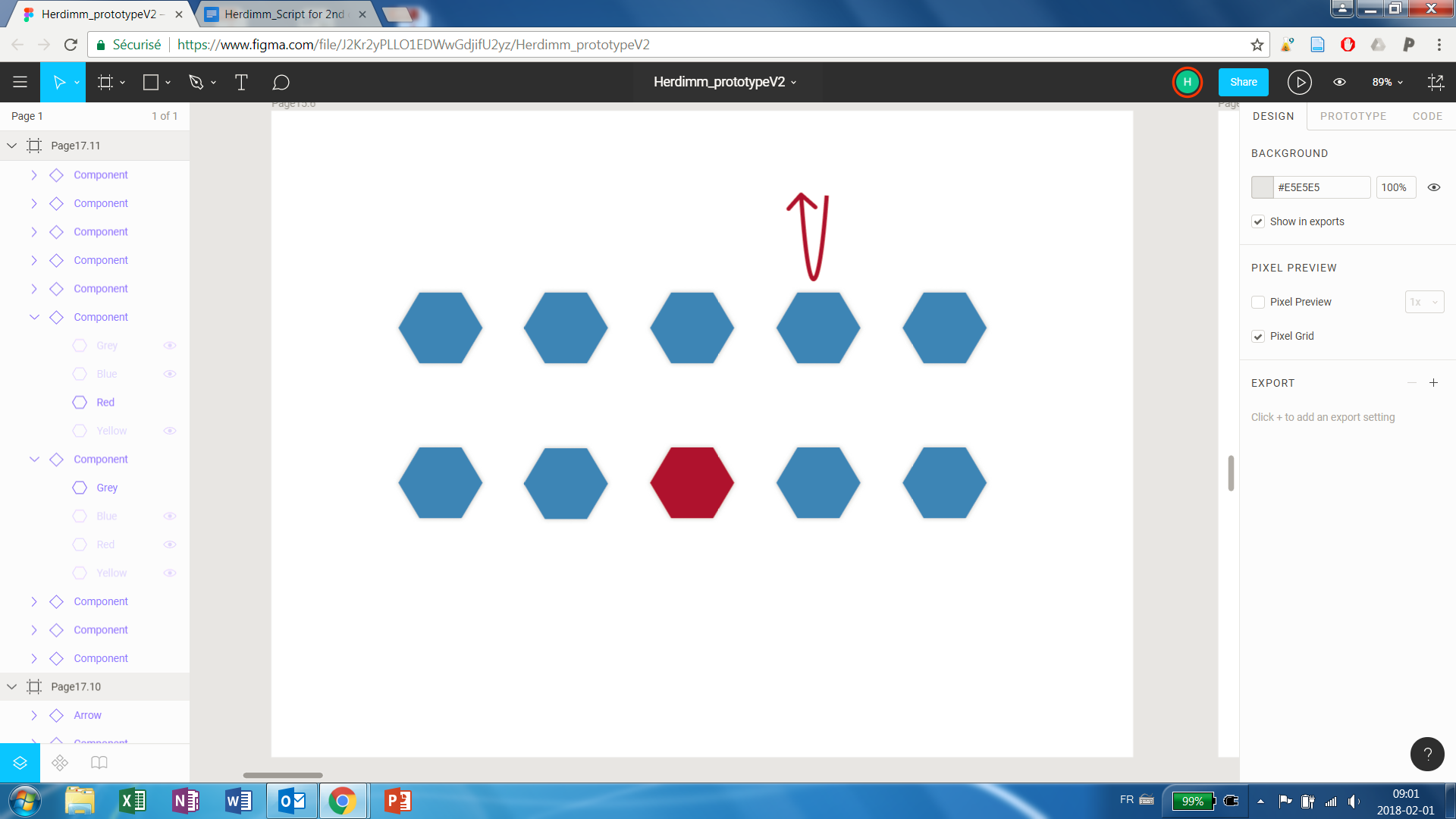 | (bing!) | (bing!) |
| 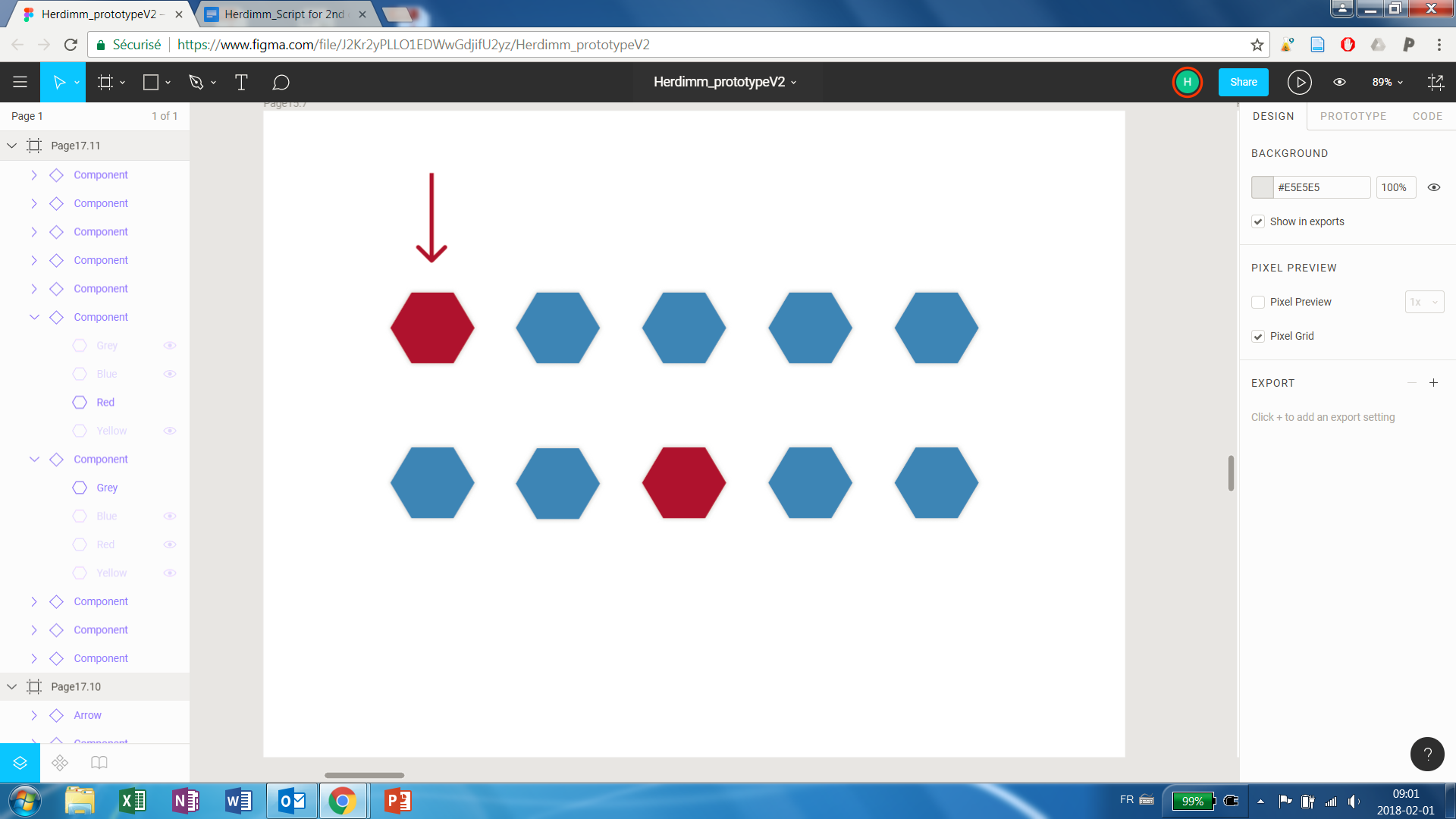 | (bing!) | (bing!) |
| 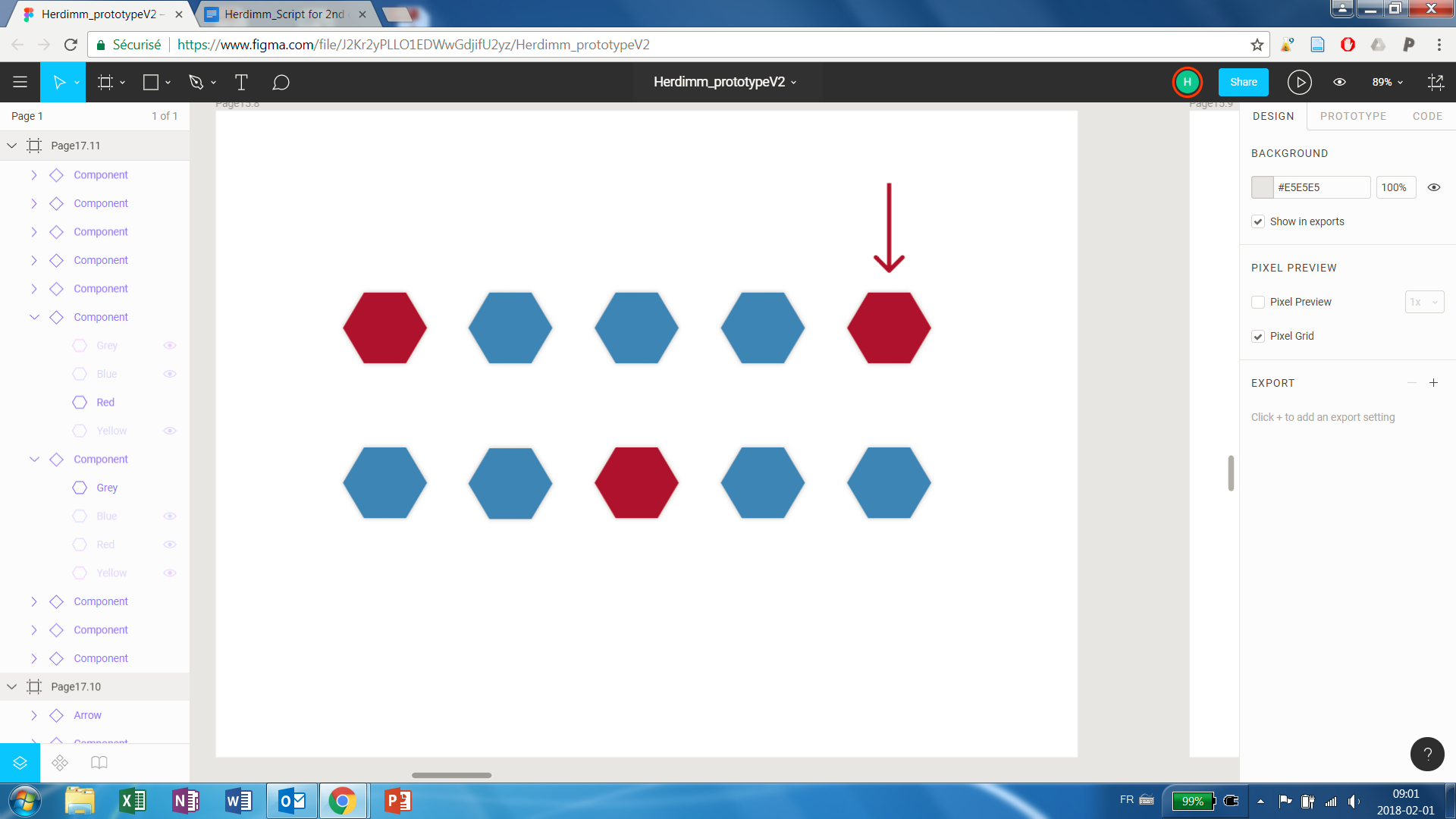 | (bing!) | (bing!) |
| 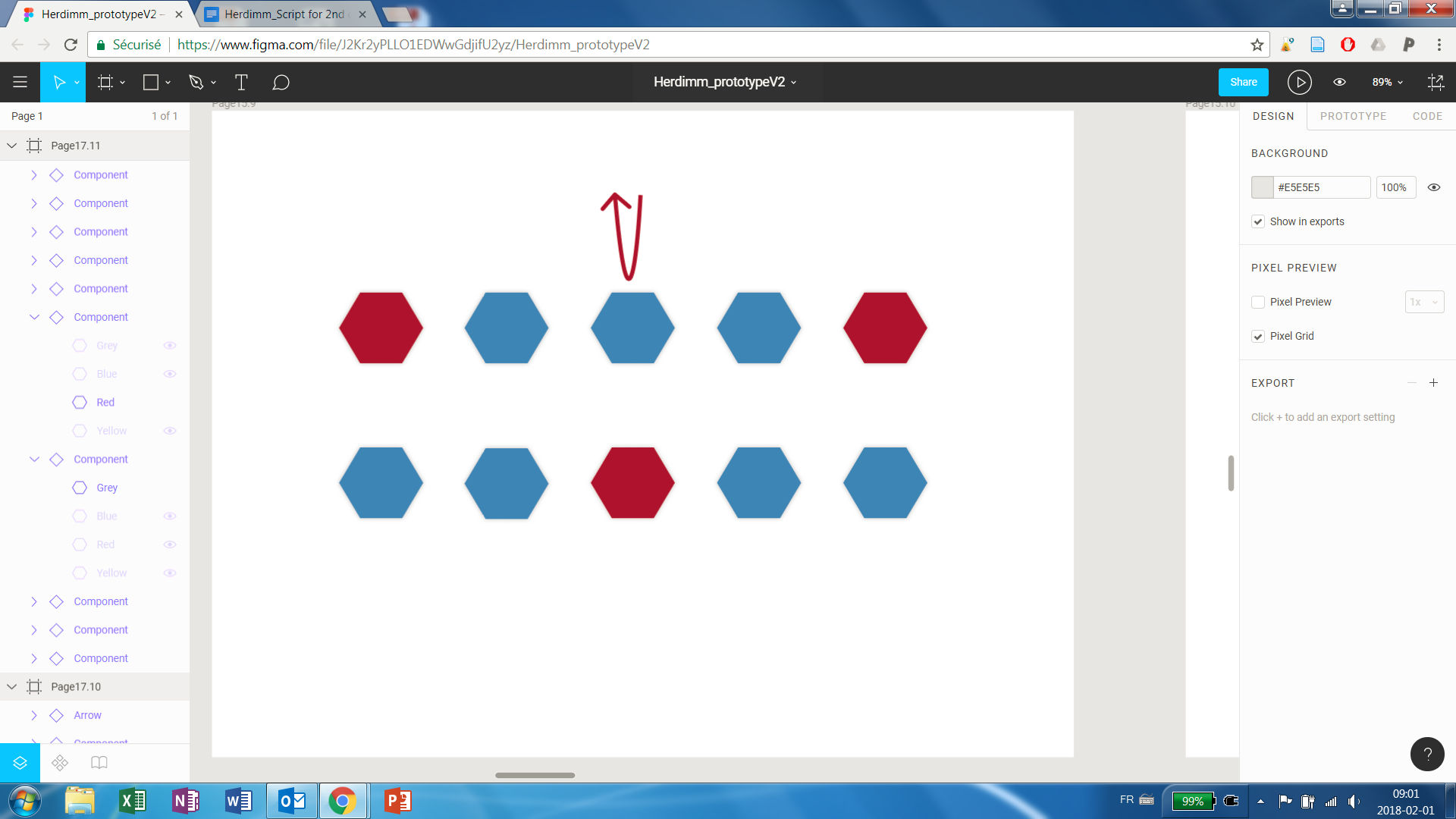 | (bing!) | (bing!) |
| 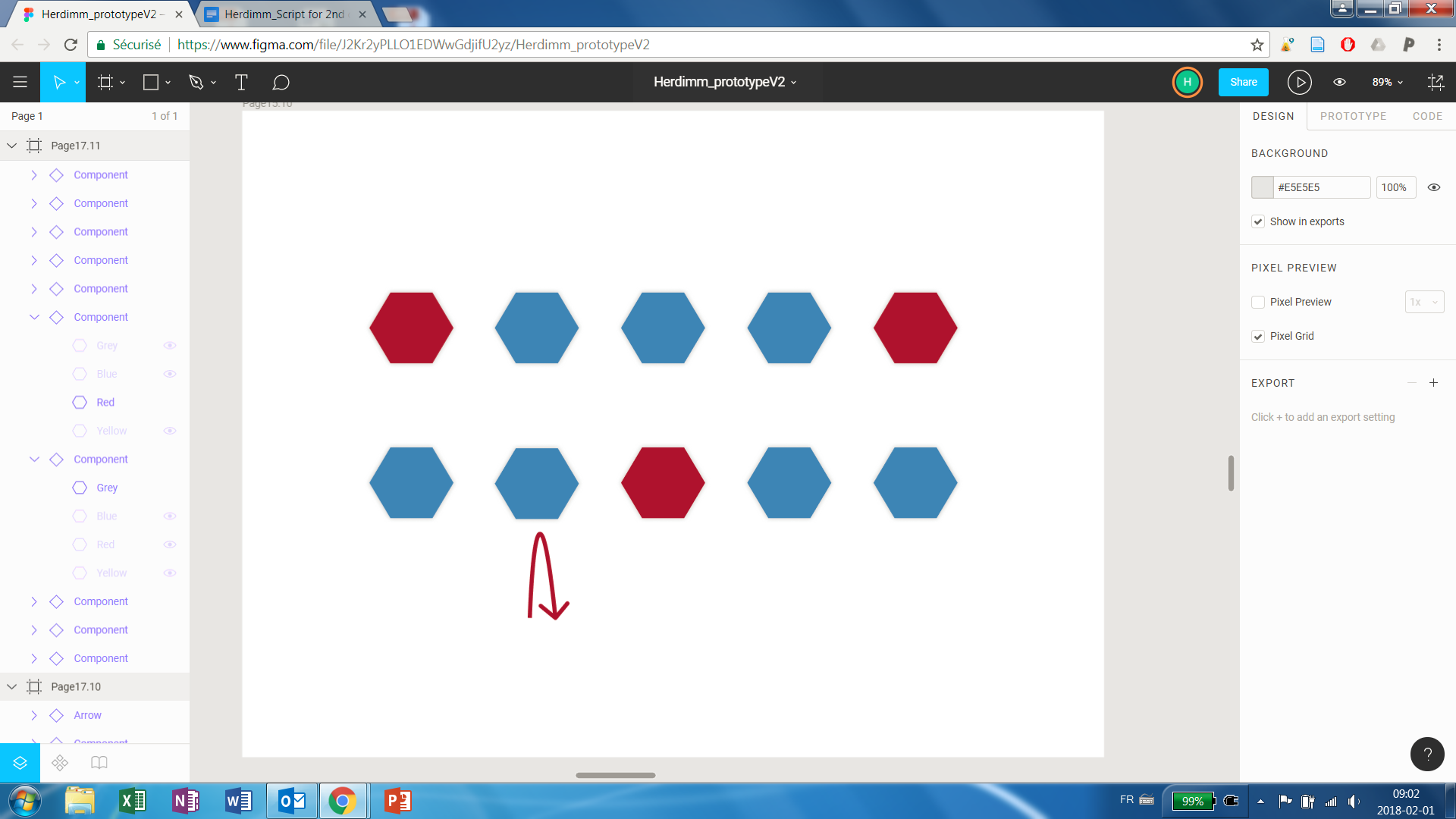 | (bing!) | (bing!) |
| 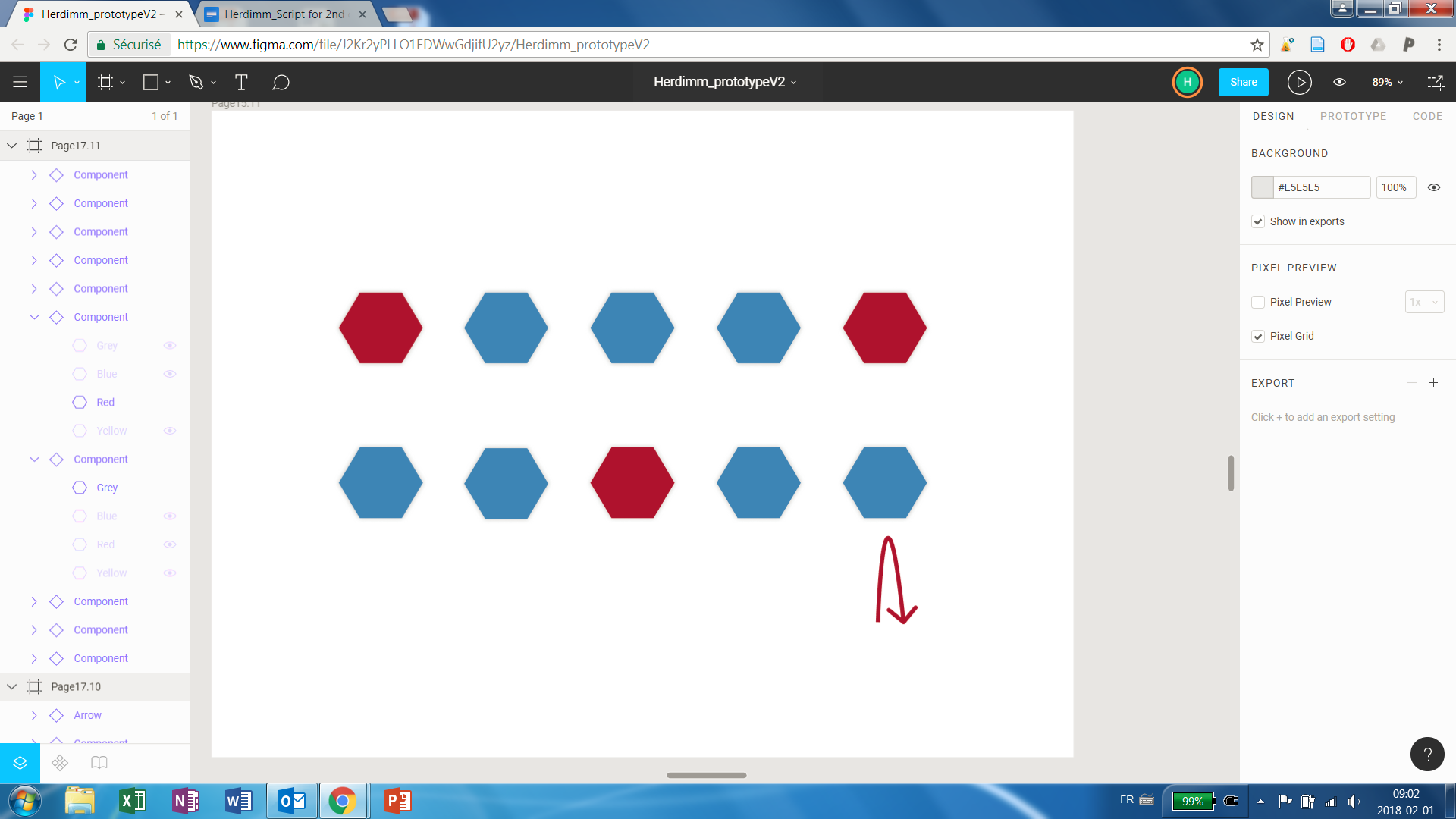 | (bing!) | (bing!) |
| 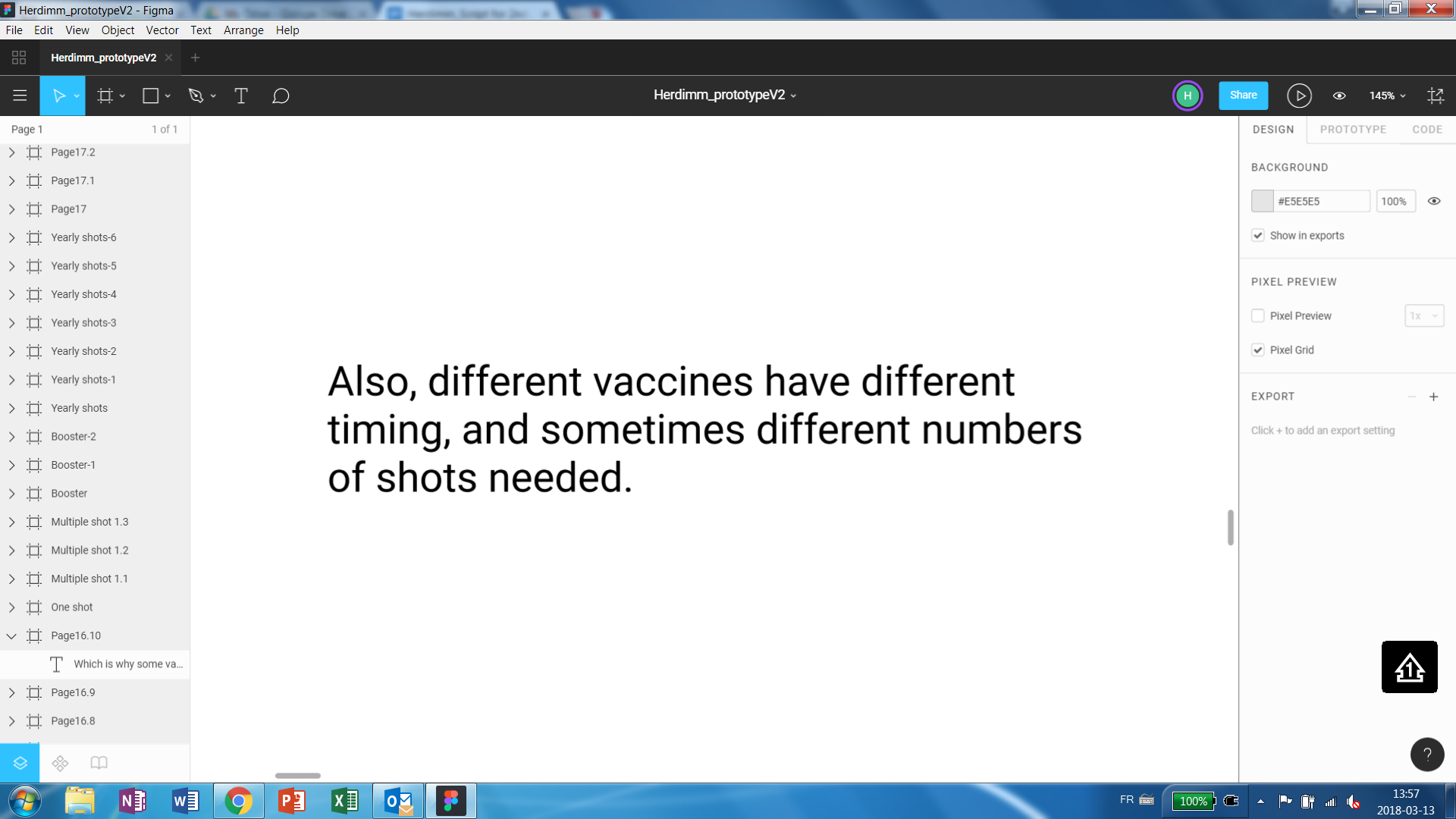 | Also, different vaccines have different timing, and sometimes different numbers of shots needed. | La protection de certains vaccins faiblit au fil du temps et parfois un nombre différent de doses de vaccins est nécessaire. |
| 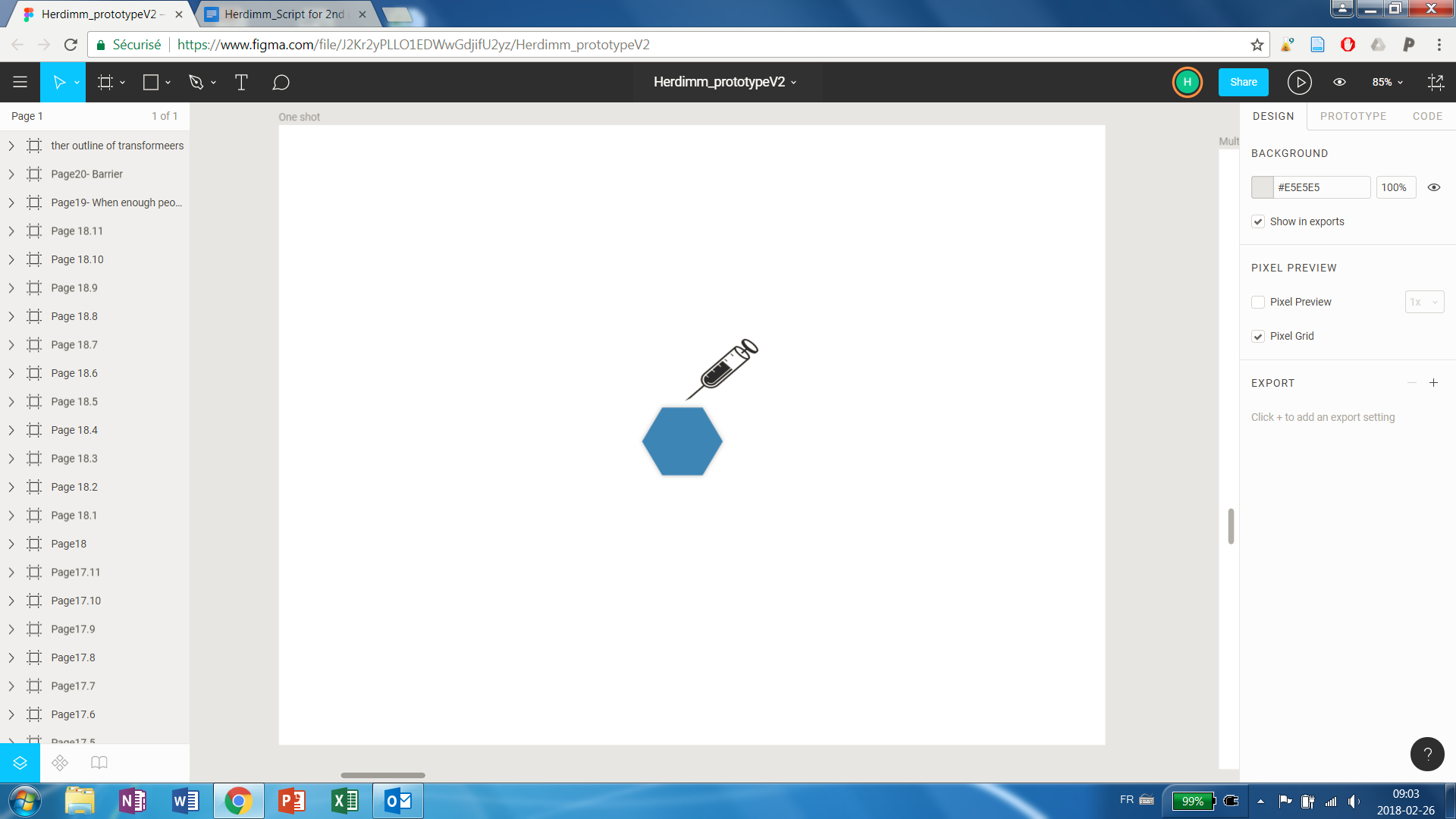 | Some vaccines need just one shot. | Certains vaccins ont besoin d'une seule dose. |
| 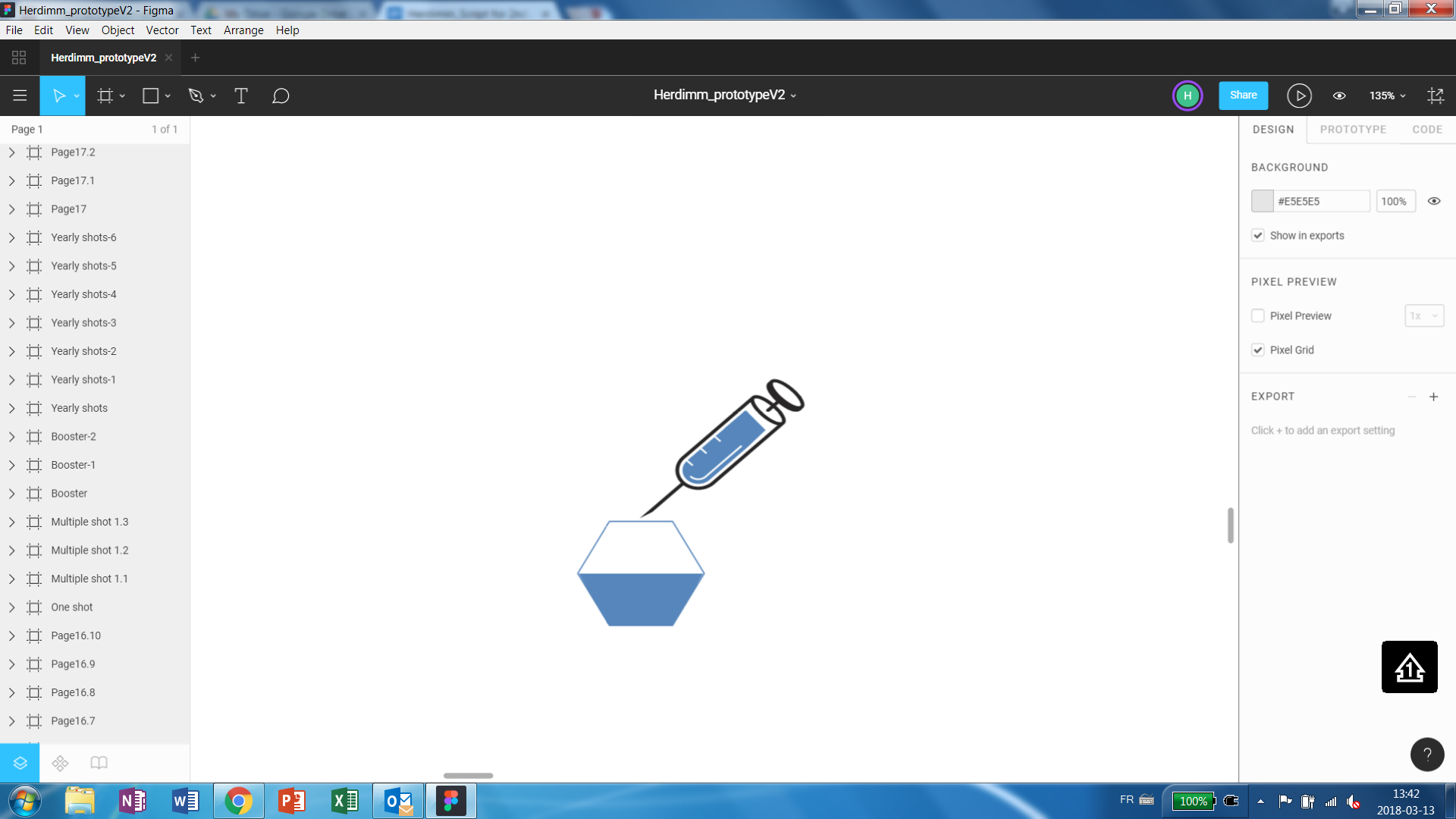 | Other vaccines need multiple shots to achieve full immunity. | D'autres vaccins nécessitent plusieurs doses pour assurer une immunité complète. |
| 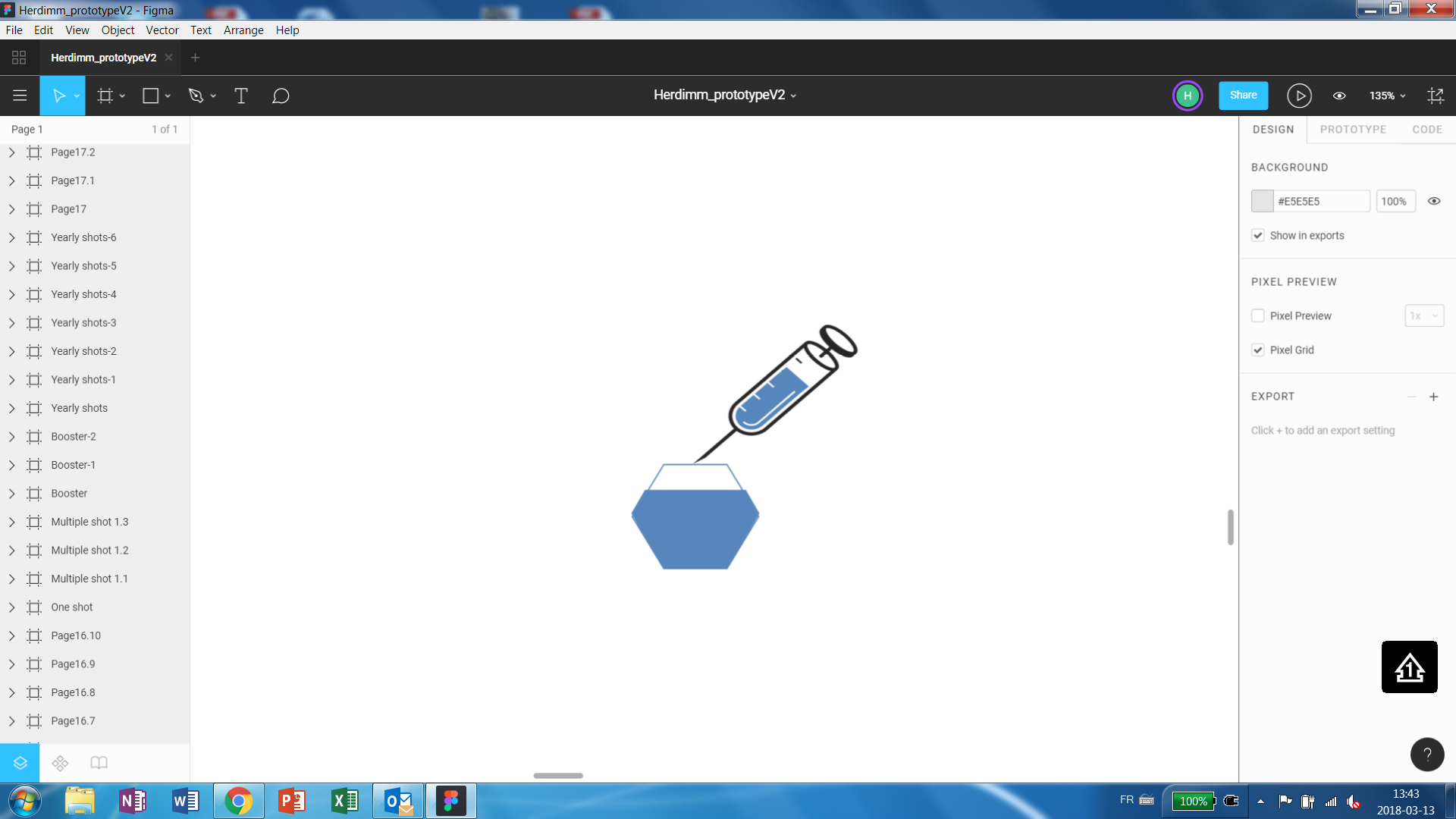 |  |  |
| 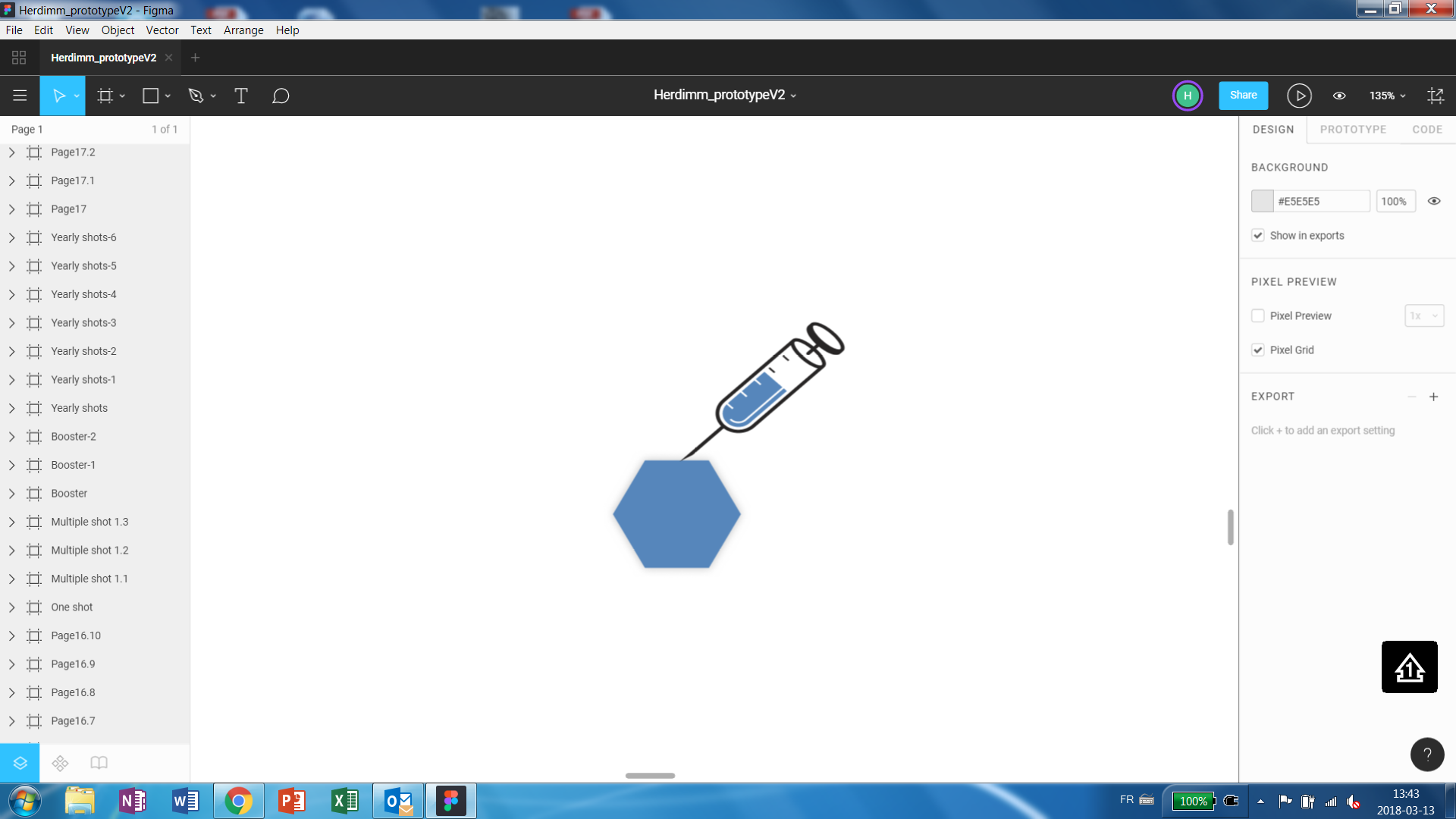 |  |  |
| 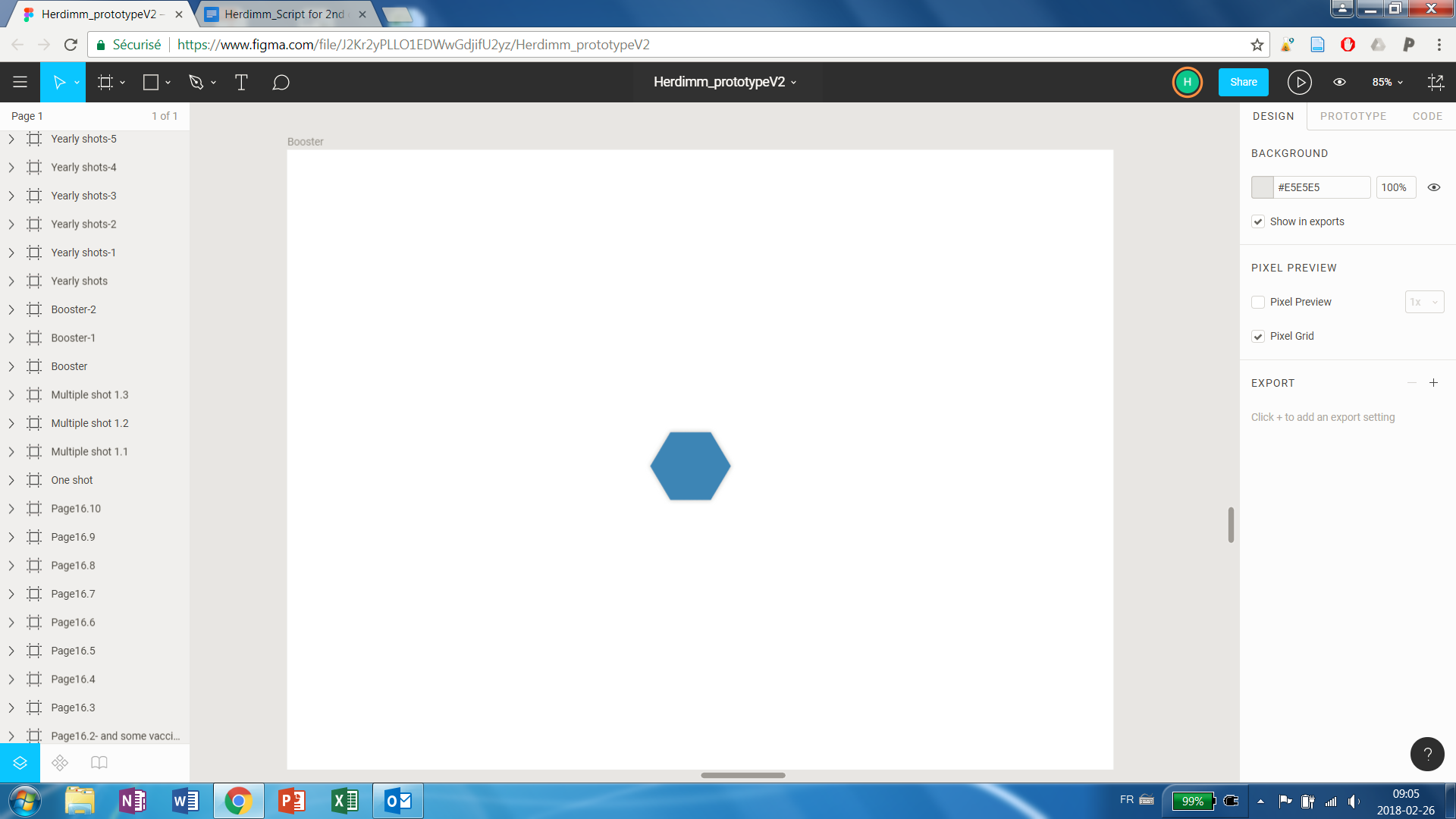 | Some vaccines... | Certains vaccins offrent une protection qui ... |
| 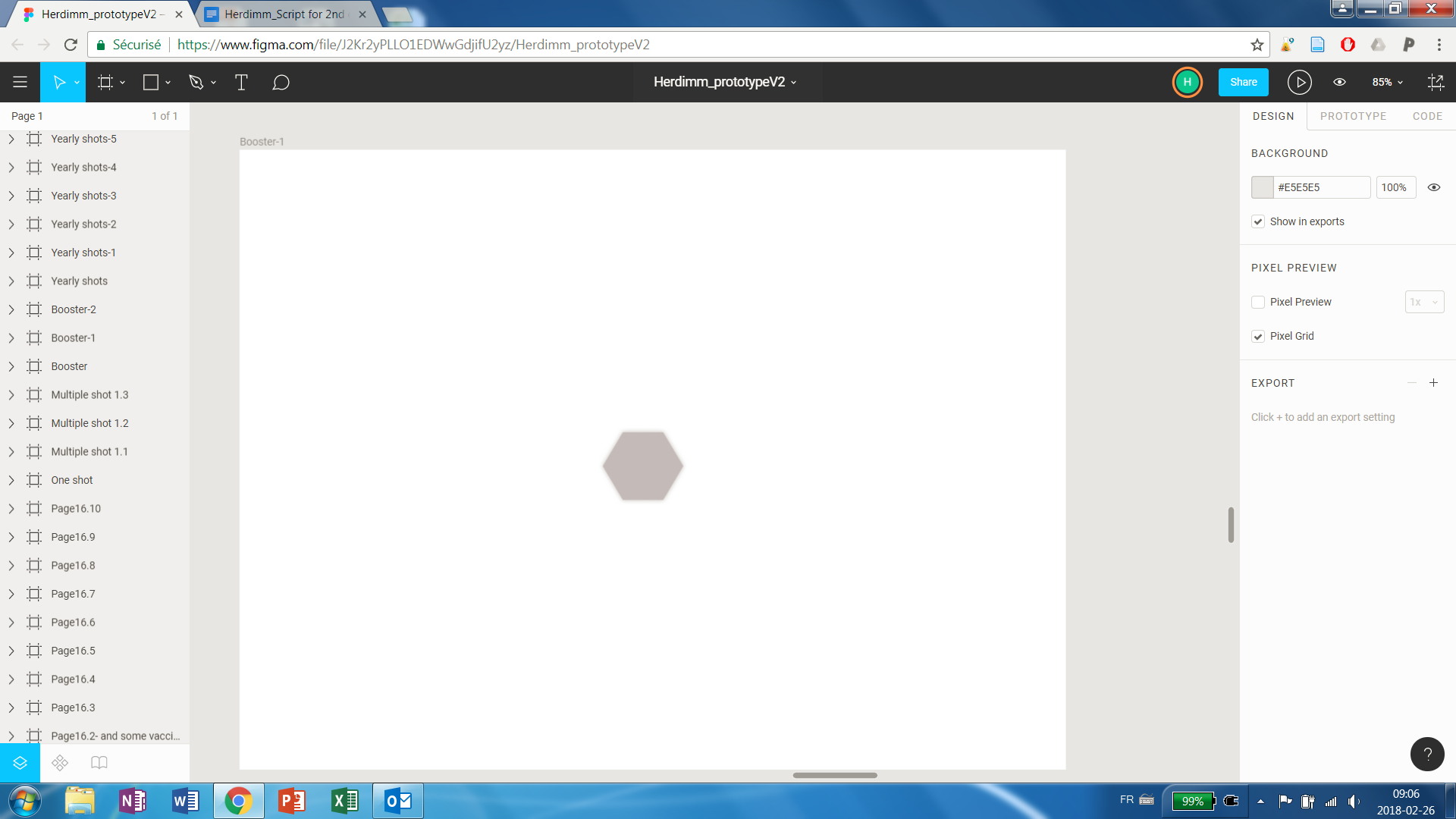 | wane over time ... | faiblit avec le temps ... |
| 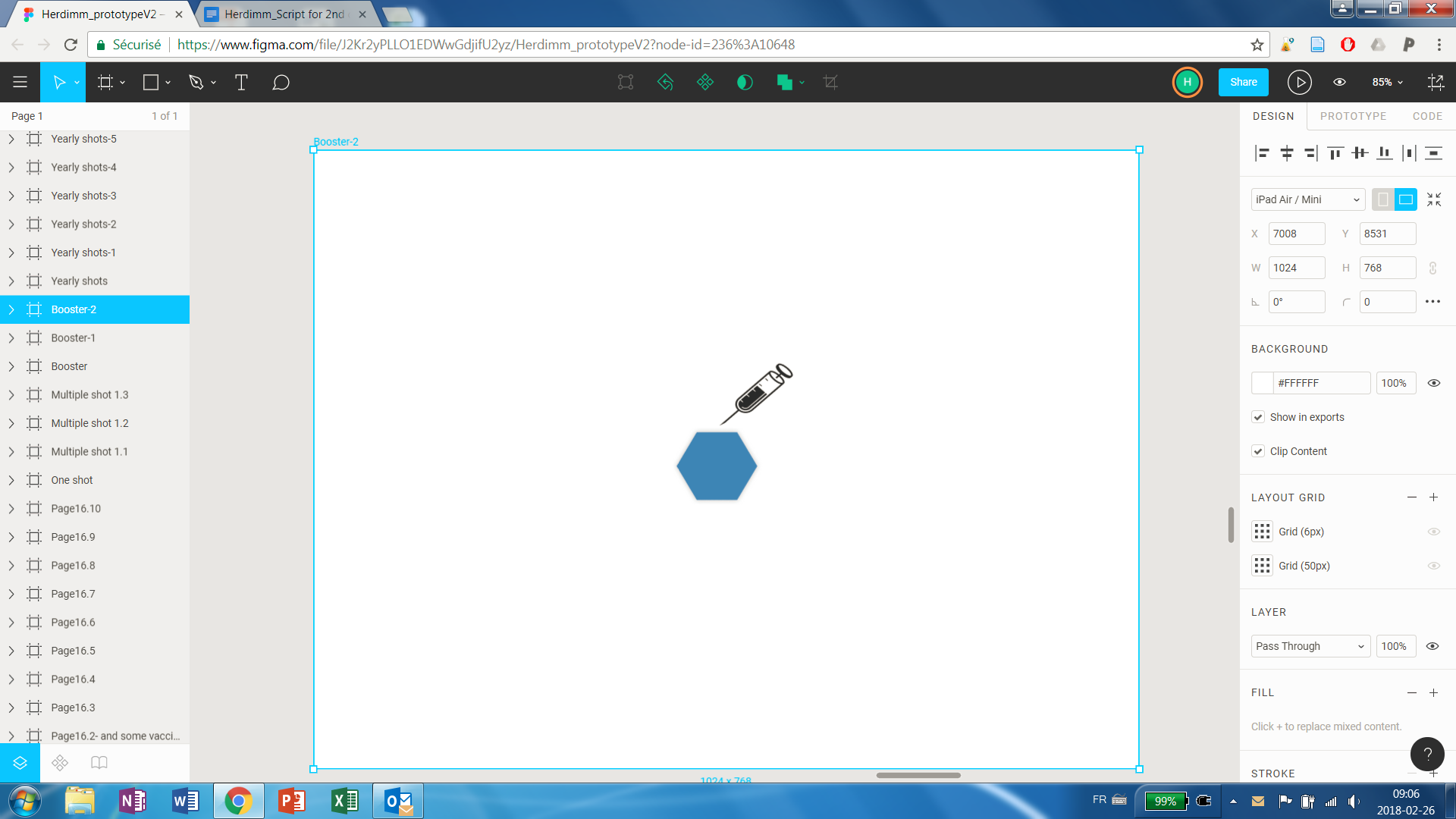 | … and need boosters. | ... et ont besoin de doses de rappels. |
| 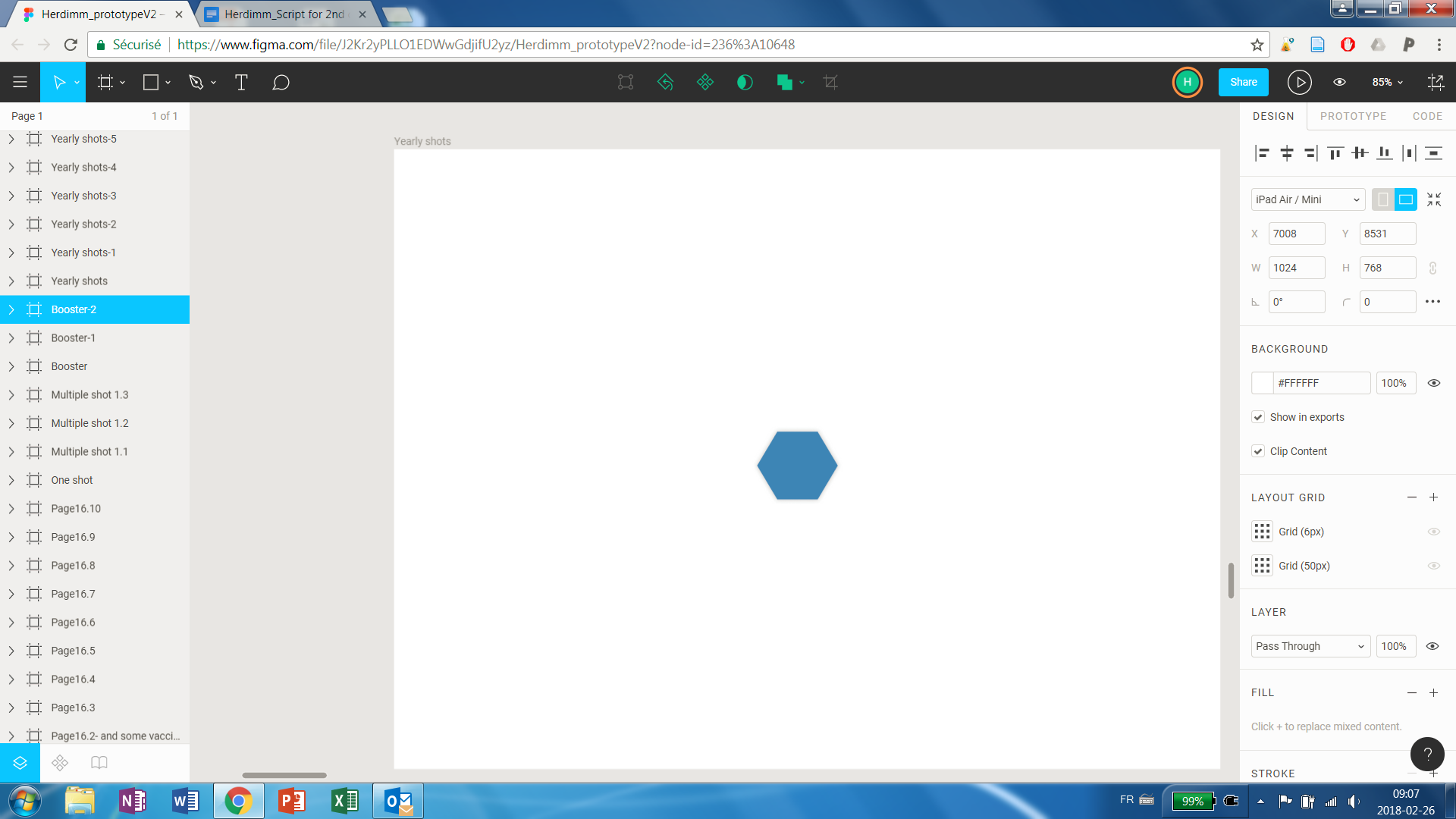 | Some need annual shots, possibly because the disease changes from year to year... | Certaines infections nécessitent des vaccins annuels, parfois parce que la maladie change d'année en année ... |
| 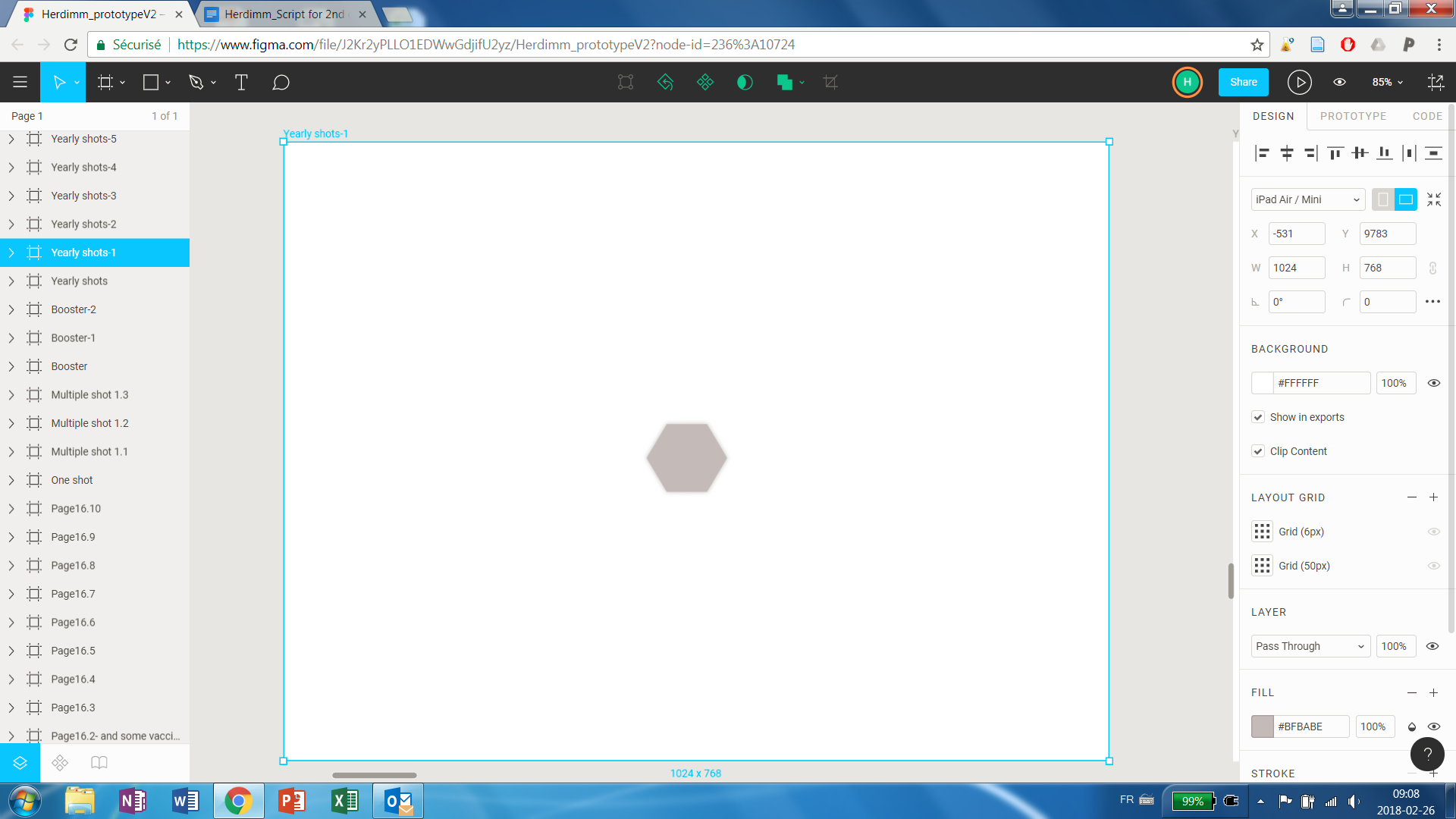 |  |  |
| 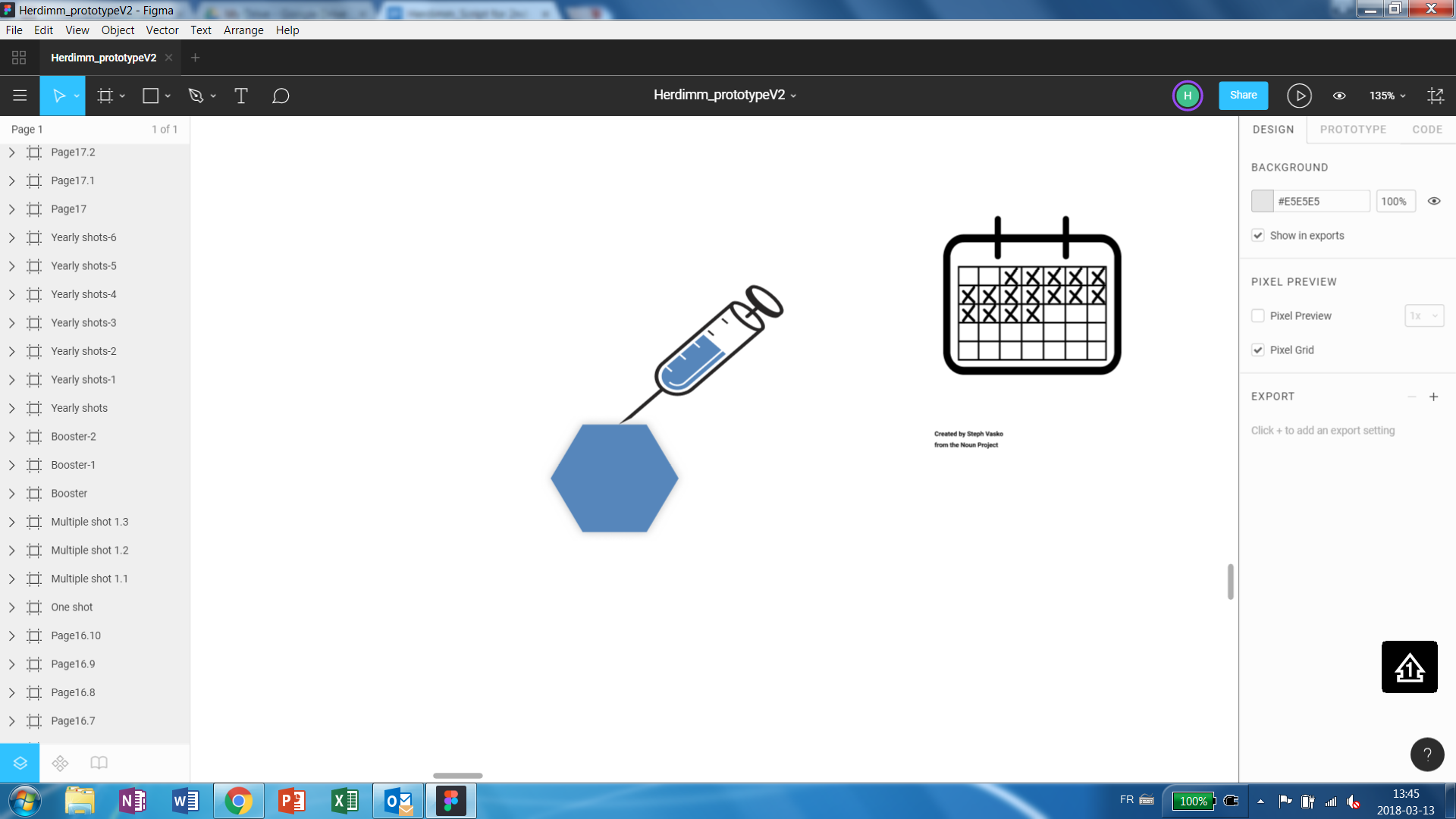 |  |  |
| 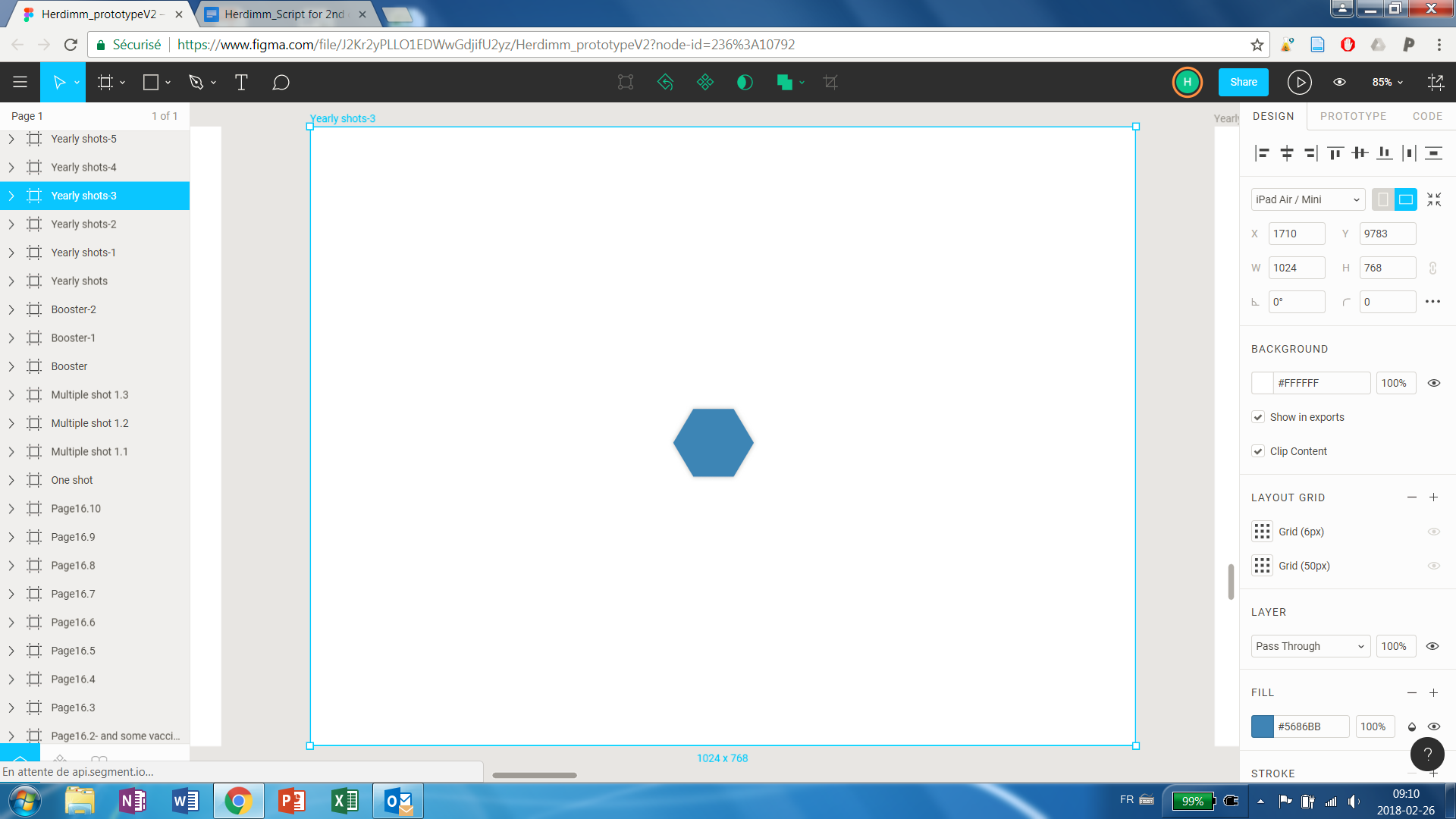 |  |  |
| 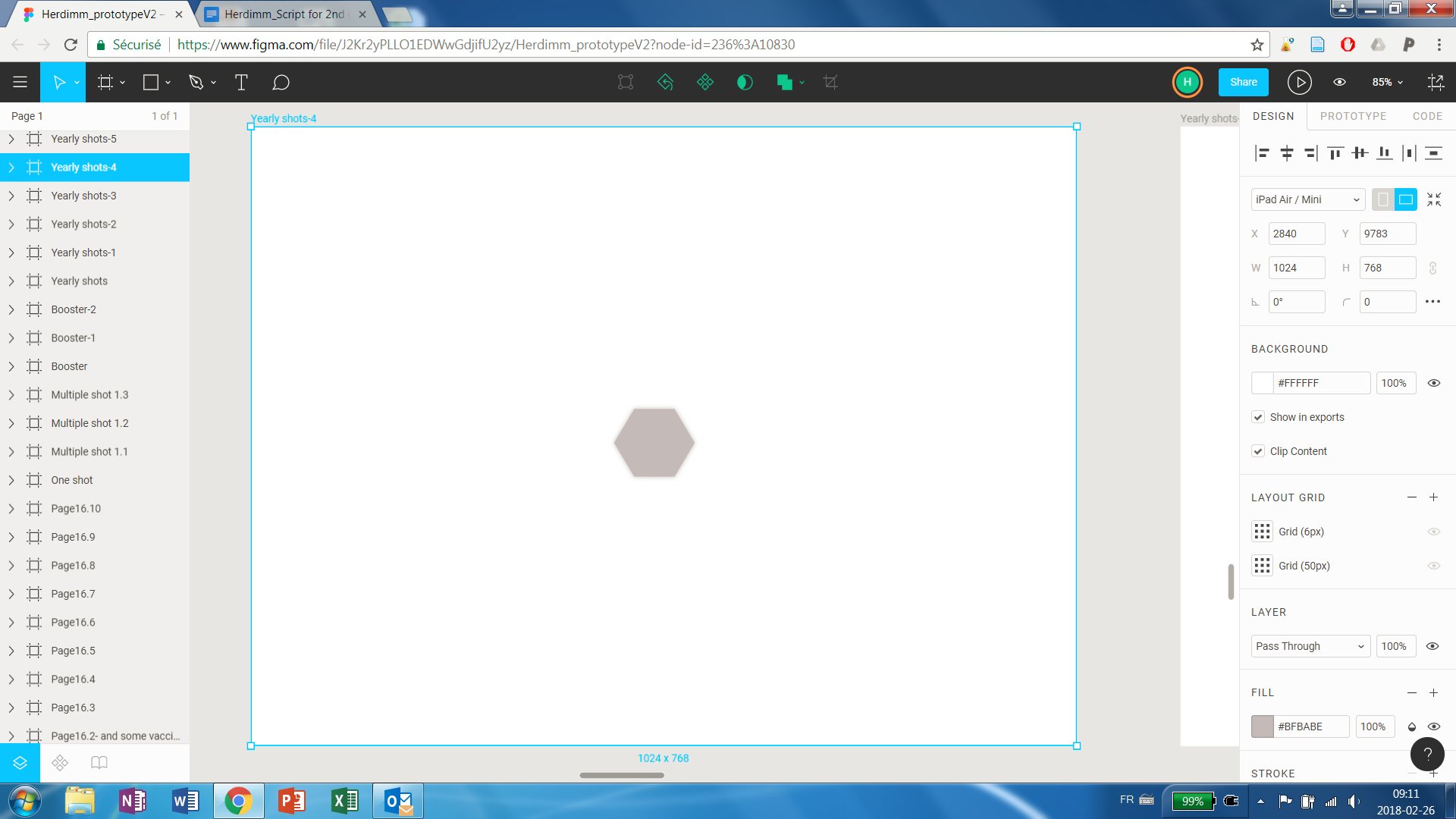 |  |  |
| 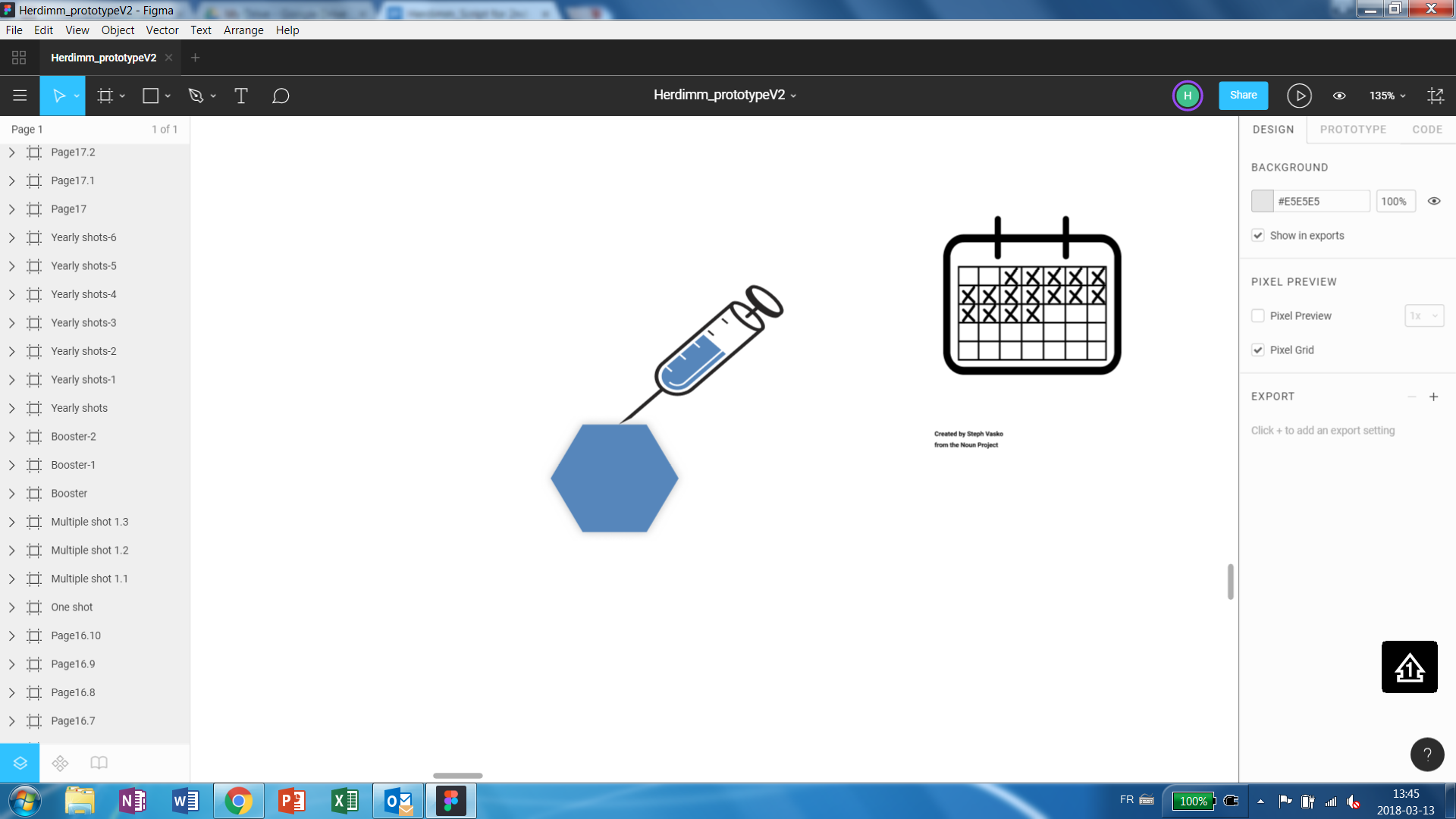 |  |  |
| 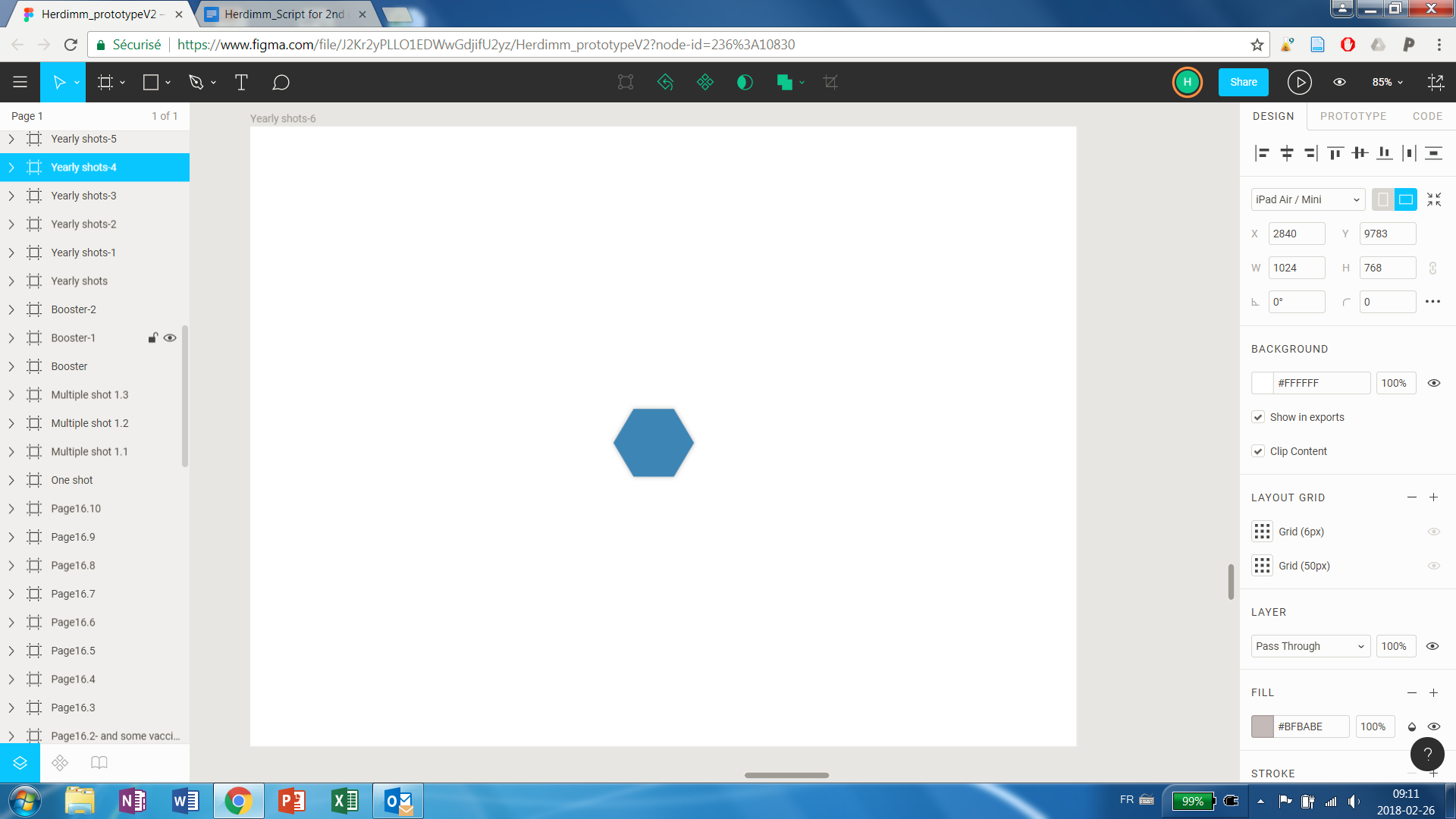 |  |  |
| 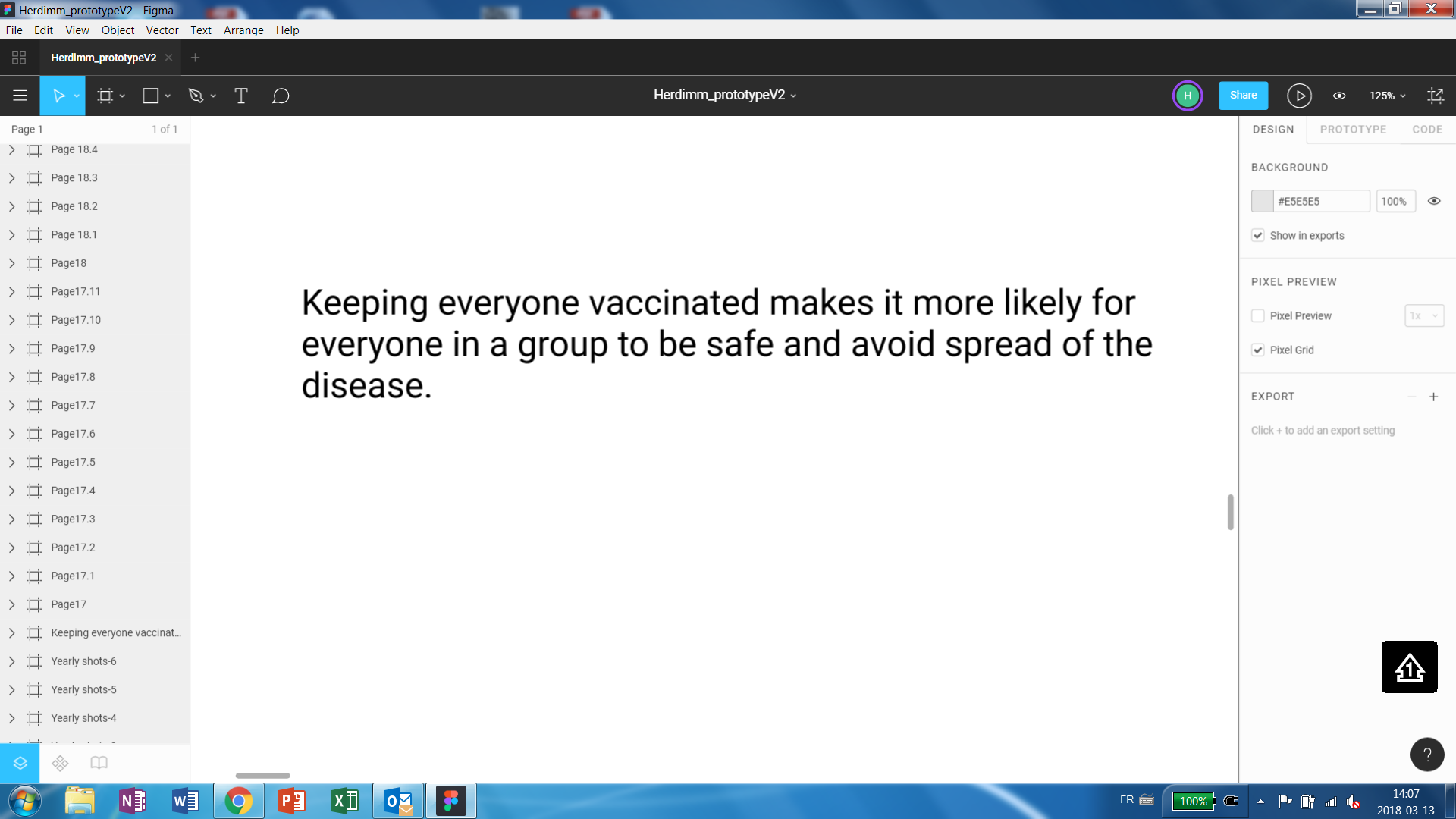 | Keeping everyone vaccinated makes it more likely a group to be safe and avoid spread of the disease. | Lorsque tout le monde est vacciné, il est plus probable d'être en sécurité dans un groupe et d'éviter la propagation de la maladie. |
| Community Protection | | |
| 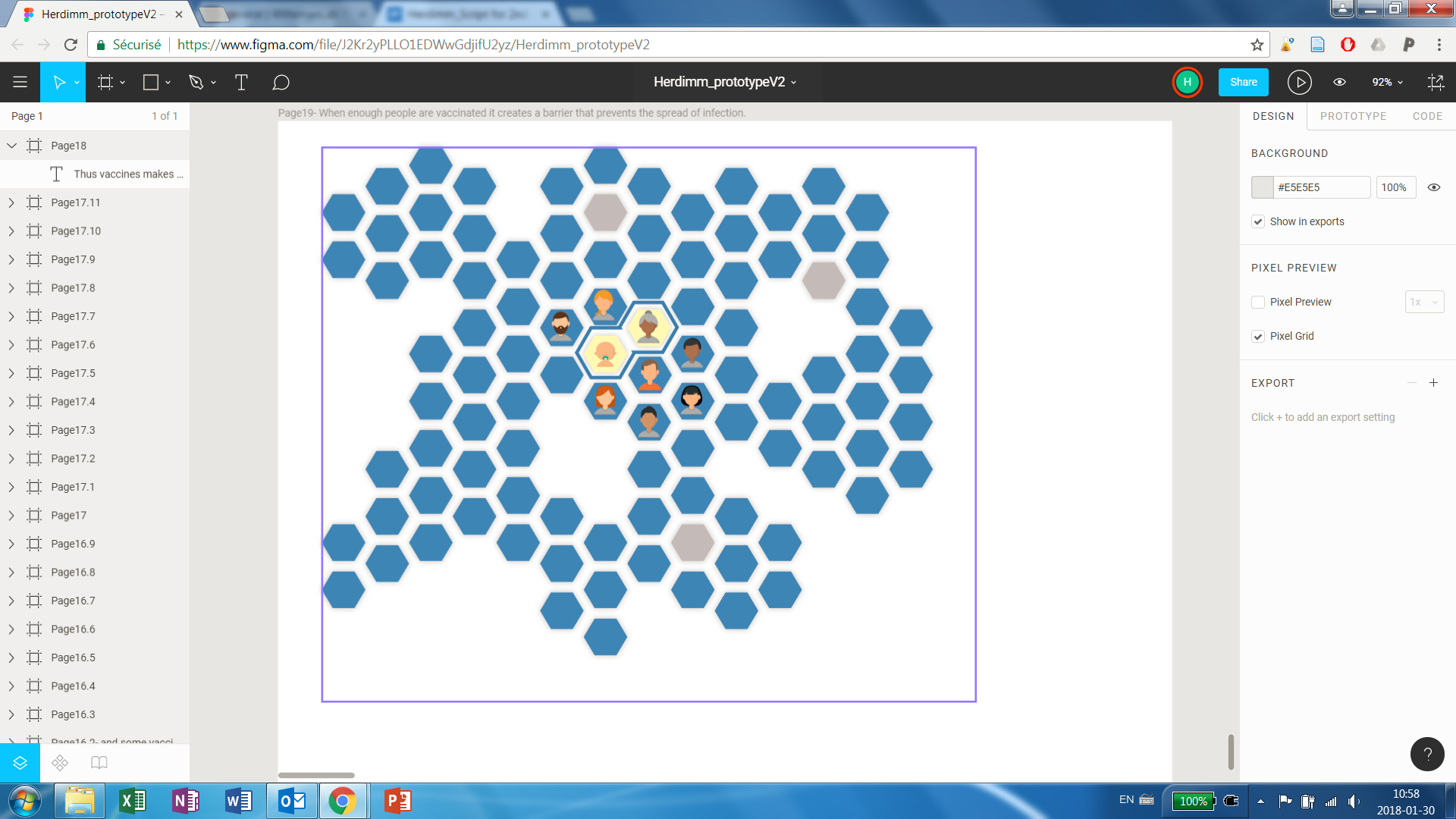 | When enough people are vaccinated they provide a barrier around everyone. | Lorsque suffisamment de personnes sont vaccinées, il se forme une barrière protectrice qui touche à toute la communauté. |
| 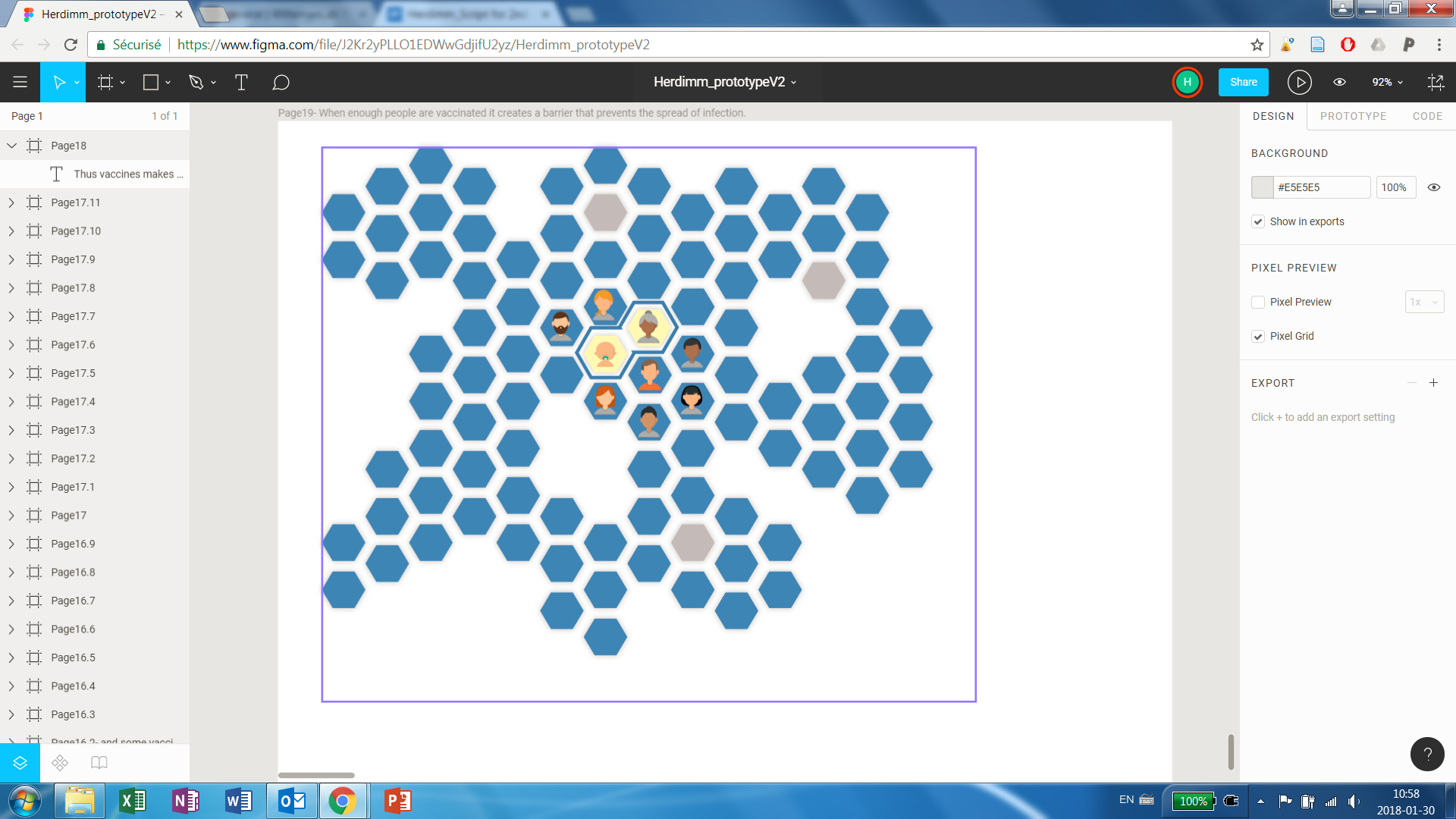 | This barrier is known as community protection. | Cette barrière est connue sous le nom d’immunité collective. |
| 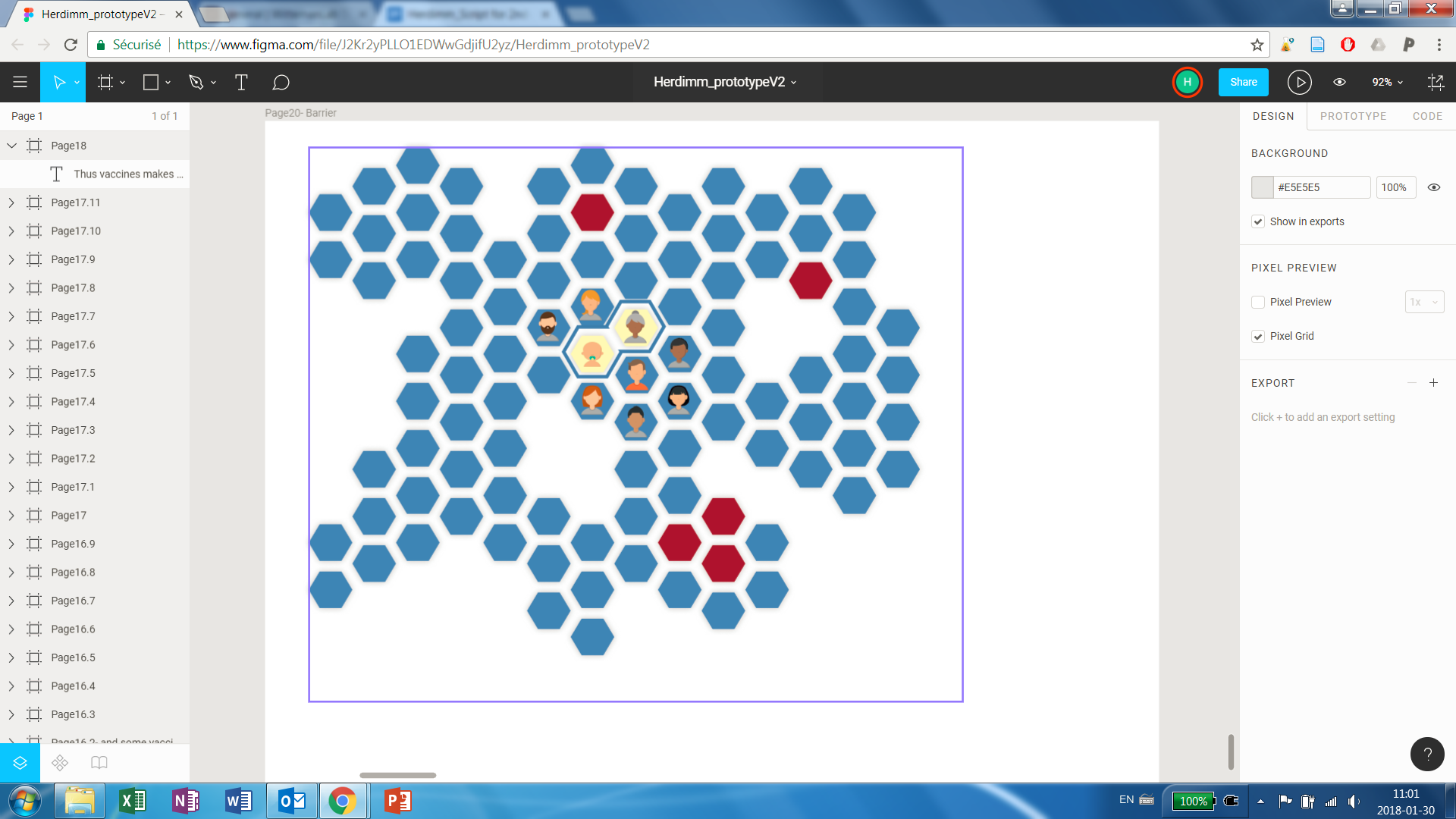 | So when a disease enters a community ... | Alors, si une infection entre dans la communauté |
| 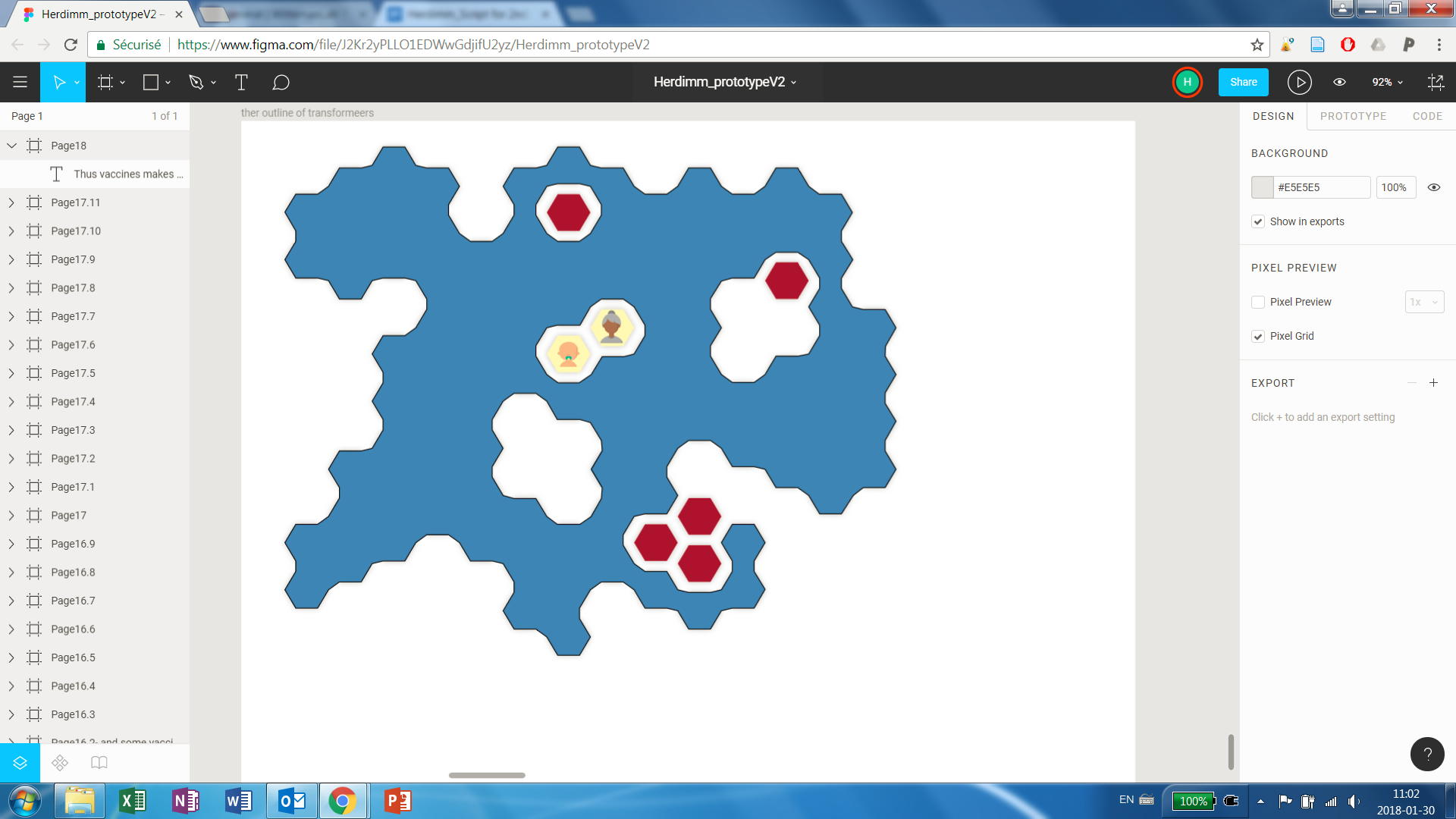 | … this barrier of community protection protects many people in your community by stopping the spread. The disease can’t get past people who got vaccinated and are immune. | Cette barrière, l’immunité collective, peut protéger les gens en arrêtant la propagation de la maladie. La maladie ne peut pas dépasser les personnes vaccinées et immunisées. |
| 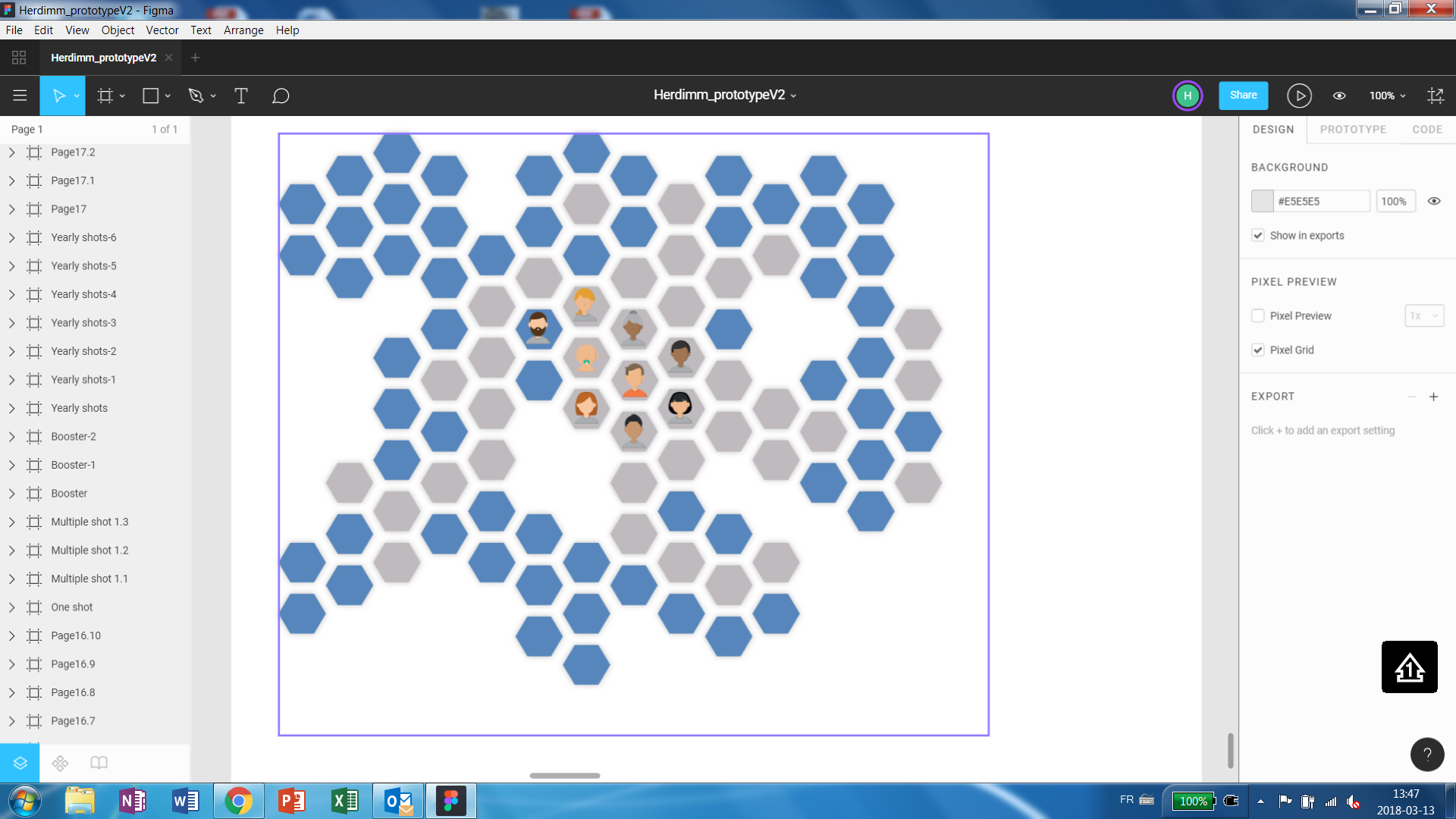 | But if **not** enough people are immune... | Mais si trop peu de gens sont immunisés .. |
| 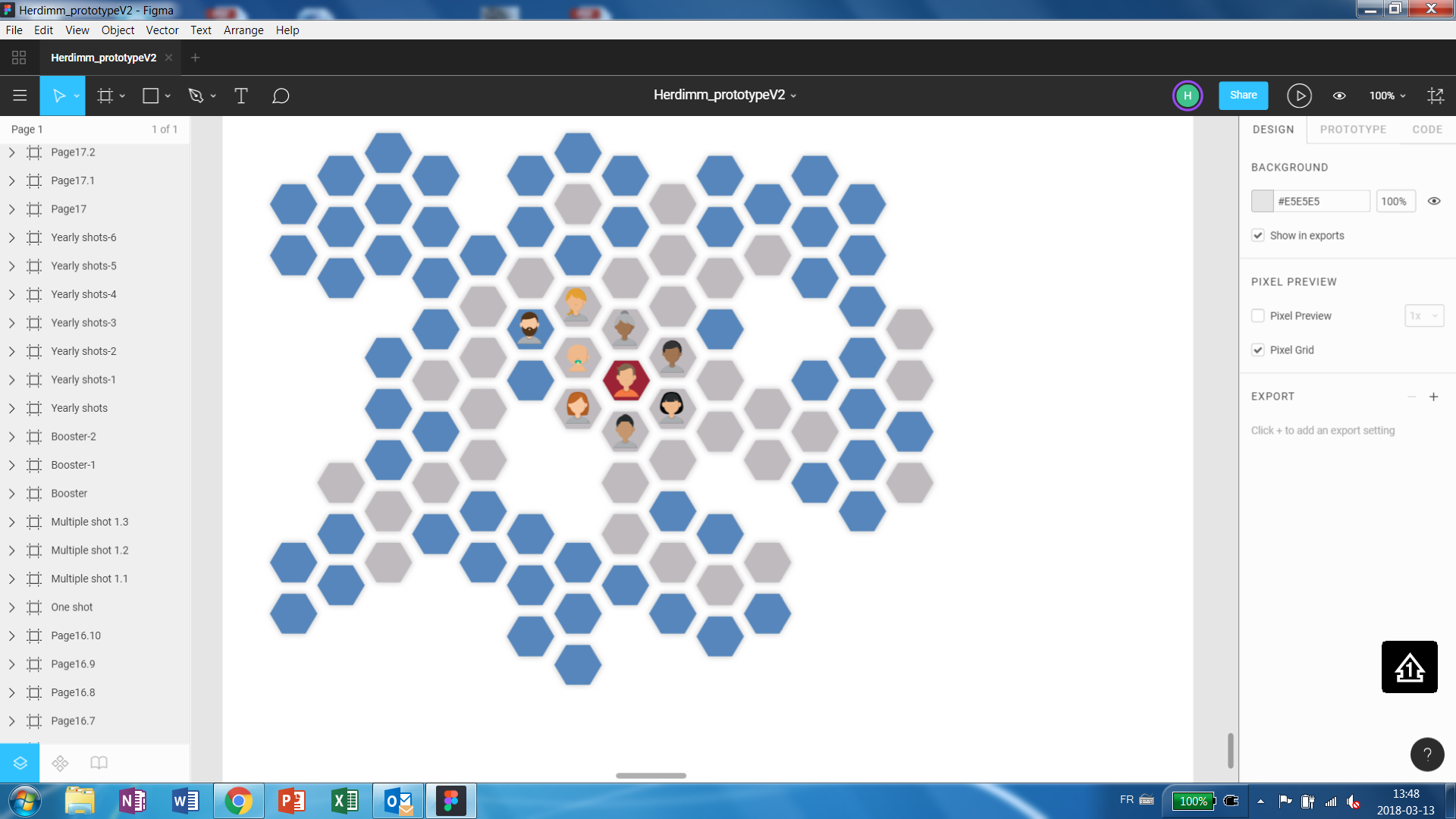 | …..the disease can easily spread | ... ..la maladie peut facilement se propager. |
| 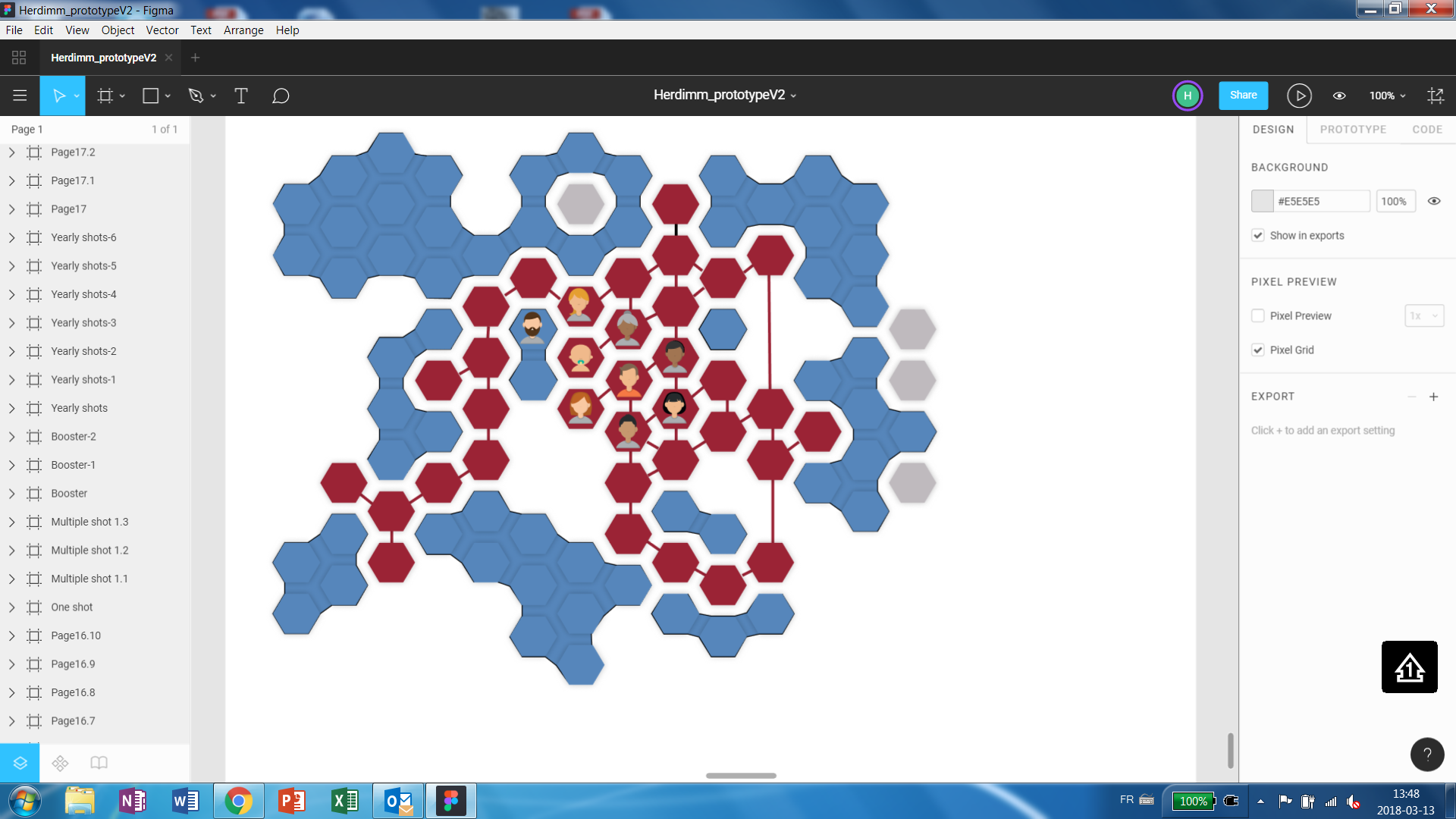 | ….and can reach you, the people around you, and others in your community, including people who are the most vulnerable. | La maladie pourra ainsi vous atteindre, atteindre les gens autour de vous et les autres membres de votre communauté, y compris les personnes les plus vulnérables. |
|  | | |
| [Conclusion] [may want to fade back to blue version with protection? Might need more transition] | This means that your decision to get vaccinated or not has an impact on the people around you, and especially on the vulnerable people in your community.  *Test with and without:* Your decision to vaccinate could help save somebody else’s life. | Cela signifie que votre décision de vous faire vacciner ou non a un impact sur les gens autour de vous, et particulièrement sur les personnes vulnérables de votre communauté. |
