# Supplementary material for: A Web Application About Herd Immunity Using Personalized Avatars: Development Study
Source: J Med Internet Res. 2020 Oct 30;22(10):e20113. doi: 10.2196/20113 (PMC7665952; doi:10.2196/20113)
Supplement: Multimedia Appendix 4 [file jmir_v22i10e20113_app4.docx]

**Appendix 4: Script for cycle 3**

| **Visuel** | **English narration** | **Narration française** |
| --- | --- | --- |
| [Introduction]  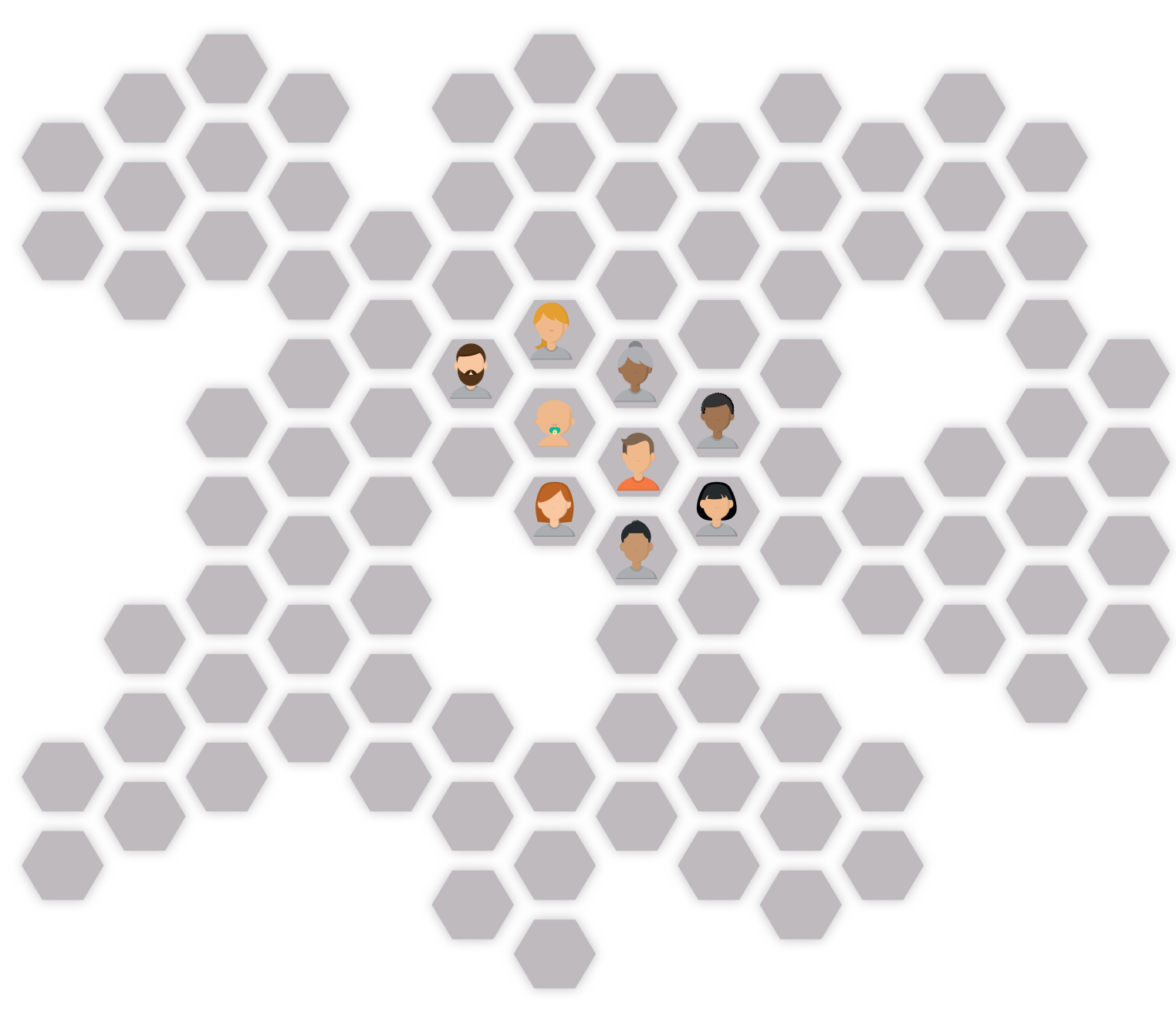 | What role does each of us play in protecting our community? | Quel est le rôle de chacun dans la protection de la communauté? |
| Community | | |
| 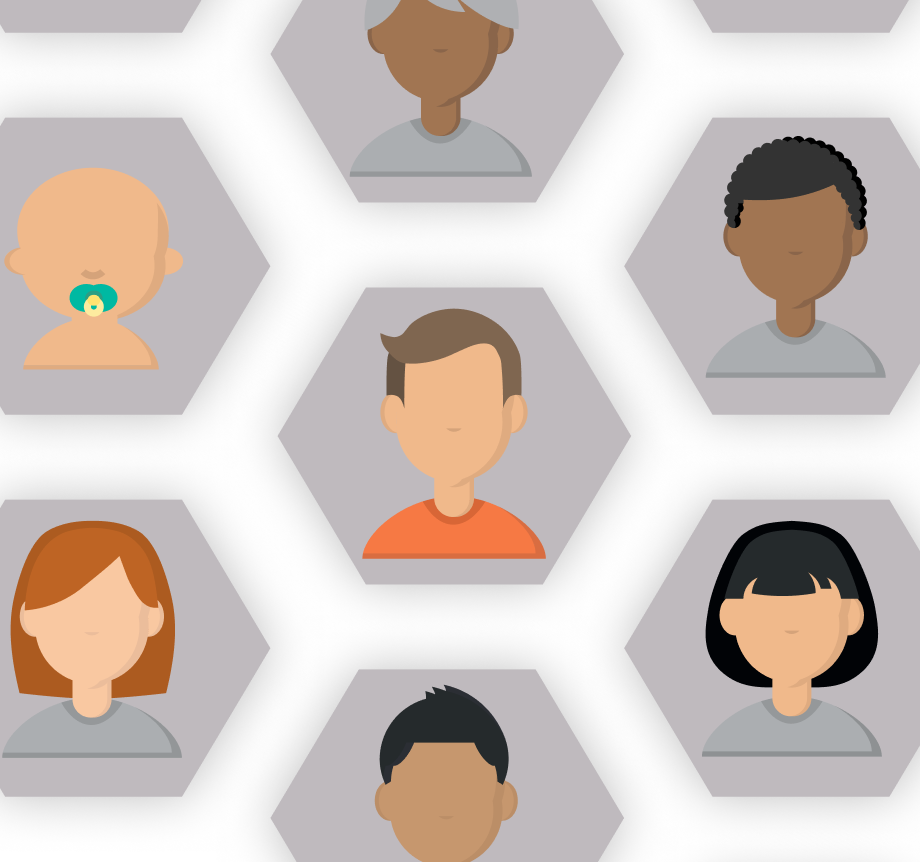 | Imagine this is you. | Imaginez que c’est vous |
| 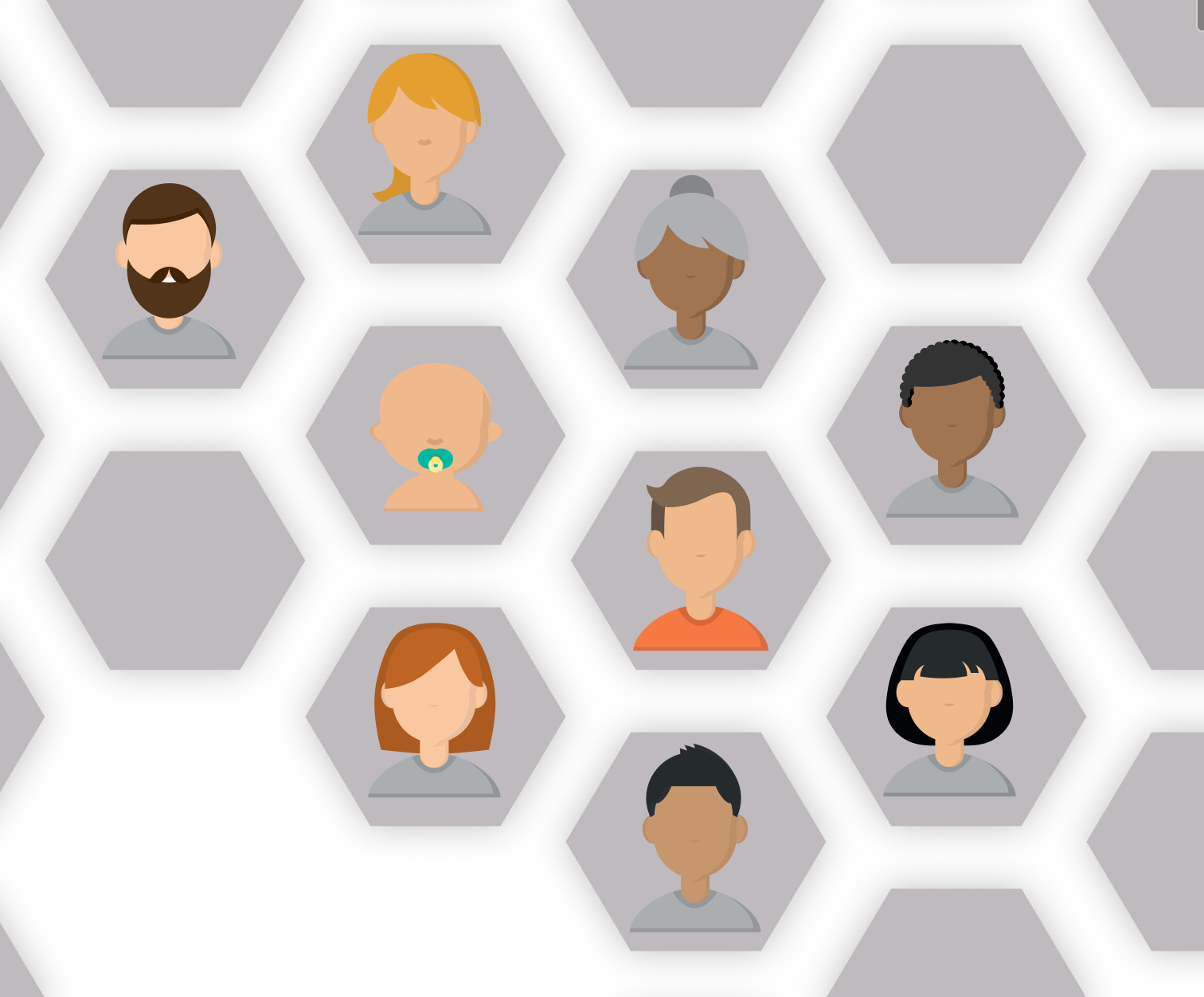 | These are people around you--people you see often, like your family or your coworkers, and then the people around *them* that *they* see often. | Voici des personnes que vous côtoyez souvent, comme votre famille ou vos collègues, puis les personnes qu’eux-mêmes côtoient souvent |
| 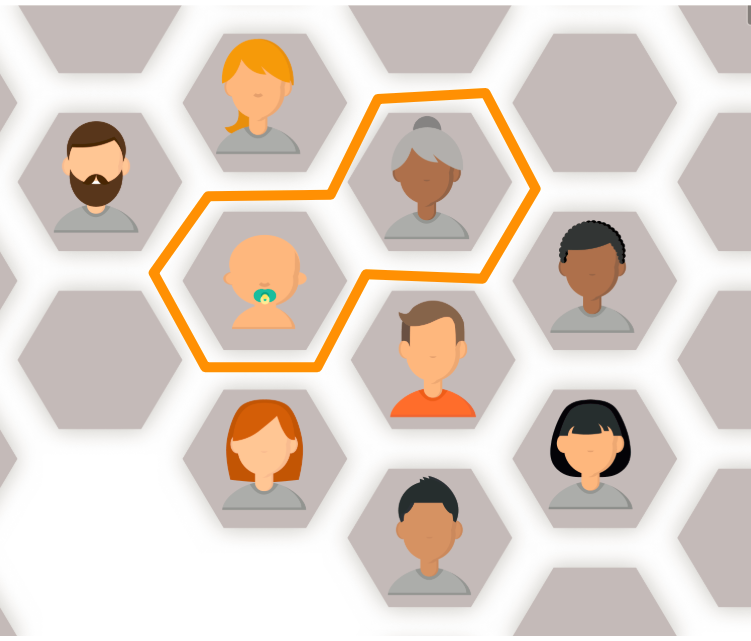 | Some of these people, like babies, older people, or those with fragile immune systems (for example, cancer patients) ... | Il y a des personnes dans ce groupe, par exemples, des bébés, des personnes âgées ou des personnes avec un système immunitaire affaibli comme les patients atteints du cancer... |
| 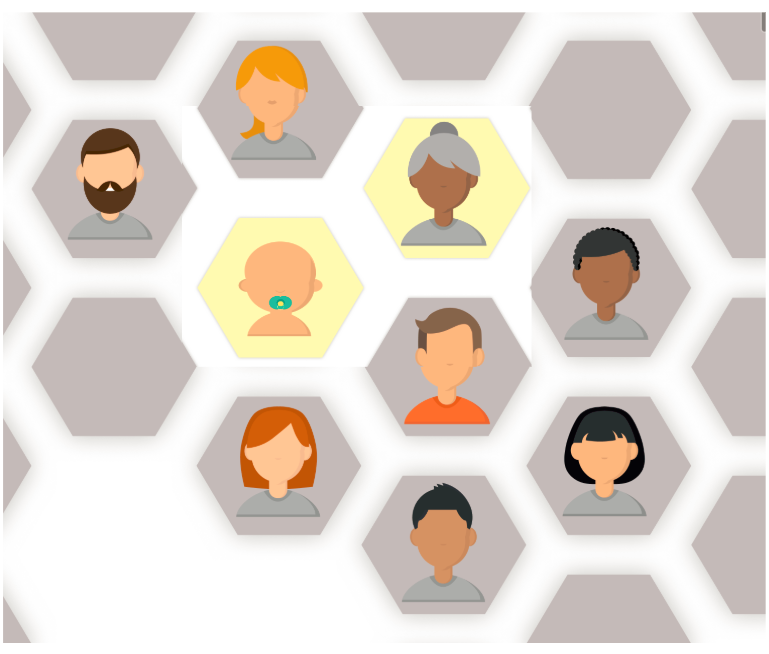 | … are more vulnerable to diseases, either because they can catch them more easily, or the diseases can make them sicker. | ...Ces personnes sont considérées comme vulnérables. aux maladies, car elles peuvent les attraper plus facilement et les maladies peuvent les rendre plus malades |
| Infection/Disease | | |
| 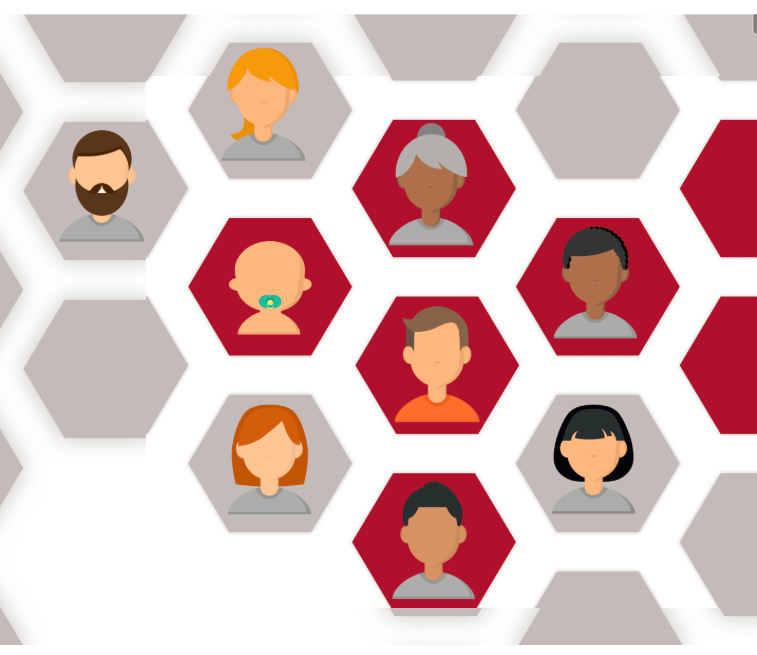 | When a contagious disease is going around, anyone can catch it, pass it on to others, get sick, or even die from it, especially vulnerable people. | Lorsqu’une maladie contagieuse se propage dans un groupe, n'importe qui peut l’attraper, la transmettre aux autres, tomber malade ou même mourir, surtout les personnes vulnérables. |
| Vaccines | | |
| 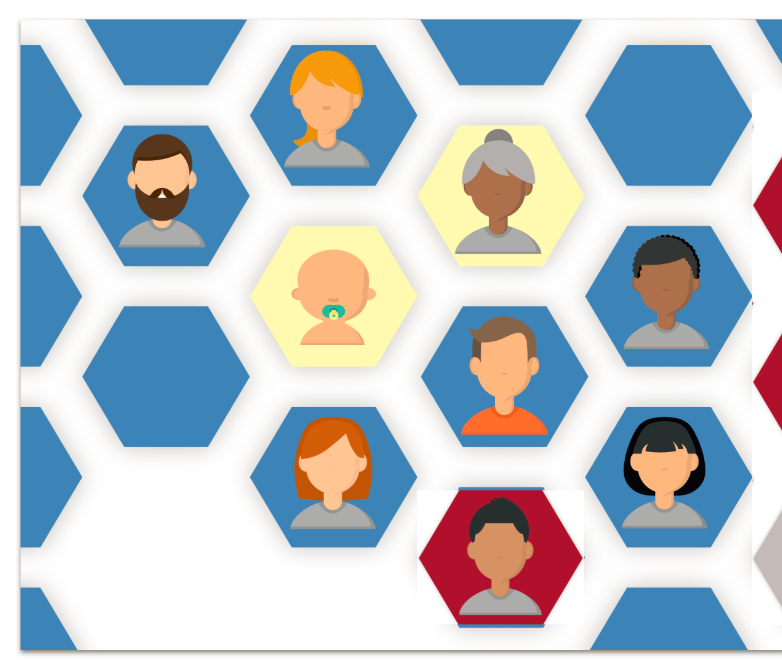 | Some contagious diseases have vaccines to protect us. Vaccines train your immune system--your body’s protection system--to fight against diseases. People who are vaccinated are less likely to catch and pass on the disease.  Sometimes, vulnerable people may not be able to be vaccinated, or the vaccines might not work as well for them because their body doesn’t respond as well to the vaccine. [add panel]  Even for everyone else, vaccines are not perfect. | Des vaccins existent pour nous protéger de certaines maladies contagieuses. Les vaccins entraînent votre corps à lutter contre les maladies. Les personnes vaccinées sont moins susceptibles d’attraper et de transmettre la maladie.  Pour les personnes vulnérables, il n’est pas toujours possible d’avoir un vaccin. Parfois, les vaccins ne fonctionnent pas aussi bien pour elles car leur corps répond moins bien à l’entraînement donné par le vaccin.  Même pour les autres personnes, les vaccins ne fonctionnent pas toujours parfaitement. |
| 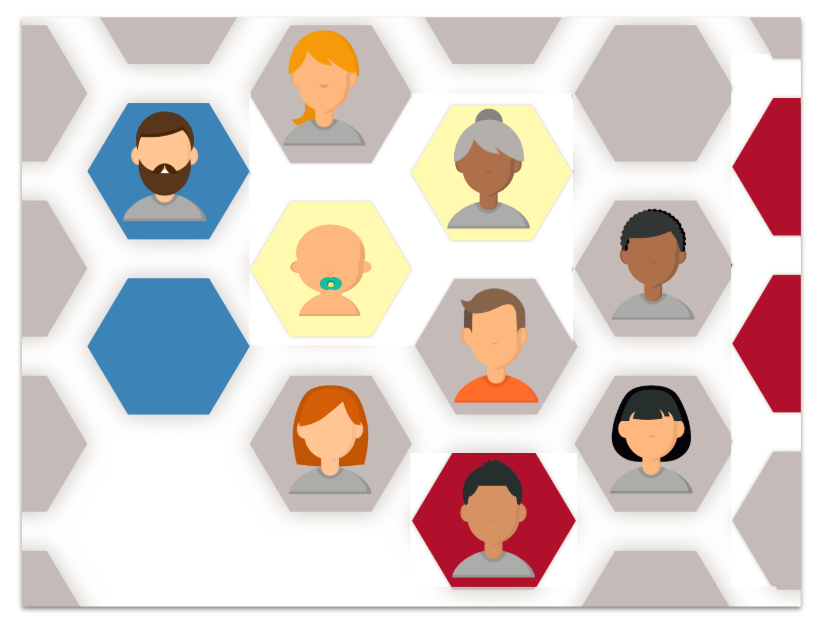 | They don’t work every time, and can wane over time. | Ils ne fonctionnent pas à tout coup et leur efficacité peut diminuer avec le temps |
| 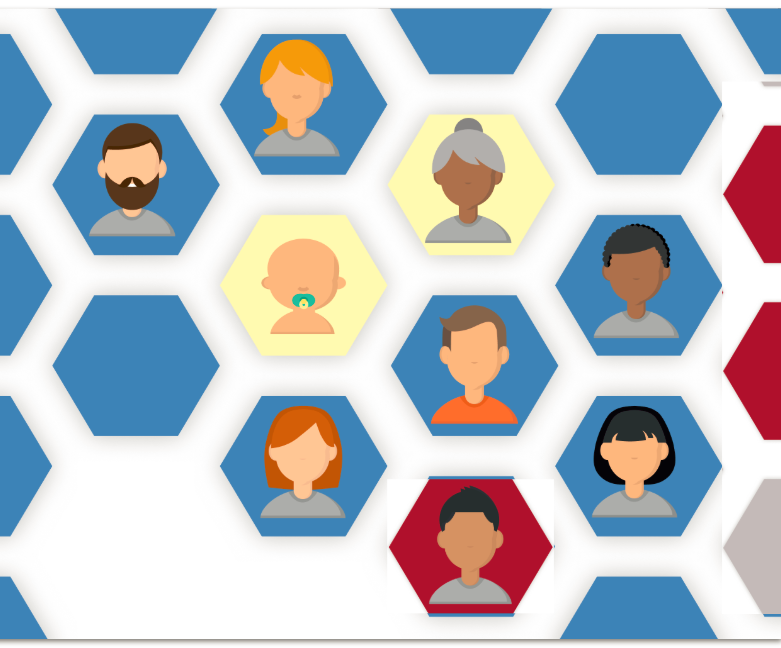 | And need to be updated. | C’est pourquoi ils doivent être maintenus à jour |
| Community Immunity | | |
| 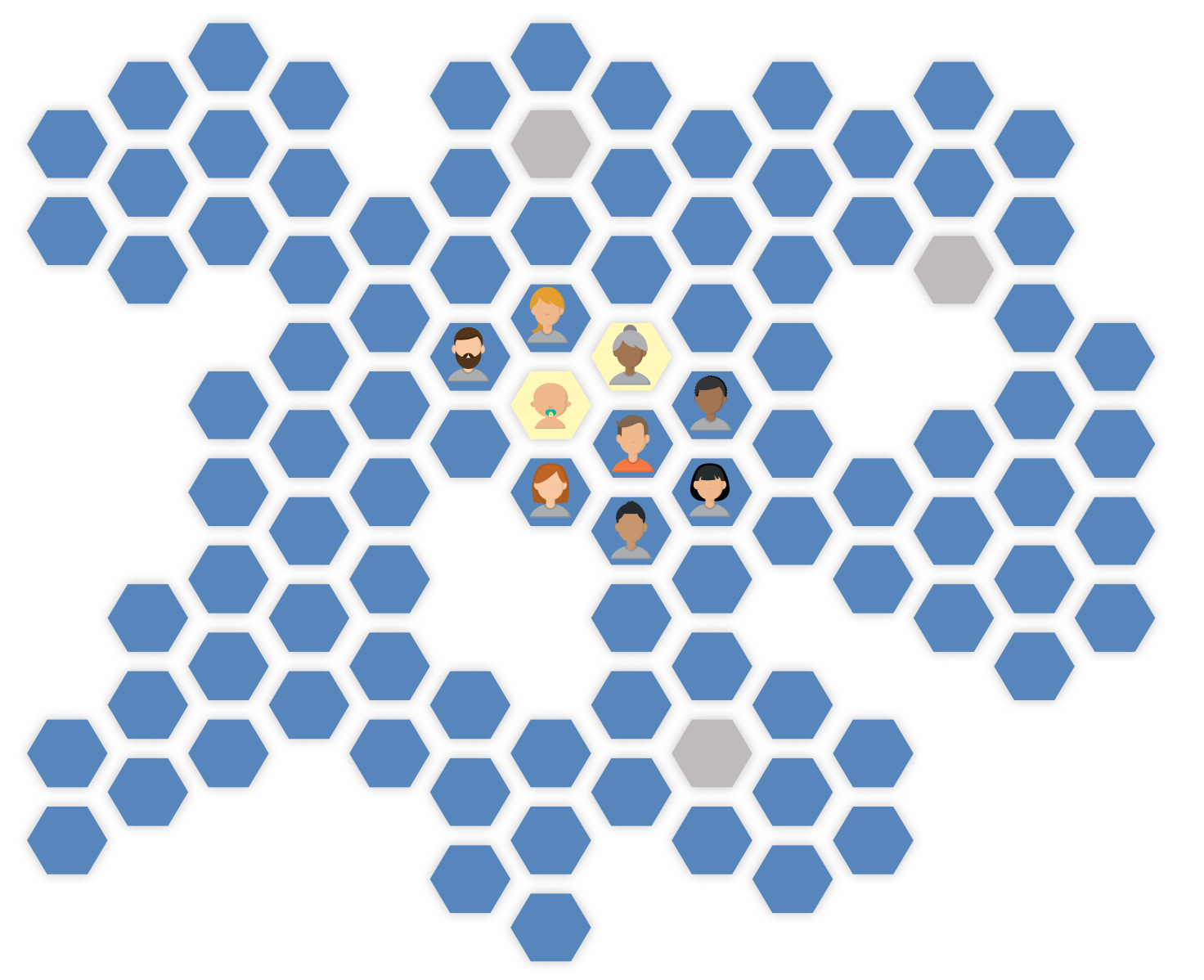 | When you and enough people around you are vaccinated, this creates a protective barrier around everyone in your community. | Quand vous êtes vacciné et qu’assez de personnes autour de vous sont vaccinées cela crée une barrière protectrice autour de toute la communauté. |
| 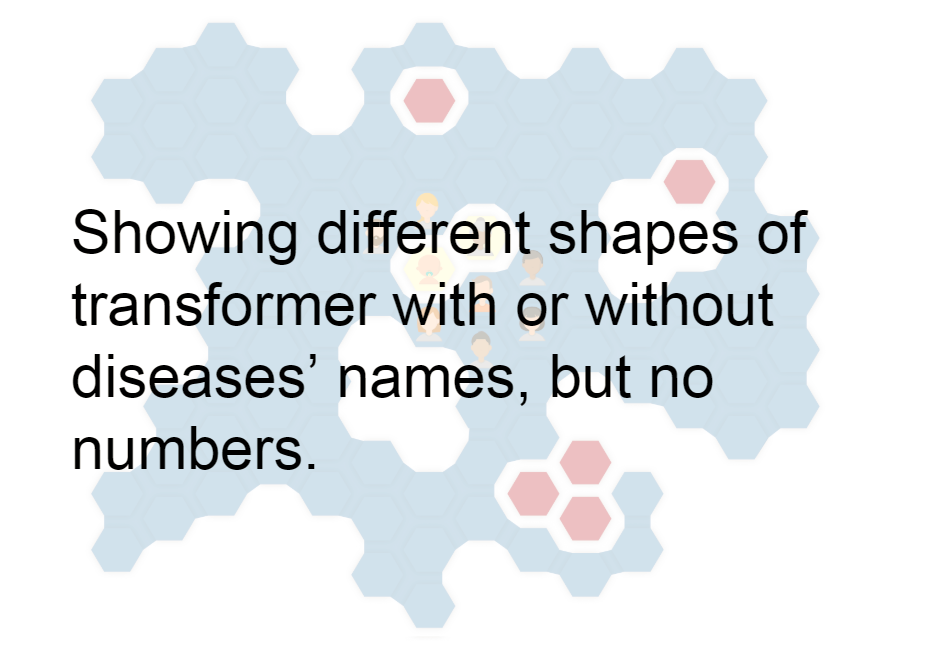 | This protective barrier is different for different contagious diseases. Some diseases spread faster than others. Diseases that spread faster need more people to be vaccinated to create this protective barrier. | Les différentes maladies contagieuses ne se propagent pas toutes à la même vitesse. Pour les maladies qui se propagent plus vite, la communauté aura besoin de plus de personnes vaccinées pour créer cette barrière de protection. |
| 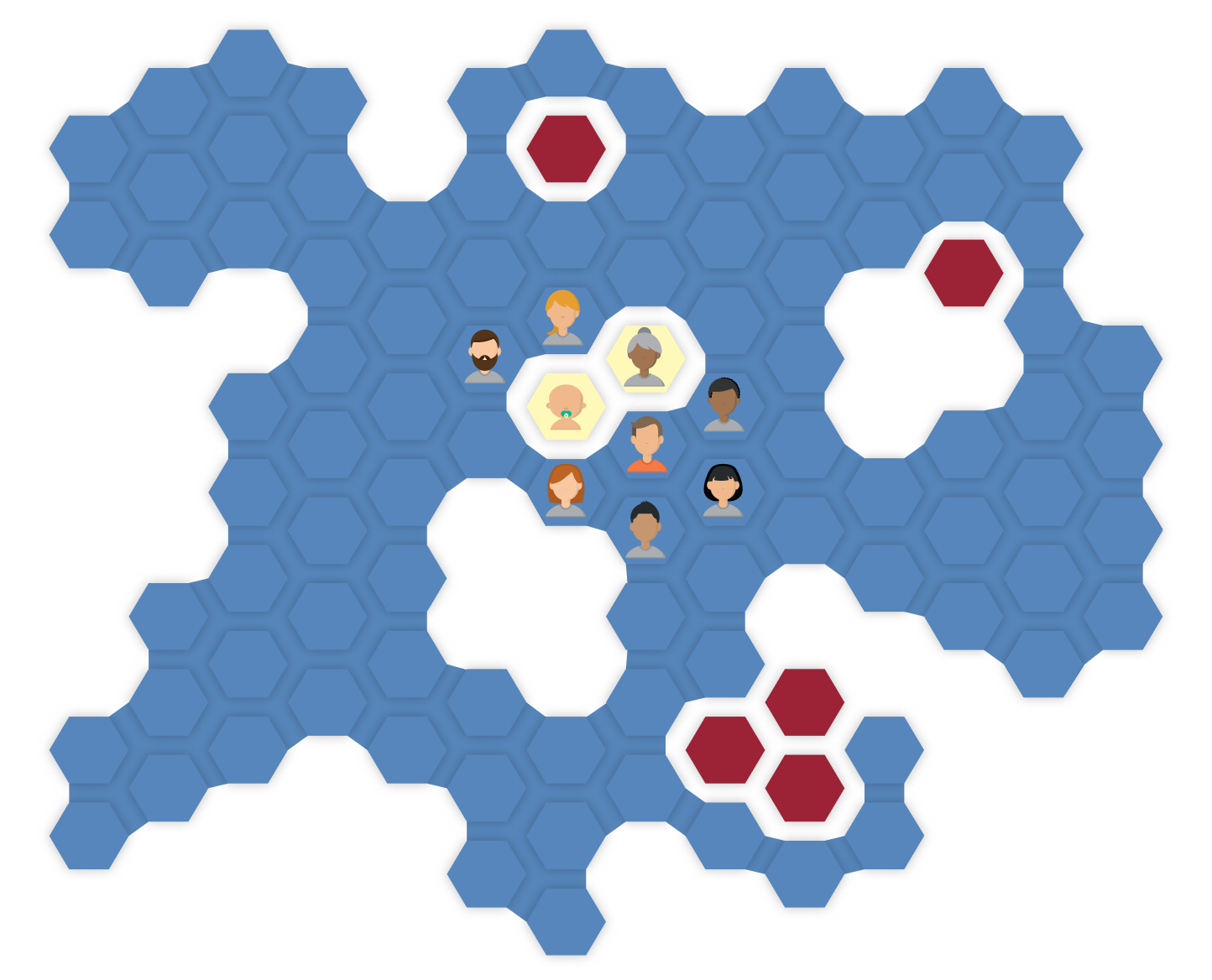 | This barrier is known as herd immunity or community immunity. Community immunity prevents contagious diseases from spreading from one person to another. | Cette barrière se nomme “Immunité grégaire” ou “Immunité collective”. L’immunité collective empêche la maladie contagieuse de se propager d’une personne à l'autre. |
| 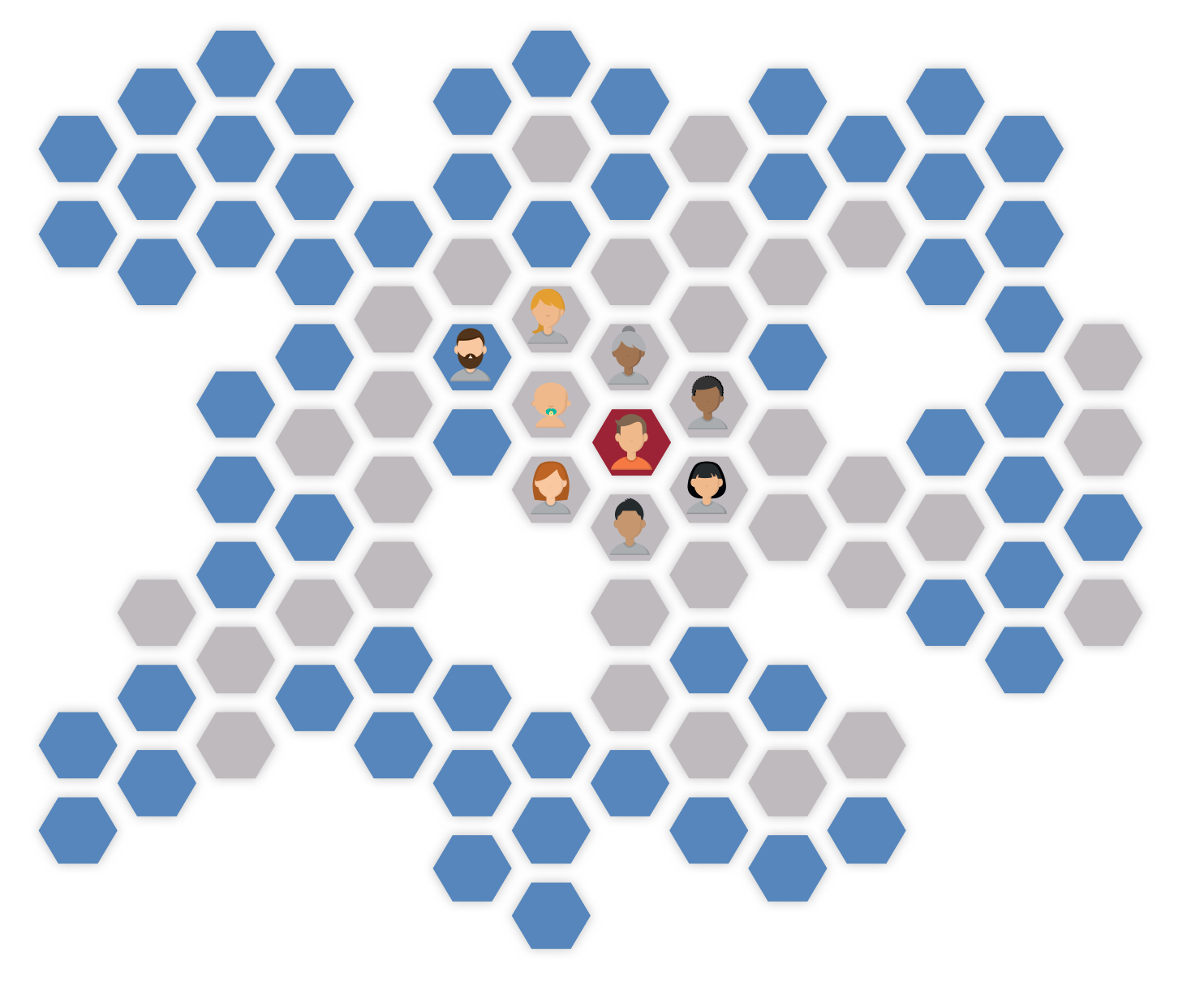 | But if **not** enough people are vaccinated, community immunity cannot exist and your community will not be protected. | Mais lorsque trop peu de gens sont vaccinés... |
| 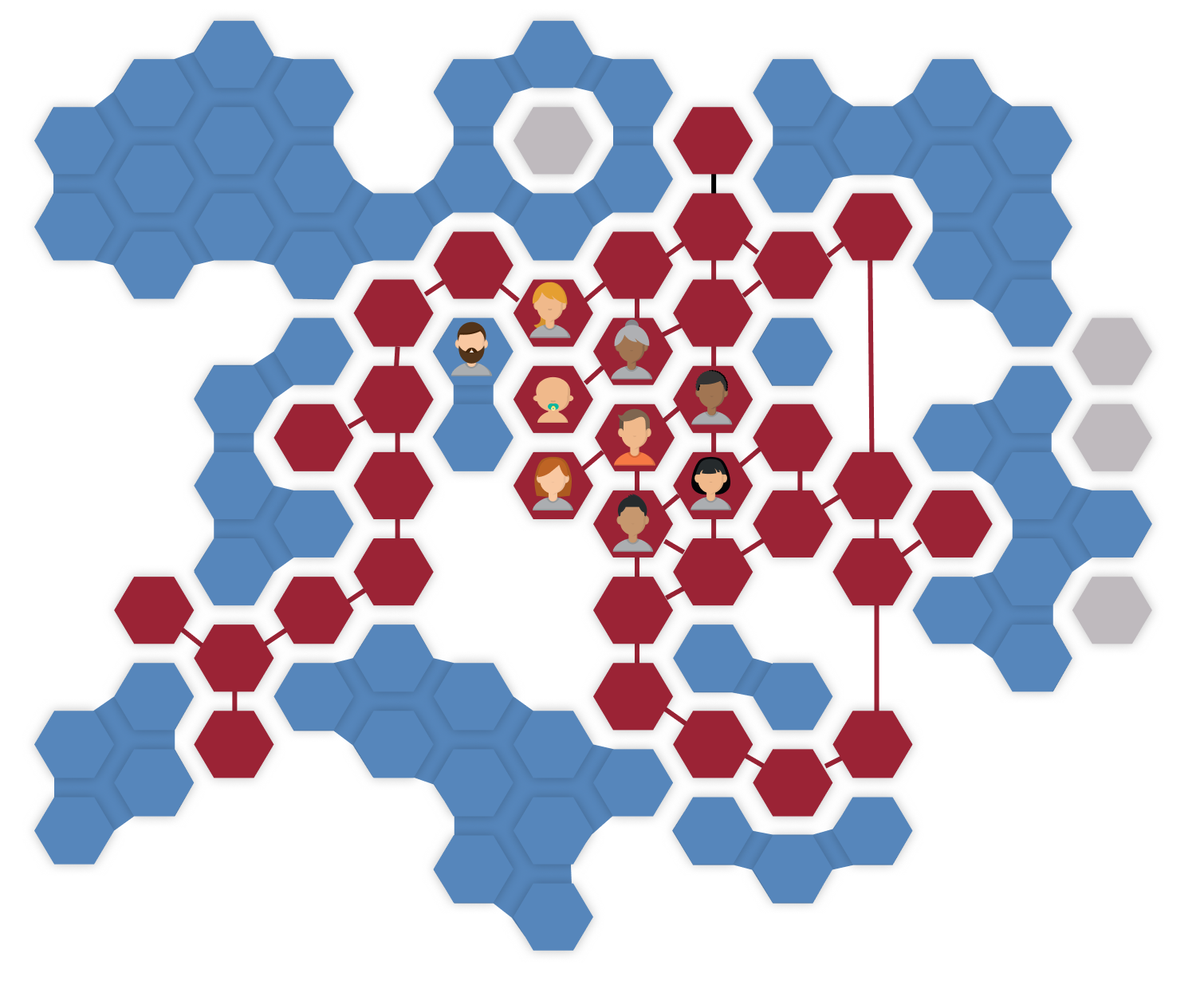 | Then, diseases can easily spread and reach you, the people around you, and others in your community, including vulnerable people. | l’immunité collective n’existe pas et la maladie peut facilement vous atteindre, atteindre les gens autour de vous et les autres personnes de votre communauté, incluant les personnes vulnérables. |
| 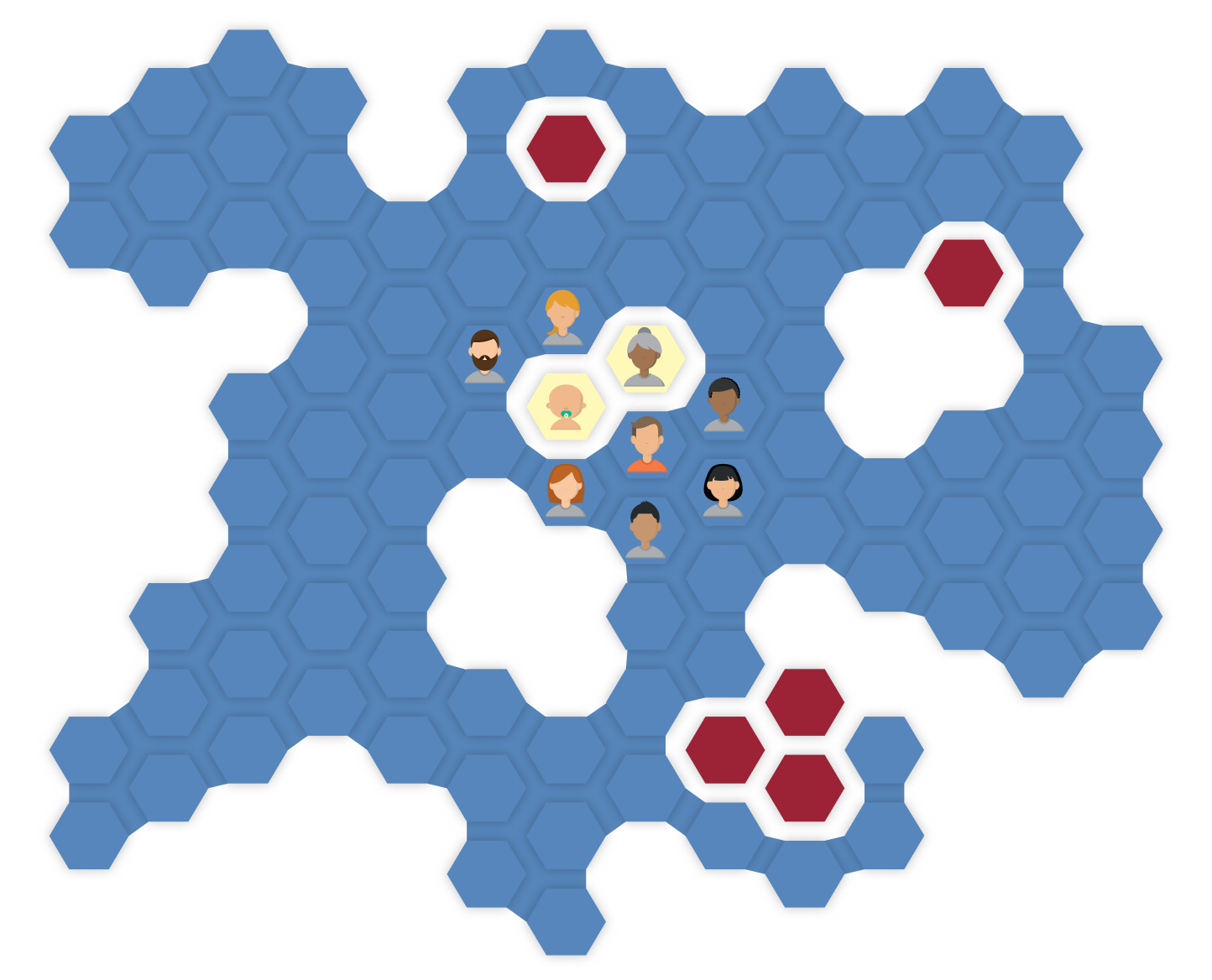 | Whenever your community has community immunity, everyone is protected. | Lorsque l’immunité collective est atteinte dans votre communauté, tout le monde est protégé... |
| 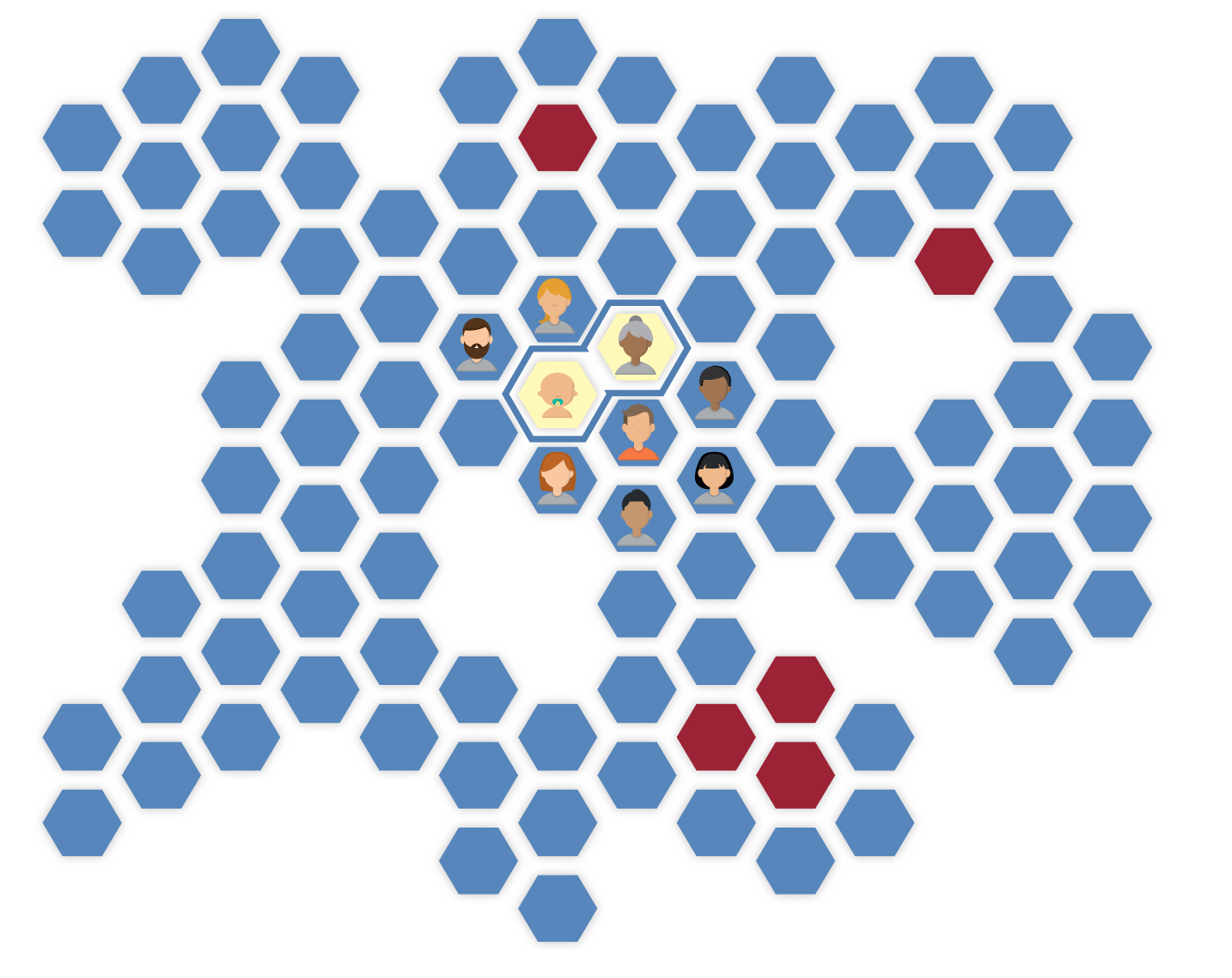 | ….including vulnerable people who cannot be effectively vaccinated.  (Blue color could start fading for some ppl in transition to the last slide) | Même les personnes vulnérables qui ne peuvent pas être vaccinées efficacement.  (La couleur bleue pourrait commencer à pâlir pour certains ppl en transition vers la dernière diapositive) |
| Conclusion | | |
| 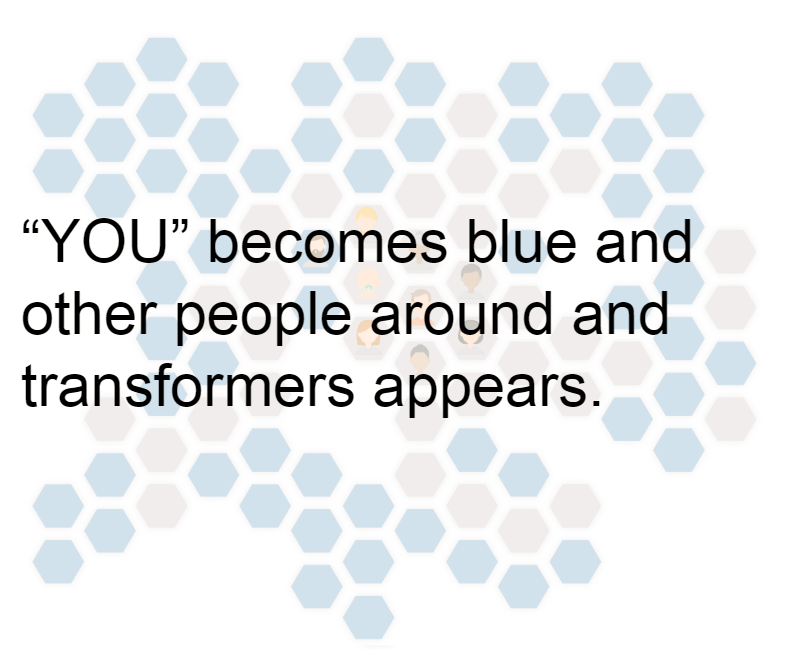 | This means that your decision to get vaccinated or not has an impact on other people in your community. | Cela signifie que votre décision de vous faire vacciner ou non a un impact sur les gens autour de vous. |
