# Supplementary material for: A Web Application About Herd Immunity Using Personalized Avatars: Development Study
Source: J Med Internet Res. 2020 Oct 30;22(10):e20113. doi: 10.2196/20113 (PMC7665952; doi:10.2196/20113)
Supplement: Multimedia Appendix 5 [file jmir_v22i10e20113_app5.docx]

**Appendix 5: Script for cycle 4 (Final cycle)**

| **Visuel** | **English narration** | **Narration française** |
| --- | --- | --- |
| [Introduction]  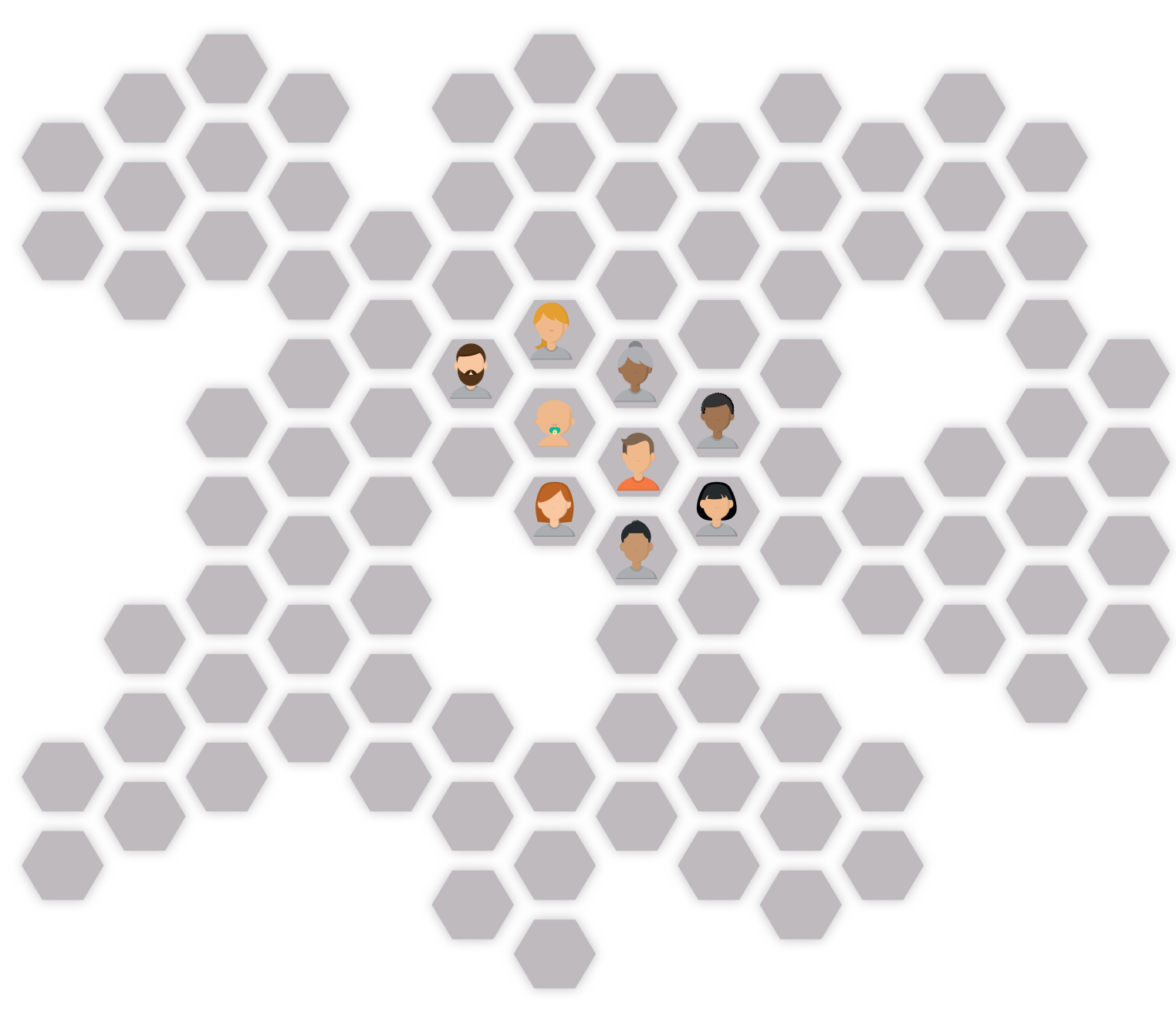 | What role does each of us play in protecting our community? | Quel est le rôle de chacun dans la protection de la communauté? |
| Community | | |
| 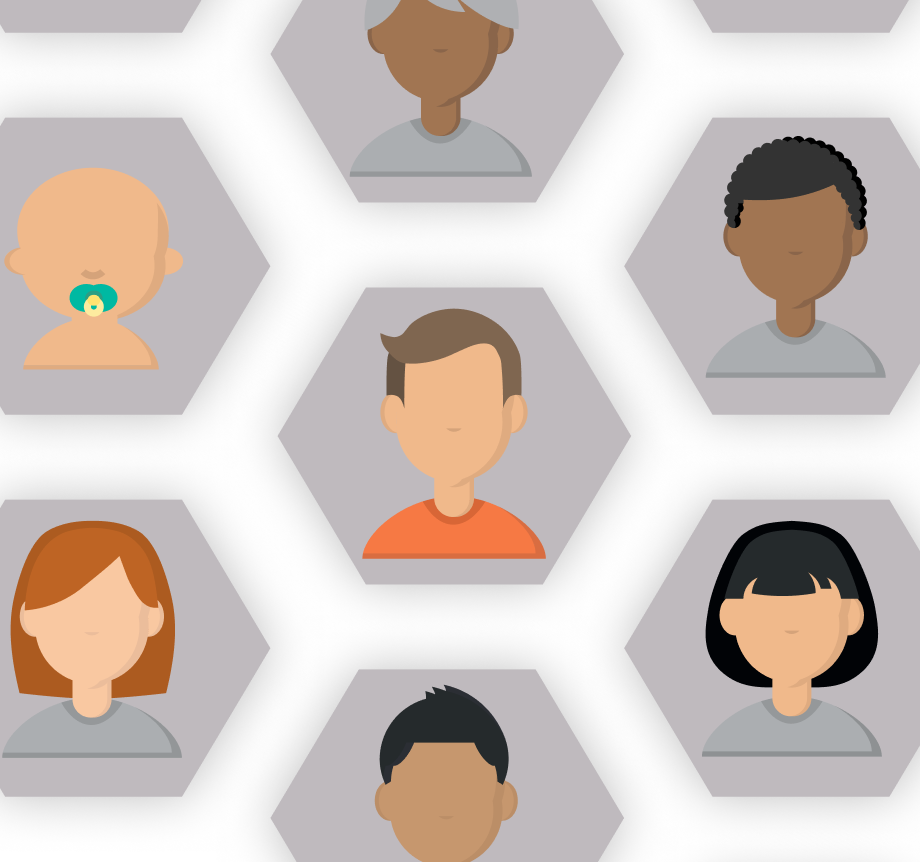 | Imagine this is you. | Imaginez que c’est vous |
| 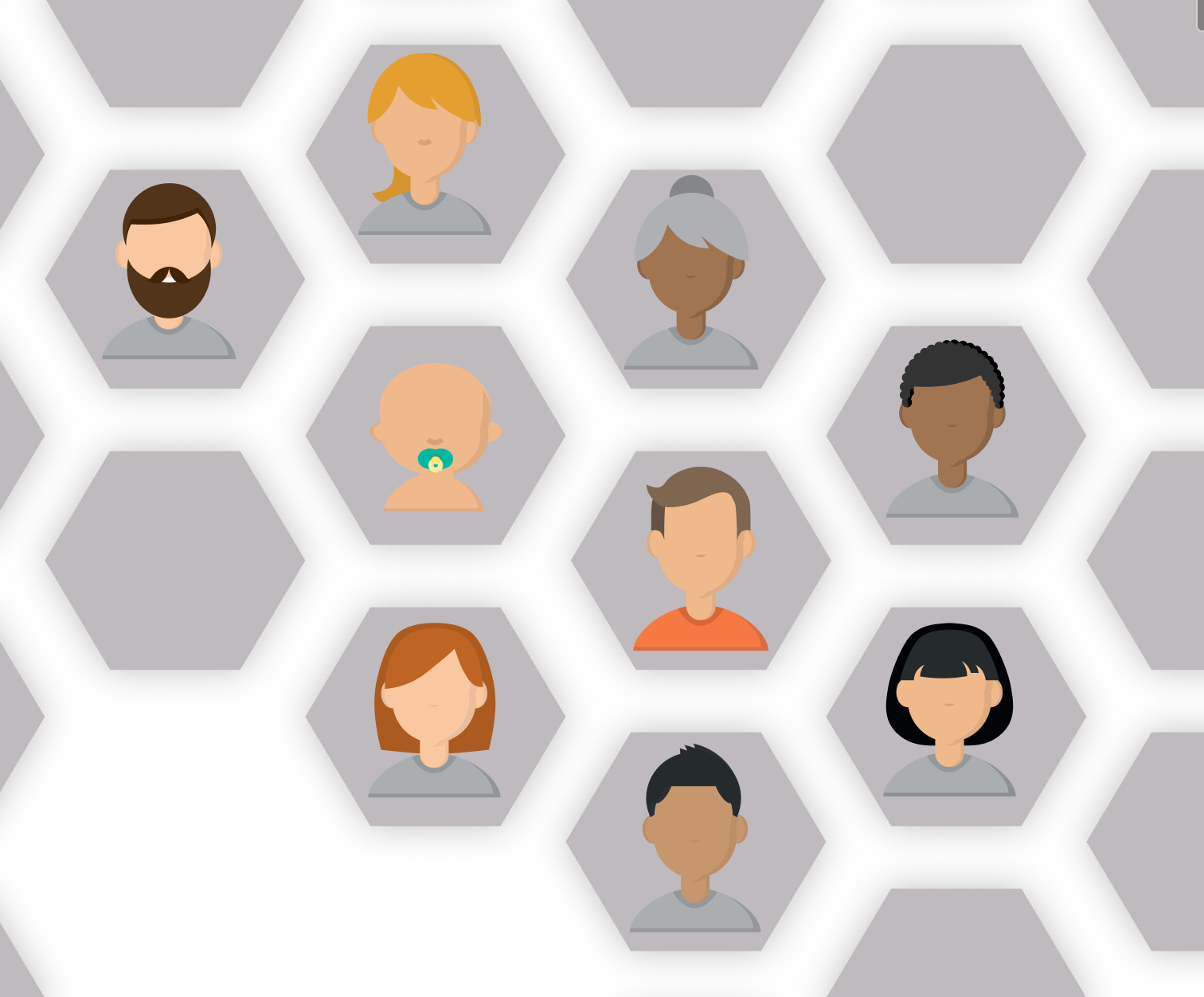 | These are people around you--people you see often, like your family or your coworkers, and the people around *them* that *they* see often. | Voici des personnes que vous côtoyez souvent, comme votre famille ou vos collègues, puis les personnes qu’elles-mêmes côtoient souvent |
| 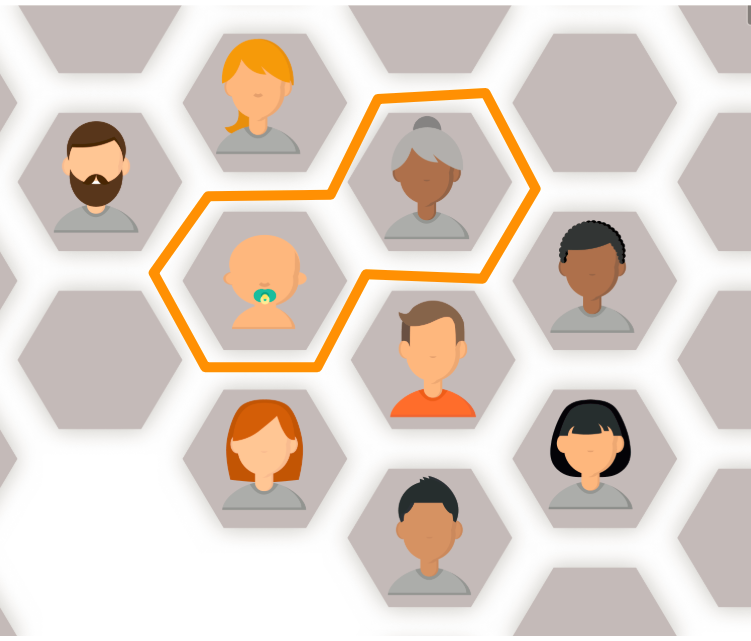 | Some of these people, like babies, older people, or those with fragile immune systems (for example, cancer patients) ... | Il y a des personnes dans ce groupe, par exemple des bébés, des personnes âgées ou des personnes avec un système immunitaire affaibli, comme les patients atteints du cancer... |
| 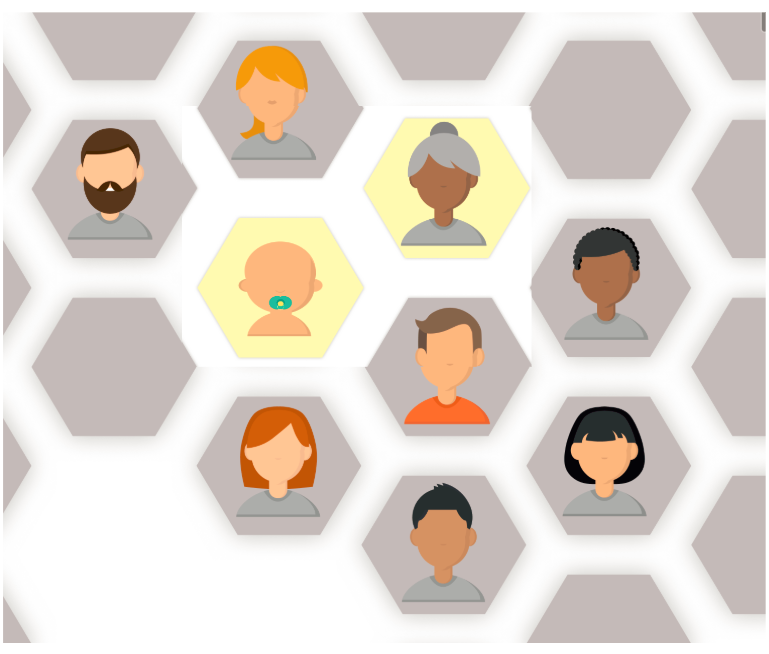 | … are more vulnerable to contagious diseases, either because they can catch them more easily, or the diseases can make them sicker. | ...Ces personnes sont considérées comme étant plus vulnérables, car elles peuvent attraper les maladies plus facilement, et les maladies peuvent les rendre plus malades |
| Infection/Disease | | |
| 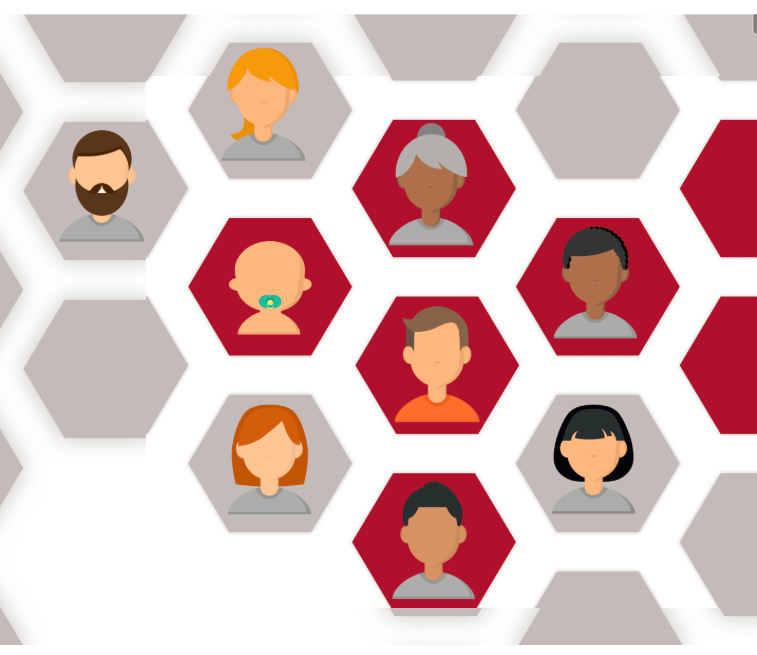 | When a contagious disease enters your community, people can catch it, pass it on to those around them, get sick, or even die from it, especially vulnerable people. | Lorsqu’une maladie contagieuse se propage dans un groupe, les personnes peuvent l’attraper, la transmettre, tomber malade ou même mourir, surtout les personnes plus vulnérables. |
| Vaccines | | |
| 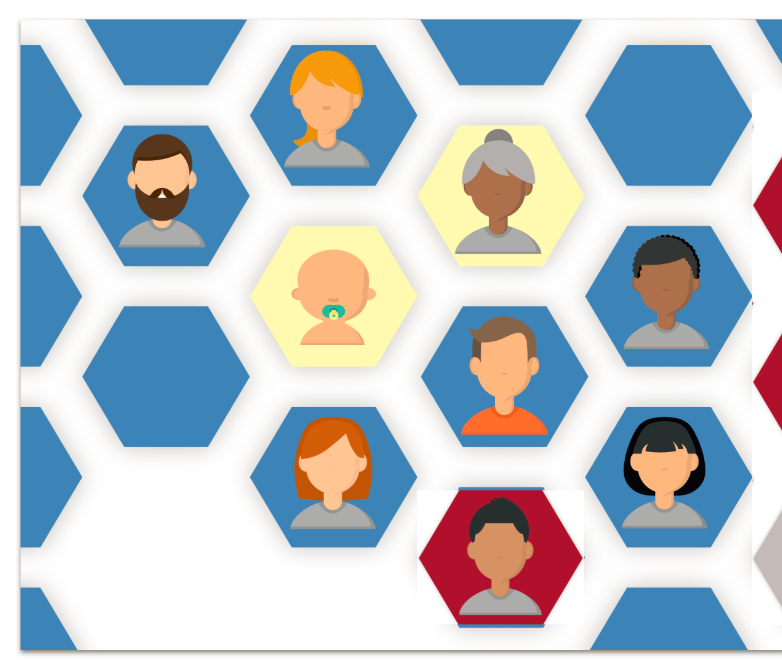 | For some contagious diseases, we have vaccines to protect us. Vaccines train our immune system--the body’s protection system--to fight against diseases. People who are vaccinated are less likely to catch and pass on those diseases. | Des vaccins existent pour nous protéger de certaines maladies contagieuses. Ces vaccins entraînent le corps à lutter contre les maladies. Les personnes vaccinées sont moins susceptibles d’attraper et de transmettre la maladie. |
| 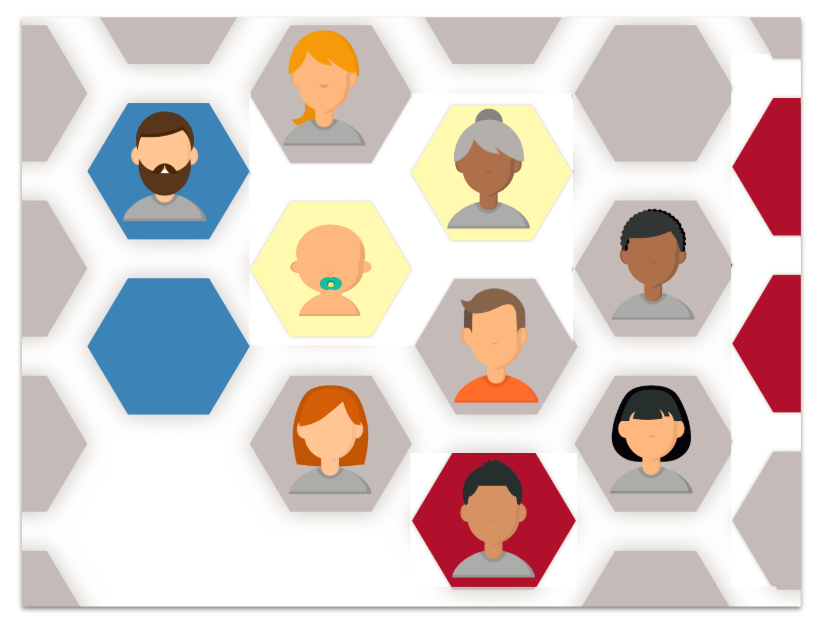 | But vaccines are not perfect. They don’t work every time. And their protection can fade over time... | Mais les vaccins ne fonctionnent pas toujours parfaitement. Ils ne fonctionnent pas à tout coup, et leur efficacité peut diminuer avec le temps |
| 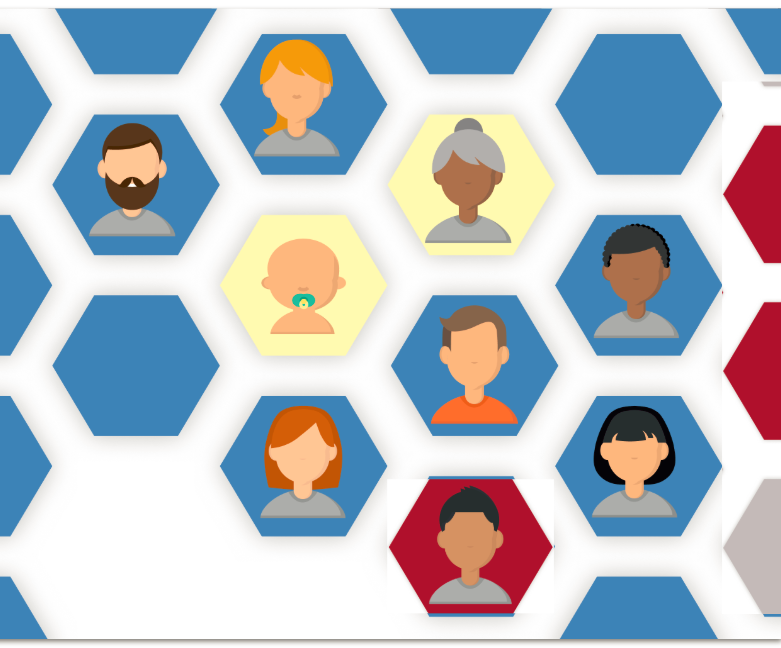 | ...making additional doses of vaccine necessary to ensure protection. | c'est pourquoi d'autres doses de vaccins sont parfois nécessaires pour assurer la protection. |
| Community Immunity | | |
| 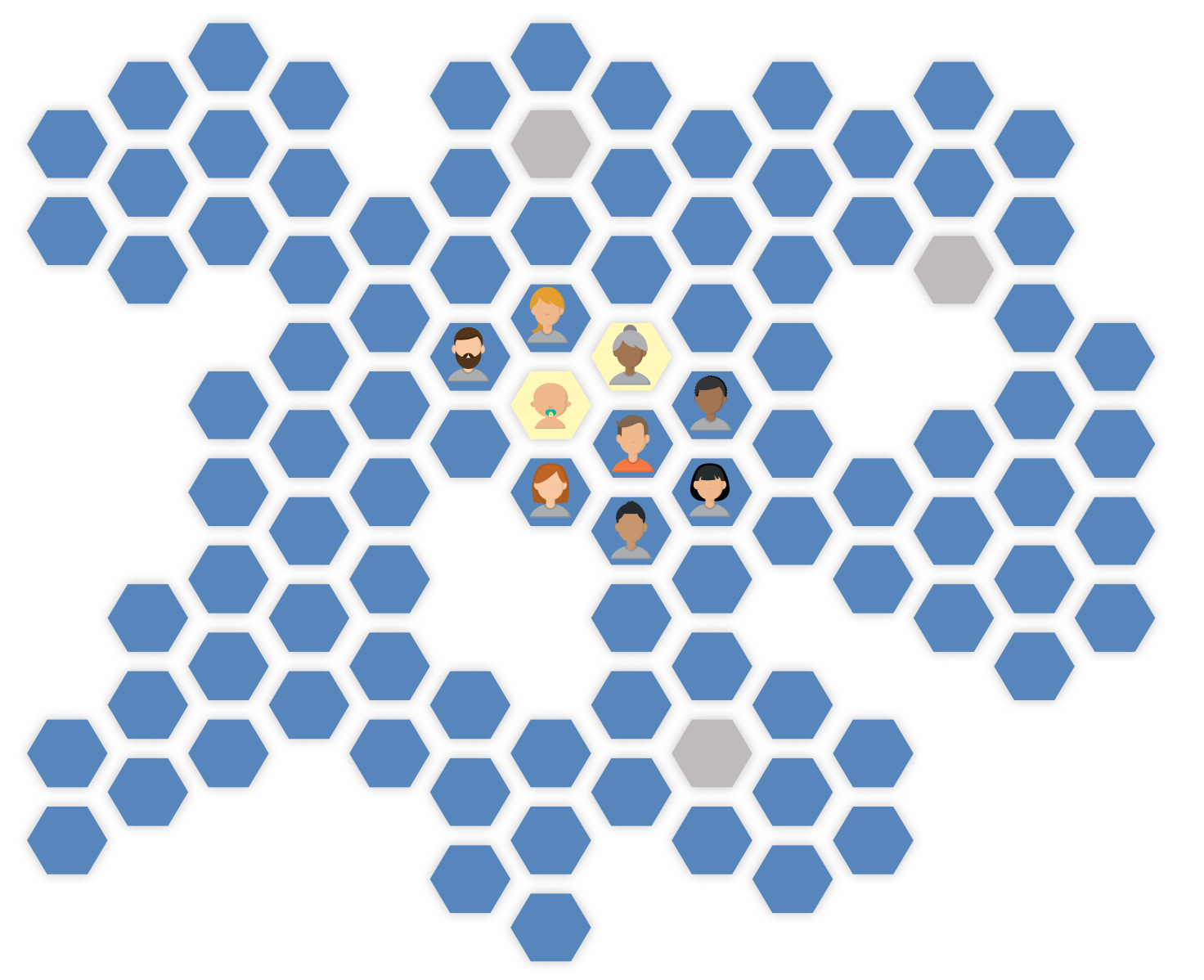 | When you and enough people around you are vaccinated, this creates a protective barrier for your community. | Quand vous êtes vacciné et qu’assez de personnes autour de vous sont vaccinées, cela crée une barrière protectrice autour de toute la communauté. |
| 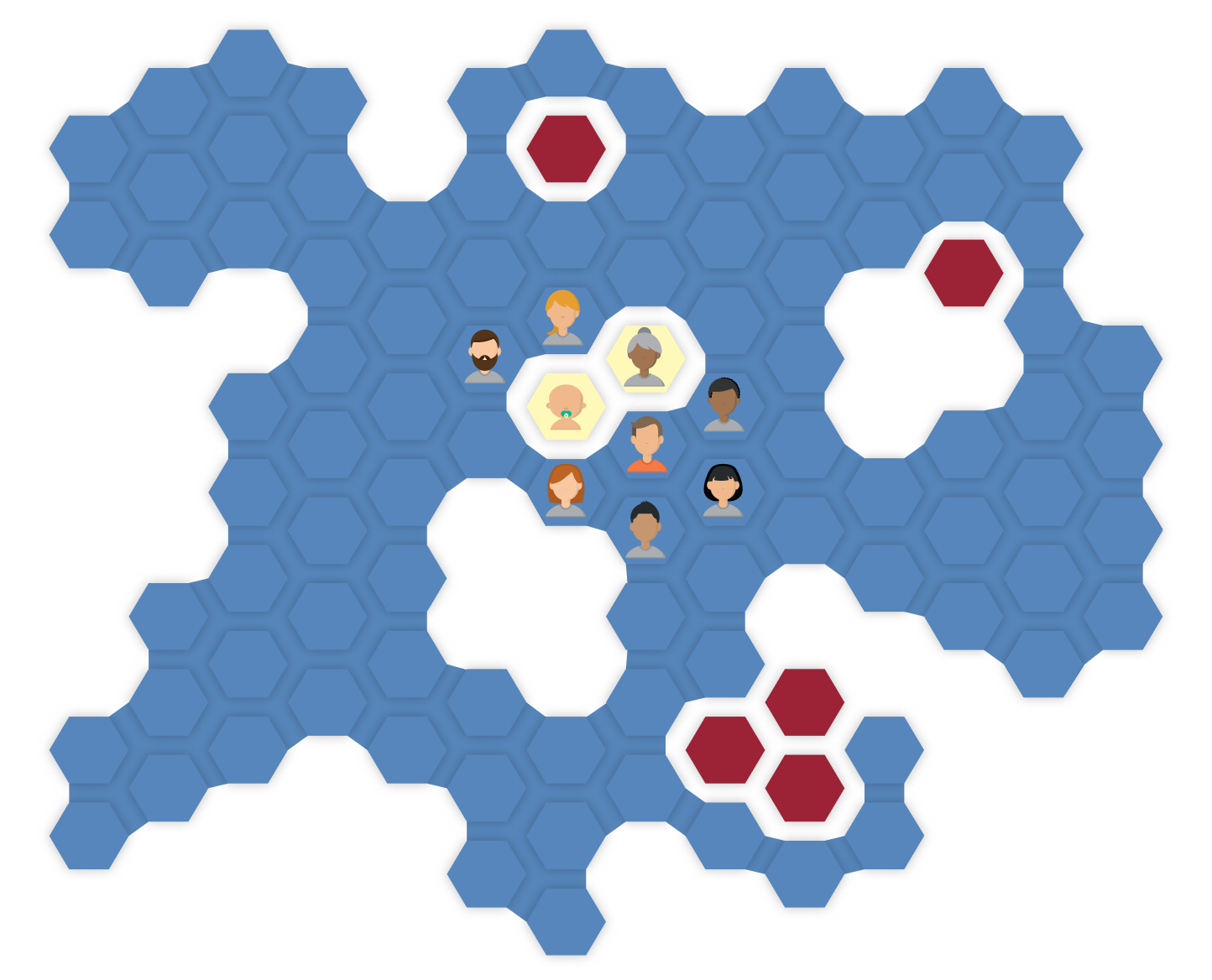 | This barrier is known as “herd immunity” or “community immunity”. Community immunity helps prevent contagious diseases from spreading from one person to another. | Cette barrière se nomme “l’immunité de groupe” ou “l’immunité collective”. L’immunité collective empêche la maladie contagieuse de se propager d’une personne à l'autre. |
| 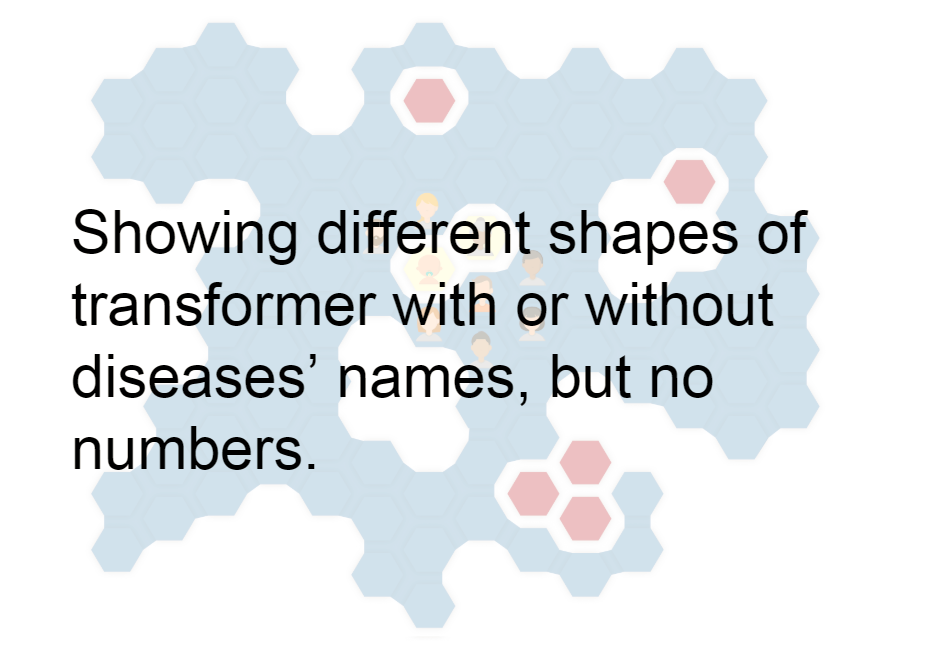 | Some diseases spread more easily than others and need more people to be vaccinated to create community immunity. | Certaines maladies se répandent plus facilement que d'autres. C’est pourquoi plus de personnes doivent être vaccinées contre ces maladies pour créer l'immunité collective |
| 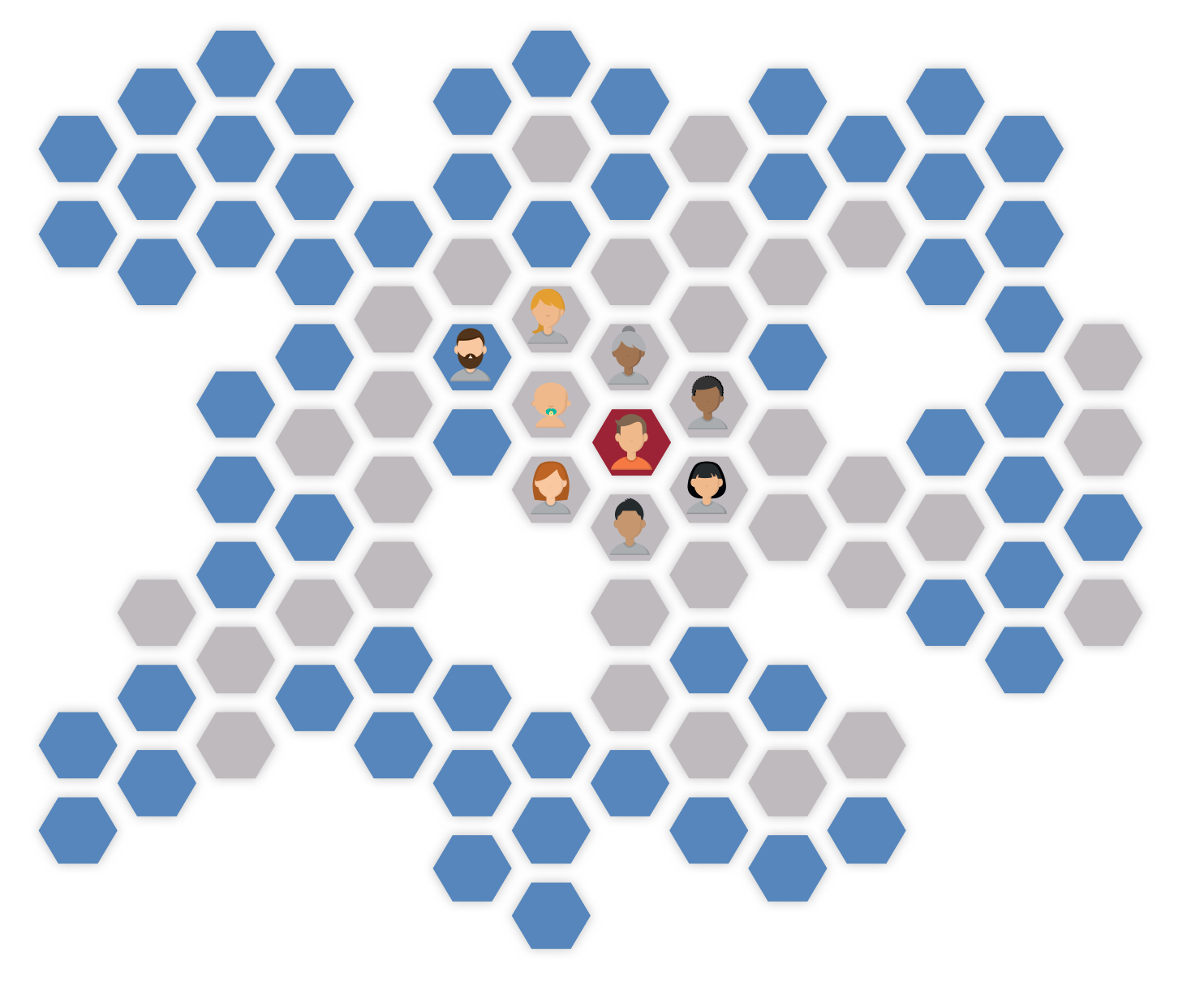 | If **not** enough people are vaccinated, your community will not be protected. | Mais lorsque trop peu des personne sont vaccinés... |
| 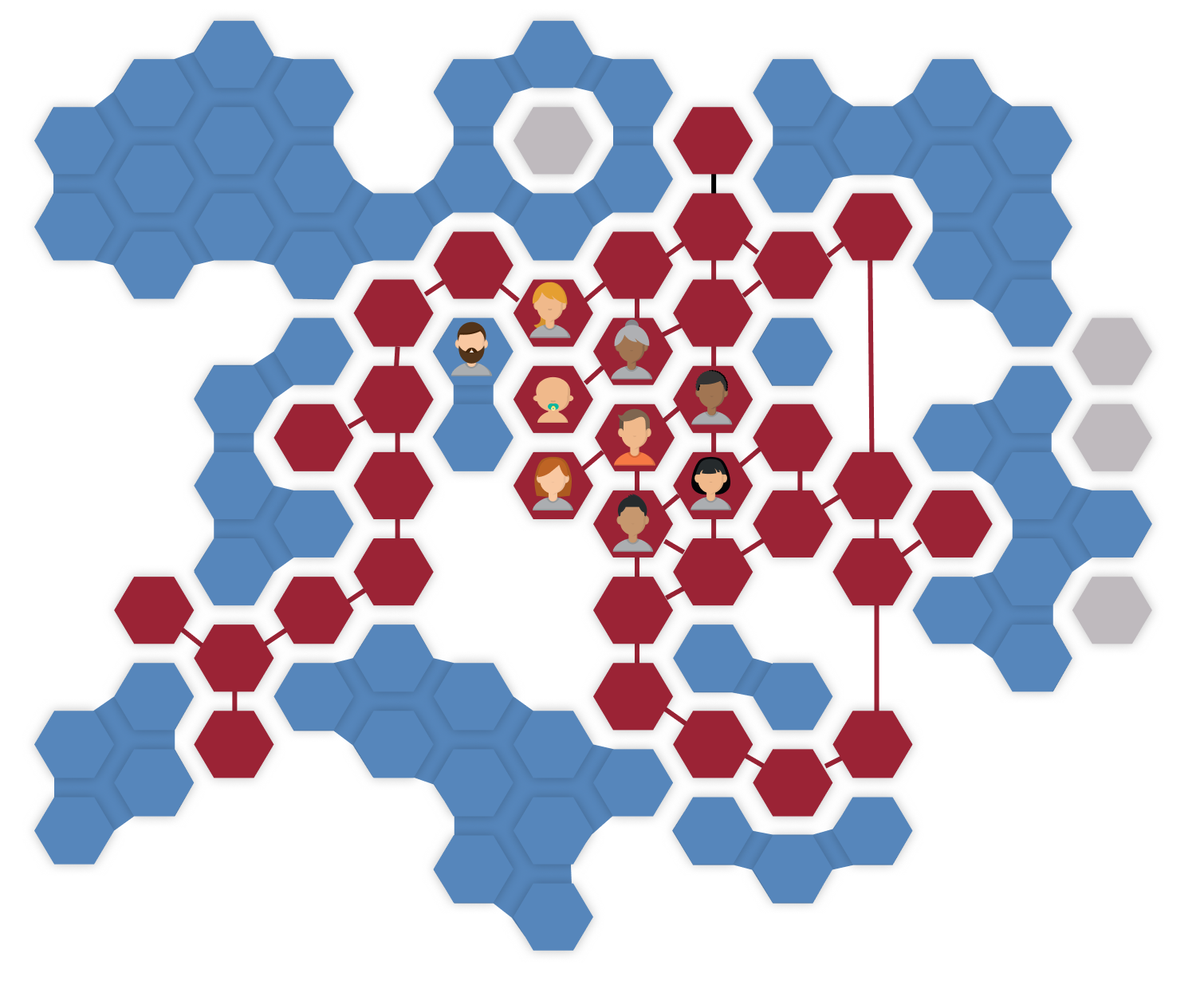 | Then, diseases can more easily spread and reach you, the people around you, and others in your community, including vulnerable people. | l’immunité collective n’existe pas, et la maladie peut facilement vous toucher, , toucher les gens autour de vous et d’autres personnes de votre communauté, incluant les personnes plus vulnérables qui peuvent devenir très malades. |
| 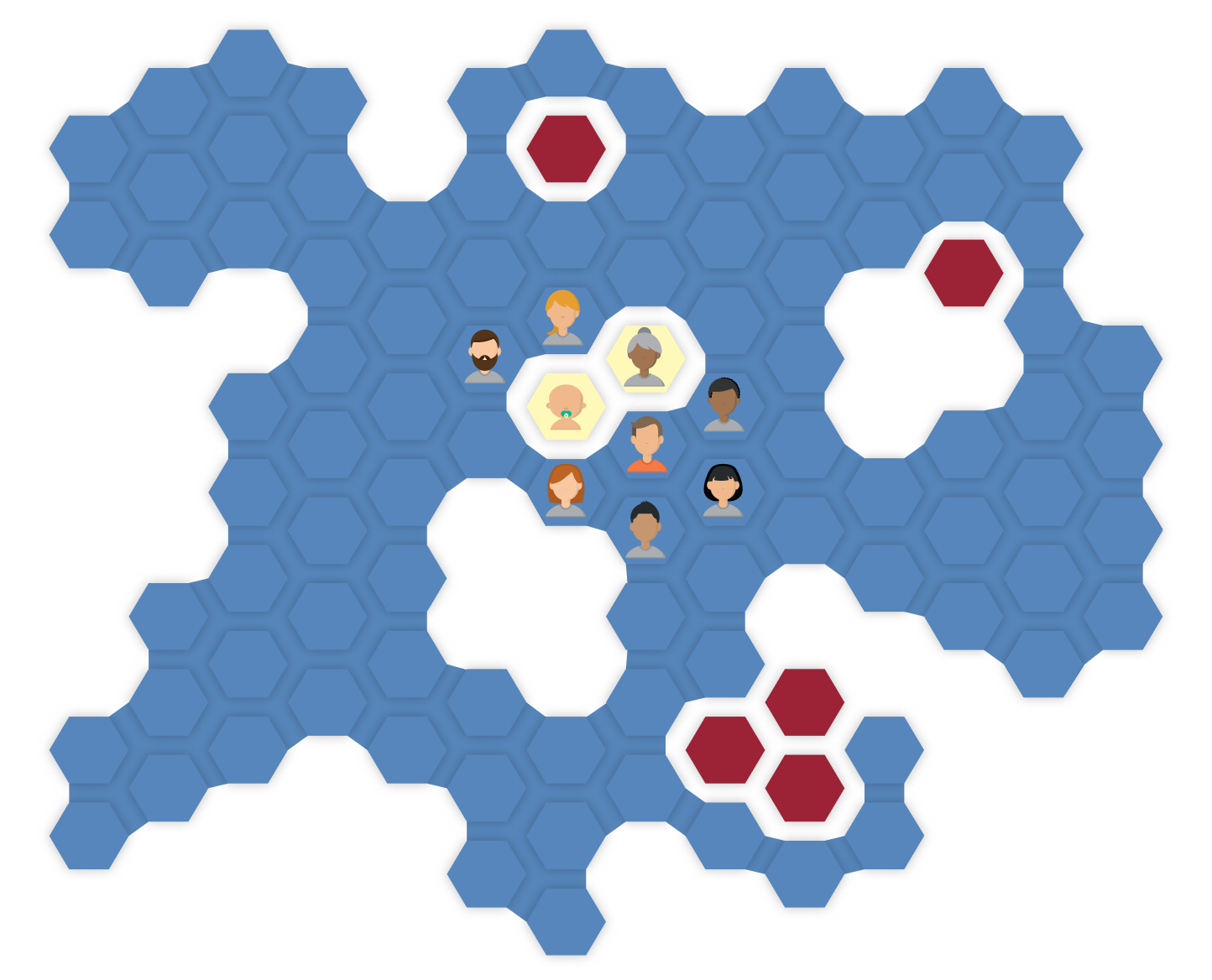 | When your community has community immunity, your community is protected. | Lorsque votre communauté est l’immunité collective, cela protège votre communauté. |
| 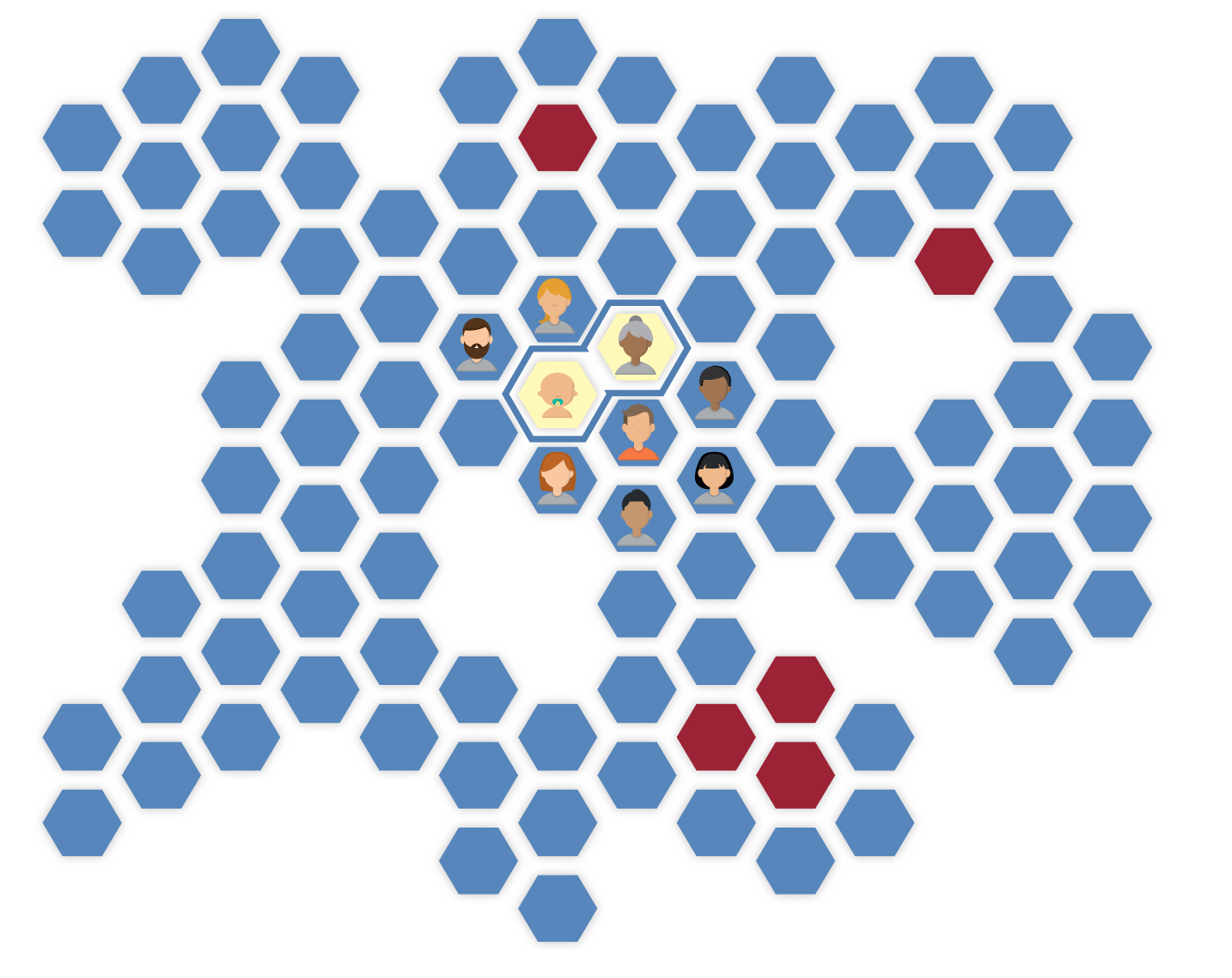 | ….including vulnerable people who depend on those around them for protection.  (Blue color could start fading for some ppl in transition to the last slide) | ...même les personnes plus vulnérables qui ne peuvent pas être vaccinées efficacement.  (La couleur bleue pourrait commencer à pâlir pour certains ppl en transition vers la dernière diapositive) |
| Conclusion | | |
| 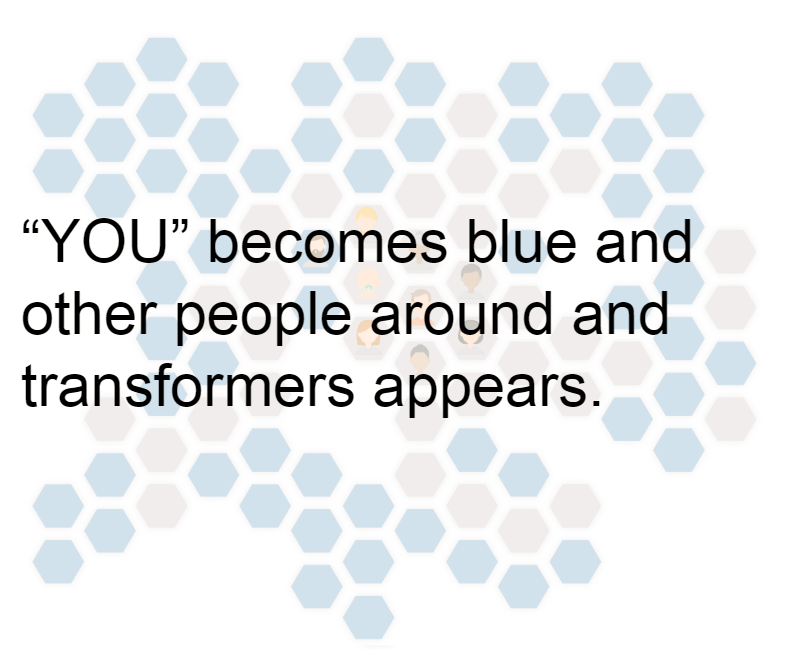 | This means that your decision to get vaccinated or not has an impact not only on you, but also on other people in your community. | Cela signifie que votre décision de vous faire vacciner ou non a un impact sur les gens autour de vous. |
