# Supplementary material for: A Web Application About Herd Immunity Using Personalized Avatars: Development Study
Source: J Med Internet Res. 2020 Oct 30;22(10):e20113. doi: 10.2196/20113 (PMC7665952; doi:10.2196/20113)
Supplement: Multimedia Appendix 9 [file jmir_v22i10e20113_app9.docx]

**Appendix 9: The communication goals set for the second iterative cycle of visualization:**

| **S.No** | **Design element or a concept** | **Message design elements intended to convey in the visualization** | **Our expectations or goals of our design element** (**desired interpretation)** | **What users reported when viewing these design elements (verbal feedback) (n=11)** |
| --- | --- | --- | --- | --- |
| 1. | 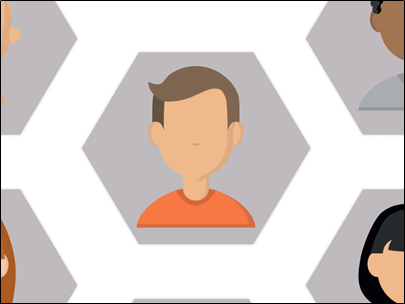 | The avatar represents the participant. | Did participants’ explanations of the visualization include that avatar represents them? | 5/11 participants reported that the avatar represents them. |
| 2. | 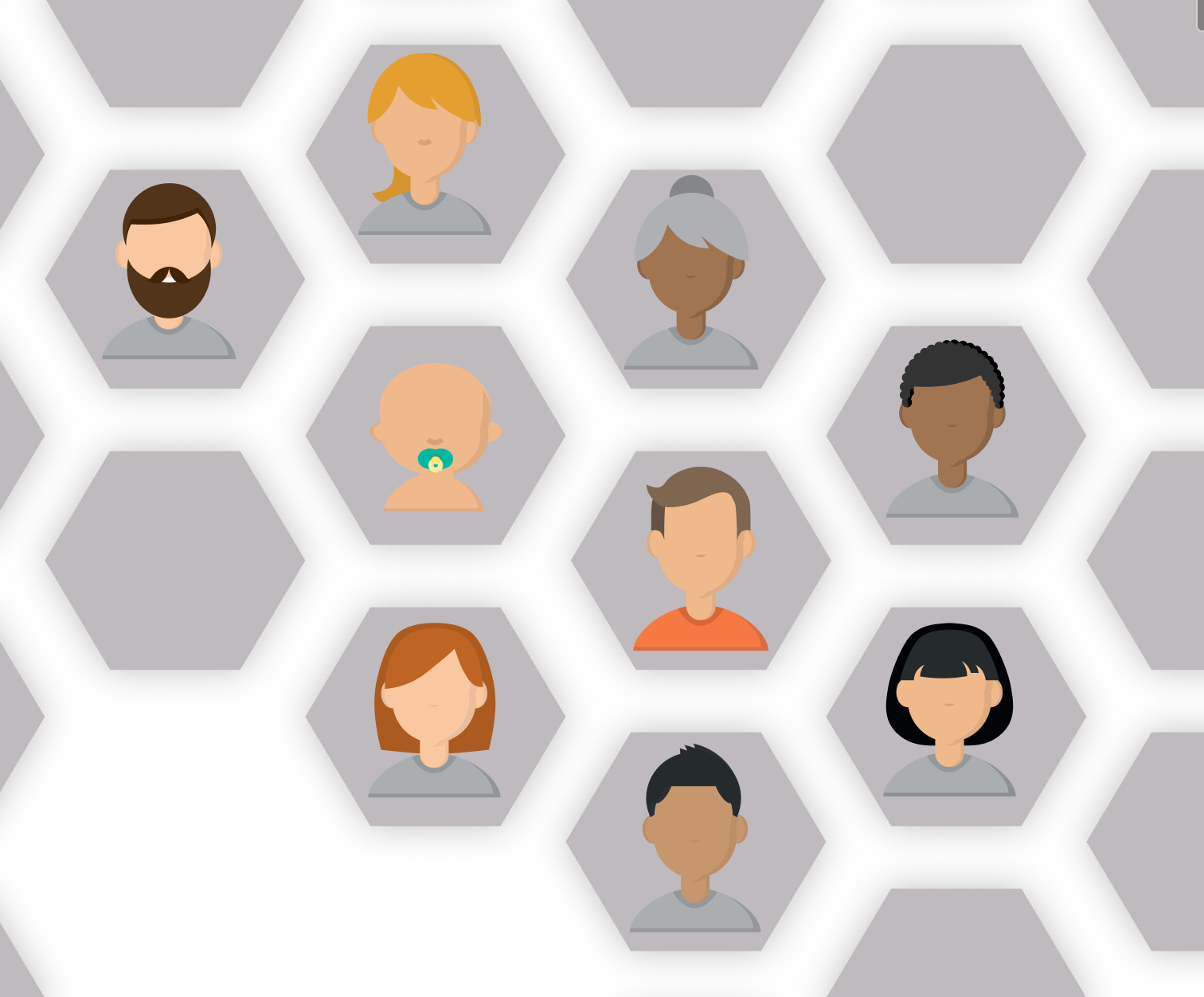 | People in hexagons represent members of the participants’ community and people around them. | Did participants’ explanations of the visualization include that people in hexagons represent members of the participant’s community and people around them? | 10/11 participants reported that the people in hexagons represent a member of their community or people with whom they are in daily contact. |
|  |  |  |  |  |
| 3. | 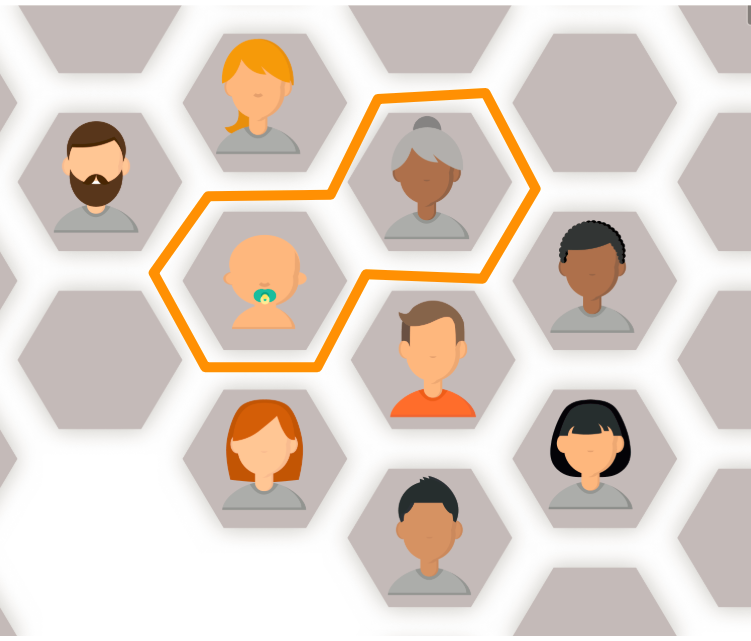 | Icon of an older woman and/or a baby represents vulnerable people or those with fragile immune systems (for example, cancer patients). | Did participants’ explanations of the visualization include that the older woman and the baby represent vulnerable people? | All participants (11/11) reported that the older woman and the baby represent vulnerable people. |
| 4. | 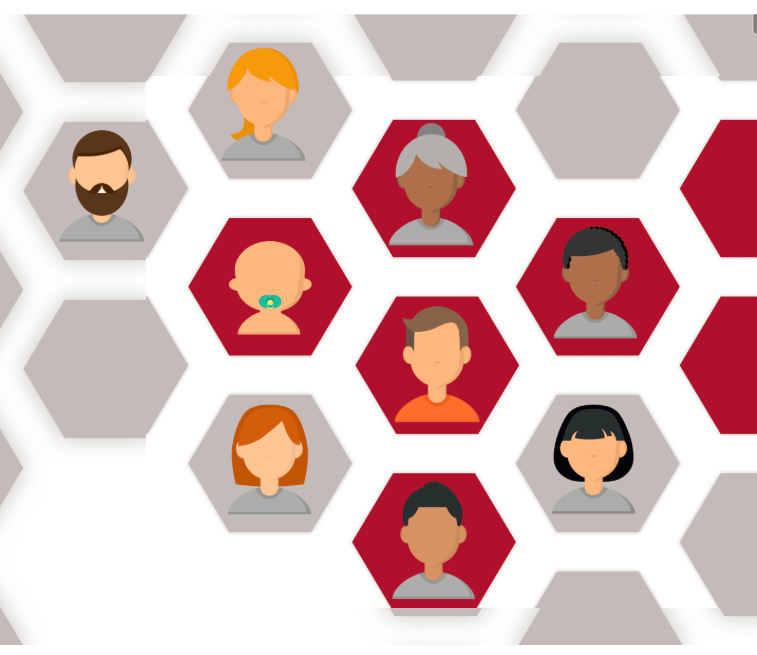 | Red colour spreading to various hexagons indicates the spread of contagious disease. | Did participants’ explanations of the visualization include that the red colour spreading to various hexagons indicates propagation of a contagious disease? | All participants (11/11) reported that red colour shows propagation of a contagious disease. |
| 5. | 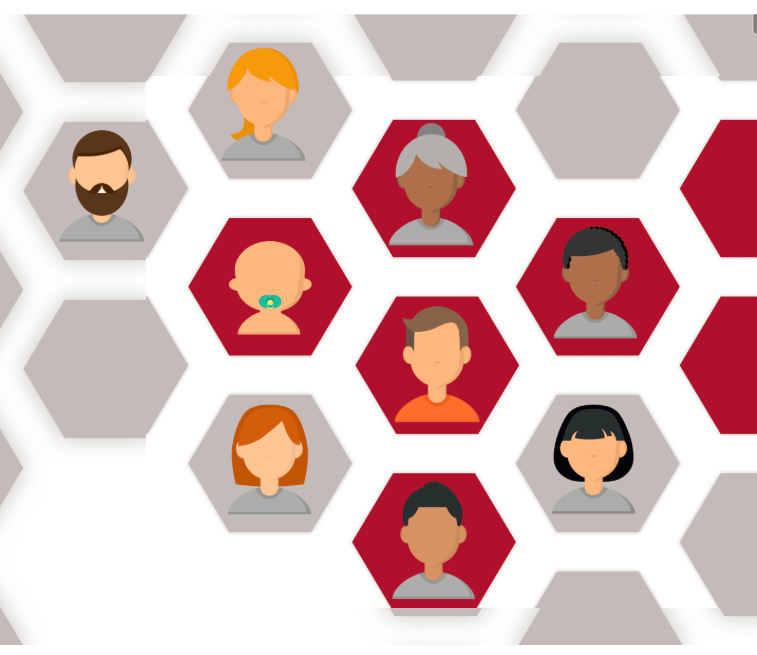 | Vulnerable people getting infection represent that vulnerable people can get very sick or die from a contagious disease. | Did participants’ explanations of the visualization include that the spread of contagious disease makes vulnerable people sick or die? | All participants (11/11) reported that spreading of a contagious disease makes vulnerable people get sick or die. |
| 6. | 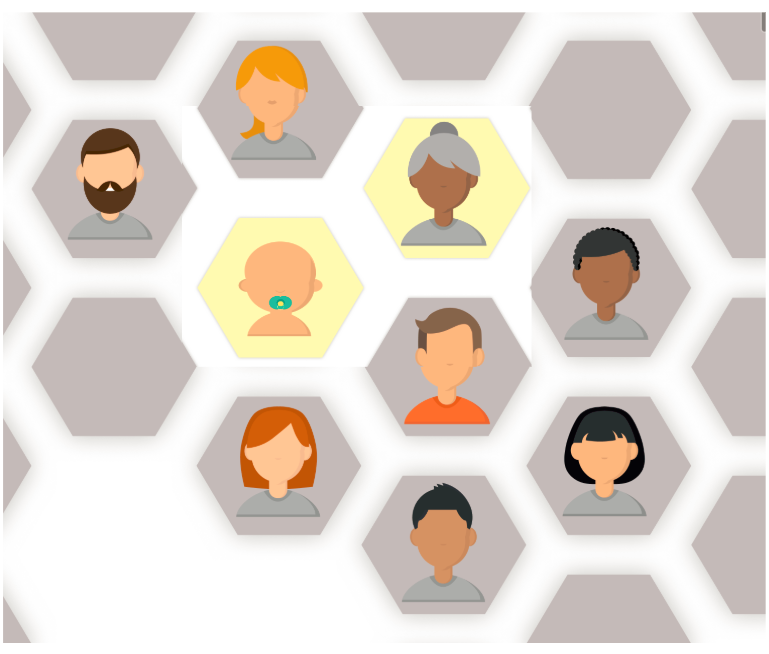 | Yellow colour behind “baby” and “an older woman” represents vulnerable people. | Did participants’ explanations of the visualization include that yellow colour represents vulnerable people? | All participants (11/11) reported that yellow colour represents vulnerable or susceptible people. |
| 7. | 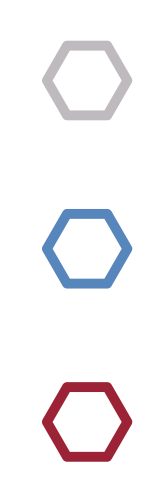 | Red colour signals diseased or infected  Blue colour signals vaccinated/protected  Grey colour signals susceptible to disease or infection | Did participants’ explanations of the visualization include that ; Red colour signals diseased or infected; Blue colour signals vaccinated/protected;Grey colour signals susceptible to disease or infection? | All participants (11/11) reported that red colour indicates disease, infection, or danger.  None of the participants (0/11) mentioned the meaning of grey colour.  2/11 participants reported that blue colour indicates vaccinated or protected. |
| 8. | 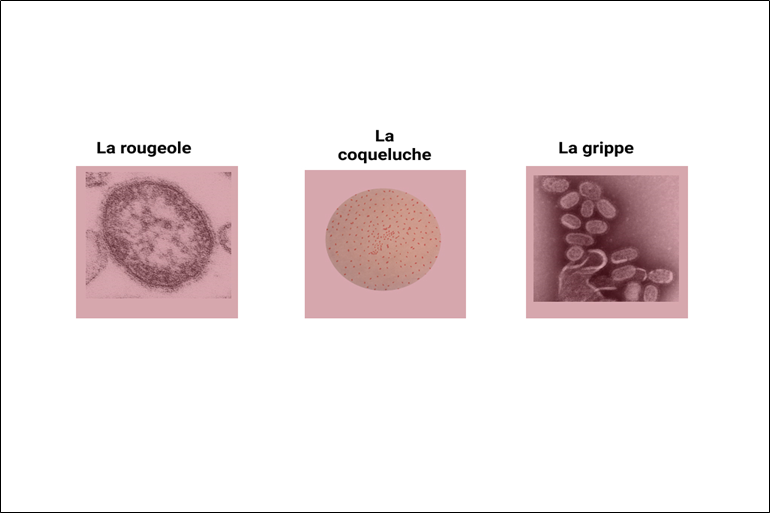 | Different diseases are different from each other (measles, pertussis and influenza as an example). | Did participants’ explanations of the visualization include that different diseases are different from each other? | 3/11 participants reported that diseases are different from each other. 8/11 participants found it confusing. |
| 9. | 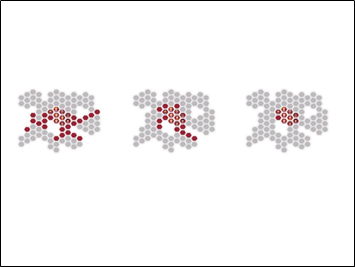 | Different diseases spread at different rates (measles, pertussis and influenza as an example). | Did participants’ explanations of the visualization include that different diseases spread at different rates? | 3/11 participants’ explanation made a link with the previous image that different diseases spread at different rates. 8/11 participants reported that it was confusing for them as there was too much information to retain from the visualization. |
|  | 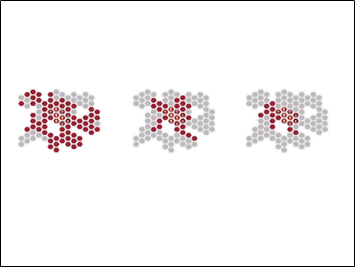 |  |  |  |
|  | 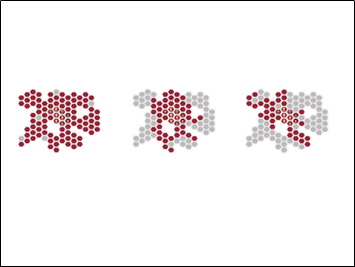 |  |  |  |
|  | 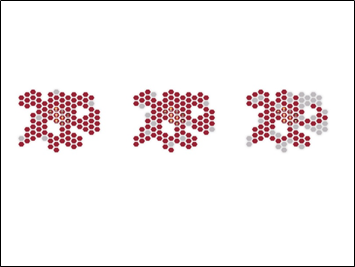 |  |  |  |
| 10. | 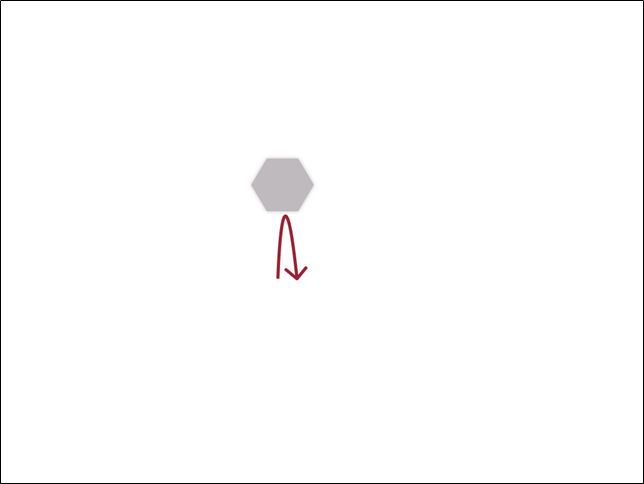 | Red line hitting a hexagon indicates an infection reaching a person. | Did participants’ explanations of the visualization include that the red line reaching a hexagon is an infection? | 5/11 participants reported that the red line reaching a hexagon represents an infection. |
| 11. | 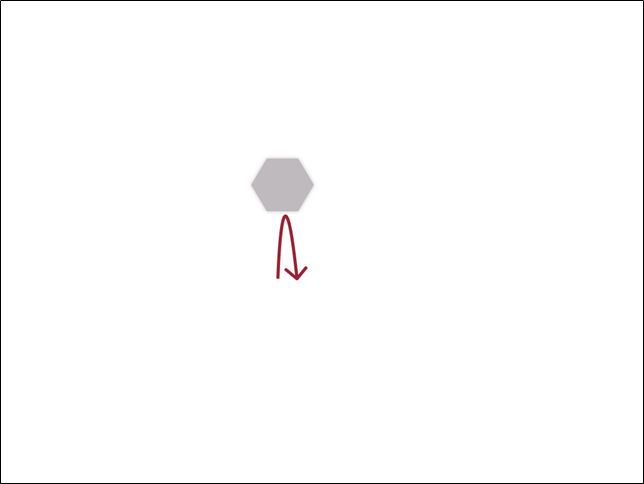 | Red line bouncing back from the hexagon without any change in colour indicates natural immunity. | Did participants’ explanations of the visualization include that the red line bouncing back from the grey hexagon without any change in colour means naturally immune? | None of the participants (0/11) reported that the redline bouncing back from the grey hexagon means natural immunity. |
| 12. | 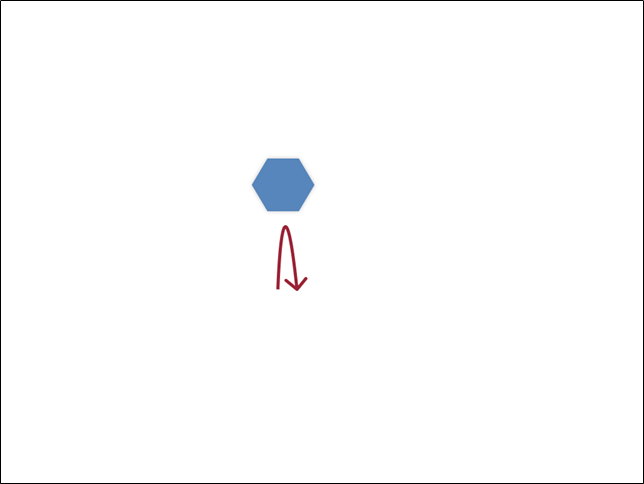 | Red line bouncing back from the hexagon without any change in colour indicates the vaccine-induced immunity. | Did participants’  explanations of the visualization include that the red line bouncing back from the blue hexagon without any change in colour means vaccine induced immunity? | 2/11 participants reported that the red line bouncing back from the blue hexagon means vaccine induced immunity. 9/11 participants did not understand and found it confusing. |
| 13. | 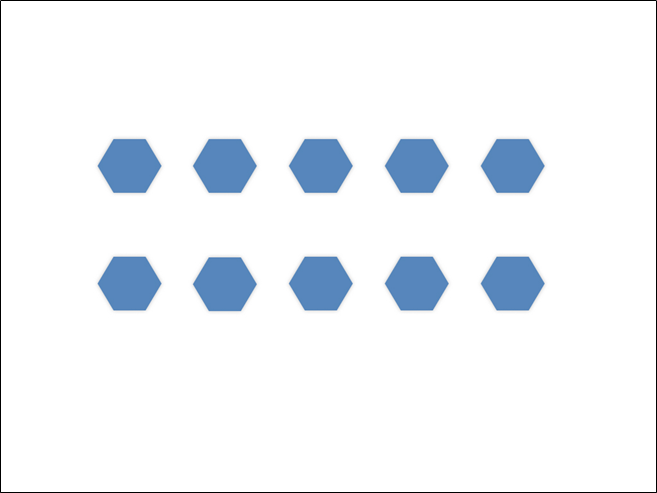 | 10 hexagons indicate individuals or people. | Did participants’ explanations of the visualization include those 10 hexagons as individuals or people? | All participants (11/11) reported that the 10 hexagons represent 10 people. |
| 14. | 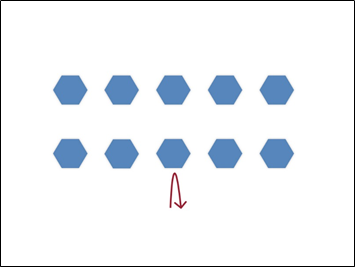 | Vaccine protects people from infection (9/10 hexagons remain blue in colour). | Did participants’ explanations of the visualization include that blue hexagon represents vaccinated people who are protected? | 6/11 participants reported that the blue hexagon represents vaccinated people who are protected. |
| 15. | 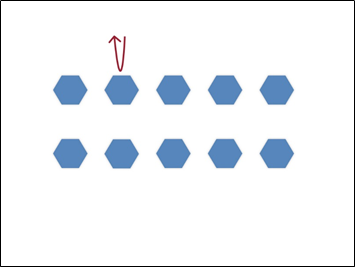 | Person getting infected despite vaccination (when 1 blue hexagon changes to red after infection). | Did participants’ explanations of the visualization include that vaccinated people can get an infection? | All participants (11/11) reported that vaccinated people could catch an infection. |
|  | 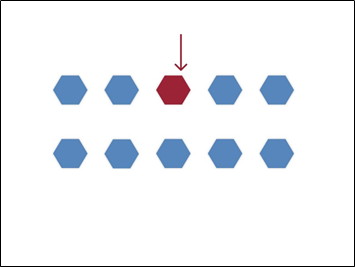 |  |  |  |
| 20. | 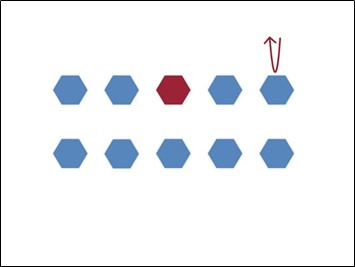 |  |  |  |
| 16. | 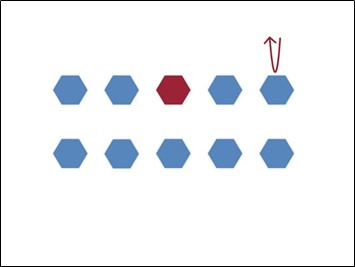 | Vaccines are not always perfect (when 1 blue hexagon changes to red after infection). | Did participants’ explanations of the visualization include that vaccines are not always perfect? | 7/11 participants reported that vaccines are not always perfect. |
| 17. | 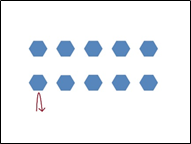 | Some vaccines provide less protection than others (when 3 blue hexagons change to red after infection). | Did participants’ explanations of the visualization include that some vaccines provide less protection than others? | 3/11 participants reported that some vaccines are less effective than others and provide less protection. |
|  | 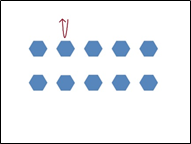 |  |  |  |
|  | 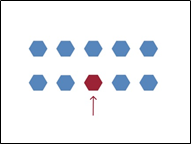 |  |  |  |
|  | 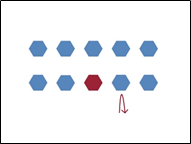 |  |  |  |
|  | 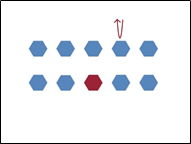 |  |  |  |
| . | 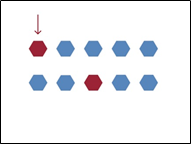 |  |  |  |
|  | 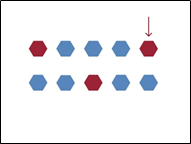 |  |  |  |
|  | 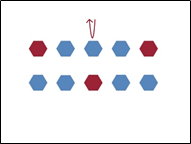 |  |  |  |
|  | 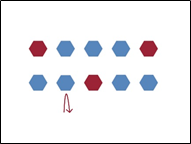 |  |  |  |
|  | 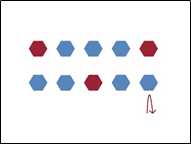 |  |  |  |
| 18. | 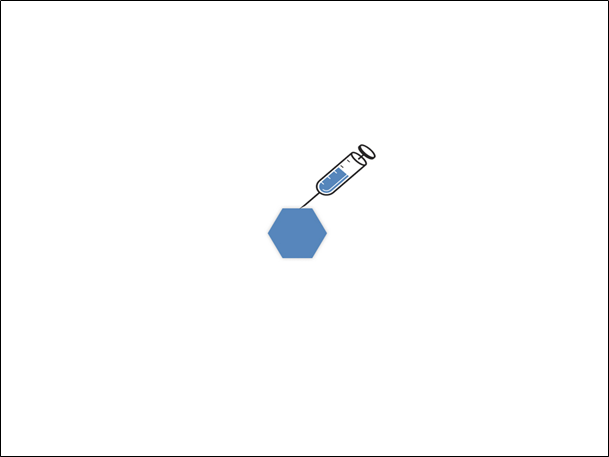 | Some vaccines require a single dose of the vaccine to provide protection. | Did participants’ explanations of the visualization include that some vaccines require a single dose of the vaccine to provide protection? | None of the participants (0/11) reported that some vaccines need a single shot to provide protection. |
| 19. | 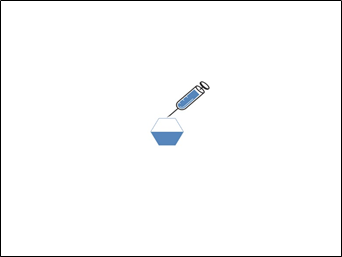 | Some vaccines require multiple doses of vaccines to provide protection. | Did participants’ explanations of the visualization include that some vaccines require multiple doses of vaccines to provide protection? | 10/11 participants reported that some vaccines require multiple doses of vaccines to provide protection. |
|  | 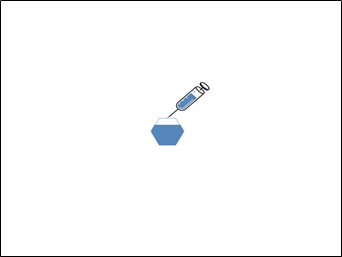 |  |  |  |
|  | 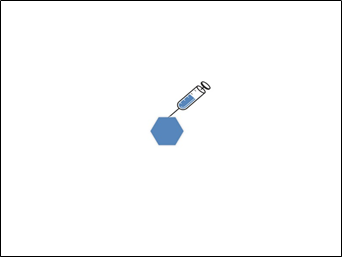 |  |  |  |
| 20. | 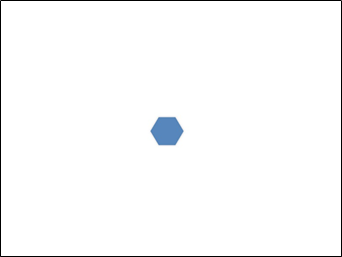 | Some vaccines wane over time and need boosters. | Did participants’ explanations of the visualization include that some vaccines require a booster dose and wane over time? | 6/11 participants reported that some vaccines wane over time and require a booster dose. 5/11 participants found images confusing. |
|  | 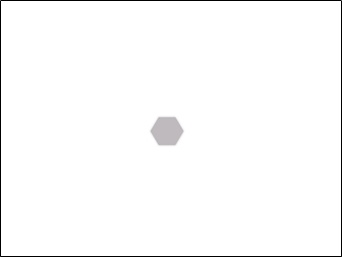 |  |  |  |
|  | 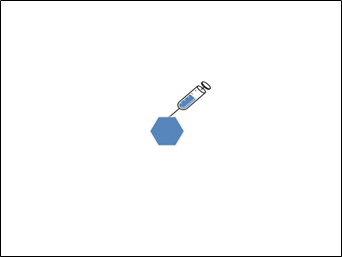 |  |  |  |
| 21. | 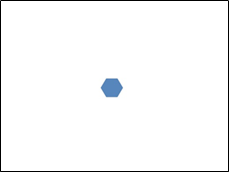 | Some vaccines need a yearly dose of the vaccine. | Did participants’ explanations of the visualization include that some vaccines need yearly doses of vaccine? | 2/11 participants reported that some vaccines need yearly shots. 9/11 participants did not understand and found images confusing. |
|  | 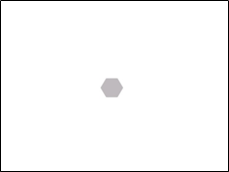 |  |  |  |
|  | 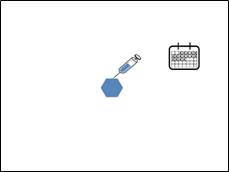 |  |  |  |
| 22. | 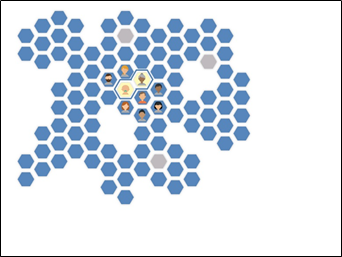 | When enough people are vaccinated, it provides a protective barrier known as community protection. | Did participants’ explanations of the visualization include that when enough people are vaccinated it provides a protective barrier known as community protection? | 10/11 participants reported that when enough people are vaccinated it provides a barrier known as community protection. |
| 23. | 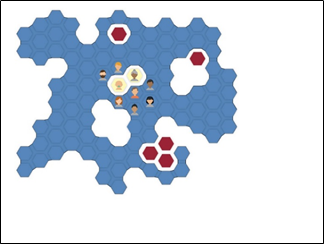 | The thick blue band is a protective barrier. | Did participants’ explanations of the visualization include that the thick blue band is a protective barrier? | 10/11 participants reported that the thick blue band is a protective barrier. |
| 24. | 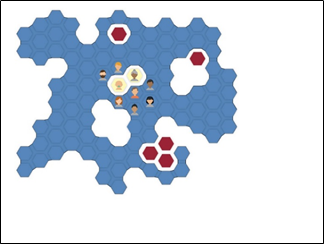 | The thick blue band around the older woman and child indicates that they are vulnerable people and the thick blue band protects them. | Did participants’ explanations of the visualization include that thick blue band around the older woman and child (vulnerable people) protecting them? | 10/11 participants reported that thick blue band around the older woman and child were vulnerable people and a thick blue band around them protects them. |
| 25. | 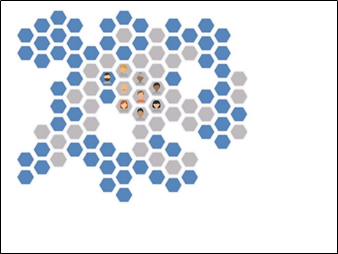 | When enough people are not vaccinated, the community protection weakens, and infection will spread. | Did participants’ explanations of the visualization include that when enough people are not vaccinated the community protection gets weak and infection will spread? | All participants (11/11) reported that when not enough people are vaccinated the community protection gets weak and infection will spread. |
|  | 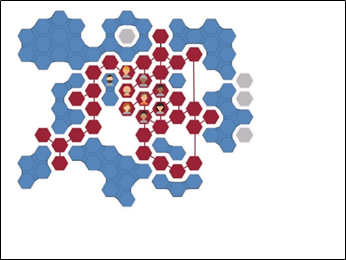 |  |  |  |
|  | 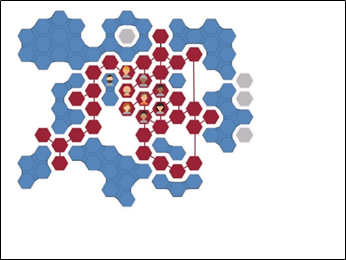 |  |  |  |

**Courtesy for images used:**

By CDC Public Health Image Library - This medium comes from the Centers for Disease Control and Prevention's Public Health Image Library (PHIL), with identification number #2121.Note: Not all PHIL images are public domain; be sure to check copyright status and credit authors and content providers.English | Slovenščina | +/−, Public Domain, https://commons.wikimedia.org/w/index.php?curid=973324

By Photo Credit: Cynthia GoldsmithContent Providers(s): CDC/ Dr. Terrence Tumpey - This medium comes from the Centers for Disease Control and Prevention's Public Health Image Library (PHIL), with identification number #8160.Note: Not all PHIL images are public domain; be sure to check copyright status and credit authors and content providers.English | Slovenščina | +/−Originally from en.wikipedia; description page is/was here., Public Domain, https://commons.wikimedia.org/w/index.php?curid=2544046

Photo Credit: Cynthia S. Goldsmith Content Providers(s): CDC/ Courtesy of Cynthia S. Goldsmith; William Bellini, Ph.D. - This medium comes from the Centers for Disease Control and Prevention's Public Health Image Library (PHIL), with identification number #8429. Note: Not all PHIL images are public domain; be sure to check copyright status and credit authors and content providers.https://commons.wikimedia.org/wiki/Measles#/media/File:Measles_virus.JPG

Created by Ricardo Moreira from the Noun Project (Needle)

Created by Steph Vasko from the Noun Project (Calendar)
