# Supplementary material for: A Web Application About Herd Immunity Using Personalized Avatars: Development Study
Source: J Med Internet Res. 2020 Oct 30;22(10):e20113. doi: 10.2196/20113 (PMC7665952; doi:10.2196/20113)
Supplement: Multimedia Appendix 10 [file jmir_v22i10e20113_app10.docx]

**Appendix 10: The communication goals set for the third iterative cycle of visualization (university sample):**

| **S.No** | **Design element or a concept** | **Message design elements intended to convey in the visualization** | **Our expectations or goals for each design element (desired interpretation and/or reaction)** | **What users reported when viewing these design elements (verbal feedback) (n= 49)** | **How users reacted to these design elements (psychophysiology) (n=49)** |
| --- | --- | --- | --- | --- | --- |
|  |  |  |  | **Question asked the participants:** Describe in your own words the visualization you just watched? |  |
| 1. | 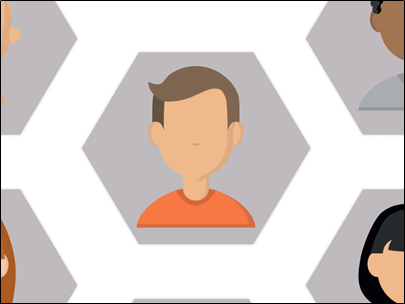 | The avatar represents the participant. (AvatarAppears) | Did this element generate visual attention?  When participants’ gaze enters the avatar element, do they demonstrate increased engagement, arousal and an optimal workload? | N/A (no verbal feedback data specific to this element). | Visual attention: 46/49 participants visually attended to this element. 3/49 participants did not visually attend to this element.  Arousal: Peaks in arousal detected among 27/49 participants during this element’s appearance. For 22/49 participants, no peak in arousal was detected.  Engagement across all 49 participants: Overall, participants were most likely in a high engagement state (median 0.49; IQR 0.10-0.81) during the appearance of this element.  Cognitive workload across all 49 participants: The median cognitive load across all participants was 0.58 (IQR 0.49-0.66) suggesting an overall optimal cognitive workload during the appearance of this element. |
| 2. | 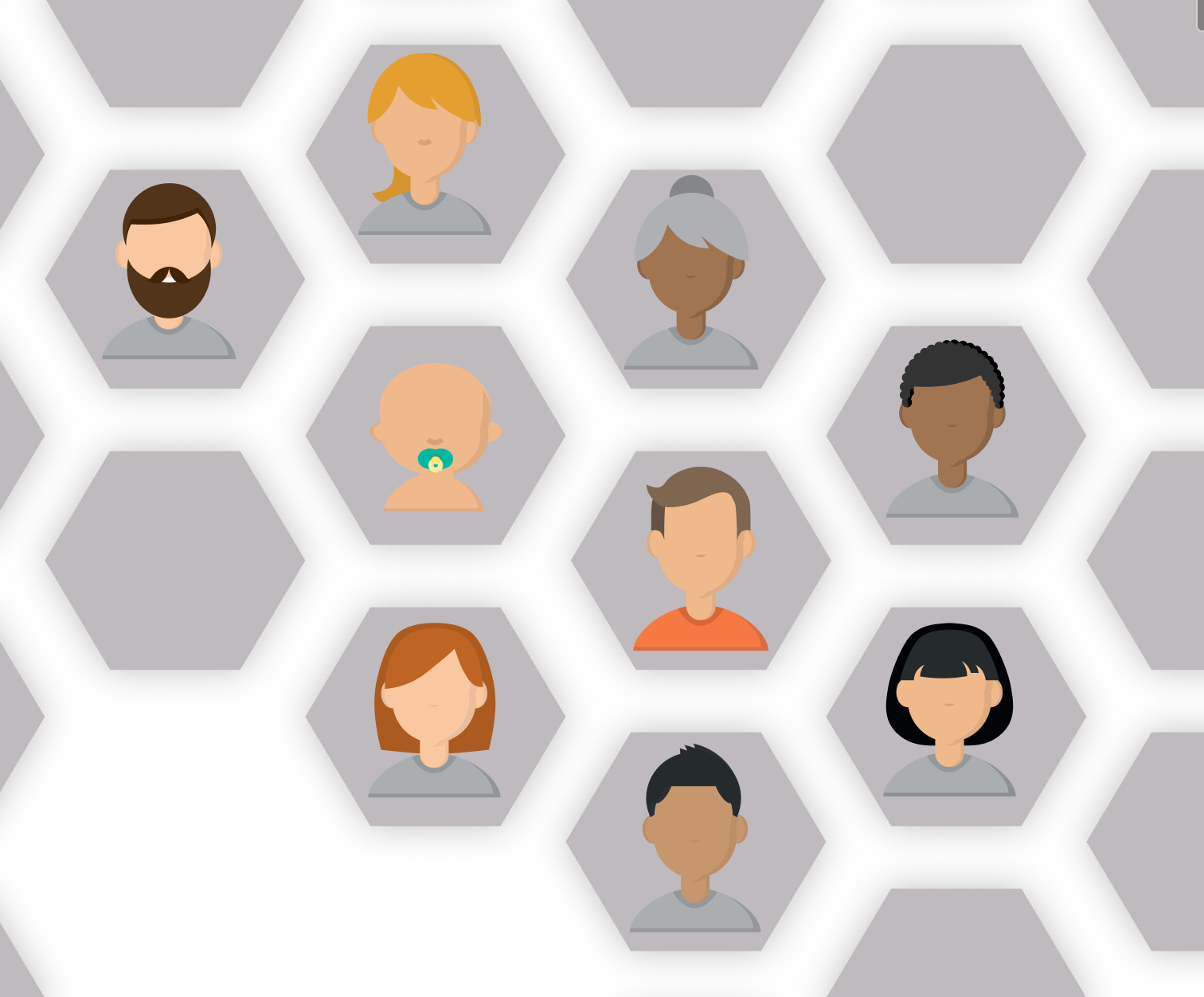 | People in clusters of hexagons represent a member of the participant’s community.  (Cluster) | Did participants report the concept of a community; for example, by referring to the community, referring to the group of people, etc?  Did this element generate increased visual attention?  When participants' gaze enters this element, do they demonstrate increased engagement, arousal & an optimal workload? | 27/49 participants reported the concept of a community for example, by referring to the community, group of people, etc. in their explanation; 22 participants did not report the concept of a community. | Visual attention: All participants (49/49) visually attended to this element.  Arousal: Peaks in arousal detected among 14/49 participants during this element’s appearance. For 35/49 participants, no peak in arousal was detected.  Engagement across all 49 participants: Overall, participants were most likely in a high engagement state (median 0.35; IQR 0.13-0.76) during the appearance of this element.  Cognitive workload across all 49 participants: The median cognitive load across all participants was 0.55 (IQR 0.44-0.63) suggesting an overall optimal cognitive workload during the appearance of this element. |
| 3. | 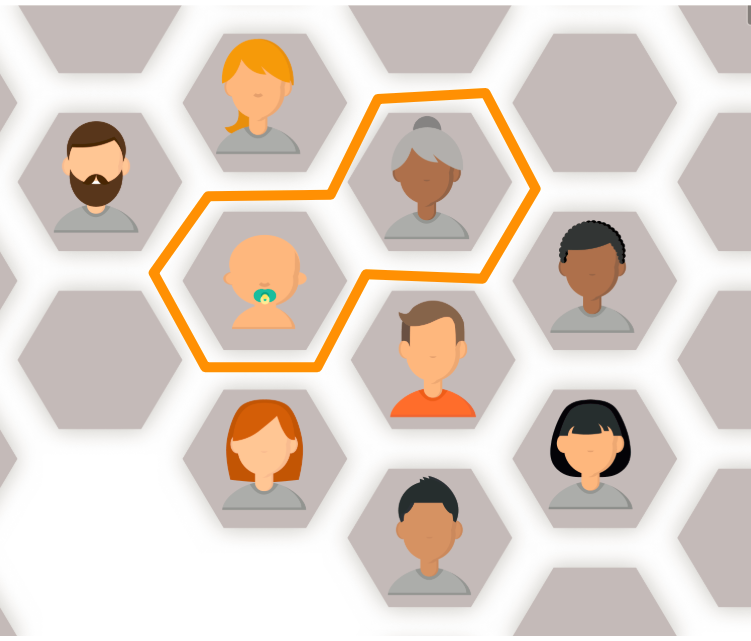 | Icon of an older woman and a baby represents vulnerable people or those with fragile immune systems (for example, cancer patients).  (Vulnerable Ppl) | Did participants report the concept of vulnerable people (e.g., by referring to older woman and a baby, people who have weak immune systems, or people who cannot get vaccinated)?  Did this element generate increased visual attention?  When participants’ gaze enters this element, do they demonstrate increased engagement, arousal & an optimal workload? | 31/49 participants reported the concept of vulnerable people (e.g., by referring to grandma and baby, people who have weak immune systems, or people who cannot get vaccinated); 18 participants did not report the concept of vulnerable people. | Visual attention: All participants (49/49) visually attended to this element.  Arousal: Peaks in arousal detected among 9/49 participants during this element’s appearance. For 40/49 participants, no peak in arousal was detected.  Engagement across all 49 participants: Overall, participants were most likely in a high engagement state (median 0.43; IQR 0.11-0.82) during the appearance of this element.  Cognitive workload across all 49 participants: The median cognitive load across all participants was 0.55 (IQR 0.44-0.62) suggesting an overall optimal cognitive workload during the appearance of this element. |
| 4. | 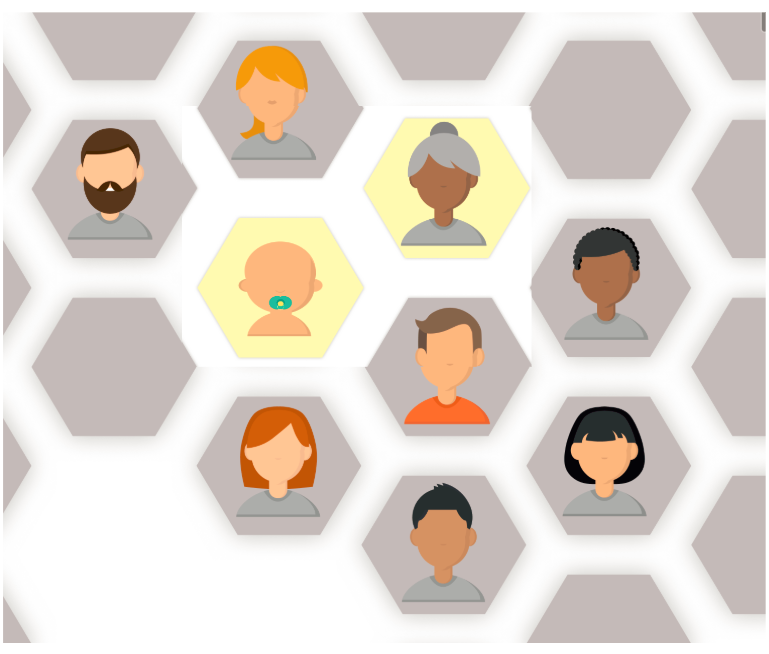 | Yellow colour behind “baby” and “an older woman” represents vulnerable people. (YellowVul) | Did this element generate increased visual attention?  When participants’ gaze enters this element, do they demonstrate increased engagement, arousal & an optimal workload? | N/A (no verbal feedback data specific to this element). | Visual attention: 47/49 participants visually attended to this element. 2/49 participants did not visually attend to this element.  Arousal: Peaks in arousal detected among 27/49 participants during this element’s appearance. For 22/49 participants, no peak in arousal was detected.  Engagement across all 49 participants: Overall, participants were most likely in a high engagement state (median 0.53; IQR 0.13-0.83) during the appearance of this element.  Cognitive workload across all 49 participants: The median cognitive load across all participants was 0.55 (IQR 0.43-0.63) suggesting an overall optimal cognitive workload during the appearance of this element. |
| 5. | When infection first entered the community. | (1stentryInfection) | Does the initial entry of the infection into the community generate visual attention?  When participants’ gaze enters this element, do they demonstrate increased engagement, arousal & an optimal workload? | N/A (no verbal feedback data specific to this element). | Visual attention: 29/49 participants visually attended to this element. 20/49 participants did not visually attend to this element.  Arousal: Peaks in arousal detected among 27/49 participants during this element’s appearance. For 22/49 participants, no peak in arousal was detected.  Engagement across all 49 participants: Overall, participants were most likely in a high engagement state (median 0.17; IQR 0.00-0.08) during the appearance of this element.  Cognitive workload across all 49 participants: The median cognitive load across all participants was 0.52 (IQR 0.30-0.64) suggesting an overall optimal cognitive workload during the appearance of this element. |
| 6. | 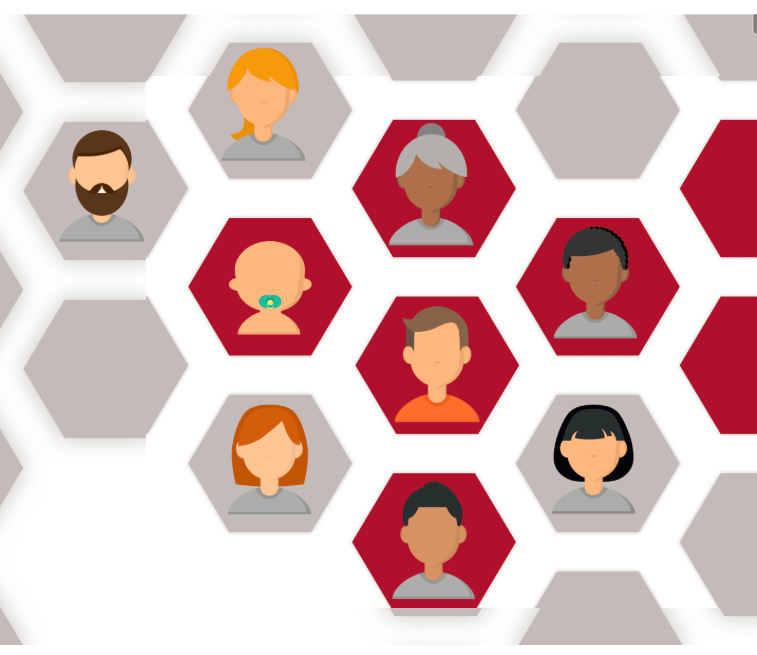 | Contagious disease can easily be passed on to others, especially to vulnerable people. (InfSpreadEasy) | Did participants report the concept that contagious diseases spread easily especially to vulnerable populations?  Did this element generate visual attention?  When participants’ gaze enters this element, do they demonstrate increased engagement, arousal & an optimal workload? | 9/49 participants reported the concept that contagious diseases spread easily especially to vulnerable populations; 40 participants did not report the concept that contagious diseases spread easily especially to vulnerable populations. | Visual attention: All participants (49/49) visually attended to this element.  Arousal: Peaks in arousal detected among 19/49 participants during this element’s appearance. For 30/49 participants, no peak in arousal was detected.  Engagement across all 49 participants: Overall, participants were most likely in a high engagement state (median 0.40; IQR 0.14-0.59) during the appearance of this element .  Cognitive workload across all 49 participants: The median cognitive load across all participants was 0.57 (IQR 0.51-0.63) suggesting an overall optimal cognitive workload during the appearance of this element. |
| 7. | 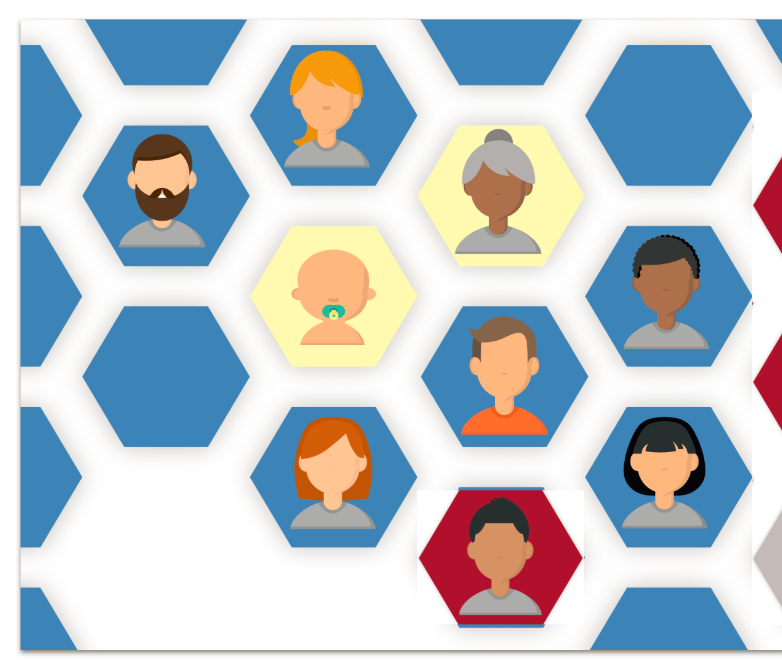  Red line cannot infect a vaccinated person. | People who are vaccinated are less likely to catch and pass on the disease.  (VaccinePreventSpread) | Did participants report the concept that vaccinated people are less likely to get the disease?  Does this element generate increased visual attention?  When participants’ gaze enters this element, do they demonstrate increased engagement, arousal & an optimal workload? | 37/49 participants reported the concept that vaccinated people are less likely to get the disease;12 participants did not report the concept that vaccinated people are less likely to get the disease. | Visual attention: 46/49 participants visually attended to this element. 3/49 participants did not visually attend to this element.  Arousal: Peaks in arousal detected among 19/49 participants during this element’s appearance. For 30/49 participants, no peak in arousal was detected.  Engagement across all 49 participants: Overall, participants were most likely in a high engagement state (median 0.28; IQR 0.04-0.62) during the appearance of this element.  Cognitive workload across all 49 participants: The median cognitive load across all participants was 0.52 (IQR 0.46-0.60) suggesting an overall optimal cognitive workload during the appearance of this element. |
| 8. | 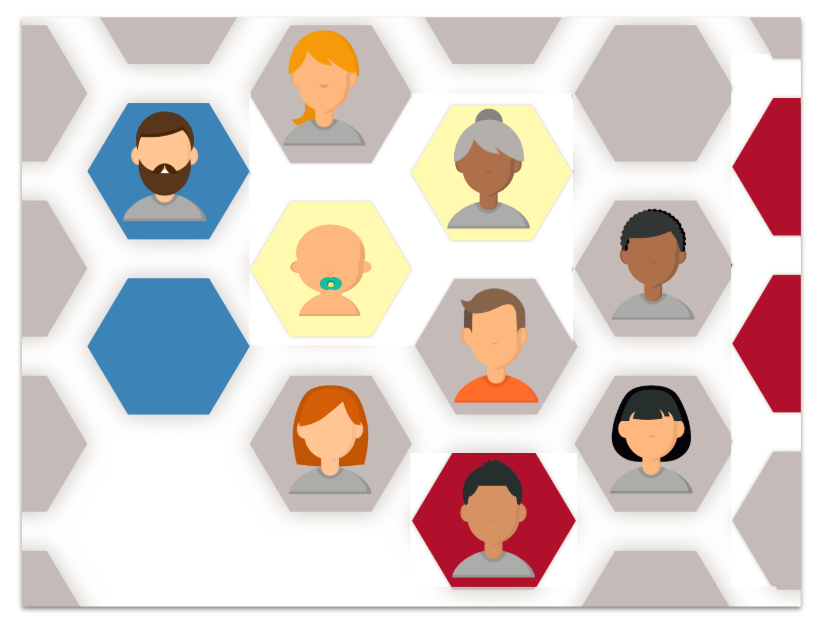 | Vaccines can wane over time.  (WaneOverTime) | Did participants report the concept that some vaccines can wane over time?  Did this element generate increased visual attention?  When participants’ gaze enters this element, do they demonstrate increased engagement, arousal & an optimal workload? | 2/49 participants reported the concept that some vaccines can wane over time; 47 participants did not report the concept that some vaccines can wane over time. | Visual attention: 46/49 participants visually attended to this element. 3/49 participants did not visually attend to this element.  Arousal: Peaks in arousal detected among 14/49 participants during this element’s appearance. For 35/49 participants, no peak in arousal was detected.  Engagement across all 49 participants: Overall, participants were most likely in a high engagement state (median 0.34; IQR 0.05-0.66) during the appearance of this element.  Cognitive workload across all 49 participants: The median cognitive load across all participants was 0.57 (IQR 0.48-0.66) suggesting an overall optimal cognitive workload during the appearance of this element. |
| 9. | 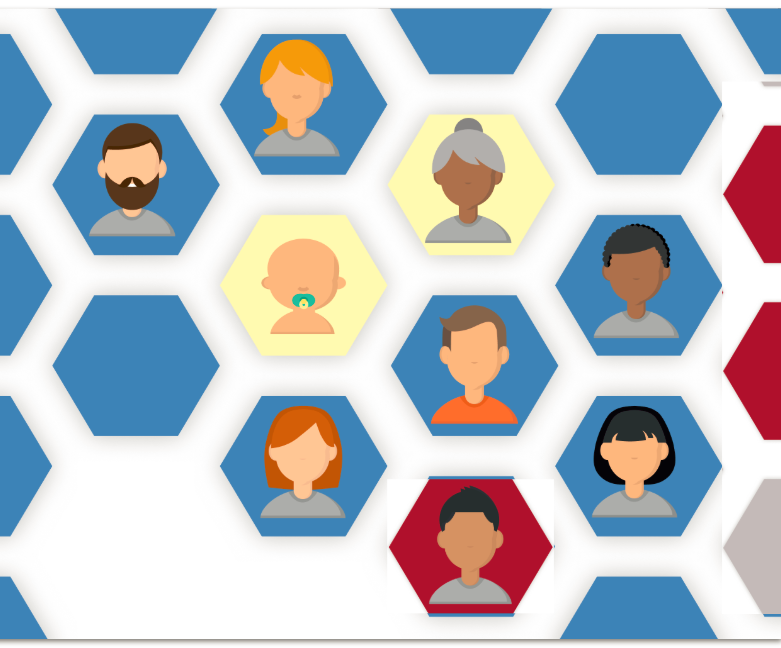 | Vaccines need to be updated. (VaccineUpdated) | Did participants report the concept that some vaccines need to be updated?  Did this element generate increased visual attention?  When participants’ gaze enters this element, do they demonstrate increased engagement, arousal & an optimal workload? | 1/49 participants reported the concept that some vaccines need to be updated; 48 participants did not report that some vaccines need to be updated. | Visual attention: 46/49 participants visually attended to this element. 3/49 participants did not visually attend to this element.  Arousal: Peaks in arousal detected among 14/49 participants during this element’s appearance. For 35/49 participants, no peak in arousal was detected.  Engagement across all 49 participants: Overall, participants were most likely in a high engagement state (median 0.49; IQR 0.16-0.75) during the appearance of this element.  Cognitive workload across all 49 participants: The median cognitive load across all participants was 0.49 (IQR 0.40-0.60) suggesting an overall optimal cognitive workload during the appearance of this element. |
| 10. | 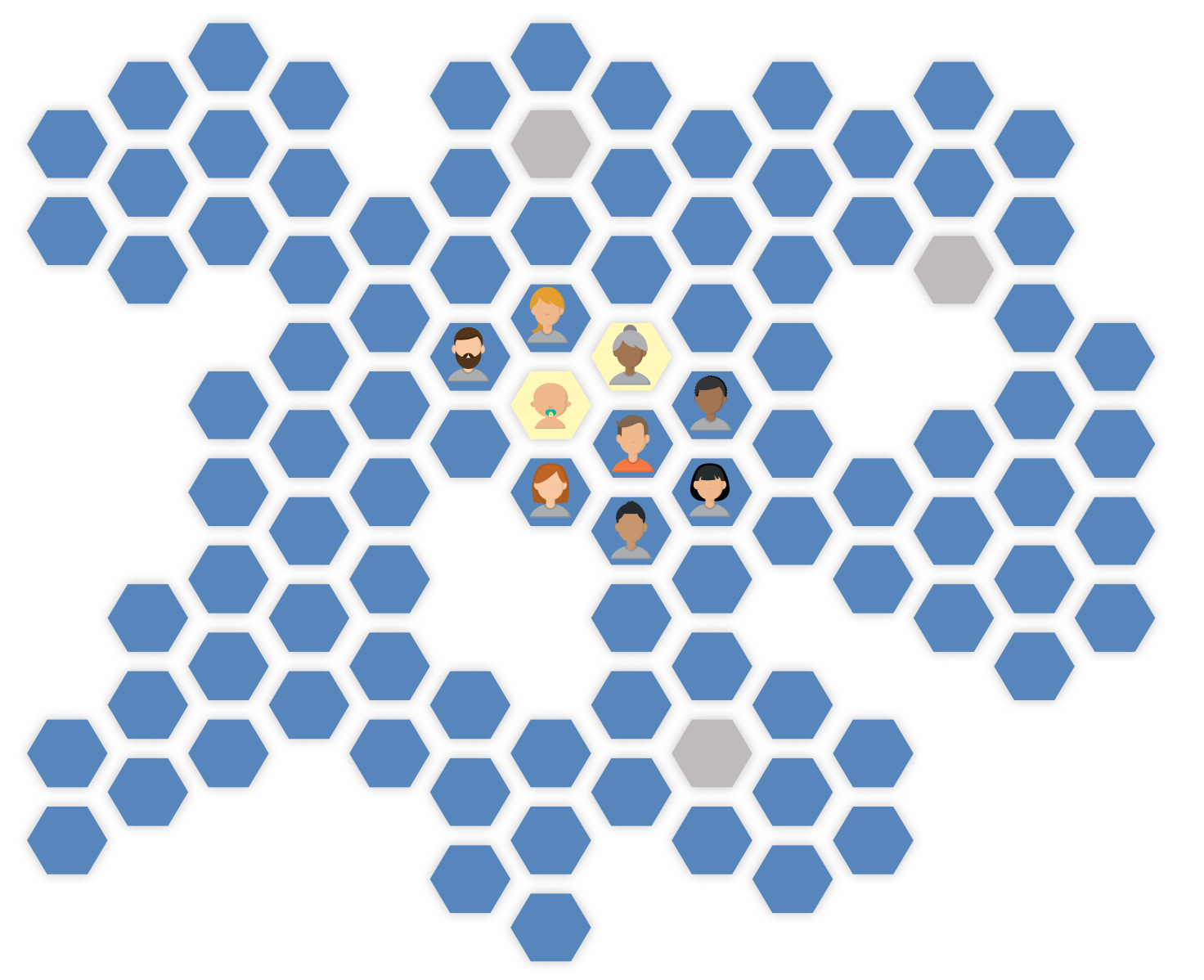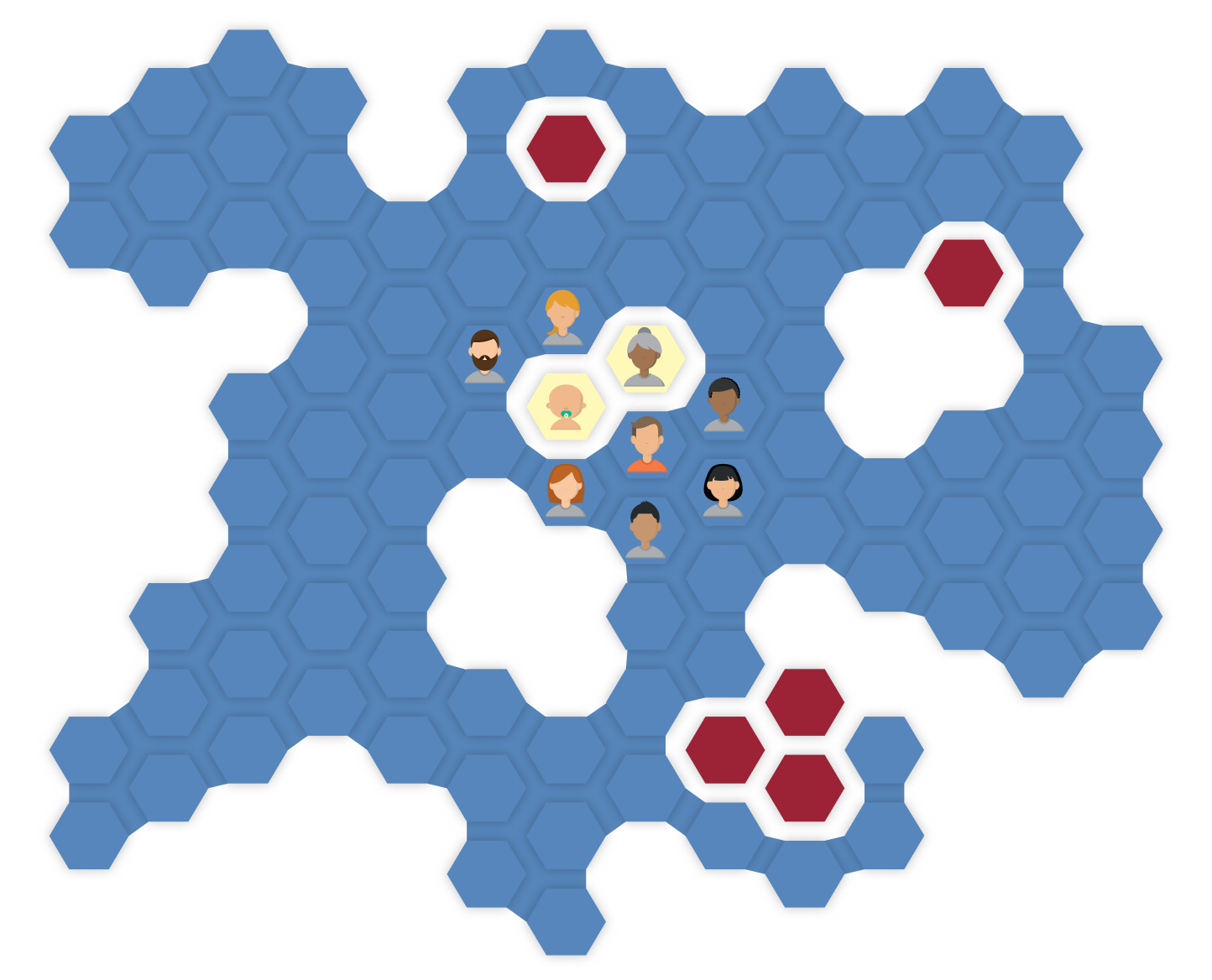 | When enough people are vaccinated, it creates a **protective barrier around** everyone in a community. (CreatProtectiveBarrier) | Did participants report the concept that vaccination of enough people in a community creates protective barrier?  Did this element generate increased visual attention?  When participants’ gaze enters this element, do they demonstrate increased engagement, arousal & an optimal workload? | 9/49 participants reported the concept that vaccination of enough people in a community creates a protective barrier; 40 participants did not report the concept that vaccination of enough people in a community creates a protective barrier. | Visual attention: All participants (49/49) visually attended to this element.  Arousal: Peaks in arousal detected among 15/49 participants during this element’s appearance. For 34/49 participants, no peak in arousal was detected.  Engagement across all 49 participants: Overall, participants were most likely in a high engagement state (median 0.25; IQR 0.10-0.68) during the appearance of this element.  Cognitive workload across all 49 participants: The median cognitive load across all participants was 0.54 (IQR 0.45-0.63) suggesting an overall optimal cognitive workload during the appearance of this element. |
| 11. | 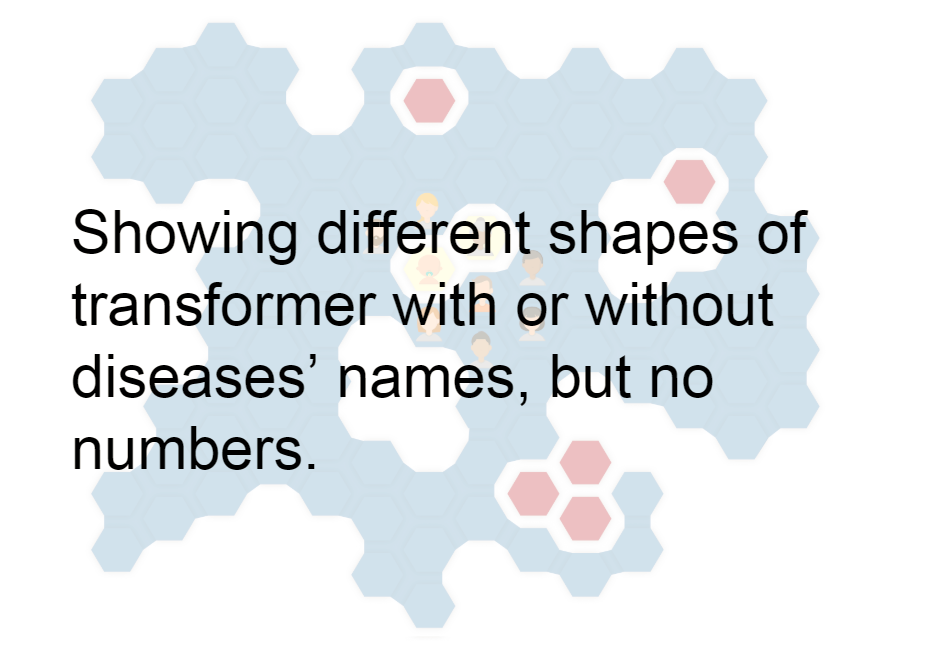 | Some diseases **spread faster than the others and need more people** to be vaccinated to create this protective barrier.  (SpreadfastneedMoreVac) | Did participants report the concept that some diseases spread faster than the others and need more people to be vaccinated to create the protective barrier?  Did this element generate increased visual attention?  When participants’ gaze enters this element, do they demonstrate increased engagement, arousal & an optimal.workload? | 4/49 participants reported the concept that some diseases spread faster than the others and need more people to be vaccinated to create the protective barrier; 45 participants did not report the concept that some diseases spread faster than the others and need more people vaccinated to create the protective barrier. | Visual attention: All participants (49/49) visually attended to this element.  Arousal: Peaks in arousal detected among 15/49 participants during this element’s appearance. For 34/49 participants, no peak in arousal was detected.  Engagement across all 49 participants: Participants were most likely in a high engagement state (median 0.34; IQR 0.09-0.69) during the appearance of this element.  Cognitive workload across all 49 participants: The median cognitive load across all participants was 0.58 (IQR 0.48-0.67) suggesting an overall optimal cognitive workload during the appearance of this element. |
| 12. | 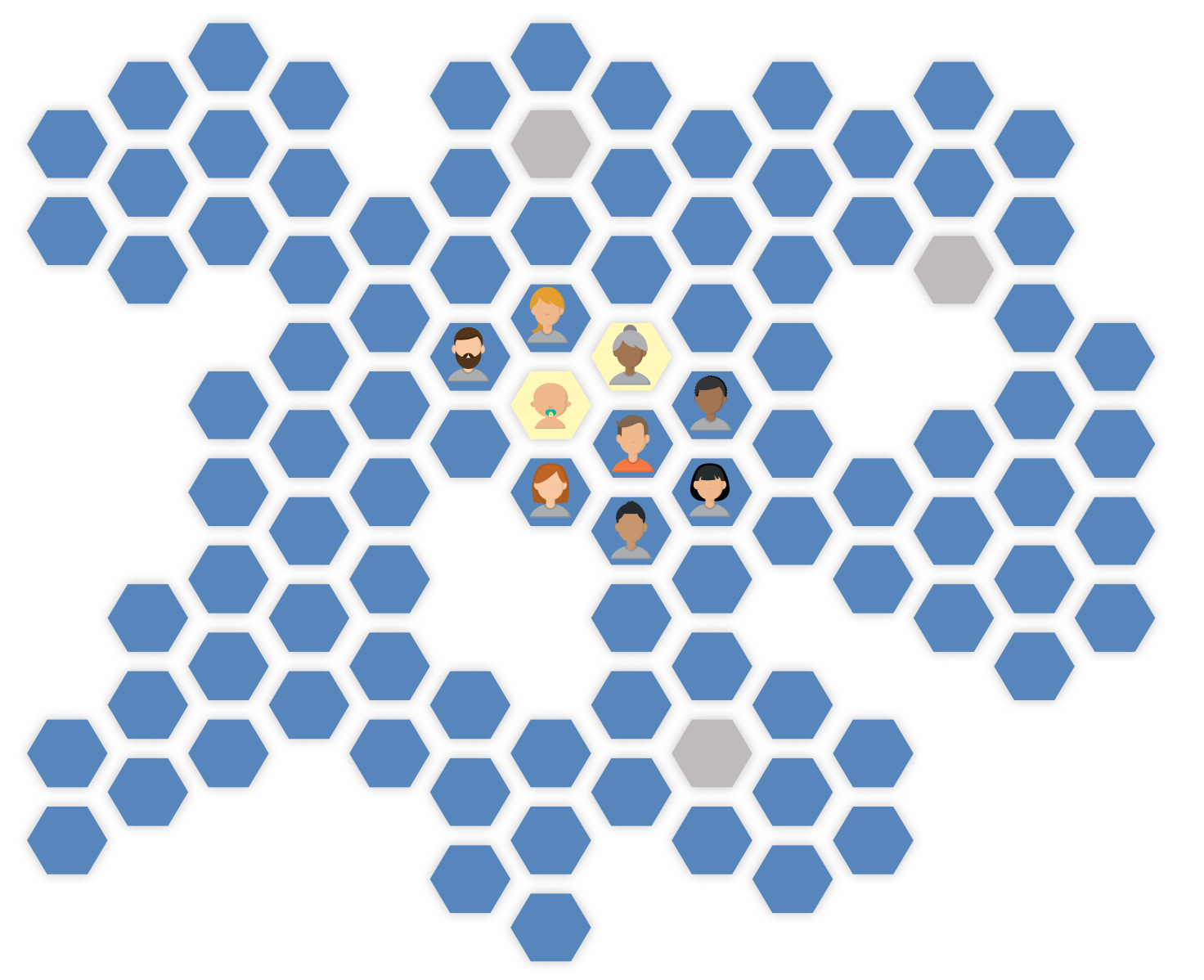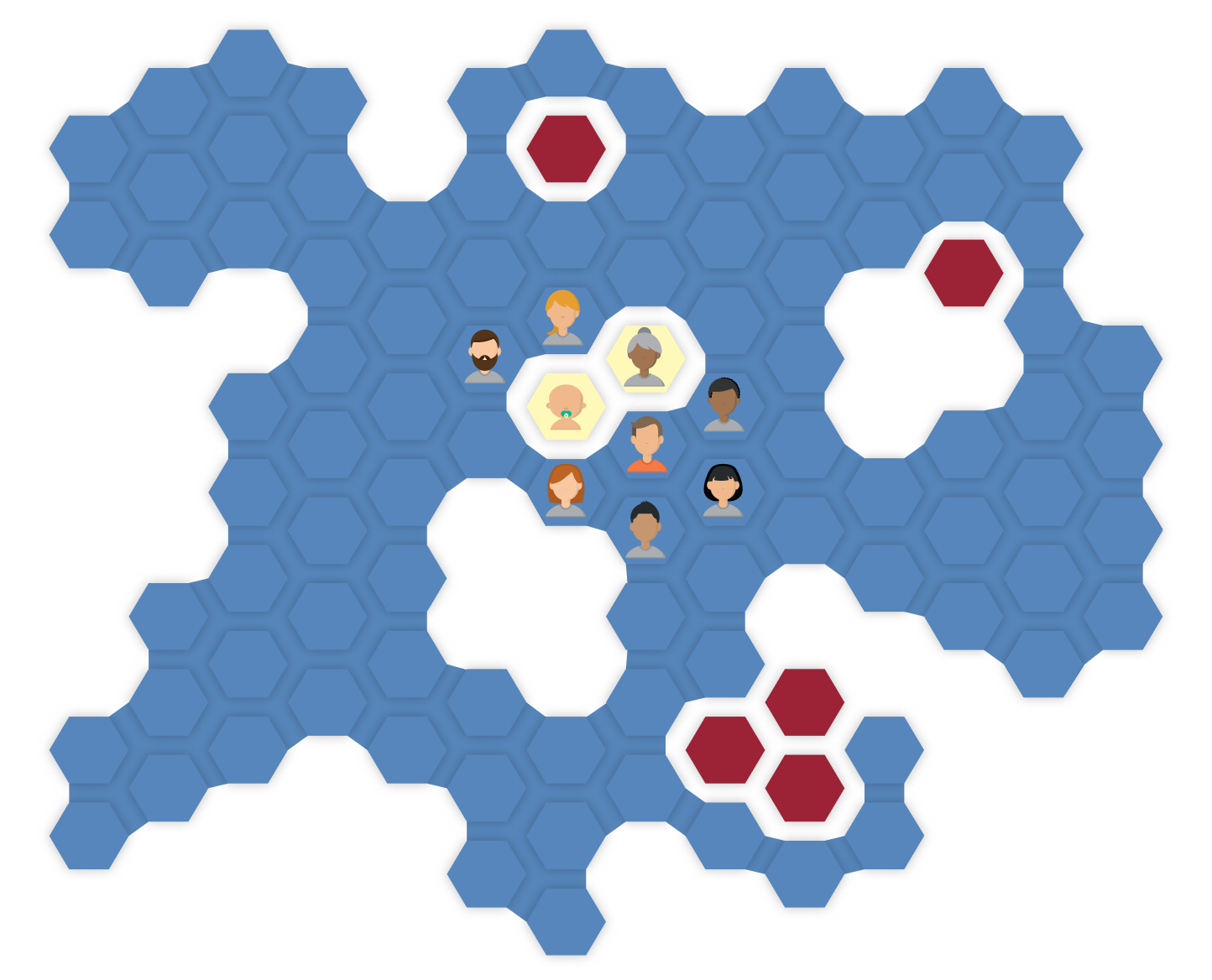 | Protective barrier around everyone in a community is **known as herd immunity or community immunity.**  (HerdImmunity) | Did participants report the concept of herd immunity or community immunity?  Does this element generate increased visual attention?  When participants’ gaze enters this element, do they demonstrate increased engagement, arousal & an optimal workload? | 12/49 participants reported the concept of herd immunity or community immunity;37 participants did not report the concept of herd immunity or community immunity. | Visual attention: All participants (49/49) visually attended to this element.  Arousal: Peaks in arousal detected among 9/49 participants during this element’s appearance. For 40/49 participants, no peak in arousal was detected.  Engagement across all 49 participants: Overall, participants were most likely in a high engagement state (median 0.37; IQR 0.09-0.79) during the appearance of this element.  Cognitive workload across all 49 participants: The median cognitive load across all participants was 0.55 (IQR 0.44-0.64) suggesting an overall optimal cognitive workload during the appearance of this element. |
| 13. | 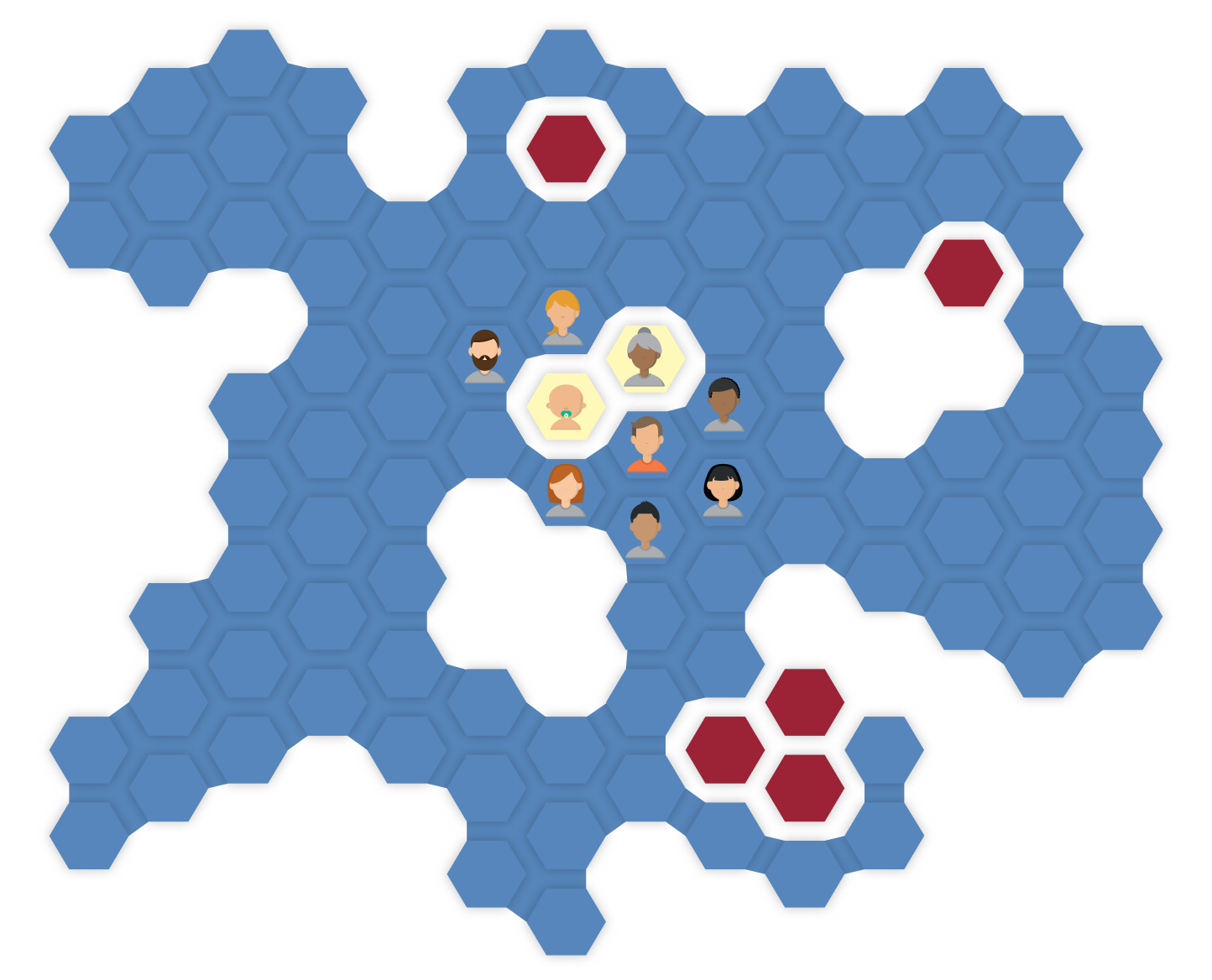 | Herd immunity or **community immunity safeguards everyone.** (HerdImmunitySafeguards) | Did participants report the concept that herd immunity or community immunity safeguards everyone?  Did this element generate increased visual attention?  When participants’ gaze enters this element, do they demonstrate increased engagement, arousal & an optimal workload? | 24/49 participants reported the concept that herd immunity or community immunity safeguards everyone; 27 participants did not report the concept that herd immunity or community immunity safeguards everyone. | Visual attention: All participants (49/49) visually attended to this element.  Arousal: Peaks in arousal detected among 9/49 participants during this element’s appearance. For 40/49 participants, no peak in arousal was detected.  Engagement across all 49 participants: Overall, participants were most likely in a high engagement state (median 0.38; IQR 0.18-0.70) during the appearance of this element.  Cognitive workload across all 49 participants: The median cognitive load across all participants was 0.63 (IQR 0.52-0.70) suggesting an overall optimal cognitive workload during the appearance of this element. |
| 14. | 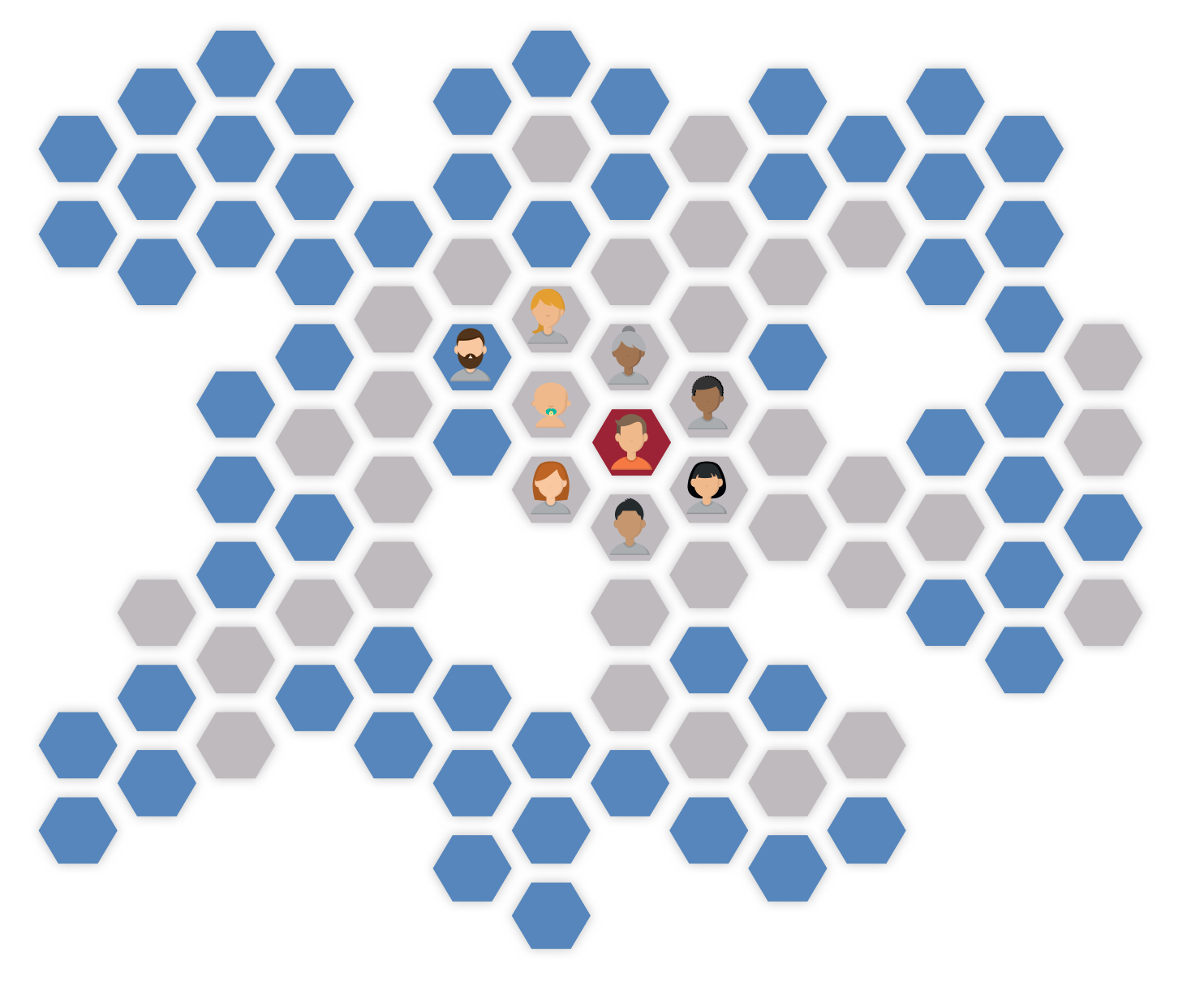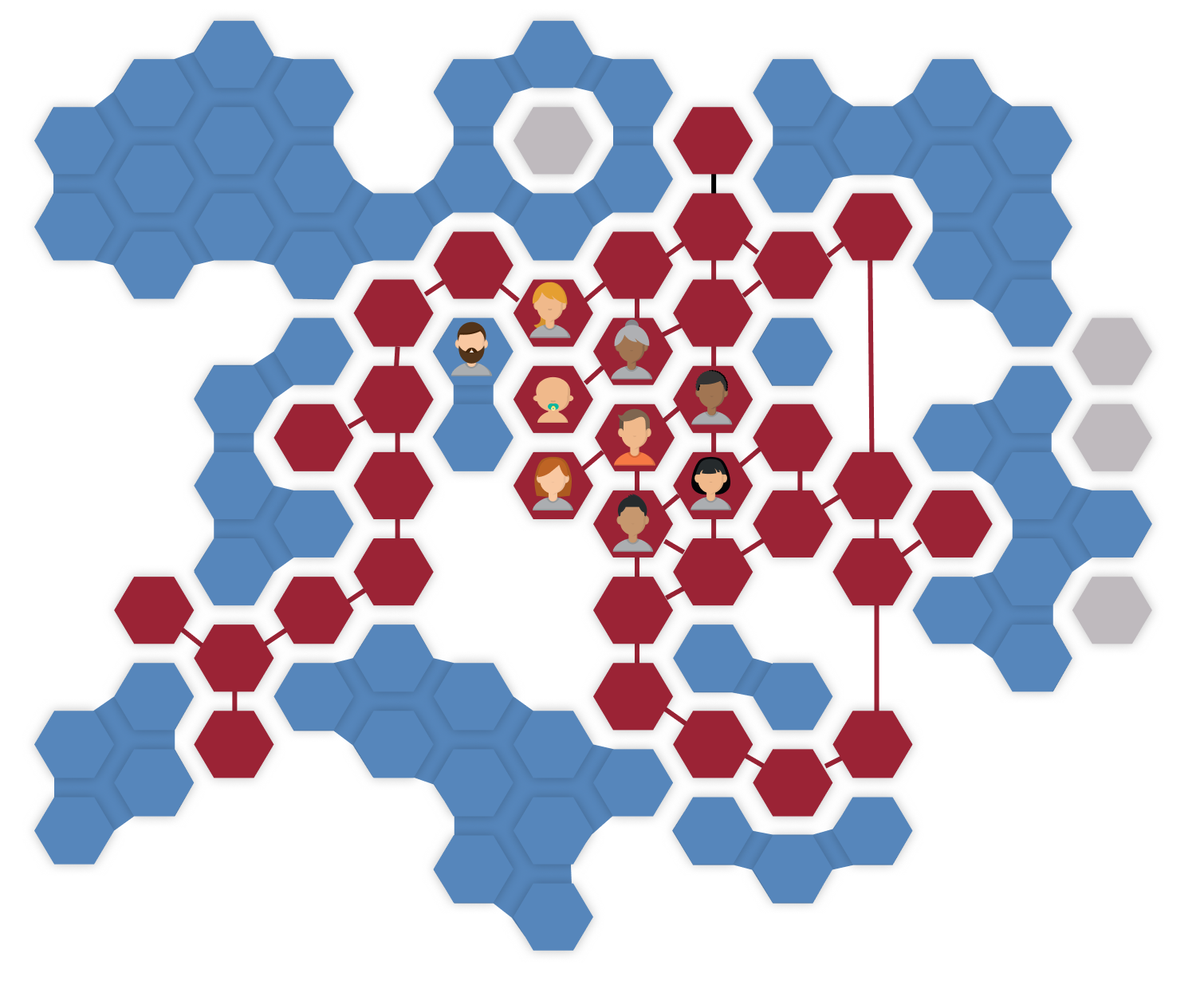 | When not enough people are vaccinated then  **infection** can easily **spread**.  (LessVacInfectionSpread) | Did participants report the concept that when less people are vaccinated, it leads to spread of infection easily and people can get sick?  Did this element generate increased visual attention?  When participants’ gaze enters this element, do they demonstrate increased engagement, arousal & an optimal workload? | 2/49 participants reported the concept that when not enough people are vaccinated, it leads to spread of infection easily and people can get sick; 47 participants did not report the concept that when not enough people are vaccinated, it leads to spread of infection easily and people can get sick. | Visual attention: All participants (49/49) visually attended to this element.  Arousal: Peaks in arousal detected among 9/49 participants during this element’s appearance. For 40/49 participants, no peak in arousal was detected.  Engagement across all 49 participants: Overall, participants were most likely in a high engagement state (median 0.36; IQR 0.09-0.63) during the appearance of this element.  Cognitive workload across all 49 participants: The median cognitive load across all participants was 0.55 (IQR 0.48- 0.64) suggesting an overall optimal cognitive workload during the appearance of this element. |
| 15. | 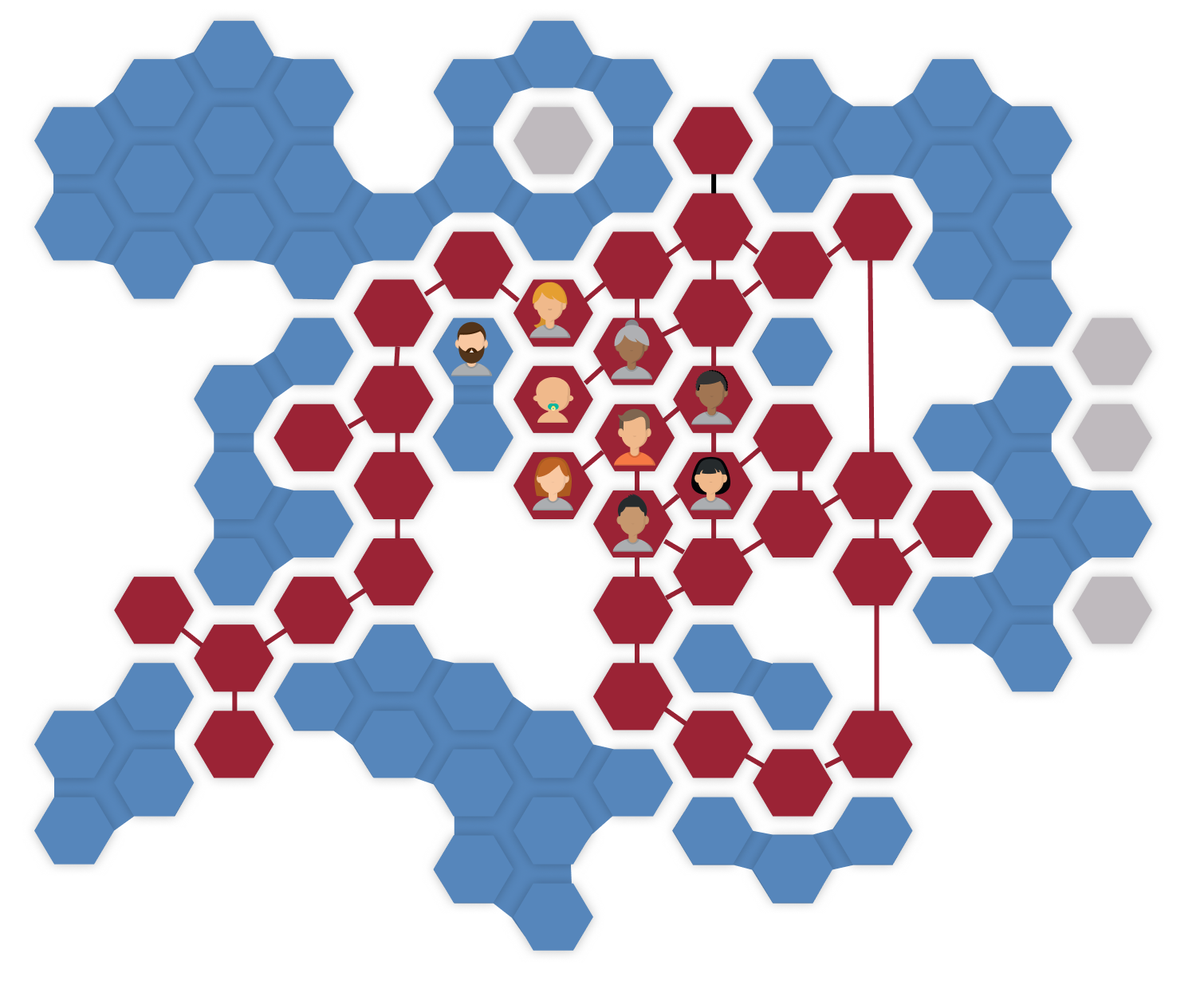 | Weakened community immunity can result in spread of **infection to** **vulnerable populations**. (InfectVulPopulation) | Did participants report the concept that weakened community immunity can result in spread of infection to vulnerable populations?  Does this element generate increased visual attention?  When participants’ gaze enters this element, do they demonstrate increased engagement, arousal & an optimal.workload? | 2/49 participants reported the concept that weakened community immunity can result in spread of infection to vulnerable populations; 47 participants did not report the concept that weakened community immunity can result in spread of infection to vulnerable populations. | Visual attention: 44/49 participants visually attended to this element. 5/49 participants did not visually attend to this element.  Arousal: Peaks in arousal detected among 9/49 participants during this element’s appearance. For 40/49 participants, no peak in arousal was detected.  Engagement across all 49 participants: Overall, participants were most likely in a high engagement state (median 0.38; IQR 0.08-0.71) during the appearance of this element.  Cognitive workload across all 49 participants: The median cognitive load across all participants was 0.58 (IQR 0.48-0.69) suggesting an overall optimal cognitive workload during the appearance of this element. |
| 16. | 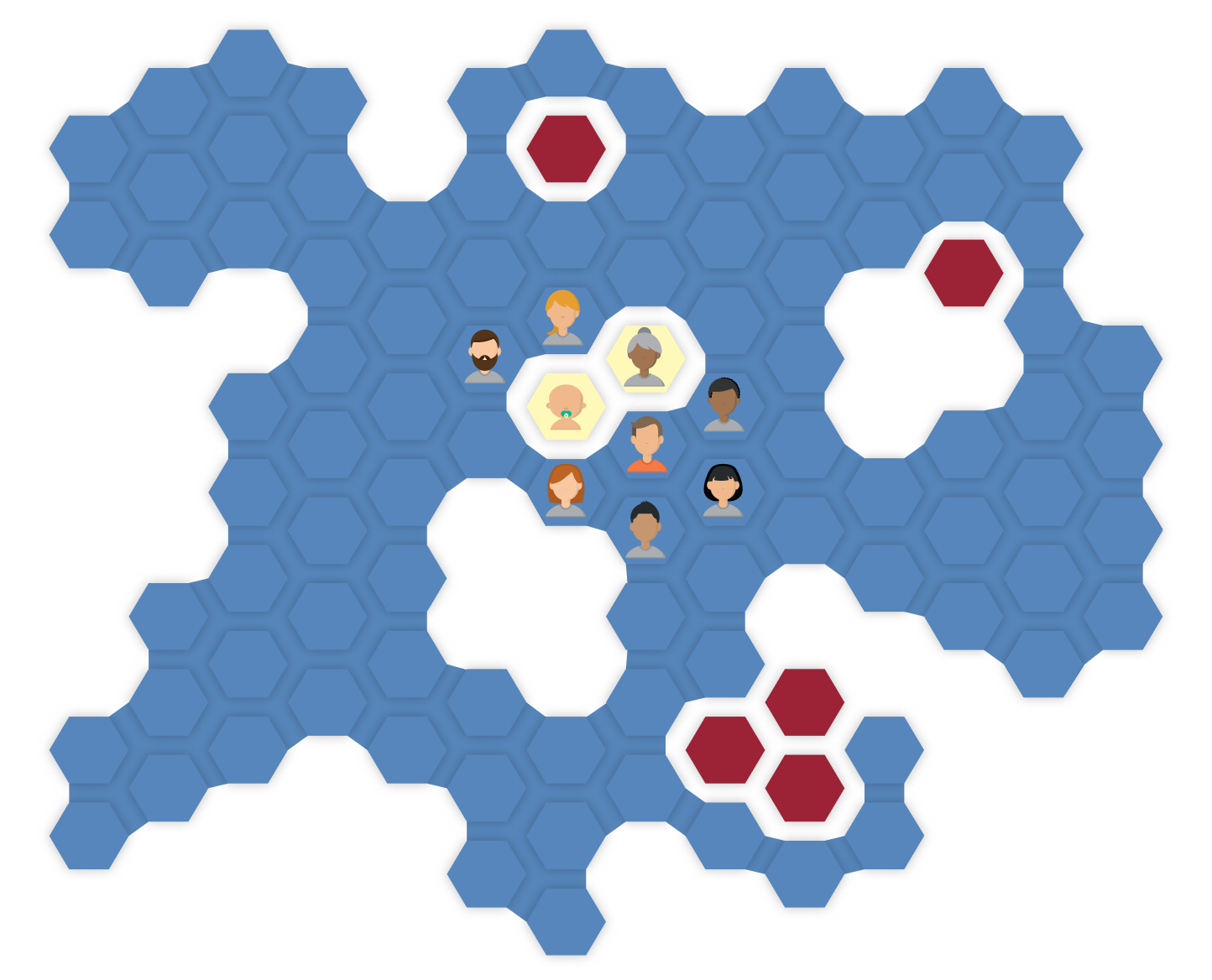 | **Thick blue band** around the older woman and baby represents community immunity protecting vulnerable populations. (ThickBlueBand) | Did participants report the concept that the thick blue band around an older woman and the baby represents community immunity protecting vulnerable populations?  Did this element generate increased visual attention?  When participants’ gaze enters this element, do they demonstrate increased engagement, arousal & an optimal workload? | 19/49 participants reported the concept that the thick blue band around an older woman and the baby represents community immunity protecting vulnerable populations; 30 participants did not report the concept that the thick blue band around an older woman and the baby represents community immunity protecting vulnerable populations. | Visual attention: 45/49 participants visually attended to this element.4/49 participants did not visually attend to this element.  Arousal: Peaks in arousal detected among 9/49 participants during this element’s appearance. For 40/49 participants, no peak in arousal was detected.  Engagement across all 49 participants: Overall, participants were most likely in a high engagement state (median 0.34; IQR 0.08-0.70) during the appearance of this element.  Cognitive workload across all 49 participants: The median cognitive load across all participants was 0.58 (IQR 0.47-0.66) suggesting an overall optimal cognitive workload during the appearance of this element. |
| 17. | 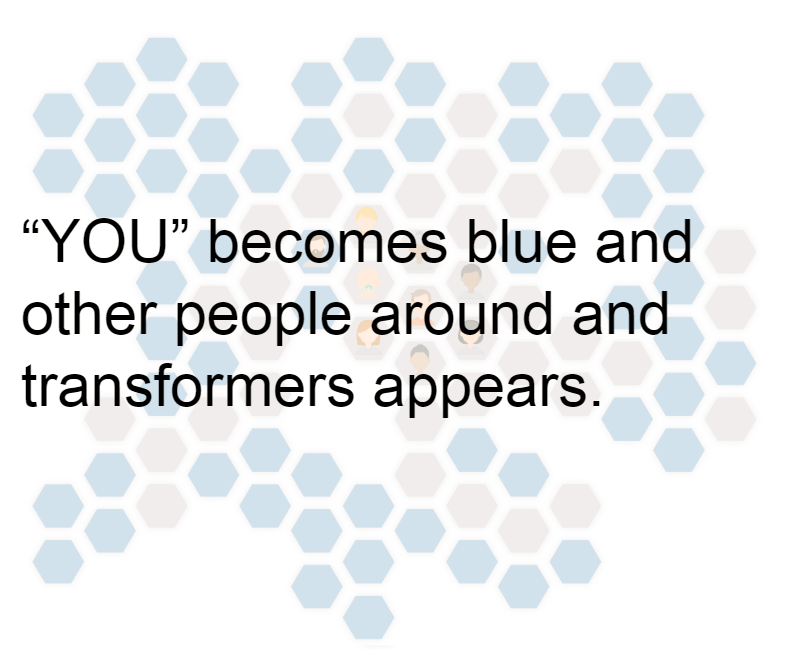 | **Your decision** to get vaccinated or not has an impact on other people in your community.  (YourDecision) | Did participants report the concept that the decision to vaccinate or not has an impact on other people in a community?  Did this element generate increased visual attention?  When participants’ gaze enters this element, do they demonstrate increased engagement, arousal & an optimal workload? | 19/49 participants reported the concept that the decision to vaccinate or not has an impact on other people in a community; 30 participants did not report the concept that the decision to vaccinate or not has an impact on other people in a community. | Visual attention: 46/49 participants visually attended to this element. 3/49 participants did not visually attend to this element.  Arousal: Peaks in arousal detected among 9/49 participants during this element’s appearance. For 40/49 participants, no peak in arousal was detected.  Engagement across all 49 participants: Overall, participants were most likely in a high engagement state (median 0.41; IQR 0.14-0.80) during the appearance of this element.  Cognitive workload across all 49 participants: The median cognitive load across all participants was 0.57 (IQR 0.50-0.63) suggesting an overall optimal cognitive workload during the appearance of this element. |
